# Use of transcriptomic profiling to identify candidate genes involved in *Polyporus umbellatus* sclerotial formation affected by oxalic acid

**DOI:** 10.1038/s41598-021-96740-7

**Published:** 2021-08-30

**Authors:** Yong-Mei Xing, Bing Li, Xu Zeng, Li-Si Zhou, Tae-Soo Lee, Min-Woong Lee, Xiao-Mei Chen, Shun-Xing Guo

**Affiliations:** 1 Key Laboratory of Bioactive Substances and Resource Utilization of Chinese Herbal Medicine, Ministry of Education, Institute of Medicinal Plant Development, Chinese Academy of Medical Sciences and Peking Union Medical College, No. 151, Malianwa North Road, Haidian District, Beijing, 100193 People’s Republic of China; 2 Division of Life Sciences, University of Incheon, Incheon, 22012 Korea; 3 Department of Life Science, Dongguk University, Seoul, 04620 Korea

**Keywords:** Biotechnology, Microbiology

## Abstract

*Polyporus umbellatus* is a precious medicinal fungus. Oxalic acid was observed to affect sclerotial formation and sclerotia possessed more medicinal compounds than mycelia. In this study, the transcriptome of *P. umbellatus* was analysed after the fungus was exposed to various concentrations of oxalic acid. The differentially expressed genes (DEGs) encoding a series of oxidases were upregulated, and reductases were downregulated, in the low-oxalic-acid (Low OA) group compared to the control (No OA) group, while the opposite phenomenon was observed in the high-oxalic-acid (High OA) group. The detection of reactive oxygen species (ROS) in *P. umbellatus* mycelia was performed visually, and Ca^2+^ and H_2_O_2_ fluxes were measured using non-invasive micro-test technology (NMT). The sclerotial biomass in the Low OA group increased by 66%, however, no sclerotia formed in the High OA group. The ROS fluorescence intensity increased significantly in the Low OA group but decreased considerably in the High OA group. Ca^2+^ and H_2_O_2_ influx significantly increased in the Low OA group, while H_2_O_2_ exhibited efflux in the High OA group. A higher level of oxidative stress formed in the Low OA group. Different concentrations of oxalic acid were determined to affect *P. umbellatus* sclerotial formation in different ways.

## Introduction

*Polyporus umbellatus* (Pers.) Fr. is a valuable medicinal fungus, and the sclerotium has been used as a diuretic and antidote for more than two thousand years in China. Steroids and polysaccharides are two principal groups of chemicals present in *P. umbellatus* sclerotia^1^. Among these bioactive components, sterone compounds exhibited nephroprotective activity on renal interstitial fibrosis against aristolochic acid- or adenine-induced nephrotoxicity in rats^2^. In a previous study in our laboratory, an HPLC method was performed for the simultaneous quantitative determination of five sterone compounds, including polyporusterone A and polyporusterone B, which were isolated from the wild sclerotia of *P. umbellatus*. HPLC fingerprinting was employed to compare the different components of the sclerotia collected in the wild, and the mycelia and sclerotia produced under artificial conditions^3^. The results showed that the cultured sclerotia possessed a spectrum similar to that of the wild sclerotia, while the cultured mycelia shared only the chromatographic peak of a few sterone compounds with the wild sclerotia; thus, it is necessary to perform experiments to effectively induce sclerotia directly from hyphae. Under natural conditions, mycelia, sclerotia, basidiospores and fruiting bodies represent the four stages of the lifecycle in *P. umbellatus*. When *P. umbellatus* sclerotial formation occurs directly from mycelia under artificial conditions in the laboratory, three distinct stages, that is, sclerotial initiation (SI), sclerotial development (SD) and sclerotial maturation (SM) have been demonstrated^4,5^. It is important to produce sclerotia from *P. umbellatus* mycelia to yield medicinal-quality compounds.

Oxalic acid (OA) plays different roles in fungi, plants or animals. For example, it played dual opposing roles in *Sclerotinia* pathogenesis, as it initially inhibited ROS, but later promoted ROS production in the host plant^6^. Although oxalic acid is traditionally considered to be an antioxidant in fungi^5^, this gives rise to our interest in investigating the effects of exogenous oxalic acid on *P. umbellatus* sclerotial development. Based on the previous study, we are trying to find whether exogenous oxalic acid especially the minimum and the most suitable concentration promote and the concentration of the oxalic acid just completely inhibit *P. umbellatus* sclerotial formation, thus different concentrations of oxalic acid on sclerotial biomass is further studied in detail in the present study.

ROS play essential roles in redox processes, and H_2_O_2_ is the most important source of ROS. Ca^2+^ dynamics and signal transduction also participate in many cellular processes^7^. According to a previous study, calcium antagonists inhibited *P. umbellatus* sclerotial formation^8^. Calcium ions and ROS are generally stimulated by each other and function coordinately during signal transduction^9^. During the stress response, Ca^2+^ influx is induced, which activates ROS production in plants^10^. However, detailed molecular information regarding oxidative stress, the calcium signaling pathway and the real-time dynamic changes in Ca^2+^ and H_2_O_2_, as well as the correlation between H_2_O_2_ and calcium ions fluxes during oxalic acid treatment in *P. umbellatus* sclerotial formation, needs to be obtained.

To better understand the molecular process of *P. umbellatus* sclerotial formation under different concentrations of oxalic acid, we performed RNA-seq transcriptomic analysis. In this study, DEGs of the mycelia in *P. umbellatus* subjected to exogenous oxalic acid were described. After cultivation for 30 d, the sclerotia in the Low OA group and the control (No OA) group entered the SD stage, which was in a relatively stable period or stationary phase during *P. umbellatus* sclerotial morphogenesis^4,5^. At this time interval, the mycelia in the High OA group with exuberant mycelia also grew stably and had no sclerotia. In the SI stage of *P. umbellatus* sclerotial development, small interwoven hyphae could be seen^4^, but the structure of the sclerotia had not fully matured; however, in the SM phase, the sclerotia had entered the ageing and dormancy period, and the samples in this stage were not suitable for transcriptomic sequencing and analysis. Therefore, the samples of *P. umbellatus* mycelia that had been cultivated for 30d were chosen for transcriptomic analysis. As an emerging detection method, non-invasive micro-test technology (NMT) has been widely used in many fields of research, such as recording H^+^, Ca^2+^, NO_3_^−^ and H_2_O_2_ fluxes in plant systems^11,12^. To confirm whether different concentrations of oxalic acid affected intracellular ROS in *P. umbellatus* mycelia, a fluorescence staining assay was conducted in this study. Furthermore, the real-time Ca^2+^ and H_2_O_2_ fluxes in *P. umbellatus* mycelia in different groups after cultivation for 30 d were detected by NMT. Thus, the aims of this study were to identify the DEGs involved in oxidative stress and calcium signal transduction as well as the profiles of their expression patterns based on high-throughput data. On the other hand, the correlation of the fluxes of H_2_O_2_ and calcium ions was also analysed by Pearson correlation analysis. This study attempts to provide genetic evidence for the involvement of oxalic acid during *P. umbellatus* sclerotial formation.

## Results

### Colony morphology and sclerotial biomass of P. umbellatus under exogenous different concentrations of oxalic acid

**Figure 1:**
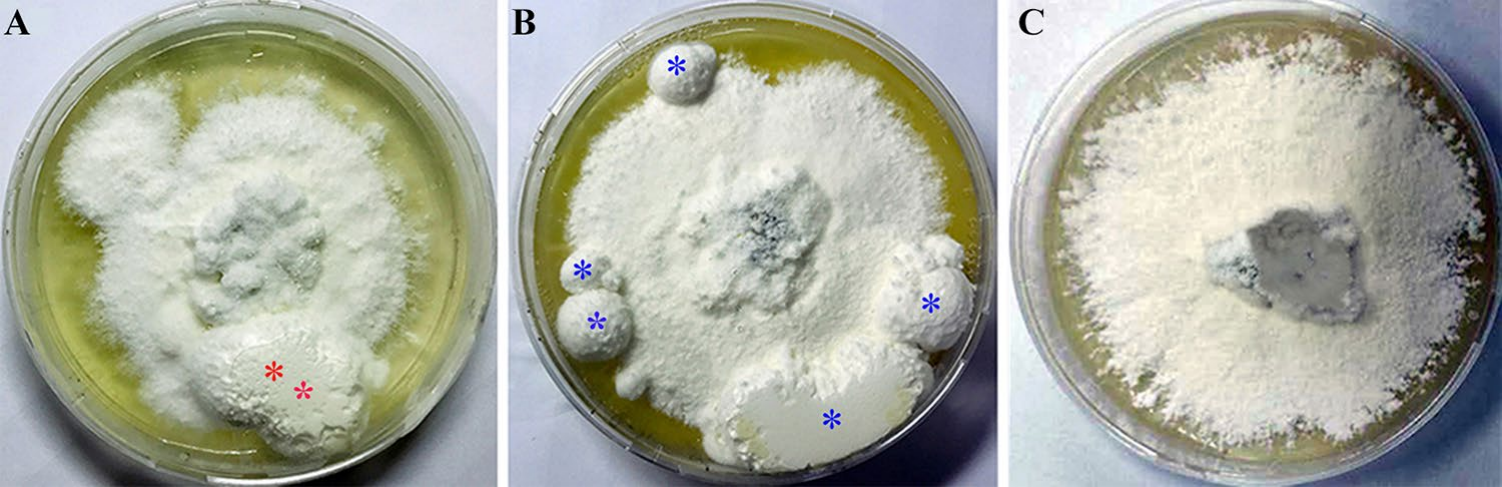
Colony morphology of *P. umbellatus* under exogenous different concentrations of oxalic acid. (**A**), (**B**) and (**C**) represent the colony morphological characteristics of *P. umbellatus* mycelia in the No OA, Low OA and High OA groups after cultivation for 30 d respectively. Red or blue asterisks represent round, oval or irregular sclerotia in the No OA and Low OA groups.

After cultivation for 30 d, the sclerotia with red asterisks* in the No OA group (Fig. 1A) and blue asterisks in the Low OA group (Fig. 1B) entered the SD stage, and interwoven mycelia with round, oval or irregular sclerotia distributed on the edge of the petri dishes, as depicted in Fig. 1A,B. From the concentrations of exogenous oxalic acid below 1 mg/mL, it was observed that oxalic acid at 0.05 mg/mL served as the most suitable concentration in *P. umbellatus* sclerotial formation (Fig. 1B), compared to that of the control group (Fig. 1A). *P. umbellatus* sclerotial biomass in the Low OA group increased significantly (*P* < 0.05). In addition, the minimum concentration of oxalic acid that completely inhibited *P. umbellatus* sclerotial development was 1.10 mg/mL (Fig. 1C, Table 1); thus, no sclerotia formed in the High OA group. With the increase of oxalic acid concentrations at certain ranges, *P. umbellatus* sclerotia was promoted or inhibited.

**Table 1:** Effect of exogenous oxalic acid on *P. umbellatus* sclerotial differentiation.

Exogenous oxalic acid (mg/mL) (n = 30)	Sclerotial fresh weight (g/20 g substrate)
0 (control)	1.62 ± 0.06^a^
0.005	1.65 ± 0.06^a^
0.010	1.67 ± 0.07^a^
0.050	2.69 ± 0.09^b^
0.100	1.75 ± 0.09^c^
1.100	0

The values were presented as the means ± SD from at least three independent experiments, with 30 replicates of each group. Significant differences were determined using the Student–Newman–Keuls method, and the values followed by different letters (a, b, and c) are significantly different (*P* < 0.05).

### *Transcriptome sequencing and *de novo* assembly and sequence annotation*

To obtain the *P. umbellatus* mycelial transcriptomic expression profiles following treatment with oxalic acid, two libraries from a low-concentration oxalic acid (Low OA) group and a high-concentration oxalic acid (High OA) group and one library from a no-oxalic-acid (No OA) control group were constructed. Illumina sequencing data of *P. umbellatus* could be obtained in the NCBI BioProject and the Sequence Read Archive (SRA) database with the ID PRJNA669949. In total, 284,190,976 raw reads were generated (Supplementary Table 1), and 276,265,498 clean reads remained after the adaptor sequences, ambiguous nucleotides and low-quality sequences were removed. The assembly of clean reads included 22,523 unigenes in the range of 201–19,256 bp, and the numbers of assembled unigenes with different interval lengths are shown (Supplementary Fig. 1A).

In total, 22,523 nonredundant unigenes were subjected to similarity analysis according to the 7 public databases. Among these unigenes, 12,864 (57.11%) had the highest similarity matches in the NT database followed by 11,236 (49.88%) unigenes in the SwissProt database. Specifically, 10,914, 10,645, 10,571, 6934 and 4063 unigenes were functionally annotated in the other five databases (NR, GO, PFAM, KOG and KO), accounting for 48.45%, 47.26%, 46.93%, 30.78% and 18.03%, respectively, as shown in Supplementary Table 2.

According to GO, 10,645 unigenes were grouped into three major functional ontologies, including biological process, cellular component and molecular function (Supplementary Fig. 1B).

### Identification of DEGs in the mycelia of P. umbellatus treated with different concentrations of oxalic acid

Due to the analysis of the DEGs, RPKM values were calculated, and the Pearson’s correlation coefficients (R^2^) were high between the three biological replicates in the same group, ranging from 0.902 to 1. The R^2^ of the samples between the different groups ranged from 0.705 to 0.816 (Supplementary Fig. 1C). The DEGs that met the criteria (adjusted *P* value < 0.05 and |log2 fold change|> 1) between the Low OA and the No OA group, the High OA and the No OA group were analysed, and the DEGs with higher expression levels in the Low OA and High OA groups were classified as ‘upregulated’, while those with lower expression levels in both groups were classified as ‘downregulated’ (Supplementary Fig. 1D). There were 725 DEGs between the High OA and the control group, and among them, 299 were upregulated and 426 were downregulated. There were 459 DEGs between the Low OA and the control group and among them, 231 were upregulated and 228 were downregulated (Supplementary Fig. 1D). According to the Venn diagram (Supplementary Fig. 1E), there were 231 common DEGs in both of the compared groups, and there were 59 DEGs upregulated and 47 downregulated in both cases. There were 44 DEGs upregulated in High OA compared with No OA but downregulated in Low OA compared with No OA. There were 81 DEGs upregulated in Low OA compared with No OA but downregulated in High OA compared with No OA.

### DEGs related to oxidative stress, calcium signaling and energy metabolism

In previous studies, it was demonstrated that *P. umbellatus* sclerotial formation was closely associated with oxidative stress and that calcium channel blockers and calmodulin inhibitors inhibited *P. umblelatus* sclerotial development; therefore, we were strongly interested in the DEGs related to oxidative stress and the calcium signaling pathway^5,8^. In comparison to that of the control (No OA) group, DEGs encoding such oxidases as NADPH oxidase, alcohol oxidase, cytochrome c oxidase (subunits 1, 2 and 3), NADH dehydrogenase subunit 1, NADH dehydrogenase subunit 4, glycerol 2-dehydrogenase were upregulated in the Low OA group but were downregulated in the High OA group. Meanwhile, DEGs encoding such reductases as aldo/keto reductase and nitrite reductase were downregulated in the Low OA group but upregulated in the High OA group. Also, specific DEGs, such as cytochrome c reductase, ribonucleotide reductase alpha subunit and quinone reductase, were all upregulated, while such enzymes as galactose oxidase, sorbitol dehydrogenase, succinate dehydrogenase and alanine dehydrogenase, were all downregulated in the High OA group in comparison to that of the No OA group. Similarly, specific DEGs in the Low OA group, such as NAD-dependent glutamate dehydrogenase, glutamate dehydrogenase and NAD-aldehyde dehydrogenase, were all upregulated in the Low OA group compared to the control group (Supplementary Table 3). The same DEGs, *c10001_g1*, *c9468_g4*, *c43_g1*, *c9143_g1*, *c3657_g1*, *c524_g1* and *c5285_g1*, were upregulated in the Low OA group but downregulated in the High OA or downregulated in the Low OA group while upregulated in the High OA group (Supplementary Table 3).

The DEGs related to calcium signaling (i.e. calmodulin / calcium motif containing genes) such as *c2043_g1 c8739_g2* and *c3257_g1* were all upregulated in the Low OA group but downregulated in the High OA group (Supplementary Table 3).

In comparison to that of the control group, *c4703_g1* was upregulated in the Low OA group but downregulated in the High OA group. In addition, *c7209_g1* and *c6742_g2*, were significantly highly expressed in the Low OA group, while *c9487_g1*, *c9511_g1* and *c9756_g1* were all downregulated in the High OA group, in comparison to the control group (Supplementary Table 3). The unigene sequences of the DEGs were listed in Supplementary Table 4.

### ROS content and the fluorescence intensity of ROS in P. umbellatus mycelia were affected by different concentrations of oxalic acid

**Figure 2:**
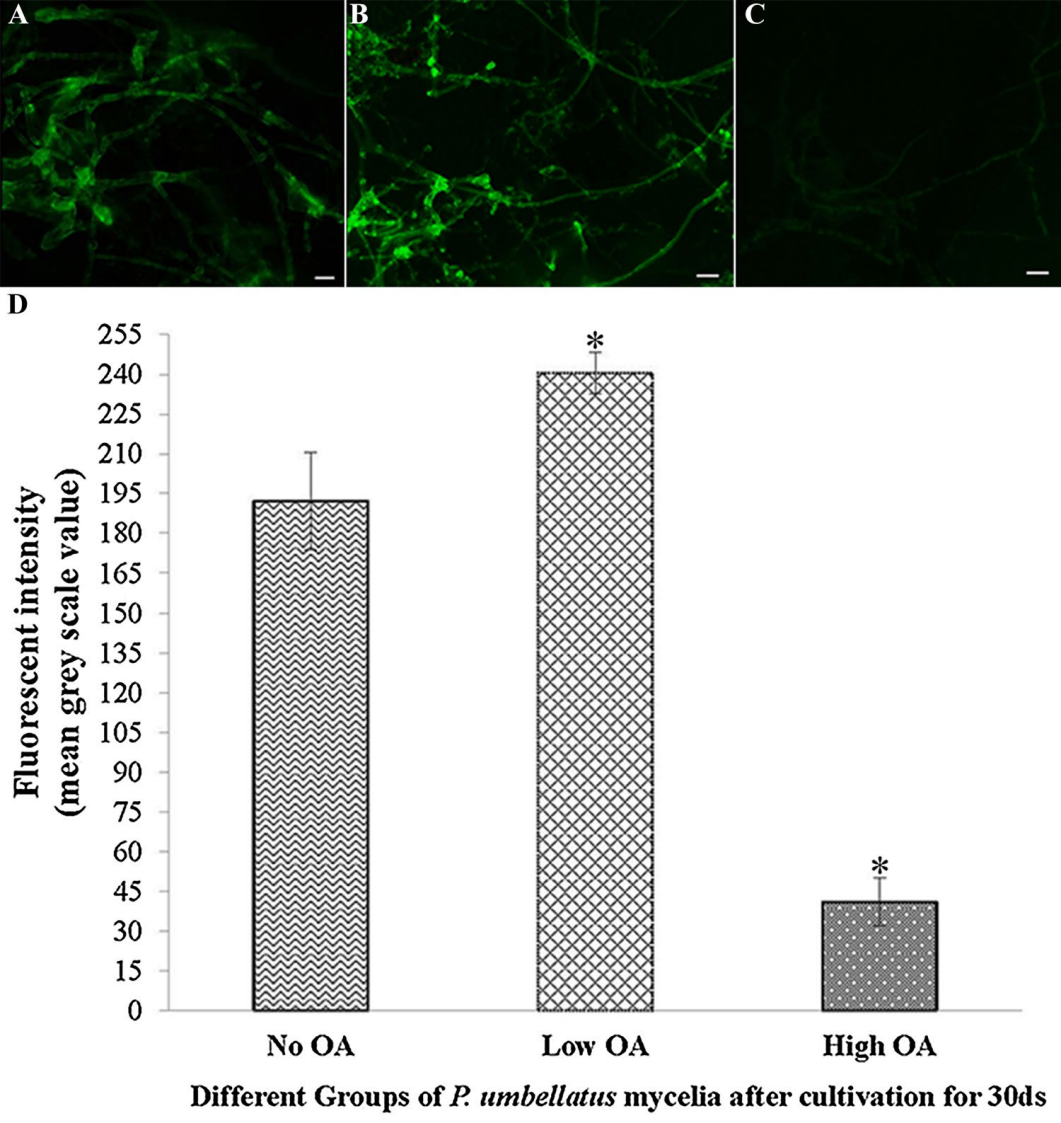
ROS content and fluorescence intensity of ROS in *P. umbellatus* mycelia affected by different concentrations of oxalic acid. After cultivation for 30 d, the ROS content of *P. umbellatus* mycelia in the No OA (**A**), Low OA (**B**) and High OA groups (**C**) is shown. Images are representative of three independent experiments (n = 30). Scale bar, 10 μm. The mean grey scale value of ROS in different groups of No OA, Low OA and High OA from three independent experiments is shown, with *standing for *P* < 0.05, compared to the control (No OA) group (**D**).

In the No OA group, the ROS content of *P. umbellatus* mycelia had accumulated to a certain extent after cultivation for 30 days as shown in Fig. 2A, while in the Low OA group, the ROS level was greater than that of the control group (Fig. 2B); however, in the High OA group, the ROS content was considerably lower than that of the control group (Fig. 2C). Compared with the control group, the fluorescence intensity of ROS increased in the Low OA group but decreased significantly in the High OA group (*P* < 0.05, Fig. 2D).

### Validation of the DEGs using qRT-PCR

**Figure 3:**
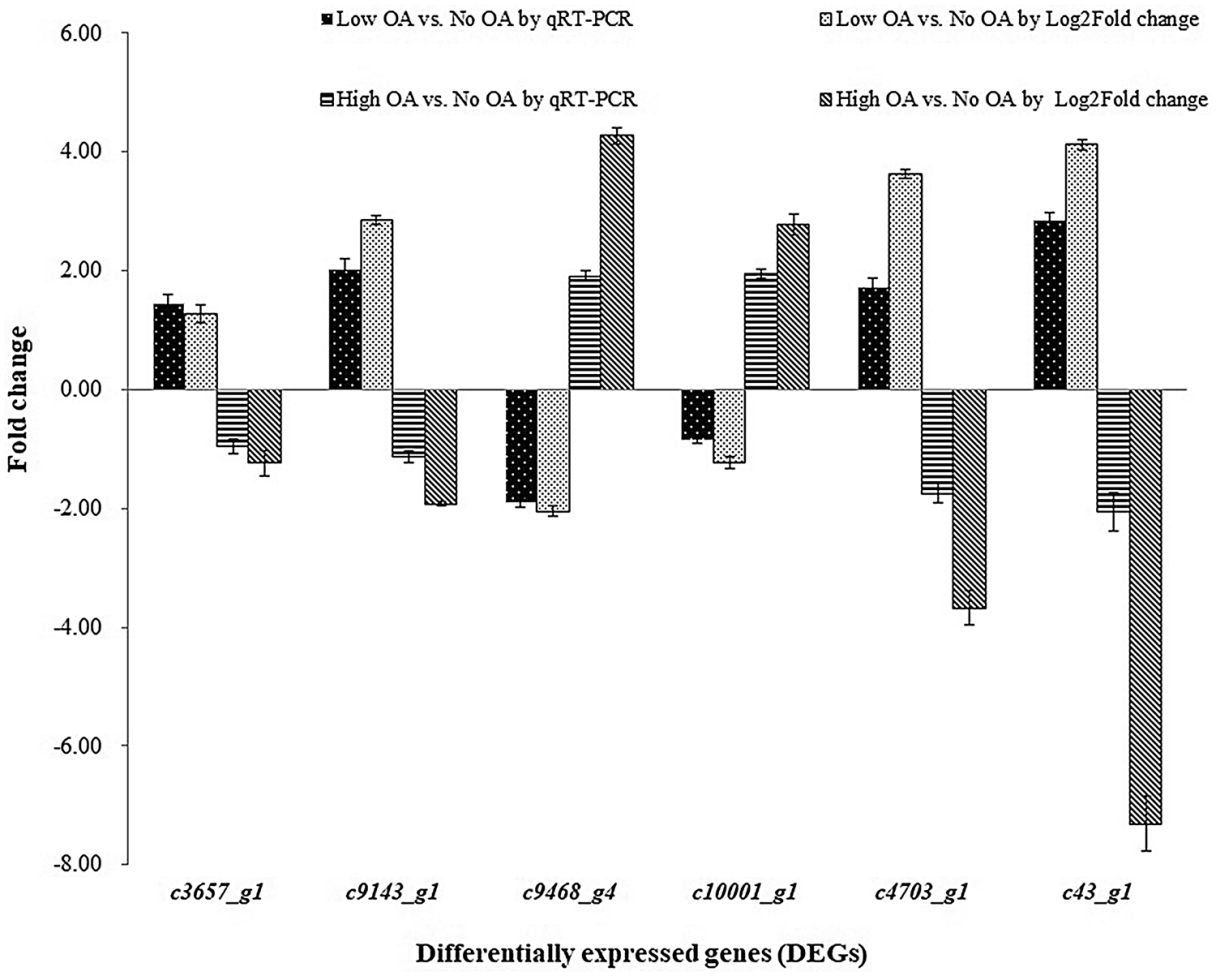
Validation of the DEGs using qRT-PCR. Fold changes of the expression of the selected DEGs including *c3657_g1*, *c9143_g1*, *c9468_g4*, *c10001_g1*, *c4703_g1* and *c43_g1* between Low OA or High OA and No OA by qRT-PCR and RNA-seq were represented.

Six unigenes were selected and examined at the transcriptional level by qRT-PCR, to validate the reliability of the RNA-Seq data (Fig. 3). All the selected unigenes showed that the trend of the expression patterns were consistent with that of the transcriptomic data, which indicated the validity of the RNA-seq results.

### The net Ca^2+^ and H_2_O_2_ fluxes during *P. umbellatus* growth were affected by different concentrations of oxalic acid using NMT

**Figure 4:**
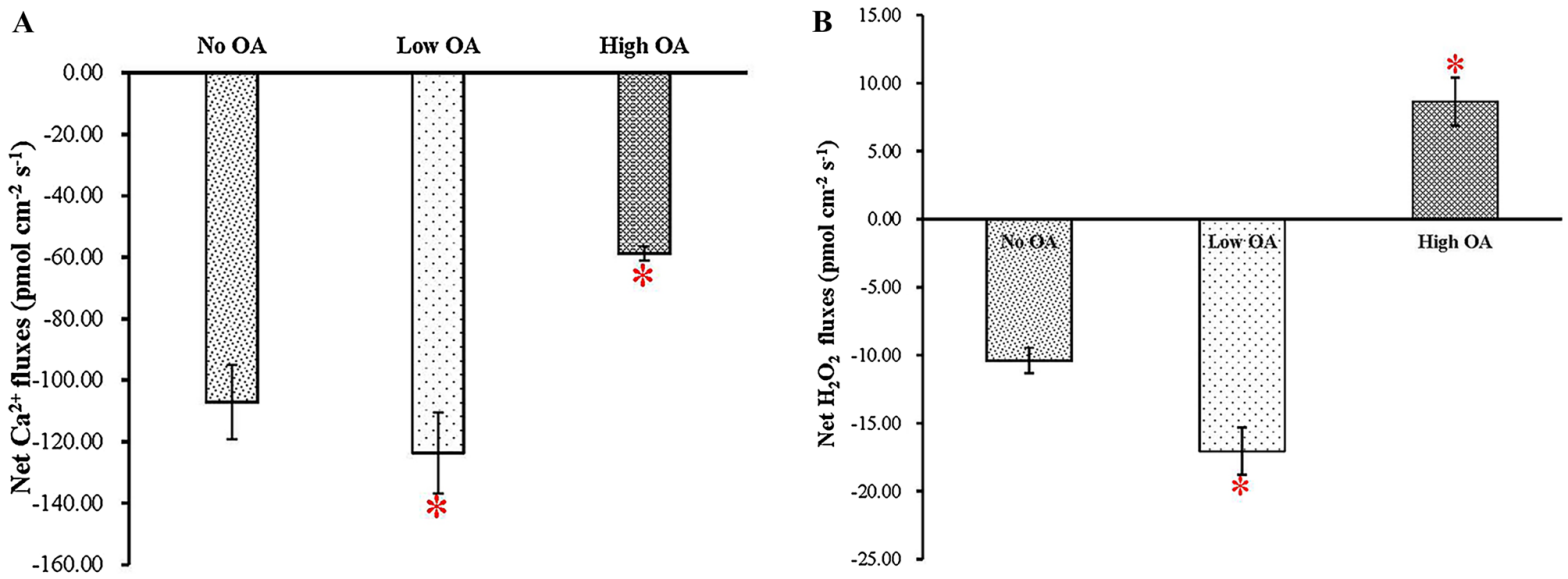
Real-time Ca^2+^ and H_2_O_2_ flux measurements of *P. umbellatus* mycelia affected by different concentrations of oxalic acid using NMT. (**A**) Real-time Ca^2+^ influxes detection. (**B**) Real-time H_2_O_2_ fluxes measurement. The values of the net Ca^2+^ and H_2_O_2_ fluxes are the means ± SD (n = 30), with * representing for *P* < 0.05.

The negative values represented the influx of Ca^2+^ and H_2_O_2_ (Fig. 4A,B) and the positive values represented the efflux of H_2_O_2_ (Fig. 4B). After cultivation for 30 d, Ca^2+^ influx was measured in the mycelia of *P. umbellatus* over a period of time in different groups, and the net Ca^2+^ uptake was observed to increase in the Low OA group but decreased in the High OA group significantly (*P* < 0.05) in comparison to that of the No OA group (Fig. 4A). The H_2_O_2_ in the High OA group presented slight efflux, with the net fluxes ranging from 4.05 to 9.77 pmol cm^−2^ s^−1^ (Fig. 4B); however, H_2_O_2_ presented influx in the No OA group and the Low OA group (Fig. 4B), and there were significant differences between the Low OA and High OA groups, compared to that of the control group (*P* < 0.05). The significant positive correlationship with Pearson’s correlation coefficient (r = 0.991, *P* < 0.01) between the flux measurement of Ca^2+^ and H_2_O_2_ was determined by correlationship analysis using SPSS, and the scatter plot is presented in Supplementary Fig. 1F.

## Discussion

It was found that oxalic acid accumulation led to lowering ambient pH and promoted *Sclerotinia sclerotiorum* sclerotial development^13^. Recently, it has also been reported that *S. sclerotiorum* sclerotial formation was inhibited by exogenous oxalic acid at 25 mM and higher^14^. In this study, oxalic acid also had a dual effect on the sclerotia formation of *P. umbellatus*: a low concentration of OA (ranging from 0.05 to 0.10 mg/mL) promoted sclerotia differentiation from mycelia, while a high concentration of OA (1.10 mg/mL and higher) inhibited the formation of sclerotia (Fig. 1, Table 1).

OA plays diverse roles in different organisms such as fungi, plants or animals^15^. For example, in postharvest exogenous application of oxalic acid on lotus root slices, 5 mmol L^−1^ concentration was comparatively less effective to inhibit increase in Browning index (BI) and browning degree (BD), compared to 10 mmol L^−1^ oxalic acid application^16^. Oxalate can be a potential source of ROS^17^. It was well documented in *Nicotiana benthamiana* that during *S. sclerotiorum* infection, oxalic acid initially suppressed ROS-mediated host plant defence response, but later increased ROS production followed by programmed cell death. Therefore, OA had dual opposing roles in *Sclerotinia* pathogenesis^6^. In addition, oxalate was observed to lead to ROS production in renal epithelial cells^18^. Therefore, oxalic acid is able to increase ROS production.

ROS are unstable and highly-reactive chemical species^19^. NADPH oxidase activation is a key enzymatic source of ROS formation^20^, and in eukaryotes, it is an important source of ROS including H_2_O_2_^21^. Other oxidases, such as alcohol oxidase and homooctameric flavoprotein, play important roles in maintaining oxidation–reduction balance in fungi and are capable of catalyzing the oxidation of methanol to formaldehyde and hydrogen peroxide. Alcohol oxidase is involved in the methane degradation pathway, which is part of the energy metabolism of certain yeasts^22^. Galactose oxidase can catalyse D-galactose and O_2_ to form D-galacto-hexodialdose and H_2_O_2_. In the present study, the DEGs encoding oxidases and dehydrogenases were highly expressed in the Low OA group, while they were downregulated in the High OA group, which led to high oxidative stress in the mycelia of *P. umbellatus* in the Low OA group and compelled sclerotial formation.

The functions of reductases and other ROS-scavenging enzymes are also essential. Glutathione S-transferases (GSTs) have been reported to catalyse reactions involving the conjugation of glutathione to electrophilic compounds and to remove ROS^23–25^. Based on the transcriptomic data, the expression of reductases, glutathione S-transferase, manganese peroxidase and catalase was upregulated to various extents in the High OA group while the expression of reductases decreased in the Low OA group, compared to the control group. This result indicated that the reducibility of the *P. umbellatus* mycelia in the High OA group was higher, but the reducibility of the *P. umbellatus* mycelia in the Low OA group was lower, which helped to maintain the oxidative stress level in the latter group.

Unsaturated fatty acids are the intrinsic reason for lipid oxidation^26^. *c9511_g1* and *c9756_g1*, encoding fatty acid synthase were downregulated in the High OA group compared to that of the control group, which suggests that lipid oxidation is lower in the High OA group. This possibility has been verified in a previous study, which indicated that high concentrations of exogenous oxalic acid (0.5–1.0 mg/mL) reduced lipid peroxidation in a concentration-dependent manner^5^.

Sclerotia, as an aggregated and dense mass of mycelia, are formed from normal mycelia under conditions such as coldness, nutritional depletion, growth factors^8,27^. It has been reported that sclerotia develop when glucose in the medium is depleted^28^, thus certain growth conditions leading to oxidative stress triggers sclerotial metamorphosis^8,29^. In our previous study, different carbon sources present different roles during *P. umbellatus* sclerotial differentation. Among them, maltose, fructose and glucose induce sclerotial formation, with maltose was the best carbon source for *P. umbellatus* sclerotial transformation, while sucrose and soluble starch only promote *P. umbellatus* mycelial growth but they are unable to stimulate sclerotial development^4^. It has been reported that intracellular ROS production was examined by a thiobarbituric acid reactive substances (TBARS), which is used as an indicator of oxidative stress^30^. Furthermore, previously, the lipid peroxidation was evaluated by TBARS and the TBARS content in the mycelia of *P. umbellatus* cultivated in maltose medium with sclerotial formation was higher than that of the mycelia in sucrose medium without sclerotial differetiation during cultivation^5^. Therefore, the level of oxidative stress in *P. umbellatus* mycelia of the control (No OA) group cultivated in the maltose medium was high and large amount of ROS generated during sclerotial formation. Since *P. umbellatus* grows slowly and its cultivation period is longer than those of the other fungi such as *Penicillium* sp. or *S. minor* to produce sclerotia. Long-time cultivation will easily lead to nutritional depletion, and under this condition sclerotia will form.

In this study, with the CM-H_2_DCFDA probe for visual detection, the ROS content and the fluorescence intensity in the *P. umbellatus* mycelia of the Low OA group were greater than those of the control group, but the fluorescence intensity produced by the ROS level in the High OA group was less than that of the control group (*P* < 0.05, Fig. 2D). In addition, using the NMT technique, in the High OA group, H_2_O_2_ exhibited slight efflux, while in the control and the Low OA group, H_2_O_2_ influxes were demonstrated in this study (Fig. 4B), and it is conceivable that the intracellular H_2_O_2_ decreased in the mycelia of *P. umbellatus* in the High OA group but increased in both of the other groups. This result was consistent with the fluorescence intensity generated by the ROS content in *P. umbellatus* mycelia affected by different concentrations of oxalic acid (Fig. 2). Thus, these results further confirmed that the high oxidative stress was formed due to much ROS production in the control group and the Low OA group and facilitated *P. umbellatus* sclerotial formation, while lower ROS content was observed in the High OA group.

NADPH oxidases require different redox cofactors and are regulated either through regulatory subunits or by calcium ions, and calcium-dependent regulation has been verified via EF-hand motif experiments^31^. NADPH oxidases belong to the respiratory burst oxidase homologue (RBOH) family. When the activity of Ca^2+^ and phosphorylation-dependent RBOHD was upregulated, ROS production increased during plant defence^20,32^. In the present study, real-time measurement of Ca^2+^ flux showed that Ca^2+^ influxes in the Low OA group were the highest, followed by those of the control group, and Ca^2+^ influxes in the High OA group were the lowest among these three groups. Greater Ca^2+^ uptake in the control group and the Low OA group is associated with higher concentrations of intracellular Ca^2+^; thus, more Ca^2+^ interacts with calmodulin, which serves as the dynamic Ca^2+^ protein sensor in eukaryotes ubiquitously and transmits calcium signals downstream^33^. Therefore, intracellular H_2_O_2_ and Ca^2+^ increased in the Low OA group but decreased in the High OA group compared to the control group. More H_2_O_2_ efflux existed in the High OA group, and less H_2_O_2_ was left in *P. umbellatus* mycelial cells. In contrast, as H_2_O_2_ presented influx in the Low OA and No OA groups, increasingly abundant H_2_O_2_ was stored inside *P. umbellatus* mycelial cells, and a high level of oxidative stress was observed. In other words, in the control and Low OA groups, mycelia differentiated into sclerotia, and elevated ROS levels were accompanied by increased levels of calcium ions. Meanwhile, transcriptomic analysis showed that the DEGs related to, for instance, calmodulin, calmodulin-binding motif, and calcium ion transport were upregulated in the control group and the Low OA group, which was different from the High OA group. This finding implied that DEGs related to calcium signal transduction and ROS cooperatively played important roles in *P. umbellatus* sclerotial formation.

The optimal pH is required for OA and OA induces ROS and PCD later^6^. It is speculated that low concentration of oxalic acid may manipulate the redox enviornment to provide optimal conditions for *P. umbellatus* sclerotial formation or regulate oxidases and reductaces to increase ROS production. Once sclerotia formed, oxidative stress will maintain at a relative high level during sclerotia development^5^. However, high concentration of oxalic acid, like sucrose or soluable starch, although they increased *P. umbellatus* mycelial biomass, they cannot induce scleotial development. Under this condition, high concentration of OA serves as an antioxidant. The mechanism of oxalic acid affecting *P. umbellatus* sclerotial formation deserves further studies.

In conclusion, a low concentration of oxalic acid induces ROS production by H_2_O_2_ influx; conversely, a high concentration of oxalic acid inhibits *P. umbellatus* sclerotial differentiation and lessens oxidative stress by H_2_O_2_ efflux. A significant positive correlation was shown between the flux measurements of calcium ions and H_2_O_2_. In addition, the DEGs encoding a series of oxidases were upregulated and reductases were downregulated in the Low OA group, the opposite phenomenon was shown in the High OA group (Supplementary Table 3). Therefore, in comparison with the control group, a higher level of oxidative stress formed in the Low OA group, which led to more sclerotial formation in *P. umbellatus*. The results of transcriptomic analysis were in accordance with those of the content and the fluorescence intensity of ROS, and H_2_O_2_ flux detected by NMT. Further investigation is required to determine the mechanism by which oxalic acid affects oxidation–reduction during *P. umbellatus* sclerotial formation.

## Methods

### *Polyporus umbellatus* sclerotia culture and exogenous oxalic acid treatments

Fungus identified as *P. umbellatus* used in our experiments was isolated from the wild sclerotia collected from Guxian of Shanxi province in China and stored in wheat bran agar slant medium at 4 °C^34^. *P. umbellatus* was inoculated on the maltose medium containing 0.05 mg L^−1^ vitamin B1 in 9-cm-diameter Petri dishes for sclerotia culture, in which maltose served as one of the most optimal carbon sources for sclerotial formation^4^. The components of the maltose medium were referred to a previous report^5^.

Different concentrations of exogenous sterile oxalic acid solution were added to the sterilized growth medium to culture sclerotia and mycelia for transcriptomic sequencing and qRT-PCR validation. At first, oxalic acid solution was filtered with a 0.22-μm Millipore filter (Merck, Germany). And then, sterile oxalic acid solution were added to sterilized maltose medium with final concentrations 0 mg mL^−1^, 0.005 mg mL^−1^, 0.01 mg mL^−1^, 0.05 mg mL^−1^, 0.10 mg mL^−1^ and 1.10 mg mL^−1^. The group without oxalic acid (0 mg mL^−1^) was treated as the control (No OA) group. The colony morphology of *P. umbellatus* was observed and the fresh weight of the sclerotia were collected and measured after inoculated for 30 days. The experiment was repeated three times, with 30 replicates being employed in each group.

The mycelia cultivated for 30 days on solid medium in the optimal concentration of exogenous oxalic acid (Low OA group) promoted sclerotial formation of *P. umbellatus* and the minimum exogenous oxalic acid that thoroughly inhibited sclerotial development with no sclerotia formation (High OA group) and the control (No OA) group without oxalic acid were subjected to transcriptomic sequencing. After cultivation for 30 d, *P. umbellatus* sclerotia in the Low OA group and No OA groups both entered SD stage or stationary phase and the mycelia in the High OA group grew exuberantly and stably and entered stationary stage, thus, about 100 mg of each sample of mycelia without sclerotia from the three different groups was respectively collected using sterilized surgical blades and tweezers in the vertical flow clean bench, and immediately frozen in liquid nitrogen for total RNA extraction.

### Transcriptome sequencing

Transcriptome sequencing and analysis were performed by Beijing Novogene Co. Ltd (Headquarters). To obtain the transcriptome of *P. umbellatus* mycelia subjected to different concentrations of exogenous oxalic acid, total RNA was isolated from the frozen tissues of the mycelial samples without sclerotia of the three groups in *P. umbellatus* (Low OA, No OA and High OA), with three biological replicates in each group. RNA extraction, concentration detection, RNA integrity and purity, library construction, mRNA purification, first strand cDNA, second strand cDNA synthesis, PCR reaction and PCR product purification all followed a previous study^34^. Clustering of the index-coded samples was performed on a cBot Cluster Generation System (Illumina). Then the library preparations were sequenced using the Illumina HiSeq 2500 platform, and paired-end reads measuring 125 bp were produced^34^.

### Sequence annotation

The raw data in fastq format were processed to obtain clean data. By removing adapters, poly-N and reads with low quality, clean data with high quality were generated. The DEG analysis in this study was based on clean data with high quality. The assembly of all the reads and gene function annotation were all performed based on a previous study^34^.

### Differential expression analysis of transcripts

The nine samples of the total RNA extracted from *P. umbellatus* mycelial tissue were used for three independent sequencing libraries. To better obtain the molecular information of *P. umbellatus* sclerotial formation affected by different concentrations of oxalic acid, it is important to identify the DEGs among the three groups. Then, to verify the functional annotations of the genes, the clean tags were mapped to the all-unigene information with the short read alignment program^29^. The data from the three biological replicates of each group were merged, and the transcript abundance of each gene was determined by the reads per kilobase of exon model per million mapped read (RPKM) values.

To identify genes responding to different concentrations of OA and sclerotial formation related DEGs, |log2 fold change| of gene transcript abundance among the sequencing libraries for the mycelial tissue of the No OA, Low OA and High OA groups was calculated. The analysis of the differential expression in the three groups was determined with the DESeq R package (1.10.1)^36^. The statistical significance of the differential expression level for each gene was conducted by assessing the probability. The false discovery rate (FDR) was also used to determine the threshold of the adjusted *P* value. A gene with an absolute value of |log2 fold change|> 1 and an adjusted *P* value < 0.05 was regarded as a DEG.

### Validation of DEG expression using real-Time quantitative PCR (qRT-PCR)

All primers (Supplementary Table 5) were designed using Primer3 input (v. 0.4.0) from https://bioinfo.ut.ee/primer3-0.4.0/^37,38^, assessed by AmplifX 1.5.0 and synthesized by Genewiz Company (China). The 18S rRNA gene (EU442272) of *P. umbellatus* was used as a reference control^12^. The primers detected were PCR-amplified in the cDNA of the samples of the No OA, Low OA and High OA groups. All the 6 selected genes related to oxidase, reductase and energy metabolism from the RNA-seq data exhibited differential expression in *P. umbellatus* in response to oxalic acid. The qRT-PCR protocol and the reaction procedure were all performed according to a previous study^39^. The gene expression ratio was evaluated by the comparative 2^−ΔΔCt^ method and the fold changes were calculated by logarithm of 2. Combined with the expression patterns of qRT-PCR and the DEGs from the RNA-seq data, the column chart was made (Fig. 3).

### ROS detection and fluorescence intensity of ROS in *P. umbellatus* mycelial cells

ROS content was measured following a GMS10010.7 kit protocol. *P. umbellatus* mycelia of the No OA, Low OA and high OA groups were picked off using scotch tape. The detailed procedure was conducted according to a previous study^40^. The fluorescence intensity of ROS was calculated and quantified as the mean grey scale value from 30 samples in each group with Axiol Vision Rel 4.6.

### Real-time Ca^2+^ and H_2_O_2_ flux measurements of *P. umbellatus* mycelia affected by different concentrations of oxalic acid using NMT

Ca^2+^ and H_2_O_2_ fluxes were measured on the surface of *P. umbellatus* mycelia in different groups after cultivation for 30 days as mentioned above using an NMT System (NMT100 Series, Younger USA LLC, Amherst, MA, USA). Ca^2+^-sensitive and ROS-sensitive microsensors were provided by NMT Service Center, Xuyue Beijing Sci. & Tech Co. Ltd. The experimental voltage is + 600 mV. The primary position of the Ca^2+^ or ROS-sensitive microsensor was placed 30 μm from the *P. umbellatus* mycelial surface, and the more distant position was 60 μm.

### Statistical analysis

The data were analysed with double-factor Pearson correlation analysis and all statistical analyses were performed using SPSS 17.0 (SPSS, Chicago, IL, USA). The data were analysed with one-way ANOVA and using the Student–Newman–Keuls (SNK) method, significant differences were determined. Data are presented as the means ± SD from at least three independent experiments. *P* values < 0.05 were considered to indicate a significant difference.

## Supplementary Information

Supplementary Information 1.

Supplementary Information 2.
