# Supplementary figures and images for: Use of transcriptomic profiling to identify candidate genes involved in Polyporus umbellatus sclerotial formation affected by oxalic acid
Source: Sci Rep. 2021 Aug 30;11:17326. doi: 10.1038/s41598-021-96740-7 (PMC8405643; doi:10.1038/s41598-021-96740-7)

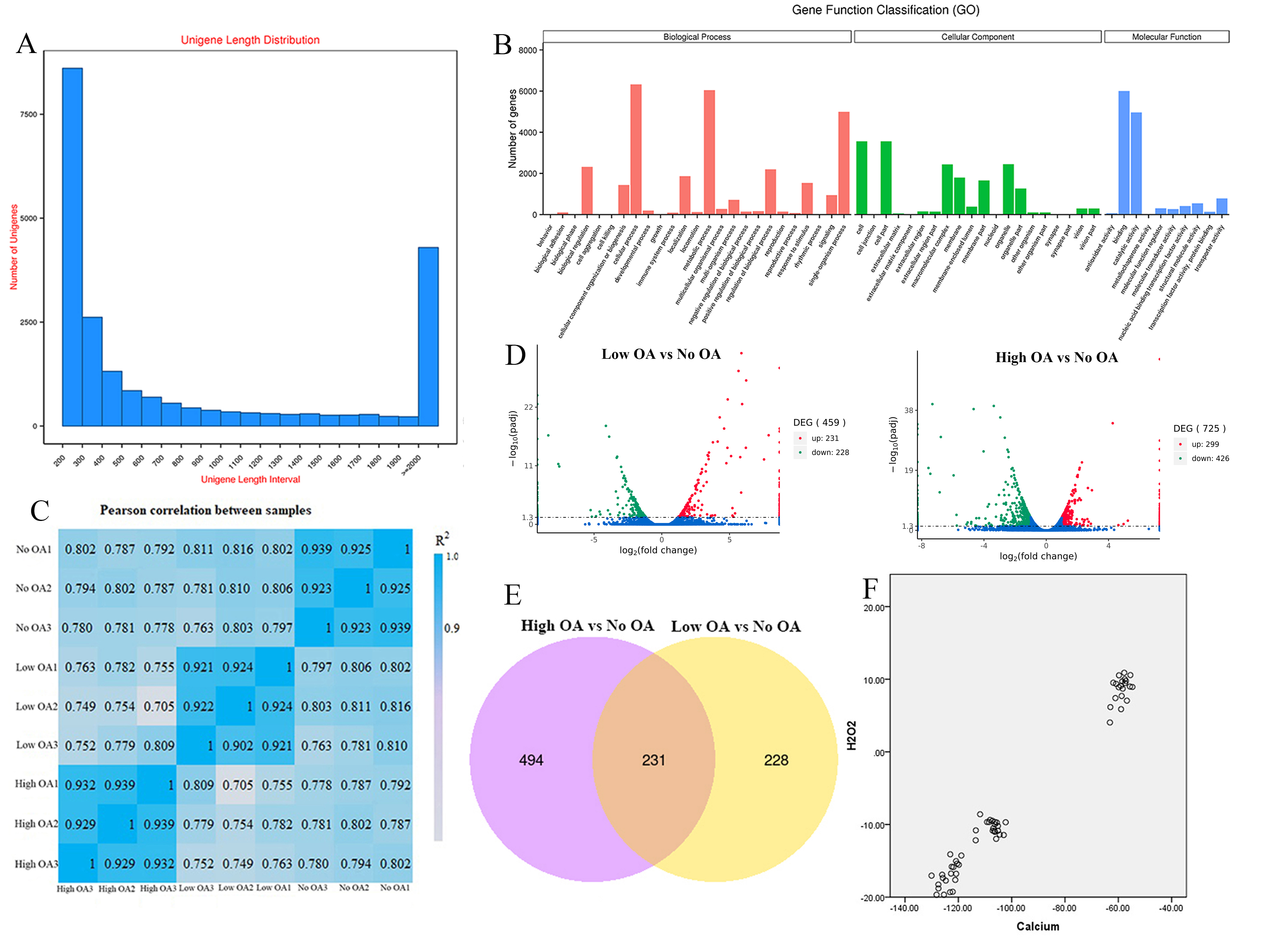

Supplement: Supplementary file 1 — Supplementary Information 1. [file 41598_2021_96740_MOESM1_ESM.jpg]
