# Supplementary material for: Use of transcriptomic profiling to identify candidate genes involved in Polyporus umbellatus sclerotial formation affected by oxalic acid
Source: Sci Rep. 2021 Aug 30;11:17326. doi: 10.1038/s41598-021-96740-7 (PMC8405643; doi:10.1038/s41598-021-96740-7)
Supplement: Supplementary file 2 — Supplementary Information 2. [file 41598_2021_96740_MOESM2_ESM.pdf]

Use of Transcriptomic Profiling to Identify Candidate Genes Involved in *Polyporus umbellatus* Sclerotial Formation Affected by Oxalic Acid

Yong-Mei Xing[#1], Bing Li[#1], Xu Zeng[1], Li-Si Zhou[1], Tae-Soo Lee[2], Min-Woong Lee[3], Xiao-Mei Chen*[1], Shun-Xing Guo[1]*

[1] Key Laboratory of Bioactive Substances and Resource Utilization of Chinese Herbal Medicine, Ministry of Education, Institute of Medicinal Plant Development, Chinese Academy of Medical Sciences & Peking Union Medical College, No. 151, Malianwa North Road, Haidian District, Beijing, P. R. China 100193

[2] Division of Life Sciences, University of Incheon, Incheon 22012, Korea

[3] Department of Life Science, Dongguk University, Seoul 04620, Korea

*Correspondence:

Xiao-Mei Chen, E-mail: cxm_implad@163.com;

Shun-Xing Guo, E-mail: sxguo1986@163.com.

[#]Yong-Mei Xing and Bing Li contributed equally to this work.

Supplementary Table 1. Summary of quality evaluation of sequencing output data

Sample	Raw reads	Clean reads	Clean bases	Error (%)	Q20 (%)	Q30 (%)	GC (%)
No OA1[a]	48862698	47515246	7.13G	0.02	96.46	91.35	55.30
No OA2[a]	55825490	54510292	8.18G	0.02	96.61	91.62	55.54
No OA3[a]	45034602	43841868	6.58G	0.02	96.06	90.70	55.78
Low OA1[b]	44804502	43382478	6.51G	0.02	96.47	91.51	55.95
Low OA2[b]	46263970	44731548	6.71G	0.02	96.13	90.85	55.45
Low OA3[b]	47793444	46365222	6.95G	0.02	96.46	91.44	55.51
High OA1[c]	43868088	42749076	6.41G	0.02	96.65	91.81	55.14
High OA2[c]	44566228	43376858	6.51G	0.02	96.58	91.66	55.13
High OA3[c]	47705028	46319972	6.95G	0.02	96.51	91.50	56.66
Total	424814050	412792560	61.93G				

[a]No OA1, No OA2 and No OA3 represented the mycelia of *P. umbellatus* in the control group.

[b] Low OA1, Low OA2 and Low OA3 stood for the mycelia of *P. umbellatus* in the Low OA group.

[c] High OA1, High OA2 and High OA3: the mycelia of *P. umbellatus* in the High OA group.

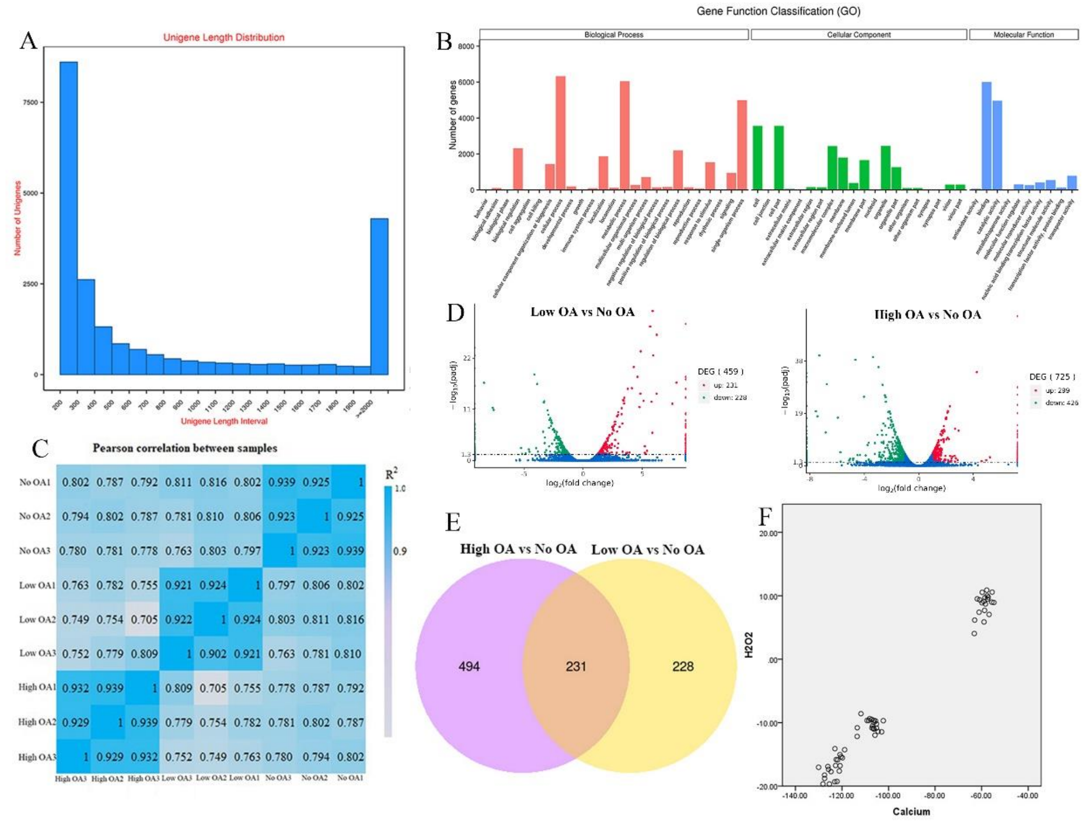

Supplementary Fig.1A. The length distribution of the assembled unigenes.

Supplementary Fig. 1B. GO categorization of unigenes.

Supplementary Fig. 1C. Correlation of RPKM distribution between both biological replicates of each group. Comparisons of estimated RPKM distributions between the three biological replicates for the No OA, the Low OA and the High OA groups. Pearson's correlation coefficients (R^2) between the replicates are presented.

Supplementary Fig. 1D. Volcano plot of the DEGs between both of the samples of each group. The red dots and the green dots represented the up regulated and the down regulated expressed genes respectively with adjust p value < 0.05 and |log2 fold change|>1 among the Low OA and No OA, the High OA and No OA groups.

Supplementary Fig. 1E. Venn diagram of Low OA vs No OA and High OA vs No OA.

Supplementary Fig. 1F. Correlation of real-time fluxes of calcium ion and H_2O_2.

Pearson's correlation coefficient (r=0.991, *P*<0.01) between both of the groups are presented, indicating a great significant positive correlation between the real-time fluxes measurement of calcium ion and H_2O_2.

Supplementary Table 2. Gene function annotation analysis in 7 public databases

	**Number of Unigenes**	**Percentage (%)**
Annotated in NR	10914	48.45
Annotated in NT	12864	57.11
Annotated in KO	4063	18.03
Annotated in SwissProt	11236	49.88
Annotated in PFAM	10571	46.93
Annotated in GO	10645	47.26
Annotated in KOG	6934	30.78
Annotated in all Databases	2469	10.96
Annotated in at least one Database	18855	83.71
Total Unigenes	22523	100

Supplementary Table 3. DEGs between Low OA *vs*. No OA and High OA *vs*. No OA groups.

Annotation or function of enzymes	Compared groups	adjusted *p*-value	Log_2Fold change	Up or down regulated	Gene ID
Aldo/keto reductase	Low OA *vs*. No OA	0.043349	-1.28	down	*c8217_g1*
	High OA *vs*. No OA	1.56E-05	1.10	up	*c9378_g1*
	Low OA *vs*. No OA	0.00084016	-1.71	down	*c9881_g2*
Nitrite reductase	Low OA *vs*. No OA	0.00067558	-1.23	down	*c10001_g1*
	High OA *vs*. No OA	0.0222	2.78	up	
Glutathione S-transferase	Low OA vs. No OA	0.010097	-2.05	down	*c9468_g4*
	High OA *vs*. No OA	1.29E-34	4.27	up	
	Low OA *vs*. No OA	0.011893	-2.79	down	*c6055_g1*
Manganese peroxidase	High OA *vs*. No OA	1.13E-07	1.30	up	*c6600_gl*
	Low OA *vs*. No OA	0.00025727	-2.62	down	*c6769_g1*
Cytochrome c oxidase subunit 1	Low OA vs. No OA	3.2751E-19	4.13	up	*c43_g1*
	High OA *vs*. No OA	1.1009E-40	-7.31	down	
	Low OA *vs*. No OA	1.048E-18	lnf	up	*c1151_g1*
Cytochrome c oxidase subunit 2	High OA *vs*. No OA	1.8551E-20	-7.56	down	*c159_g1*
	Low OA *vs*. No OA	3.66E-08	lnf	up	*c18027_g1*

Cytochrome c oxidase subunit 3	High OA *vs.* No OA	1.25E-18	-7.44	down	*c13857_g1*
	Low OA *vs.* No OA	2.12E-9	lnf	up	*c10530_g1*
Alcohol oxidase	Low OA *vs.* No OA	3.22E-10	2.87	up	*c8987_g1*
	High OA *vs.* No OA	1.84E-05	-1.14	down	*c8176_g4*
	Low OA *vs.* No OA	0.0096364	1.47	up	*c8020_g1*
	High OA *vs.* No OA	0.00078	-2.80	down	*c5369_g1*
	Low OA *vs.* No OA	0.0018874	2.86	up	*c9143_g1*
	High OA *vs.* No OA	0.04752	-1.93	down	
NADPH oxidase	Low OA *vs.* No OA	0.037599	1.28	up	*c3657_g1*
	High OA *vs.* No OA	3.19E-07	-1.24	down	
	High OA *vs.* No OA	0.000177	-2.02	down	*c8851_g1*
NADH dehydrogenase subunit 1	High OA *vs.* No OA	9.20E-13	-6.82	down	*c17518_g1*
	Low OA *vs.* No OA	0.000277	lnf	up	*c1946_g1*
NADH dehydrogenase subunit 4	Low OA *vs.* No OA	0.000199	lnf	up	*c1893_g1*
	High OA *vs.* No OA	2.3558E-22	-lnf	down	*c524_g1*
	Low OA *vs.* No OA	4.78E-12	7.64	up	
L-rhamnose 1-dehydrogenase	Low OA *vs.* No OA	0.00060777	3.63	up	*c4703_g1*
	High OA *vs.* No OA	5.43E-09	-3.68	down	

Calmodulin	Low OA *vs.* No OA	0.00809	3.49	up	*c2043_g1*
	High OA *vs.* No OA	0.0001615	-Inf	down	
	Low OA *vs.* No OA	0.023908	1.413	up	*c8739_g2*
	High OA vs. No OA	0.000316	2.09	down	
	High OA *vs.* No OA	0.0003234	Inf	down	*c3257_g1*
Calmodulin-binding motif	Low OA *vs.* No OA	0.00019948	Inf	up	*c4558_g1*
	High OA *vs.* No OA	0.00031588	-1.09	down	*c8133_g1*
Calcium ion transporter	Low OA *vs.* No OA	0.0090109	1.44	up	*c9830_g2*
	High OA *vs.* No OA	0.00010713	1.23	down	*c8205_g1*
Cellular calcium ion homeostasis	Low OA *vs.* No OA	8.14E-07	1.87	up	*c3191_g1*
	High OA *vs.* No OA	3.0275E-26	-2.49	down	
Glycerol 2-dehydrogenase	Low OA *vs.* No OA	0.000029484	1.69	up	*c5285_g1*
	High OA *vs.* No OA	0.001855	-1.04	down	
Glutathione peroxidase	High OA *vs.* No OA	0.00023161	Inf	up	*c4316_g1*
Cytochrome c reductase	High OA *vs.* No OA	9.12E-08	Inf	up	*c16064_g1*
Ribonucleotide reductase alpha subunit	High OA *vs.* No OA	2.28E-06	1.17	up	*c4563_g1*
Catalase	High OA *vs.* No OA	6.24E-05	1.20	up	*c6329_g1*
Quinone reductase	High OA *vs.* No OA	2.27E-05	1.52	up	*c8932_g1*
NADH dehydrogenase	High OA *vs.* No OA	2.84E-30	-6.77	down	*c1494_g1*

Rhamnogalacturonase	High OA *vs.* No OA	0.033252	-1.75	down	*c4388_g1*
Rhamnogalacturonan acetylesterase	High OA *vs.* No OA	7.50E-12	-2.69	down	*c4271_g1*
Fatty acid synthase	High OA *vs.* No OA	3.92E-13	-1.69	down	*c9511_g1*
	High OA *vs.* No OA	3.90E-09	-1.40	down	*c9756_g1*
Fatty acid desaturase	High OA *vs.* No OA	0.0019857	-1.76	down	*c9487_g1*
Galactose oxidase	High OA *vs.* No OA	2.27E-05	-1.11	down	*c7390_g2*
L-gulonolactone/D-arabinono-1,4-lactone oxidase	High OA *vs.* No OA	0.01134	-1.12	down	*c7166_g1*
NADH dehydrogenase subunit 2	High OA *vs.* No OA	1.02E-08	-Inf	down	*c1758_g1*
NADH dehydrogenase (ubiquiNone) Fe-S protein 6	High OA *vs.* No OA	4.16E-05	-1.04	down	*c6419_g1*
NADH dehydrogenase subunit 3	High OA *vs.* No OA	0.00027	-Inf	down	*c2305_g1*
D-isomer specific 2-hydroxyacid dehydrogenase	High OA *vs.* No OA	2.60E-13	-1.79	down	*c3247_g1*
D-xylose 1-dehydrogenase	High OA *vs.* No OA	7.37E-08	-1.47	down	*c7606_g1*
Sorbitol dehydrogenase	High OA *vs.* No OA	9.22E-07	-1.22	down	*c7742_g1*
Succinate dehydrogenase	High OA *vs.* No OA	8.14E-06	-Inf	down	*c14038_g1*
D-arabinitol dehydrogenase ($NADP^+$)	High OA *vs.* No OA	2.28E-05	-1.06	down	*c7145_g1*
Acetaldehyde dehydrogenase	High OA *vs.* No OA	6.90E-05	-Inf	down	*c10255_g1*
Alanine dehydrogenase	High OA *vs.* No OA	0.000394	-Inf	down	*c12085_g1*
	High OA *vs.* No OA	5.37E-05	-Inf	down	*c19597_g1*
Glyceraldehyde 3-phosphate dehydrogenase	High OA *vs.* No OA	0.003745	-Inf	down	*c17591_g1*
NAD-dependent glutamate dehydrogenase	Low OA *vs.* No OA	3.32E-06	2.26	up	*c8341_g1*
Glutamate dehydrogenase	Low OA *vs.* No OA	8.92E-05	1.93	up	*c8341_g2*
NAD-aldehyde dehydrogenase	Low OA *vs.* No OA	0.000187	1.87	up	*c8523_g1*

Isocitrate lyase	Low OA *vs.* No OA	1.54E-13	5.12	up	*c7209_g1*
Fatty acid hydroxylase	Low OA *vs.* No OA	3.53E-24	4.87	up	*c6742_g2*
L-threo-3-deoxy-hexylosonate aldolase	Low OA *vs.* No OA	0.00054459	-1.83	down	*c7929_g2*

Supplementary Table 4. unigene sequence of the DEGs

>c8217_g1

GTGAACCAGATTGAGGCGCACCCACTCAACCTACAGGATGACCTCCTTCAGTTCTGCA
AGGAACACAACATACACATCACGGCATACAGCCCGTTCGGGAACAACATGGCGGGCG
CCCCAATGCTCACGGAATACCCTGAGGTCATTGAGATTGCTAACAGATTGAACGCGAC
GCCGGCGCAGGTGCTCATCGCGAGGGCCGTGAAGCGCGGGTACTCGGTCATCCCGAA
GAGTGTAAACCCAGCGCGGATCGAGTCGAATTTCCAACAGGTAGCGCTCTCGGACGAG
GATTACGAGAAACTCAACGACCTCGGCCGGAAGAACCCTAAGCGGTACAATGTCAAG
ACGACATACCCGGTGTTCTGGGATGTGAACATCTTCAACGATGAGCTCGACAAGACGA
CAACGAATCAGATCAAGATTGCGTGAGTTGAATATTCGAAGGTGGAGAGGGCTAACGC
CACGATGCTATTGTGCTTGGGGAGCAAACCTGACAGTCTGAAACTTCGAAAATGTACT
TATATGTATCCATATATCTTGCTTCTCGGAGCCGTCCATGTCCATTCTATGTTCATTTCCGT
CGCGCACGGCTGCGGATCCTTCACAATACATAGGCCCCATATCCTTGTCTCCCCGTTGA
CTGTTGACGACCTATCAATTCTAGCCGTCGGGAGGACCTCCTTGCGCGACTGTAACTGA
CCATGTTGGCATGTCTCAGCAGAATGGAAGCGGACCATCACACTACCTGACATCCCTG
CGCCCCGACCGCTGTGGCGCGAAAAGTCCCAACTGCCTGCTTTTCTTCAGCAGGATGA
ACACTTTCTGCCCTTCTACAGGAAGATATGCGGTCATTCGCTTGGATCCCATCGCCATG
GTCAGGCATCTGGAGGATCCAGAGGCTTTCGCGGCTGCCGCTGCCATGCGACCTAAGT
CTCATCTGGTCTACATCAGCCGCGATCTAGGCTTGCCGTTTCCCGAGGATCCATGGTATT
CGTTCTCTGTGATGCCAGTTGCGCCATGTCTCCGCGTGCTCGATGAAGAGCAGGGGGT
TACGGAAGACGTGTGCACGCCTATCTTTCCGAACGAATCCCATGCGACCGGGCGGCGA
GCGCTCCGTCCCGATACACCCTTTCCGTACTCGGAATGCTACCAGTGGGCCATGAGCGA
GTTGGAGGTGCGCGTGTGCGCGCGACAGGAGGGATTCAATGATAGTGAAGCGGTCAA
ACTCCCGGTCGTGGAGCGGATGGAGCTGATTTACAATCTGGAACGGGACCAAGCGCA
GTTGGAGGATCGCTACACGACCGGCTGTATAAGCACGGGTCATACGGACGGTCTACGA
TCCCGCATGACCTCTGAAAGTGTGGAGTTTTATCCTCACCGAGGCACGTACAGTTCTGA
CGAATCCGCGTGCGACACGAGCAGCTCGGAGATGGAGTCGAGCTGCAATTTCCGGAA
ATTCACGAGGGACATTGAGACTGTCCCTCTTGTTCACCTTTGGCTCAATCTCGAAGAGC
ATGTCAGACAAAACGACATCCCCAGTCCCGCTCAGCTCGTAGAAGAGTGCGACAAAAT
AGCGAGGTATGTCTTGCACATTTCGCGTTTCTTCCAGTCCATGTTCATGTCACCTTCAGC
ATTATTCGACAGGCGAGGGCGCGAAACCCGTCGCTCTCGTCCTTCGACACCATCCTTTG
TGCAGACACTTGCGACTCTGAGGATGACAACGACTGGATATCAGAGGCAGACTCCATC
CCTGACCTAACTTTGGCTTTGACGATCGAGGGGTGTCCGAACGCGATAGTTCGGTAGC
CTTGCCAGTGGTAGATCGTGTGAGCCGAAACAATGTTGCGTCACCCACCGTGTTATATT
GGACCTGAGAGCCAACGCTTCTGCAACATTGACTCACAGGTCGTCGTCCTGCTCGAGT
TTCTCGTGGCCTCCACAGAGCGATTACTACGTCGTTCATCTGCATGGCATGATCAAGAC
CAGACGGGTGATAGGTTGGCATTGGATAAAGGCAGAAGATCGAGATTTAGAATCGTGT
GTCTAAAGCGCGCCTGCTCATCCAAATTGTCCATCGTCAATTGCAATGTCGAATCCCTT
CCTTAGACTTTACTAACTTCAAGTTTCTTCCCCCAAACCTGACTCTGCTCTGACGCTCT
TTCGCACACGAGAGCTCCGTTCCTCTTGTTTCAACACTCCATTTATACATGCGCATACCC
ACCCGTTTCCTTTGATGTGTGCTTGTCGCGTGCATTCCTGTGCGCATCCTGCTGTGAGAT
GATGACATCTCTGCCAGTGAGATGCTCTGAATTCTCAGAGTGTCACAGTGCTAGGGTG
GTTTTCACATTGTGTAACACACAATTTCAATTGTATAATGCAGCAACAGCTGTTGTGCAT

GTATGGAACATATGAGACAGTTCTGCATGTTGAACAAATGGCATGTGTAGACTTCTTAT
CCTTCTTGTTCTTTTTCCTCTCCTTCCTTTCACGATCCCGA

>c9378_g1

GTCTCCGATGATCCGCGGGAAGGAAGATATGGGGACTGCTATGCCGCTTCGCGTCTCCC
TGGCGCAGTAAACGGTAATAGTAACCATGACATCATTTGGTCGGCTGTTTTCTCCCGTC
CGTTCTACGGAATACATGCTGAGACTATGTGTAGAGCGTTCGACTGTGTCCGAGTCGAT
AGATAGATATAACCAGCGACTGCAACAATGTAATGTAACTCGTGAATAAATGTGGTAAC
CCGCTTGATCCCACGCCCGCCGGCACTCTTGTGAAGACGTGGTAAAGTTTGTCGCACG
AGGGTTCACTGTGAGAGAAATATTCCCAAATTACCTTTGCGTGTGCGGGGGTTCACAAT
AGCGGCCCGACAGCTTGAACTCTATTTTTCTCTTCACAGGTTACGATCACGCCGCGTCG
ACGGGGTTCCAGGTAATTGCTCCGCCCTTCCCCCGGTCGAGTGCATTAATGGCAGCCAT
GTCTTCCGTACTGATCTCGAAGTCGAACAAATTTGCGTTCGACACGATGCGCTCGGGCT
TCTCCGACTTGGGAAGAGGGACGAAGCCGCGCTGCAAGGACCATCGAACGAGCGTCT
GCGCCGGATCCTTGTTGTACTTCTTGCCAATGTCGACGATCGTGTCGTTCCATCTTCCCT
TAATCAGAGGGGTATACGCTTGCACAACGATGCCGTTCTCCGCGCAGTACTCGACAATA
TCTTTCTGCTGGCATAGCGGGTGCAACTCGAGTTGGTTCACACTCGGAGTCTCAAGAC
CTGCGATTCTGATCTCCTCGAGGTGTTTGACGCCATAGTTGGAGACGCCAATAGAACGA
ATTTTGCCTTGGTTTCTGGCCTCGATCAACGCCTTCCAAGCCTGCAGACGAAGATCCTT
GCCCGAAAGCGGGCTATGGAGGAGCATCAAGTCCAGATATTCAAACTGGAACTTGTCA
AGCGAATCATCCACAGCAGCGAGGGTCTGGTTGTACCCGTGCTCCGGGCTATTCACCTT
GGAAGTGACGAAGATTTCCCCACGCGGGATGCCACTGGCTCTAACGGCCTTTCCAACC
TGAACCTCATTCCGGTATACGCGAGCAGAGTCAATATGTCTGTACCCATGCTTCAACGC
TGCGAGACATGCGAGTTCACACTTGTCGATGTTCTGAAACACCCCAAGTCCAAGCAAA
GGCATAGCATATCCTTGTGTTGTCATCAGCATACCTGCGCAGCCCTTCTCTCGCGCGAG
GGGACGACAAACCAGAGGAAAGCTTGACGATGGAGGCGATAGTAAAAACGGCGGCC
ATTTCGGTTAGCGCAATTGCGTCCCGGTCTTTGTGGTCTGTGGATACAGGATGGCAGAA
TCAGCATGCCCGTGTATATATAGCATGTTCAAATTCCACTGCCTGAGAAAACACCCCAT
GCTCTTGGGAAACGTTGTAGTTCCGTTTGCTTTCATCATCGTGTCGATGGCCTCCGGTC
GGGTTGATGACAAAAGTGGCAACATAGGCACTTAGCAATCGCTTAGCAATCAGGCGCC
GCGAAGAGAGTCTATGTGATAGAGGATCACAATGAGGGACGGAAAAGACAATACCTCT
ATATATGCAAACTTTCAGACCATGGACACCTCCCCCTTGTCACTCCTCGGTGTTAAAGA
TGTCGCTTCATGCTCCCTCTTCTCTTTCAATACGTCCTCGACGATCGCTCCCCCATCGGC
CGCGCTCTCCCGGTCCGACTCCAGCTTCTCTTCGTACTGCGGCACGGGGGCCTTACGA
AAGCCAAAGCCGCCCTGCCACCCGATCCATGTGTACCGTGGACAATGCGGTCCGCGGA
GAAGCAGCACAAACAGCGTCCACCCGATCCACGCAAGGTTCAGCGCAGCGTTGGCGC
GCCAGCCGTGTTCGATGAAGACCTTTGACCCGACGGACGTACCCATCACGGTGCCAAG
GAAGAAGGCAATCAAGATGATGGAATTGAGACGCGCCCGTGCGTCCGCGTCGAGCGA
AAAGACGGCCGTGGTGAGCGAGACCTGCTGGGACTGTCGGAACACGTCGAGGCCGAT
ACACACAATGATGACAACCACGACGTGCAGACCAACTGCGGCGGTCTGGATAATAAAC
GTGATGAGGAGCGCGAGAGTTCCGACGAGTGTTGCAGACCAGGGTACGAGGTTATCG
ATCATGCGCCCGATGAGCGGAGCCATCGCAACACCGGCCATGCCGACCAGGCCAAATA
GACCAATGACAAGCGTAGAGTAGTTGTAAGGCGGTTCAGATAGGAGGAATGTCAGTGT
GACCCAGAAGTTTGAAAACACCGCGCTGGCCGCGGCGGTAATAAGCACCGCCTGGAC
GAGCAGCGGCTCCGTCACAGTAAATTTCCCCATACTATATAGTATACCCCAGTATGTGGT

CCCGGTACCCACGTTCTTCGCGGGATAGTCGGGGAGCTTCCAATAGAAAACCGCAAGG
ATGACATACTGCATCCCGATAGCGATGTAGTACACAACACGCCAGGAGACGAACTGTG
CCACGACTCCCGAGAGTACCCGCGCAATGAGCACCCCAAACAGAAAACCGGAGAGGA
CTATGGACATGGCAGTTGCGCGGCGGTTGGGCGGGGCGAGGTCGGCCGCGAGTGGCA
TAAGAATTTGCGGGACGACATTGCAGACTGCCAGGAGAAAACAGAGAACCTCGAAGG
TGAGGAGGTTAGATGTAATCGCTAAACCAATTGTGAGGGAAGCCGTGATGAAGGTAAG
CAGAAGTATCAACGGCCTTCTCCGTACAAGGTCGCCCAGGGGTGCAACTAGGAGTAGC
CCGGTAGCATAGCTGTTGTGTGAAATTCATAGTTCTATCAGGGCCATGCCTCATCATATC
AAGGTTGAAGAGGCATTTACCCTGCTTGGAGGAGTGTCGGAATGTTGGACACCTCGTG
GTCTGTTACTCCGAACGACTCGGAAAATTGTACTGTGCTCGTCATATCTATTAGTCTCCG
ACCGACCGACCGACCGACTTTCCCAACGATGACCCACTCAAAAGAGGCTGGCAATAG
TAAAGGTTTGCAACGATAAATGTGCCCGTGATTCCGAACATAGCATTCATCAACAGCCC
AAAATGTGGGGGTCTTTGAGGATCATGCTGCACGTTTCTCGGGATGGGGAGAAAACCA
AAATCTTTGGTCGGCCGCTCGGGAACGCCGTCTTTTGGTTCAGCGAAAGGAACGACAG
TCTCAGCGATGGACATCGTGCCCGACGTTTGGCGTCCGTTTGACAAGTCCTCGACAGA
CATGGCAGATGGAAATGGGAGGTGGGAGTGTCTGAGCCGGAGATCCTGCCATGATGAG
TGTTTAAAATACTTGCAAGCATGGGAATGACTCAGAGTGCGCGCCGACTCGTCCGCTT
GAATAGTAATACAACTCGCGGCCAAATGCGTGTATGTAAGGTCGCCTGAATCGACATTA
GCAATCTCCGTGTACTCATGTTGACCTTCTGGATTTCAGTGGCACATGTTTGCACTTCG
GGAACTCGACGCATTGCTATTTAGTCAAGGCAATACGCACTTTAGCCCCGGCG

>c9881_g2

GAATGGGGGAGTATATGAAGGGCCGCGTCAGTTTCCAGAGCAGGGGACGCGCTCTCGC
GGGCGGCTAATGAGCGGAGTTGGGGGTAAAGCCGAGATTTCTGCTTCGACGTCCGGGA
TTGGCAGGCGGGCTCCGGGCGTTTGGACCAAGTGAAAGGCACGTCTTCTCCCGACATT
ATCCCGCTCCCTCCCAGGTTTGGAATGCCCGCTCTTCCATCGGCCGGCCATGGCCCTCG
TATCGGAACATAAATAAATAGCTCACCCGCTAATAGTACTACAATATCTCCAAACACCCC
TTCCGGCCTCTTCCAACTCAGCGGCCGTTGGGATCTCCGGTCGTCTCCGGCTACCCTAA
CCCCACCCTCCACCGTAATTTCGGCCGTTACATTCCGGCCCGACTGTCAAGGCGCGCAC
ATTCTGAGATTGCGGTTACCAGAATCACACCGAGCTCTTCGCATGGGAGACCCTCGTC
GCCTGCGCAAGCTACTCTCTTTCAACACGCCCGTTCAGACAAGGATGAAAAGGCGACT
CGTTGTCCGCTGTCGTACAGCGTGCGAGGCGAGTTGCGCACCAAGGTCCTCACCTGCA
TGAAGGCTTGCTGTGCACCTTCGTCTTTGTGCAACAGCTGCGTGAATACCACGACAAG
CAAGCTTTTTGACTTCTGCGATCCTCACAACACGCAGCAGCCATCGACAGTGTCTCCA
AGCCCCGCCATTCCCGGCCGACGTATGGCTAGTTCGCCGGCCGGGAACGCACACAAAG
TCGGGTTTTCTTCTTCTCCCATGCCCCAGATAATCTTCATTTGGGCACTTTCTTCTGCTT
CCACTTCATCGATGCATCCTACGTTGCACCCCTCGGTGGATCTGCGCCAACGGTCTCAA
CGGCAGTCTTGCAGCGTTGTGGTGCATCCCTGTCTCTTTCGAGCATTCCCCGTCATCGT
GATCGATTTCGATTCTCACATCGAGGATAGACAGTCGCATTGTCTAATCAGGTAAGATA
GATATACGCCGATTGCTTGCGTCCGTATGGTTTGGTGCGAAGCACGTATGTGAAGTGCC
TTCGTACCTGGTCAATCAGTCACCGTCCCCCACTCATCTCCGCCACCCAATCTTTCTGC
GCAGCACTCAGGCTTATCACACGAAGAGAGGCACATTCGTCTCAGGATTCAGCACGCA
TAACGGCCGCCTGAGAACCAGATCATCTTTGCCTACGCGTAGCTATTCCTAGCACCGGC
AATTGGGACGAACAGTGTGGCCGCCGACGCGCGGCGCGAATAGACTTGACAGGGCTA
CGCGTTCTTGTTCCCTCATCTCGGCGTTTCGGCACTTTCATCAGCGCGGCGAGGGTCAC

TCGCCAGGGACGTGGAAGCTAGTAGAACGCAAGAGCGAGAGGACGAGGACGAACCG
GCTGCGTGCGGTGCTCCGTCGTCCTCGAGCGTGGGTGAGCCCGCAGCCATGCGACCGT
CGTTCGCGAATCCCGCACGCGCCTCCGGCTCCTTGCCGCGCAGCGTAACGTATGGTAG
CCTGAAGGCTTGGCTTTCTCGCTCCCTCTTCGCTCTCGGACGCTCTCGGTGTTTTCCAC
GCTTGCGCGTGGGAGGACATGCGAAGCGTCGACCTTGTCCTGGCTTTGCACAACGTCA
CTTTCCCCATTCGGCCACCGCACATCCCCCTGCCTCCCCAATCAATTCAAATTCCCCGTC
CAGAATGATACGGAGTGGGTGCCAGTTACGACGCCGATGCGTCTGGACCGTCGACATC
GACAGGCGCGCTGGATGCGTCTCGAGCCGAGTGGACGCTACTTGCAACGAACCAGCC
TTGAGCTTGAGCCTCTCCACCCATCGGCGATCTCGTTTCCGTTTCTTTCTCTGCTTCTGA
CGGTACCGTTCTGTTTTCTCTCTCTATTTCACTCGAAATCGGCGGCGGCCAATAACGGG
GTGCTCGTGCTCGAGATAGCGATAGCTCCCAAGACGCCGTCGCCTTCTTCTCATCCAGG
TTTACGGTTCCCTGTGCCTGGTCTCTGCTACTCGTGGGACGTGCGTGCTTGCAAGCCTT
CCCTTTGGGCGGGCGTCTCGTTCTTGTGCTCCCAGCACCATTCCTCGCGCGCAACACA
GGGAAATGCGACGTTGATATCCAGTACTACAGGGTGCATCGGCCCGTACCGTCTTCAAC
TAGTCCTCATTGAATGTCGTGAACATGACCCCTACTGTCACTGTACAACGTCGTGCGGC
GCACGCTTCCCCATTCGCCTCGGGTGCTCCTGCAGCAACGTTTCGTTCACGGACTTACT
ATTCAGGCGCCGGCTGTGTATGCTTCCGAGTCTATATGGACCAACGCATCATGCATATAC
CGCCGGCGTCTGCACCTTCCCTCTGAACGGCGAAGGGAGAACGAGCCTAGAGGGACT
AACTTACAAGCCCCCCAGGGGCTCGTTCCCCAAGTCACAGCGCGGCACGCGTGCAGA
TGAGATTGGGGGTGGCTTCTCCAAGTCTTCCGTCTTCCGTCTCTTCCGACTGGTCGTGG
TCTGCGGTTCGCGGGCTAGCCTTGCTATTCACTCGCTACTCGACCTCGACCCCGCATCT
GCGCATCTGCGCCCTCCCTCGGCGATCAACCCAGGGTACAGTTGTTGCCTCCACGCTA
GTATGCATATGTAGTACATAGTCAGCACACACTGTAGCCTGCTCGCCTGTCGAAGCCCG
GCCCTCCGGAAAGGTGGGTTCGGGTTCGAGAATTCGGGGCCCCGGCCTCGCTGCGTTC
TTGGAGTCTGGACCTTTGCAGGGGCATACTAATACCATACTATCGCGTCTGACATCCTC
ACCCCTTCTTTTGCTCGCGCTCGCATCTGTCGACGGGTCGGTCTTCTTTGCATTTACCAT
CTACTGCGGTACTAGTACGTTCATGCGACGTCGTCATATACGGCGCTAGGCTCGACACC
ACCCTCGGGCACAGACCTCTCTGTATTGTGCACAGGGACTGAAGAGGCGCTCCCATTC
TGTCGTTCCAAGCTCCAAGCTCCAAGCCTTGCGTCCGGCAGGCCTGTGGGACAGCGA
GGAAGAAAAGACACTCACGGCGGAAACCTCGGACCAATTGACCCCGCCACCGCCCGC
CAGGTTCATCCAGTTTCTCCGTGTGTTCTCTGCCTCGCCTCTGTTGCGGGAGTTTGTAG
TGCCTGCCGGCGGGGTTCTCTGGCACGTCGTGGTGTGTGTGAGTCTCACTCACTCACT
CGCACTACTAGCACGCCTTCGCGTCTGGGATCCTCAGAGTGCTTCTGCCGATGCGCATA
GGAGTCTCTCCCCAGTCGTTAGCTCGAGGTCGCCTTGGGAAAATGAATGTGGTGCTGA
AATGCCAGATACATAGATTGTCCACTCACACTTTCGACTTCATGTGGAGTCCCGGCAGC
CATACGCTACAGTCGAGCCCCTGCAAGTTTTGGACACTCCACTGACACCGTGGCGCAC
GCTCCAGCGTAGGTGTTCGTGCAACCCACCTCCACGAGGTCCTGGGGCCACTGCGGCT
CACATACCGGTTATCGGTGGGACTCCGATAGCTGCGATATCTGAATGGTATACTGATCGT
GATTGTCTTTTTTCCTCCCTTCGAATCGCCATTGTGTTGATGAGCGGCGGGCCGATGATT
ATGACTTTTGTGTGCACTACGTACGCAATCGTCCTGACCGTCTGGCGTTGTTTCTCCGA
GCGCGGATGCTGACTGACGGCGCGCGGCGAACTTGTGTATGGGCACCACGATACGATG
CGTCGGACTTGCACGATTGCAGCCATGTCGTTACGAGTAAATAATCCACCCGCGAACA
GCAATAAAGACACTAGTTTTGTTGAGGTTGAGATTCGGACAGGCGCCTTGGGGCGCTC
CGAAGACTTTCCGTTCTCGCGGAAGAAAAACATCAACATCCCAACAGCTTGCGCGGTC

GTACGACGCCTCTGCTCAGGGCAATGCATCACACGGCGACTATCCGGACATGAAAGCC
ACGATGAGCAATAGACCTTCCTTGCGGTGCTGCGCAGAAACGAAGCAAATTTGGCATC
GTCAAGGTCATCGGGGAACTCGCAACAAACAGTATCTTTCTTCCAGGTTCACCACTCA
TACAGGAGAACTCGTATCTAGGGCGCCGCCGACATACCCGGGACCCTCTTTCTCGTACA
CCAGAGTTTAAACGAATCGCTCGAGCTCGCTGTCCGGCAATAATCCCTGTTCATTCGTG
CGCTCGAACACGCTCAGCGCGCCGCCTTGGCATACGAGGCTGAGCCGTCCCCGCTGTC
CGTGCTCCTTCACGAGGTCCTGGATTGCGCGGAGCGCGTCGACGAGCTGGCGGAACA
CCCGCTGCGTGGACGCCCTGTTCTCGACGTTCCGGAGTTCGGGCGTGCCGAGCTCGTG
CTTCTTGATGCCTTGGAACGTACCGCGGTCGTGCACCGCGAGGAAGAGGTGCGGGGTC
GCGGAGAGGAGGAGCTGGGGGTACGCCTCTGCCCAGTCGAACTCGTCGACGTACTTG
ACCGAGCGCGTGGTGAGCTCGATGATCGAAGATTGGGGGACTTCGGAGCCCCCGTGG
ATGACGGCGATGTCTGCGCTACTGGAGACTGGGGGGGTGGTTGAGCTTTGGGACGCG
ACGCTGAGGCCGGCGAGCATGTCTGTTAGGCTGTCAACATTTGCGGGGGTACTTTTGG
GACTGCTAGGCTGGGAGGCGGTCCGGGTCGTACTCCGAGACGAGGCTGGTTTAGGGAT
GCATGCATCCACCTCGAAGCGGACAATGAGATGTGCCGCTCACATATTGCACAATACGG
TGGTATCCGGTGCTCCGCTCACATCCCTTCGCAGGCATTGTACTCTCCCGCTCAAAGTT
GATACCGCAGCCCGACCTGGGGGGCACGGGGACCTCCCTCGTACGCTTCTCCCAGCGA
TGCATCAGTACGGTCTTTGACCCACCAAGCTGGAGGTCGATGCGGAACTCTTTCAGCG
GCTCGTCGTCGATGCTGCTGGAGTGCTGCAGCCACCGCATGAGCTTGCGGAGGCCGTT
GCGGTCCGTGACGATGTCGACTGCGCTCCAGTCGATAGAAGTGTCCGCGTTGTCCTCG
GAGAAGACGCCAACGGCGCGGATGAGCGGAAGAAGGCAGGATGCGGTGCCCATTCGG
AAGCTGTTCTGGTCGACGAACCGGACATCGGTGTCGAACCGCACGCGGTACGGGAAG
GGGCGCTCCCGCCATTCGGGAGGAGAACCAGGAACGATGATGGTAGGCTGTGATTCCT
CGACCCAGTTGTAGGACCCGATGTACTTGACATCCTCTGGCTTGATATGGTTACCAGTA
GATGGGCTGATTTGAGGTTTGGAAAGAGTCTTTATGGCTGTGTTTTGCAGTCCCTCCTG
AATATCGCGGTCAGGCGGGAGTGCAGGTGTGGCGGTCGGACGACTGGTGGGGTGATTT
CCATAAGTATTGCCGCGAGAAGCAGAGCGGGGTCTGTACGAAGAATAGGAAGACATCT
TAAGAGGTTAGTGAGAGGTGACGAGAAGTTGCGGCAAGGGAGAGGGACTTTATATAC
AAGTTCCAGTGCTGCAGGAAGGCTCACGCTCTGACGCTGGCCTTGACACTACCGCAG
CATAAGAACTTGAAGTCTGGTCCTCAGGATATGCCATAATAACGCAGGTAATTCTAGAC
TGATTGACCATTTCTGAGTTGCGCAGTTTCATACTGCAACTCACGCAGCCTACCGTAGT
CACTTGCTGCGAGTGGGGAGGACGCTGACATGCCATTGCAGGGCTTCGGCATGATCAC
TCCCAAGCTCACTCCTTCTGCAGGTCCTCTTCTGCAGGTCCTCTACGAGCGGTCTGCAT
ACTGCAGCAGCGTTGACGTCTGAGAAGCTTCATGACCGAGATAATGATCACGACATCG
GTAAGTTCTCTCGCTGTAGGGTGCGACATGACCGGAAAAGGAGGGGGGTCACGGATTT
CGACAGAAGTTGAGGTTGAGGCTGCAATACCAATACCGAACCACATCCCCAGATGTGT
CATTGTGGCTGCAGGTCGTGCAGCAGCATGCTCGGCTGACGAACACACATATACAGCG
GACTATTCCAAAAGCGAAAGAGGCTTGCGTCGACTCTCTGCGGTCGATTGGCACGAGA
CAGACACATGCGTGATGCGCAAGGTTCGAATCACAAACCATGAATGCACGTGGCGTAC
AGGGCGGACGAAAGATACCAAGCTTAGTCCTTAAGGATGAGACGACTGCGCCTGTGA
ATCAGTACCGGTTCGATGCCTGCATGGTCACAAAAAAGCAAGACGAGCGCGTCAAGA
GCCTTCTCCCTGAATCCTGAACTGCTGCCAGGCGCGTTGAAGACGCTGGGACTATTAG
CTATATTGGCGTACACAAGGGAGAACATGACGATGAGGGAAAGAGGATGGCCGCCATT
AGTATGCTGCTGTGTCGCCGTGTTGAATCAAACAGTAGAGATTAGTACTACGTTGACAT

AGTCAACGTTCAAACTACCACATGATTCCACAGTTATGCATTGCGGCTCAACGGCCGCC
ATCCAAGCCACCAGTACCATACTACTGGCACTTGGATGGGGTTTCCTTGGGTAGCACTG
GATGATTATTTGAACGAGTAAACCAAGAGGAAAACCTGCGCTCGTGCCAGACCGAGTC
GAATCTTGCCACATTATTCGCGGTCTCTCTACAATTTATAAAACGGACATTGGATGTCTT
ACTGAATAATAGTGCGTTGAGAACAAGTGTTGAGAAGTATTGGAAACAAATAAGTGAA
GTTGCTTATCCGAATTGAAGTGGATTACATCATAGCCATCAATGCTTACTGAACCTTTGT
GTGAAAGCCTTACCGTGATAATAAATCCATCATGTCCCCCTACCACCCCTTGCCGCAGC
GTGAGGTGGACATCGAGCAGCTCAAGCAGCCTGACCTCCAGCACGAAGACGTAGAAA
CCCATCGCAAGCAGACGTTCATCTGGAAGCGCCTTCTACTCGTTGTCATAGCCATGGCT
GCAGGATGGCAGCTCGCCTGCCTTGTAACCAAGCACATCTACTACAGCGCAACTTCTC
ATCTGGCGCATCGTTGCCGACCCACTTCACGCTCCCTTCTGGCGATAAGATTCCTGCCG
TCGCACTCGGTACGTCTCACTCGGTACGTCTCACGGCGTAGGCGAGGTACGATGCTGA
GTGTGAGATTGCGTTAGGTGTGTGGCAGGCTGCTAAGAATGAGGTTGGACAGGCCGTC
AAGGTCGCCTTGAAGACTGGCTATAGGCACATCGACGGCGCCTGGGCGTATCAGGTAT
GTGTCTTCAATCATGTGCACCTTGGTTGCGGCCTGGCTGCACAGCACAGAACGAGGAA
GAGGTCGGCGAAGCTTTGCGGGAAAGCGGCGTTCCTCGCGCACAGGTTTGGCTAACG
TCGAAGGTATGCCGTGCTCAACACAAGTGTTCACGCTTTCACCATGCATTCCCACGTGC
TATAAATACAGCTTGCTGGTCCCTTCCACGCACCTGAGGACGTAGAAGCTGCGCTTCAC
GATTCTCTGCAAAAATTGGGCGTAGACTATCTTGACTTGTATCTGGTGCACTGGCCCAT
CGCCATCGAGGAGGGTACAGACGAAGTTGACGAGGCTCTCACTGCCAACCCCTATCCG
ACATGGCAGAAGCTCGAGGAAATAGTTGACAAGGGGAAAGTTCGCAACATCGGTGTT
AGCAACTTCAACGTGCCAAGGCTTACGCAACTTGACAGCCAACCCTCTAAAGTACAAA
CCTGCTGTCAATCAGGTTGAGCTCAGCTATTGGAATCCTCAGCCTGAGCTACTCAAGGC
GCGCAGTTTTGTAGTAGCGAATTTACCACAAATCTGGGGAGTATGGTTTGCTCCTTGAG
GCGTACTCCCCGCTTGGTAGTGGCACACTTGTGAAGGACACTCTCGACGTACCTGAAG
TCAAGCCAATCCCACATGTGCTGGGTATAACGCCTGCTCAAGTCATCCTCTCATGGAGT
GTCCAACGCGGCACGGTCGTTTTACCTAGGAGCGTGACACCATCGCGCATCGAAGAGA
ACCTGCAGGTATTCAAGTTCAAGCTGCCAGAGCCGCTTTTCGACAAGTTGGAGAAGGC
TGCTGCGGACCCACCCAAACGCGTCGTCAATCCATCGAAGCGTTACAATCTTGGGTAC
GATATCTTCGATGACGACGGGGGCGTGATAATGCCCGATGAATGAATGTAGGGCCAGG
AAACGACGGCAGTAAAGGATGTATTGCATATCGTACGAATGAATCAAGGTGCTTTACAA
TAAGCGAATACTACATGTACACCACTGTATCATAAGTAACGAGTAAAACGCCAGACATG
AGGATTTCGTGACGTTACTAAACTACAAGTACGCACCGCAGATAGAAGTACACCATTA
GAAACAACCTGACTGCGAGTGCCAATACCGATATGCCATGTACAGCTTGCGATGGCGC
GGGGTGAATGAACGAGATCCGAGTGGAAGTGAGGAGTGAATAACGAGTGGAGGTGGG
GAGTTAATAACGAGTGCAGAATGTGACCCTAGATGACCGTATGACCAGATGAGAGGGT
ATCGAGGAGTGCTATGATCAGGCGTGCAGAAAAATCGGCATGTCATAAGACATATACCG
ACAAAAGAATTTTGTGGAGCTCGGGCAAATTATTCCCCATGCCACGCCGAGGAGACGA
GCGGCCTATGCCGACGTTCTCTCGGGGTATGCCCGTCCAAGCTCTTGCTCCGCAACGAC
TGCTTCTGCTTCGCGCCGGGGATGGGGATGGGGGTCGCAGGCGCCGCGAAACCCGTC
ACGCTTCCCGACGCGTACCGGCTGAGCGCCCCCTGCGGCGTCAGGGGAACCATCCATG
GGGATGCCTGGCCGGGCGACAGTGCAGGTGGCATGCTCTCGTTGCGCAGCGCCTTGCG
TAGCTCGTCGCGCACGCGCGAGAACGCACGACCGCGGCCACGTGCCCCGACGCTGCT
CGGGTCCATGTCCGGGTCAGCATCTGGACTGAGAGCCCACCGCTCGTCTTCGTCCAGT

GTCTCGAGTTCCGACAATGTGAGCCCCAATTCTGGAGAGGTCAGCAGGGGGCCGCGA
GGTGGCGACTTTCGGTGATTCAGCGCTGCATCTAGAGGAAGGTCCTTCGGGGTAGCCG
GCATCTCGTGGGGGCTGCGTGAACGCGACACCGACTCGGACGGTTCTGTCTGCAGGC
GGTTGCGGGGTGCACGGCCGCGCTCGAGATCCATGTCAGGGGCATTTGATAGACTAGG
CTGGAAGATGATGACGGATTCTTGAGTGCCTGAGTCCGATGATGCGGTAGAGGTCGTG
CGCAAGATAGCGTTAGTGAGCGATGAGAGGCTCGGCTGCGAGGCCAGAGGATGCACA
CGATGGGTTCTCGTCTGGAGGAGAGGATGAATGCTCGGCGCATTGTCATCGCAATGGG
GAGGTGGACGAGACTTGCTCCGGCTGCACGAACGAGCGAAAGTCGAACGGCTGGAA
GAGCGTGCTGGACGGGACACAGAACGGCCACTGTCCTCGTCGTCCAGGGCGTGCTCT
TGCCGCGTCTGAACACGAGATCGGGACCGAGAACGCGACACAATGGCTTCAGACGGA
GGCGTAACGTCATCGGCCGCTGAACCCCAGCCAATGCCCCATGCTACATCGTCAACCAT
GGCAACGGTCCACCGGTTGGTAACTGACCGATCCATGCATCCCCGGAGCGCCTGCTCT
GCACTGCGTCCAATGCCTTGAGGCATCTCAAACACGCCTGCCCGATAAGAGGCGATGA
TGAGCCTGTTTTCATCCGCTCTGAGGTTGTTACGTACCGTGAAGAATCTTCATTTGCAA
CCGTGGCTCAAATGAATCAGAAAACGGAAGCTTCCCGGTCAGAAGGGCAAAGAGCAT
CACTCCCAATGCCCAAATGTCTTGGGCGGAATT
>c10001_g1
TGCCTTTTCGACGGTCAAAAATCAATCTGGAAATGCCCGCCGTGTACCTTCAAGTTGG
GTCCTCTTTGTGACCGCTCGTGGACACAGGGTCCACGTTGACGCGAAACGCGAATTGT
GCAGGCGACCCAGGCGAGGATCGCCGTTACGTGGTGGTCTATACCTTTATTGCTCACGC
ACAGCGGCACTACATTTACTCCAGCGCTGGCGCACCTCCAAGCACACGCCTCCTCATC
TCTCTCCTGCCCAGACCCTAGCAAACTCGCTTGTCGGTTACCGTCCCGACCTGACCAG
GATTCCACTTTCTTTTCCAACCGCCCGGCTCGCGATGTTGCACGTACCGCCGCATCCAT
CCCCGCCCCCCGCTACTCCGCCAAAGCCCGACATCCTCCGCGGCTGGATCAAGACTAC
AAAGGACGCTATCCTCGTCTTCGAGGCGACACGCGCAGGCCTCGTTCCACGCGTCACC
CGGCGGTTCCACGACCTCGAAAAAAAGAGCATCATCCAGAGCGGCGCCGTCCTCGTCT
TTACCGAGGAAGAGAGTGGGATAAAACGCTGGACGGATCCATACCTGTGGTCGGCCTC
CCGTATGCAAGGGAATTTCTTGATGTACCGCGAACGCGAAGACGAGGATCCCGCCTCG
GAGATGGGTTCTCCGTATCGCTGTTCAGCTATCGCGCCCCCCGCTGAATCGTCCGACCA
GCTCCTGGACACGGAACTCGAGCACTACATTCTGGGCAGTTGGAACAAGGGCAAGGG
GCTCAAGGAGAATGGTCTCATGAAGAAGACGATCTCGATGACCATCCAAGGGACGACC
TACCACCTGATATCGTACTACTACCCGTCCGATGTTCGCTCTGGTGTGCTTAAGACGCCT
ACCCAGATGGCAAGGTTTGAGGGTCTAGAAATTGGTCCTGCTATGCTGGCGAGTCTATC
GCAGTTTCGCCAGCCACCGTCCGTGGGGAAGCCCAGGCGAGGGAGACCACCGCGCTC
ATTCCGCAACATGTCAATGCCAGTGAACGATCTGCCAGTGCCGCCGACGGGATTACTC
GACCGACCCTCAGGCTCACCCTACCCATCTCCCGCAGATTTTCAATCCCCTCAGGACTC
GGATCCTTTCCTTAAACCAGGCGATACGCCGGCATCCGCTCCTTGGCAGCGGTCTTCCC
GCTGTCAACCTCACGATCATATGAGCCTTGGCAACCCTACGGGCCAACTTCCATCCGGG
ATGGTACAACCTGAAGATATAGCCGCACCCATCCCTCACTTTTATCGTCACCACAGCGC
CCCGGAACTTGCTCACCCCTACTCCCATGTTCCCCACCCGTCGTATCACAAGGCCGGCA
TCTCCGCTAGCTCACACTCGTTGTGGCACCACACCCCCCAAACATTTATACCCTCCTCT
CTCGCAATCGCGCACGAAGCCCGCGTCCCTTCGTACGAGCTCGAGTACTCGGAATCGA
CGGGGGTGGTGAGGTATGAATGCGAGCTTCCGAGTCCCCCTGTGTACGTGAACCGTGA
CGTGCGTGCTCAACAGGCCCTGATGCGTACCTGGGAGCCCGTCATCGAGGAGGCGCG

ACATTTGGTGCCCTCGGCAACGGGGGAGAGGACCGGGCGGGATAGCAACTTGCCTCC
GGCGGTCGTCCACTACGCTCCCTCCGAGCAGTACTACCCATCACCATGAACCCGCGAT
GTTATTTGGTACGTGCATGGATACGTACGTTGAACACATTCGTCTATTACCCAGTTACGA
TATGGATTTGATTGTACAGTGATACCCACCCCTGCCTGTTTACGACGTTACGAAGTTGTG
ACGATTTTTCCATTTATCTGTATATTACGATATAGATATATATGATGTAGTGTACTCACCCA
ATAGACGAATGTCGTGTAGTACATAGGTCCGGGTCTAGTATTTCCTTCACGACCACCATA
GCTACATATGAGTTCTTGGTTGTATTAGTTGAGTTGGGGGCGTTCCGCGACACCCCTCA
CGCACACTCGCCGACAAAGTGCACCTACTTTACTTTTGTGATATTGGAGTCCCGTCAGT
ATCAGCTGGATGCACACAGTGGTCGTGGAGATTTCCCAATACAAGGCTCGGGCTGCCA
CTCAATAAGATGATGCGCAGGTTCAGGGCCTGGGCTCACTCAATTGCCCTCTAGTGTGA
GAAGCGGTGTGTCTTCGTTGCCTGACGCCGCAGGTTCGGGAACAACGAGCTCGGTAC
CGATGAACTCATGCGTCTTCTTCTTTCTTTCATTCAACCGGATCCATCCTAGTCGGGCCC
TGGTAGATATCATGCGCAACGAGGGGTGCAGGACAAGAGCACCACGAGCGGCCCTGC
GAACTCGAGGACGCTGATGCTAGCTAGAAAAGGATTATCGCCACATTCCCGTAAGTAC
ATATTCAATGGTCGGCTGCGATGGCGCTACGCGTACATACGGGCTCGGAAGCTACAAAG
GTCGTTCGCCGTCGTCCTCGTTGCATGGAGACCTGCGATGGAAAGTGGAAAGCGATGC
CAGGTCCACAGAGATTGGAGCATACGCGAAGCAAACTTGATAGAAGACGAGCTTCGG
ACCACGACCACCGAACTCGCTCCGAGGAAAGTATCGCTCGCATGGCGAGGTTGGTCG
AAGCTCAGTGGTCCACATTCAAGAAGCGCAGAGAGATCGCCGGAAGAAATGAGTTGG
ATTAATTTTGATAGGCAATTGGGAAGGACACAAGCGAGATGGGTCGTCAGCATATCATG
GTCTCAACTTCGACTCGAGGAGGTGAGGAGCTGTACATCCGTGTCATCCCTCAAATTAT
TAGTGGGTGATGCTTCATGTGTCATGATGAGAGGAAGTTTACTTTCGTTAAGATCTTGT
CAAAGTTAAGCCCATGGCATATTCTTTTGGAGAGACAATCCTGCTGCCGACGGATGTCC
TTCCATACTTTCACCGAGAATCTTGATTGCGACTCTCGCGAGCTAGTATCCCGCCATAAA
GTCACGCGTCATATTCGGAGTTTGTCTGGGTCCGAAGTCTTTGCAGAACAATCAAGCTC
AGCTTCATATCCCAGGTCCTACGTCAACATCTAACTAACTATAGAGCGGGAATCAAGAA
TTGTCCCTAAACGTTAACGTGAACGCCTCCATTCTCTCCAGTTCTCTTCTCTCGAGTCC
CCATTGTATCTTGGACGCACGCACTTTATGCTCTAAGAGCTCCCGCGATACCCGGGTAA
TAAAAGACAAATGGCGCAAGTAACACAGTCATCTTCCGCCTCAATATCCAGTGGTCCA
CGAAAAGACGGCGGCCTTGATTCCCTCTATCAAGTCCGATTATTGTCGGCTTTACGAAG
CGGTGATCCCGCCCGCATTCATCCATTCCTCACCGATGTATCAAAGGTTCGACGTACGT
CGTCCGAAAGCGATGTTGACCTTGGTGCTGCAGCGCTGCATCTAGCCATTCGATGTGCC
ACATGTGATACCGTCGCCCTCTTGTTGTCCCATCGCTCAATCTCTCCGAATTCCGTCTAT
CCTCACGGCTCTGGCACTACCGCTCTCCATCTAGCCGCCTCATTATCACGCACTGATGTT
GTCAACCTGTTACTTGACCAGGAGAACATTGACGACAGTATCCGTGATAGTCAGGGCA
AGACATGTCTAGAGGTCGCGAGAGGCAAAGAGACAATAAATGCTATCCGAGATTCAAG
AGCTTTCCTTAACGCTTCCTATCGCTCTCTCTTGCGTACCTATATTCACTCAAGCCCCAA
CACTCCTCCCCCTGAAGCGCTATTGACCGTATTGTCGTCACCTCGTATACGCCAGGTAG
ATCTGTCATATCTTGACGATGCCACAGGACGTGCTCTCTTGCATGAAGCTGCGCGGCGC
AAAGACTTGCGCTTGATAGAGTTGGCTGTTCGCGCTGGCGCGGACATCTTTGTACGCG
ATCGCAAAGGCCGGGCCGTCTATGAAAGTGCAGGCAAGGATGACCGAGTTCGAGTATT
CCTGCGACAGTTCACCAATCAGGATAATTCGTTGATCAAAGAATCATCGGAGCCGCCA
GAACTCAGAGGGTATCTTAACAAATACACGAATGTAGCAAGGGGGTACAATACACGCT
GGTTCGTCCTACACCAGGGTGTTTTATCATATTACCGTCATCAAGACGACGAGAATTTA

GCTTGTCGAGGGTCTATTGCATTGAGAACGGCCATCTGTGCCGTCCCAAGCGGGTCGG
TCGGAATGCGCTTTGAAATTCAATCGACCCCTTCCCGCGGTCACTCGTCTACGCAGAA
ATGGTATCTGAAGGCTAATCACCCCATTGAGGCGTCTCGGTGGATCACGGCGCTACAGA
AGAGCATGGAGATTGCGAAACGTGAGAGCGAACACCAGGAACGTCAGAGCGGAGAG
AGCGACGTGCCGAGTCTCAAACCTTCAATGTCGATTAGCTCGCACCACCCCAGACGTA
TGGAGAAGGCCAGCGGAGCTACCTCGACGGTGAGCTCTGCAGTGGGCGACTTGGAGT
CGGGTGGGGAAACGGGTGAACGCAGAGGCGCGGTCGGGGATGACGAGCGTGACCC
TGAAGCTGAGCATGAGGACTCTTCTGACGCAGACTCCACGCAACAGGCTCCGCACGC
AAACGCCTTCGGGCTTCAGGGAAACGCGCTTCTTGCACAAGTCGAGCTTGCATCACAG
TTACTGTCGAGTCTCCCTCGTCAGATGCCAGGATCCTCGCGTGCCGCGGAAATGAACA
AGGCGGTCGAAGAGACGTTCCACTCTGTTTCCGGGATGCTCTCCGAGTATGTGCACAT
GGTAAAGGAACGCGAGGACTGGTACAAGTCTAAGCTGGAGCGCGAGCGCGAACGGC
AGAACATCTGGGAGGAAAGTCTGCAGGCGGTCGTGCGCGAGGGCGATATGCTCGAAA
GGGAGCTACGCAGCAAGGCTCGACGTCGCAGCCACGTCCCAAGTGTGGCGCAAGAA
GGCACCCTGCGAAGACGGATGTCTCAGCTCGTGTCGTCACCGACCATTATGCCTACGA
CTGCTTCCCCTACACAGCTGCAATCGTTTGAATCGGGGGGCGCGCCGTCTCCAGCAGC
GACTACTGCGCAGCGCACACTTTCTCGGAGGACGTCCGTTGCCTCTACTGGCTTCCCC
CTGTCCGCTGCTACCTCGCGTCCGTTCTCACTCAAGATGGGTAATGGACCGTCGTCTCC
TGGGATAGAATCGGAAGCGTTGGATGAAACGGGCGACACAGATGAAGAGGATGAGTT
CTTTGACGCGATCGAGTCGAACACGCTCCCGAACCTCGTCATCACCAAGTCGCTCATT
CAGCCCGCCCCGGCACAGTTTTTCTGAGCAGGGAAGTGTATGAGGGGTATATGAAAC
TGCGCGATAGTCTGGCGATCTCGACGGACGACAGGCCGCCGATGAGCTTGTGGGCGGT
GCTCAAGAATAGTATAGGGAAGGATTTGACGAAGATCTCGTTTCCTGTATTCTTCAACG
AGCCGACGAGCATGTTGCAGCGGATGGCGGAAGATATGGAATTCTCGGAGTGCTTGGA
CGCAGCGTACGCAGAAGATGATCCGCTTCGCCGAATCGCGTTCGTGGCGGCTTTTGCT
ATGTCGAACTACTCGTCTACCATTGGGCGTATAGCCAAACCGTTCAACCCCATGCTCAG
CGAAACCTTCGAGTACGTTCGGCACGATAAGCAGTACCGCTACGTGTCGGAGCAGGTA
AGCCACCACCCACCCATGTCCGCATGCTGGGCCGAGTCTCCCGTGTGGCGTTACTACG
GCGAGGTCGATGCGCAGAATAAGTTCATGGGCAAGTCGTTCGAGATCCGGCCGACCGG
TATTGCGCACGCAGAGCTGCTCCTGCCCGAAGAGCGTGCACCGGACTACCCGCAGGCG
AAGGGTGCGCATGTCAAGGGTCGGGTTGTAGAGCACTACAGCTGGAAGAAAGTCACG
ACGAACGTCTCGGGGTTCATTCTTGGCTCGCCTACGATCGATCACTATGGTGATATGGTT
ATCACGAACCATCGTACGGGAGACAGGTGTATCTTGACCTTCAAGCCGCGTGGTTGGC
GTGGAAGGGACGCATACGAGATTTCTGGGTACGTTGAGGACGCGGACGGCAATGTTGC
ATATGAAATTGCAGGACGCTGGAACAGTCAACTCGTGGCTCGTCAGGTCGGAACGGGC
GTTGGCCAATTACTCCCAGACATCGCGATGGGCTCCCCGTCTGCGTCGACTGAGTATAT
CCTGTTATGGCGGAACTCCGTGAAGCCCATCTCCCCCTTTAACCTGACGCCGTTCGCGA
TCACGTTGAACGACTGTCCGGAAGACGTGCTCAAGCCGTACCTGTGTCCGACGGACTG
CAGGCTCCGCCCTGACCAGCGCGCATTCGAGCTCGGCAAGTACCAGCGCGCGAACGA
TCTGAAAATCAAGCAGGAGGACTTCCAGCGCGCGACGAGGCGTGCGCGCGAGGAGG
GACGGGCGGCGGCCCATCGGCCGCGGTGGTTCTCGGCGACGACGGAGCCGGACACGG
GCGAGCGGGTGTGGATGCCGGAGATGGTGGATGATCGGCTGGAGTATTGGGTCGAGCG
GGAGAGGGTGTGGAAGGCGAGGCATGGGTCGGACACGGAGGCGACGTGGAAGGAG

GTGGATCAAATTTTCATTGATGATGAACCGTGAGTCTTTGCGCGATCCCTGGGGGTGGT
GTCGAGGACGAGAGAGGCAAAGGTGTGAGAGAAGGAGCAGGACATTGATTTATCCAT
TCCCTATTACGGTCGGAGGGCAGTATGCATACCAGCACATTCGGATCCGAAAAGGCTTT
GTTTGAGTGTAGTCTATCAGTATCACTAGTAATAGATGCCTATCAAGTAGGCTCTCCTCT
ACATAATTGCAGACAAAATCAAACCAACACAAAGCATATCGAGCTGGGAATCCATGGG
TGTGCAGGCCAGTGCCCAACGGACGTTCGGGGACATCCCGACAACAAGTAATGGTAAT
CCCCGTCTACCAATCTAGCTTCCTGTCTCCGCAGGACGTACCAGAACAACCCACACCA
GCAGTGGCCAGCGTAGTCTCGTTGGGTCCAGTGATCTCTACGGTGGAATTGTTGCTGA
GTCCTCGATCGAGTAGCTCAGCGGTGGCTTGTCTGATCATCCACTTTGAGGTGCCAATG
GCGTCGTCCAATTGATCTGATTCAGGAAGGAGTAATAACACGTCGTCGCCCTCCTCTTT
GACATCGAAAGCGATGATACCGTAGGAGTCATCGTTCAGGCAGTCACCTGTAGTCAGG
CTGAAGTTGCGCTTGTGCATTGGGCAGGAGACGTAAAGGACACCTGACTTTGGGTCGT
CTCCGATGATACCGTGGTCGAGGACGAATGCACGTCGATGAGGGCACATCTGTTGTGT
GGCAAAGTAGCCTCGCTTTGGGACGTGGTAAATGACAATCTGGCTGTCTCCGTATCGA
ACAGCAGCCGCCGTTGTTCCGCCATCACTGGGACTGAGGTCCTGTTTACTAGCCAGTTT
GTGCCACTTCCATTCGTTCTTCGGAGAGGGAACATCCGTTTCGCGGAGTCTAAGAGGC
GGGGTGGTTTGACTCCAAGCTGCCGGACGTGCTTGCCCTCGTTCGACTATTGTCTCCAT
CTGCCCAACTCGCTCATCGGTGTTCACAAACTGACGGAAACGCTTCTGCCGCTCCGGA
TCATCGACGACCGCCTTCCACTCGTCGCGATATGTTCCGACAAGTTCGCTCATCTCGCG
TTCCAAATCTTCACAGATGCCCAACTCGTCGTCCAGAACCACTCTCCGTAGTTTCTCGA
TACCACCTTCCATCGACTCTAGCCACCGCGCTGTCCGCATGAGCTTATCTGCTGTGCGG
ACATAATACATAATGAACCTGTCAAGAATGCGGATAACTCTAGATGGAGGAATGTCTGC
TGCTAGCAGCGTCGCATGTCTTGGAGTTGCTCCTCCGTTGCCACAAACAAAGATGTTC
CAGCCCTTGTCAGTTGCGATGAGTCCAAAGTCTTTGCCTTGCGCTTCTGCGCACTCTCG
CACGCATCCACTCACGCCGCCCTTGAACTTGTGCGGTGACCGGATTCCTTTGTAGCGCT
CCTCCAGCTGGATAGCCAGTCCGACCGAGTCTCCTATTCCATAGCGGCACCATGAAG
TTCCCACACATGACTTCACGGTCCTCAAGGCTTTCCCGTACGCGTGGCCACTCTCAAA
CCCGGCATCAACGAGCTCCTTCCATATAGACGGAAGGTCCGCCCTTTCGGCACCAAAG
AGATCTATTCGTTGTCCTCCCGTGATTTTAGTATACAGCCCATACTTCTTTGCCACCTCG
CCCAATATGACGAGTTTATCCGGTGTTATCTCGCCGGCGGGAACTCGAGGGACGACAG
AAAACGTCCCGTCTCGCTGAATATTCGCAAGGAAGCGATCGTTGGTGTCCTGATTCGC
GTGATGAATAGGGTTCAGGATGTGTTCGTTCCACAGCGAGGAGAGGATGCTACCTATC
GCAGGTTTGCAGACTTCGCAACCTACAGAGTCAGATCTGATGCCCGCTATCTTCATGAT
CTCTACAAAGGTCTTCAATTTCTTCAATTTGACTACGTTGAATAAGTCGGCCCTCGATAT
ATTGAAGTGGGGACACAGGTTGTTGTTAACTGCAATGCCGGCTTTCTTCAACTCTGCCT
TGATGATGTTTGTCATGAGTGGGACACATCCTCCACACCCAGTACCGGCTTTGGTGACA
ACCTTGACCTCGGCTACGGTCTGAGCTCCATCCTTGACAGACTGCACCACACGACCCT
TGGTCACATTATGACAGCTGCAAATCTGTGTGTCATCGTCCAGATCTTCGCCCAAGTCA
TCCTTCCCACCGCCTGCGCCTAGTATCAGCTGCGATGGCGGTACCTCAAGCGCTTTCTG
CAATGTATCCAAGGATCAGGCAAATATACGTTTACGTTTCGGCGCCACAACACTAGAAG
GCGCGAACAGTACCCGCCTTTTTCTTCACAATTCCGACCAACTTGACATAGTCTGACG
TGTCGCCGACCATCATTCCGCCAAGGAGGTACTTGCCGTCCGCGCTAAAGATGTATCTG
CACGACGATAGTGACAACACGAACCCAGACCCAGAACCATGAGCCGGCAGACTGCTA
ACCCACGTCGGTACTGTACCTTTTGTAAACGCCCCCAAAGGATCTCGATATATCAGTG

CTTCAACGGGTCCGTGGGCAACTGCTTCACCGCGCCGTCTCCTTGGGGTAGATGCATC
ATGCCTGCCTGGAAGGCTTGCACCAGGAGACTGTTGAACGACCGATATGGCTTGGCTT
GTCCCCTGATCCTTCGGGCGGTAGCTTGTTGGAGCGTCCTTCTCGGCGAAGAAGTCCC
CAAACGAGGCGACGTCAACACCCATCAGTTTCAGCTTCGTGGACAGGTCAGGATTGTT
CATCTCCCTCGGCTTGAAGCTCCCAATGTCGGTCTGTGCTTGAGTAAAGTTGAAAGCG
AGGATGTCCGCCATCTCGATGCCAGGCGCGATTAGCCCATAGGTGTTATTTTTCCAGCT
CGCGCACTCCCCGATAGCATACACATCTTTCGCGCTCGTCTGCAGAAGGTCGTCGACG
GTGATTCCTCCTTTCGGTGTGCATGCTATCCCGCCCTTGCGCGCGAGCTCATCTCGCG
GCGTGACGCCAATCGCGAATATTACCATATTACAACTCAGCGTGGAGCCATCGGAGAGC
TGCAGTCCTACGAGGTTGCCAGGCTCGTTTGTGATGAGCCGTTCAATGGAAGTACATCC
AAGAAATTTGACACCCAGGTTTTCGATGCGTCGGAGGACGATCTCGCCACCCTGCGCG
TCAAGCTGACGGGAAAGCGGGTATGTTTGCCGGTGGACGATGGTGACATCTCTGACTG
CAGGGAGATCGTAGACCGCCTTCGCCGCTTCAAGACCGAGAAGTCCTCCTCCGAGTAC
GACAGCCGAAGCGTCCTTCATGCCTCCCTTCTCCACATGAGCAAGCAGCTTGTGCACG
TCAGCAATGCTGCGGTAGACAAACACGCCAGGTACAGACAGGTCCGCAAAGTTTGGT
AGGGTTGCATCAGAACCTGTCGCCAAGACGCAATAGTCGTACGATATGGTGCGCCCTTT
GTCCGTTGTCACGGTGTGCGCGTCAGCATTGAGGGAGGCCACCAACTCTCCAATATGA
TAGGAGAAACGATGTGGATCCTGTTGAGCGTACCAATCCGGTGAGGTAAGATATAGCTT
GTCGATGTTACGATGCTGAAAATACTCCGTCAAAGCAACCCGGTTGTACGCAAGGAAA
ACCTCTTCTCCGCAAGTCACAATGCGGTAATGGTGGCTGCTATCCAAGTCCAACAACTT
CTCAATGAATGCAATTCCTACCATTCCCAAGCCGACCACGACAATAGTCTTACGTTCCA
AAGGCGAAGGTATTTCCATA

>c9468_g4

CCACGAACTCGGTACGGTAGGAATCGATGTCTTCATCGTTAAATTGCGCGAATTCGGAT
TTCTGAAAGTGAGCGACGAACTCGGTGGCGGTGCGTGGGACGCGATTCTCATAGCCTT
CGCGGACGGTGCGACCGTTCGGATCCGTCACCGCTACGAGGAAGTCGGCAGTGGTCT
GACGGTTGGCAGGCTCGTATCCCATGTCGATGAAGTACTGTCGGGCGCGATTTGCAGG
GCCGAAATATACCTGCTCACCCTCGTAGATGACACAAACCTTGTCAAAGAGTTCGTAA
AGCTGCTCTCCAGCTTGGTAAATGGCTACGACGGTGGATTGCCGAGCAATATCGGTGG
CAAGGCGTAGCGCATGCACGAATTCACGGGCAGTCGATGCATCGAGCCCGCGCGTGGA
GTTATCCCACGAGCTCAACCGGCTCCTCGTCGCCAATGCCTCACCAATCGACACGCGC
TTTTTCTCACCCCCGGACACTCCCCGGATAGACGCGTCCCCGACGAGGGTGTTCTTGA
CATGCCGTAGCCCGAACACAGTCTCGATGACCTCTGTGACGAAGTCGATATGCGCCTCT
CTAGACATCTGGTCTACTCGCGTATGAGGCGTGCGTGTCTGCACGGCGAACCGGAGCG
TCTGGTCCACAGTGAGCGTCGGGAAGTGCACGTCATCCTCTGGGCAATACTGGACGTC
TCCACGGTGATGTTGTTGAATGTCCTGCGGAGTGAGCGAGTCGTACCACACCTCGCCT
TGCACTGAATGATAATCGTCTCGCTGGTTTGCGAGCACCTTCAGGAGGGTACTGCACCC
TGAGCCGGGACGCCCGAGGACCAAAAGCATTTCGCCTGGGCGAACAACGCCGCTGAA
GCCGCCAAGTATATCGCGCGTTGCGGGGTGGACCGCGTTGCGAATGTTTCGGGTGAAG
TTGAGCGGGTTAAGGGCCGACCCGAACGTGGGCTGATACGAGGCAGAAGCACCTACG
CCGACGACGCGGAGATTCTTGAACATAACACCAAGCTCTCGCCGTTTAATGTCGGACT
CATCGAGCTTTTTGAAGACTCTCCGCAGCGTCTTTTCAAAATCAAACGGTCCCTCGCTT
TCGGGGTTGAGCGTAGCGTCCGTTCCAGATCGTATGCTTTGGGAGCTTGTGCGGGTATC
GTAGGGCTGAGGCTGAGGGGAAAAATGGGCGCCTGCATCGGCGAACGCCTGCGATTC

GTGACGGGACAGTGTGCGACGAAGCTCCTCAACGCCTTTGGGGTCAAAGAAGCCGAT
GTCCACGCGTGAGCCTGAAGATAGTTGCCTGGAAAGAGGATTACTACTGTTAGCGTTG
TGATTTTCGCGGGCAGGAGAACAAGGATGGTCTCGATGATGGAAATTGCGGGCGGCAG
CGTCGCCAAAGGCAGCTGCGATGTGAGTAGCGGTGGGTAGCTCTGGAACATTGCCCCT
GCTCTCAAGAGACTGGAGCTGAGCAGGGTCAGCCATGGTGGTGGGCGAGAGTCAAAG
GCAGAGGGGAGGTCGTAGATGAAATGGCGGGAATGGGTCGTGCGGATAACATAATGCA
AAGCTGGACCGAGATGAGATATTGGGGACGCGAGGTTTGGATTGGGCCTGGGGGTGG
ACATACTCGATCGGGCGAGAATTGCCAGCCAGCTCCGATGGCCTTACGAAGCTACTGC
CCGTCGGCTCCACACCGTTCTTCCGATTACGAGCGACGTGAATGTAGACTCCGTCTTTC
GGTAGATTGGGGGAATCATCCGTGCTTGACTTGGTAACATCCTGCTTGTCCGCTTGCAT
CGAGCGCAATAGTATTTCCGCATTATTGCGTCATCGTTAGGGGGGCACGACTCGGTATG
TATCACCGCGCGGCGCAAATGCATGAGTCCGTGGAAATCGTGGATGGAACCATATCAA
GGCGCACTCCATTAATATTTATGCTCAGTTGCGGTGCAGTAGTACTTAGTGGGGCTGCG
ATTCAACACGAATGGGTACGAGGCCAACAAACACGAGCATGTGCATCGGGTCAATTCA
AGTAAGATTGGCCTGTGGTTGTGCAGCGCCGCAATTGTCAGATGCAGCAGCCATACGT
CTCTTCTCCCCTTACTACTCCTCGCTATCATTGCGGAACCCATCTCGTATTTCTCCCTATC
CGCCGTCAATCCCGGCTTTCGCATTTGCCGAAGATCCACCCACTGGCAGCAACGGGAC
TCACAAACATGGCGGAAATTTATCAAGAAAGTTGCGTGGTATATGTAGTATGTACAGGA
GATTCGAATTCCTGTGCGCGAGGGAATTGAGTTCAACAACGGTCGTCATATTGGCATCA
GAAGCTGGGAGTCGTTTTGGTAAATGTAAATGTGGTGGATGAGGCTCGTAACTACCCG
CGCTTAGTAAGCCCTGATCTCGTTTCGTTATCACGACGCTCAAGCTTGGATCGACCATA
CTCTGGCCAAGTAATCTATGTACTTCACGGCTCATTCACCTGGGGCACCCTGCGAATAA
TTTTGAACCATCTGCGTGTGCTCGGTGCGTTCTTGCGCAACTTGAAAGTCCTAGTGGTT
GGACTCGCTCTGCAGTTCCTCTAGAGAATATGTTGTAATCTTAGCGTTCCTCGTGTGAG
TGTTGTCTGATTCTCCAGTACTAGTCGGTGGCAGTGTAAAAAGGAGATACTTGGGATGC
CAATTGTCTAAGTCTAATAGTCACTGCCTTCAAGCCGCCCTCACAGATGCTGAGGACTG
CGTACTCGCCGACACGCATCACTGAAGGCCTCGCTGTTGGGGATAGCTATAGCGATCTC
GGTACAATTCCAGGAGACCAGCAAATCCAAATTTCAGACCACTTCCGCCCGGCCCGTA
TGTCCGTGCCATTAGGATTTCGTTCAGCTCAGGCTCTGGAGTCACAACGGACTCGGCA
CCGCTGGCAGCGATGACGCTGAATAATCCTTCTTCAGCATCAATGTAAAGCCGCATACA
GTCTGGAGAGTCCTCGTGTTCATAACTAGATTACAAAGCTTCATCTCAAGCCTCCACGT
CGAACATGCTGCTGCGATGGATTAGTAAGTCTTGCAACTGTCTTACATCTCACTTGCCT
TGATATGTCAGCTTGGAAGAGGTCGGTCAATTGCTGCAATGTGTCAGCTCAAAAAATT
GGACGTCTAACTTGCTGCCATGCGTAAGCCGCGCCATTGGGGACTATATATTGATCCAG
GTCCTCTCTTGCGACAAACAAAAGCTCCCATCTATCGATCAACAGCCTAATTAACTACT
CATGTCGCACAGCAAGCAGTTCACGCTCTACACCCACATCACGGGCCCCAATGGCTGG
AAAGTTGTGTTCGTGCTCGAAGCGCTCGGCCTCACCTACCACAGCATCTACCTCGACTT
CGCAAAGAACGAGCACAAGGCCCCCGCGCACACGCAGCACAACCCCAACGGGCGCA
TCCCGACACTCATAGACCACCAGAACGGCGACTTTGTGATCTGGGAGTCCAACGCGAT
CATCCTGTACCTCGTCGCGCGGTACGACACGCAAAAACGGCTCACATACACCGACGAC
AAGGACCAGTACAACGTCCTCCAGTGGCTGTTCTTCCAGGCATCAGGGCAGGCACCGT
ACTTTGGCCAGGCCCACTGGTTCATGTACCGCCACGAAGAGCGGTTCCAGAGCGCGAT
CGAGCGGTATCATAACGAGACTCTGCGAGTGCTCGGCGTGCTCGACGGTGTGCTCGCT
GTCCGACCCAGCGGCTGGCTCGTCGGCGACAAGTACACCATCGCGGACTTGTCGTTCG

TCCCCTGGAATCGCACCGCGTTTGAATACATTCTGCCGCACATCGAGGGCGGCGTCGAT
GTTGAGAAGCGCTTCCCGGCTGTTTATGCGTGGCACAAGAGGTTGCTCGATATCGATTC
TGTGAAAAGGGCGGATGAGATCCGTGTAGCGCTTGCAGCAGCTCAGTAATGACTCGTA
GCGTTTCGTCAACACACTTCAGAAAGATGCCGCTGCTGCCGAAGCAGTTTACAAACAC
GTGAACTTGTAATCCTTGTTCATGCTAGACGTACTGGATAGAATCGACGACTGAAAACT
TGCACACCCGC

>c6055_g1

CTCACTCACACCCACCATGTCTGCACCCCTTTTACTGTACACCGGGGCCACCCCTAACG
GCCACAAGGTTTCTGTGTTTCTCGAGGAGCTCAAGCTCGCATATGGTCTCCAGTACGAC
GTCGAGAAGATCGACATCAGCAAGAACACGCAGAAGGAGCCGTGGTTCATCAAGCTG
AACCCAAACGGCCGTATCCCGACCATTGTCGACCGCTCACGCAACAATTTCAACGTGT
TCGAGTCGGCCGCCATCCTCCTGTATCTCCAGCAGCACTACGACAAGGAGAATAAGTT
CGGCTTTGACAGGGAAAATGACCCGGACAACTACAGCGAGATGCTCCAGTGGATCTTC
TTCGCCCATGGCGGTGTTGGTCCGATGCAGGGCCAAGCGAACCACTTCAACAAGTACG
CGCCCGTGGACATTCCCTACGGAAAGAAGCGCTACACTGATGAGACAAAGCGTCTTTA
CAGCGTAATCGAGATCCGCCTGCAGGACCGCGACTGGCTTGCTGGCCCCGGACGCGGT
AAATACAGCCTCGCAGACTTCAACGTAATCGCATGGATCAGGTTCCACAAGACCGCCG
CCATCGAGTCTCTCGACGAGTGGCCTCGTTTGAAGGCGTGGGTGGAGGCTGCTGAGGC
TCGTCCTGGCTTCCAGGCGGGTGTCCGTGTTCCTCAGTGAACATTGGTATACTCCATAG
TGAAAGAAATCTTGGTTGTAGCACATAGCACTCAGTTGTTTTGCGATAAACACAGGGA
GGGGGAAATATAAATGGATTCTTCGATGACGAGATGGCA

>c6600_g1

ATCACTTTTACTATGGCCATTGTACAGATATCACATTCATAAGCCTGTCTAACTAACAAC
AATGGCGACGAACGGACAGGTACTATGTACAAACAGTGAGATGAGCAGAACCGGATC
GGCGGGTCCATCGGGATGTGATGGGCGCGCCGATTGCCGAAGTGCCTCGTGAAGTCAC
ATGTCTATCTCGGTGTTTCATCCTTTTGATGACGCGAGCTTCGCGTCACAACATTTAGTA
ACCTGATCCCCGTCGGCAACGGGACCGCGGTCACTCTGTCCTGCCGCTGCACGTTTGT
ATGGCTGGGCTCAACACCACAGCCACACCACAAAATAAAGACGTAACAACAACAACA
AATGCGTCAATCAACTGGTTACGGCGATGGGGCCGCGCGCGCCTCAAAGCCCGACGA
GTCGGTACCCTATGTCCGTGAGGGCCCGACACGGTCATAGAAGGCCTCGCCTGTTCCA
GTTCCTGCTGCCCTGCTCATCACATATTGATTGATTGTTGGACGGATATAAAGGCCTGCA
GGGAAGTATGCGGTTCCGCATCGACCACCCAGCACAGCAACGACGCGTCGACAAATA
AATCGTAGTACCGCCTGACCTACGCTCTGACGACCCAACCATGTTCTCCAAAGCCTTC
TTCTCCCTCGTCGTGCTCGCCGCGTCGGTCACTGCTGCTATTCCATCCATCAGCAGACG
GGCGACGTGCAGCAATGGCAAAACTACTGCGAACGATGCGTGTTGTGTTTGGTTTGAC
GTTCTCGACGATATTCAAGAGAACCTCTTCCACGGCAGCCAGTGCGGTGAAGATGCGC
ATGAATCTTTACGCCTTACCTTCCACGACGCCATCGCATTCTCTCCCACATTGACAGCTG
CGGGACGATTCGGGGGAGGAGGCGCTGACGGCTCTATCATGGCACATTCAGATGTCGA
GATGTCCTATGCAGAAAACGACGGGCTGGACGAAATCATTGAGGCTCAGCGCCCGTTC
GCCATCAAGCACAATGTGTCCTTCGGCGACTTTATCCAGTTTGCTGGCGCGGTCGGTGT
TGGGAACTGCAATGGAGGACCGCAGCTCAGCTTCTTCGCGGGACGCTCGAATGACTCA
CAGCCCGCCCCAAACAATCTGGTCCCGCTGCCGTCCGACTCTCCGACGACCATCTTGA
ACCGTTTCTCTGATGCTGGTTTCAGCGCTGTCGAGGTTGTCTGGCTTCTTATCTCGCAC
ACCGTCGCCTCACAGAACACAGTTGATCCGTCCATTCCCGGCACACCCTTCGATTCGA

CGCCATCGGACTTCGACGCCCAGTTCTTCGTCGAAACGCTGCTCACGGGTACCCTCATT
CCAGGTGACACCCTAACGAACGGCTCGGAGGTCTTGTCGCCCTACGCGGGCGAATTTC
GCCTTCAGTCCGACTTCGCCCTCGCTCGGTGCGTGCAATCGATCATTGCCCATTTCTCT
CTGCTGATGCAATTCTATCTCCGCAAATGGTATCTGCAGGGACTCACGGACTTCATGCG
AGTGGCAAAAAATGATTGGTGTGTACTATCTCCGTCCATAATGCCCTCACCATCACCGC
ACCCGCAATCAGCGGGACGTCTAAATTCCCTCCCGAGAACTGACTACGCCTCCTTCCA
CAGCGGACCGCGCAAACATGCTGGCGCGCTTCGAGCAGGTCATGCTGAAGATGTCGCT
GCTGGGCTTCAGCCAGAGCGCGCTGACAGACTGCTCGGACATCATCCCCATTGCGACT
GGTAATGTCGCGGATCCGTTCCTGCCCGCCGGCAAGACGCTGGACGATATTGAAGCCT
CATGCTCGTCGACGCCGTTCCCATCGCTCTCTGCGGCTCCGGGTGAGTGCTTCTTTCGT
TGAAAGAGACGCCGGCGTTGTTGATATGAGCATCCGCATTAGGTCCCGAAACTACCATT
CCGGTGGTGTGAGTATCTGCCTTGCTTACGAGTTGAGGCGCTGACTCTGCCTTATTCGT
GACTAGGCCGCTCGACTCGTGAGAGGGCAGTGTAGCTTGCGCTCGCGAAGACAGAAG
ACCAACTATTATGGCATGGGGTTTGACGGATTGTTTCATTTTCATTGGGTTGATAGTATC
TTCGGTGTCTTCTTAATGGTCTCTTCTTGACCACTAATAGGCAGGCCGACTGAACGTTT
GCGAGTGTGTTGTTGTTGTGTGTTCATTCAGTGGCACTCCGAACCAAATAGATGAAGTC
GTCCTGGCCGGATTCCCCGCTCGCAAACCGATGTTAGCAACGTCACTGTTCCTTCTGAT
GAGATCCCAGACAACGGTAGTTGGTATACCCAAAGGATTACAGAAGGTGGCTGGCCGA
TCAAGTATTCAATGGCACACTCGATGAAAAATCAAAACTTTGGGCGTTCGTCATGTGTA
TATAGTTGTTTGCTGTCAAGGCAACCATCTTGTATGATGCCAGCGCGTTGTACGAGCATT
TTCTTTACGCGGAAGCGGTGCCCCCTTCTTACCCGTCTGGTATGGGACTAGTCAACTTG
TTGTAGTGCGTCAAGGTCAAGCCAATTCCAAGAAGGTGCCCGTCTCCCCCGCTGCCGT
TCCGTCGGACGTTGTGCCTGTAGATGTCGATGTCAACGGGCTCGACATGAACAAAAAT
GGGATGAGGCCGGTGGTTGATCCGTCGCAGCCAAAGAGGCCCGCGGCCAAGAGGCGT
CGCAAGGCAGACGCTATTCCGGAGGCCGAGGTCAAGGCGGAGGACATGTCATTGGCC
CAAAGACGGCCACGTCGT
>c6769_g1
ATTGGAGTGAGAGTGAGAGTGAATCACTATTTATAGATTTAGTTGGTGTGGCCGAAAGC
CCGGCTCCGCAAAGCTCATATTTCGGGACTTATACCGTCGTTATCATCTTCCCATGGCAC
TCTCCTAAATTTAGCGTCCTTTTTTTAGTGTTGGTAGCATAACTGGGATGACTAGATTCC
TCGCGCAGCCGACATTGCGTGCAAGAGAGCCATCGGGAAGCAAGACACAAAGAAGGC
ACAGCAGTCCACTGCTCGATAGCTGATTGTGTGCTACCGACCGACGTTGATTATAATCG
CTAATCTTCACCCGAGATTGTGGAGGGTTATGTAGTTCCGTAATCATCTTGATGCTGCCA
GCAGCTAATGGTCCTGAGAACTTGGGGCCTCTATTCATTTGTCAGCCGAGAGTCCGATC
CACCTTGAAACAACCAGGATTAACATCCTCATGGTCATTGTCAAATGAGGGTACACCGC
GTCCTCTGGGCACATTACCCCATGCCCTCGGTTTGGTGAGTCCCACGTCTATGGCGTAA
TGTGAGTTCCAGGCGGTTCACGGTCAAAAGTCCCGAATGTTTCCACACGTCAGATAAG
GCTTGCACGTCCGTTCTTTCTTGTGCAGCTAATATCATATCCGCTAGACGTTGTCAAGAG
GCCCGTGCGCATACGATACCCGAGTGGGTGTTGGGATTTATCCAGATCTGTTACCTTCCT
GCCTTCGAGATCGAGCGAACAAGATTAGCACTGGCTTCAGCAACTTGCCAATATGCATA
TCATGTCGAGTACCAGGATATTTTTATCGGATGAAGACACGACTCTGTAAAGAAGTCAC
CGAACCGTTTCAGTGTATTGTGAAATGGCGTACCATTGGGCGTACCGCTCAACTACATC
GACACACCGACTTTATGGTTGTGTCTCCCCGCCGTTGCGCTGGTAGACCTATGCGCACG
GTGTCGGGAATAACAGAATCTGTGCAACGCGATTGGTCTGACCGGCTGCACAACAGCC

GCCTCTGCTCAATACGTCCGAACAGGCATCGGTCACGCTGCTCTAAAGGGTACCGCTC
AATGTGCTTGTCGAGACGTTCTTGCAGGTATAAAAGGGGTTCGTAGCGGCCTCCAAGT
CTCTTCAGGGCATTCAGCTTCGTCAGCTCTTTCCTCCTCAATCAAAATTGTTCATCAGCC
ATGGCTTTTAAAGCTCTGCTTTCTATCGTCTCCATCGTCGTCGCTCTCCAGGGTGCTAGT
GGTACTATCTGCCCAACTTCCCCCTCACATTCGAGACCGGGAGTTTCTGACAGAACCAT
ATTCCTCAGCTGCACTCACCCGCCGTGTCGCCTGTCCCGACGGTGTCAACACTGCGAC
CAATGCCGCTTGCTGCAGCCTCTTTGCGATCCGCGACGACATCGTCCCTAACCTCTTCC
ACAACCAGTGCGCGGAGGAGGCCCACGAGTCCTTCCGTCTCGTCTTCCACGACTCCGT
CGCGTTCTCTCCCGCCCTGACCGCTTCTGGCCAATTCGGCGGTGGAGGTGCTGATGGC
TCGATCTCTCTCTTTGCTCCGATTGAGACCGCCTTCCACGCCAATGCCGGTCTCGATGA
GATCGTCGCACTTCAACAACCATTCATTGATCGTCACAACATCTCAACTGCGGACTTCA
TCCAGTTCGCTGGCGCGATGGCTCTCTCCCAGTGCCCTGGCGCCCCTCGTGTAAACTTC
ATGCTTGGCCGCCAGGACGCTACTCAGCCCGCGCCTGATGGCCTCGTCCCCGAACCGT
TTGACACCGTCGACAAGATCCTCGCCCGCATG

>c43_g1

TGCTTACTCAGCCATTTTACCTATGTTCATTAATCGTTGATTATTCTCAACCAATCACAAA
GATATCGGAACCCTCTATCTACTATTCGGAGCCTGAGCGGGAATAGTGGGTACTGCACT
AAGTATTTTAATTCGAGCAGAATTAGGTCAACCAGGTGCACTTTTAGGAGATGACCAAA
TTTACAATGTTATCGTAACTGCCCATGCTTTTGTTATAATTTTCTTCATAGTAATACCAAT
AATAATTGGAGGCTTTGGAAACTGACTTGTCCCACTAATAATCGGAGCCCCAGATATAG
CATTCCCACGAATAAATAATATAAGTTTTTGACTCCTACCACCATCATTTCTCCTTCTCCT
AGCATCATCAATAGTAGAAGCAGGAGCAGGAACAGGATGAACAGTCTACCCACCTCTA
GCCGGAAATCTAGCCCATGCAGGAGCATCAGTAGACCTAACAATTTTCTCCCTTCATTT
AGCTGGAGTGTCATCTATTTTAGGTGCAATTAATTTTATTACCACTATTATCAACATGAAA
CCCCCAGCCATAACACAGTATCAAACTCCACTATTTGTCTGATCCGTACTTATTACAGCC
GTACTGCTCCTATTATCACTACCAGTGCTAGCCGCAGGCATTACTATACTACTAACAGAC
CGCAACCTAAACACAACTTTCTTTGATCCCGCTGGAGGAGGGGACCCAATTCTCTACC
AGCATCTGTTCTGATTCTTTGGGCACCCAGAAGTTTATATTCTTATCCTCCCAGGATTTG
GAATTATTTCACATGTAGTTACTTACTACTCCGGAAAAAAAGAACCTTTCGGCTATATAG
GAATAGTATGAGCAATAATGTCTATTGGCTTTCTAGGCTTTATTGTATGAGCCCACCACA
TATTCACAGTAGGATTAGATGTAGACACACGAGCTTACTTTACATCAGCCACTATAATTA
TCGCAATTCCTACCGGTGTCAAAGTATTTAGCTGACTTGCAACCCTACACGGAGGTAAT
ATTAAATGATCTCCAGCTATACTATGAGCCTTAGGCTTTATTTTCTTATTTACAGTTGGTG
GTCTAACCGGAATTGTTTTATCCAACTCATCCCTTGACATCGTGCTTCACGATACATACT
ATGTAGTAGCCCATTTCCACTATGTTCTATCAATGGGAGCAGTGTTTGCTATCATAGCAG
GATTTGTTCACTGATTCCCATTATTTTCAGGCTTCACCCTAGATGACACATGAGCAAAA
GCCCACTTCGCCATCATATTCGTAGGAGTAAACATAACATTCTTCCCTCAACATTTCCTG
GGCCTTTCAGGAATACCACGACGCTACTCAGACTACCCAGATGCTTACACCACATGAA
ACACTGTCTCTTCTATAGGATCATTTATTTCACTAACAGCTGTTCTCATCATGATCTTTAT
AATTTGAGAGGCCTTTGCTTCAAAACGAGAAGTAATATCAGTATCGTATGCTTCAACAA
ATTTAGAATGACTTCATGGCTGCCCTCCACCATATCACACATTCGAGGAACCAACCTAT
GTAAAAGTAAAATAAGAAAGGAAGGAATCGAACCCCCTAAAATTGGTTTCAAGCCAAT
CTCATATCCTATATGTCTTTCTCA

>c1151_g1
AGTGGTTTGATGCGGTTGGCTTGAAACCAACATGTGAGGGTTCGATTCCTTCCTTTCTT
GCACTTGTACAAAGGCTGGTTCTTCGAAGGTGTGGTATGGGGGTGGGCAGCCATGAAT
TCATTCGATATTAGTGGCAGTTAATTCGGGTTGGAGTACTTTTCGTTTTGCTGAGAGGGC
TTCTCAGACGATGAATATGAGTATGATTACGGCTGTTATTGAAATTAAGGAGCCGATTGA
GGATAGTGTGTTTCATAGTGTGTAGGCGTCTGGGTAATCTGAGTATCGTCGGGGTATTCC
AGCTAGGCCCAGGAAATGTTGGGGGAAAAAGGTTAGGTTAACTCCGGTAAATATTACT
CCGAAATGTGCCTTGGTTCATGATGGGTGTAGGGTAAAGCCTGTGAAGAGGGGAAATC
AGTGGGTAAATCCTGCTAGAATGGCAAAAACTGCCCCCATTGAGAGGACATAGTGGAA
GTGGGCGACTACATAGTAGGTGTCATGAAGGGCAATATCTAGTGATGAGTTAGCAAGG
ACGATTCCCGTTAGGCCTCCGATAGTGAAGAGGAAGATGAATCCTAGGGCTCATAGCAT
AGGGGGGTCTCATTTAATTGTTCCTCCGTGCAGGGTTGCTAGTCAGCTGAAGACTTTAA
TACCAGTTGGGATGGCGATGATTATTGTGGCTGATGTAAAGTAGGCTCGGGTGTCTACG
TCCATTCCGACTGTGAATATATGGTGGGCTCATACAATGAAGCCAAGGAATCCGATTGA
CAGTATGGCTCAGACTATTCCTATGTATCCGAATGGTTCTTTTTTTCCTGCATAGTATGCT
ACTACGTGGGAAATTATTCCGAAACCTGGGAGGATGAGGATGTAAACTTCGGGGTGAC
CGAAGAATCAGAATAGGTGTTGGTATAGGATTGGGTCTCCTCCTCCAGCTGGGTCGAA
GAATGTGGTGTTAAGGTTGCGGTCGGTAAGTAGTATGGTAATCCCAGCTGCTAGGACGG
GTAAGGAGAGGAGTAGTAGGATGGCAGTAATGAGGACGGATCATACGAATAGGGGTGT
TTGGTATTGTGACAGTGCGGGGGGTTTATGTTGATGATGGTAGTGATAAAGTTGATGG
CTCCTAGAATGGAGGAAACACCTGCTAAGTGTAATGAAAAGATGGCTAGGTCTACTGA
TGCGCCAGCGTGGGCTAGGTTGCCGGCTAAAGGGGGGTAAACTGTCCATCCTGTGCCG
GCCCCAGCTTCTACGGTAGATGAGGCTAGTAGGAGAAGGAAGGAGGGAGGGAGGAGT
CAGAAGCTTATGTTATTTATGCGGGGGAATGCTATGTCTGGGGCACCGATTATAAGCGG
GACTAGTCAGTTTCCGAAGCCACCGATCATGATGGGTATAACTATAAAGAAGATTATGA
CGAAAGCATGGGCTGTGACGATTACATTGTAAATTTGGTCGTCTCCTAAGAGAGTTCCG
GGCTGTCCTAGTTCTGCGCGGATTAGAAGGCTAAGTGCTGTGCCGGCTATGCCCGCCCA
TGTGCCGAAAATTAGGTAAAGAGTGCCAATGTCTTTGTGGTTGGTTGAGAATAATCATC
GGTTG
>c159_g1
AGAGTTCTACATCTTCATCTGAAGACTCCTAGTGGAAGACAGATTGCCAGGCAGCCAG
GACAAGGGCATTAAAGCTATGGTTAGATCCACAAATTTCAGAGCATTGGCCATAGAATA
ACCCTGGTCGGTTTGATGTTACTGTTGCTTGATTTAGTCGGCCTGGGATGGCATCAGTTT
TAAGTCCTAGGGAGGGGACTGCTCATGAGTGGAGGACGTCTTCAGATGAAATTAATATA
CGGATTGGAAGTTCTATTGGCAGAACGACTCGGTTATCAACTTCTAGCAGTCGTAGTTC
ACCAGGTTTTAGGTCGTTTGTTGGGATTATATATGAATCAAAGCATAGGTCTTCATAGTC
AGTATATTCGTAGCTTCAGTATCATTGGTGCCCTATGGTTTTAACGGTTAATACGGGGTT
GTTGATTTCGTCTATTATATATAGAATGCGTAGAGAGGGGAGAGCAATTATGATAAGGAT
TACAGCTGGTAGAATAGTTCAAATGGTTTCAACTTCTTGTGCATCTATTGTGCTTGTATG
TGTTAGTTTTGTTGTTAATATTAGCGAGATGATATAGAGGACTAAGGAGCTAATTAGGAA
AACAATTATTAGTGTGTGATCATGGAAATTTATTAGCTCTTCTATAATAGGGGATGTGGC
GTCTTGTAGACCAAGTTGGAA
>c18027_g1

TGGTTAAGATGACAGTAGTGAGGATCAGGCTTCAAAGTGTTTTAGGGGGGTAGACTCT
ACTACAATGGGTATGTAGCTGTGGTTAGCTCCGCAGATTTCTGAGCATTGTCCGTAAAA
CACTCCTGGTCGAGTGGTGATGAAGGAGGTTTGATTTAGTCGTCCAGGGATTGCGTCT
GTTTTTACCCCGAGGGCGGGTACGGCTCATGAGTGGAGGACGTCATCAGCGGTGATGA
TTACTCGAATGGGGGATTCTATGGGGATTACAATGCGATGGTCGACTTCTAGTAGGCGG
AAGTGGCCTAGGGGGAGGTCTGTTGTTGGGGTTATGTAGGAGTCAAATGAGAGGTCCT
TGAAGTCTGTGTATTCATAGGTTCAGTATCATTGGTGTCCGATGGCTTTTAGGGTGAGGT
CAGGTTCGTCGATTTCGTCTATTATGTAGAGGATTTGGAGGGAGGGGAGGGCAAGCAG
GACTAGGACAATAGCGGGTAGGATGGTTCAGATTAGTTCAACTTCTTGGGCATCTACGG
TGTTTGATGATAGTTTTTCTATAAGTGTAAGAGTTAGAAGGTAGAGTACTAAGCTGCAA
ATTGCTAGTGCGACTATCAGGGCGTGGTCGTGGAATTCAACGAGCTCTTCTATGATGGG
GGATGAGGCGTCTTGAAAGCCTAGTTGGGAGTGGTTGGCCATGTGAGATGTACAGGGT
TTGCACCTGTGATTTAGTCTTGACAGGTCTATGTAATTGGTTTACTAACGTCTCATAAGA
AAG

>c13857_g1

TAATTATACTAAGGGAGTAAGATCCTCATCAATAAATGGAGACGTATAGGAAAAGTCAG
ACTACGTCTACAAAATGTCAGTATCATGCTGCGGCTTCAAATCCGAAGTGATGTTTTGA
TGTGAAGTGAAATTTTAGTTGTCGTAGTAGGCAAACAATAAGGAATGTTGATCCAATAA
TTACATGGAGTCCATGGAATCCAGTAGCCATGAAGAATGTAGAACCATAGATACCATCT
GAAATGGAGAATGATGTTTCAAAGTATTCTGAAGCTTGGAGGATGGTGAAGTAAAGTC
CTAGTATAATGGTAATTAGTAGGGCTTGATTTATGTGGTTTCGTTTACCTTCTATAAGGCT
ATGATGAGCTCATGTAATTGAAACACCTGATGCTAGAAGTACTGAAGTATTAAGTAGTG
GGACTTCTAGAGGGTTAAGTGGTGAAATTCCTGTTGGAGGTCAGCAGCCTCCTAGATC
ATGTGTTGGTACGAGGCTAGAATGATAGAACGCTCAGAAGAATCCTGCAAAGAAAAAT
ACTTCCGAGACGATGAATAGAATTATACCATATCGTAGTCCTTTTTGTACAATAGGAGTG
TGGTGGCCTTGGTAGGTTCCTTCACGAATTACGTCTCGTCATCATTGATATATTGTGAGG
ATATTGGTGAGTAGGCCAAGGGTTAATAGTGTAATTGAATTATAGTGAAATCATATTACT
AGACCTGATGTTAGAAGGAGGGCTGAAAAGGCTCCAGTTAATGGTCATGGACTTGGAT
TAACTATGTGATATGCATGAGTTTGGTGGGTCATTATG

>c10530_g1

AAATATTTAATGGCACACCAAGCACACTCCTACCACATAGTTGACCCAAGCCCATGACC
AATCTTCGGCGCAGCCGCAGCACTACTAACCACCTCTGGCCTAATCATATGGTTCCACT
ACAGCTCGACCACCCTACTGACAATAGGCCTCCTCTCTATGCTTCTAGTCATGCTGCAA
TGATGACGAGACGTAGTCCGAGAAAGCACCTTCCAGGGCCACCACACCCCAACTGTC
CAAAAGGGCCTACGATACGGAATAATCCTTTTCATTACATCAGAGGCCTTCTTCTTCCTA
GGATTCTTCTGAGCCTTCTTCCACTCAAGCCTAGCCCCAACACCAGAACTGGGGGGCC
AATGGCCCCCAACAGGAGTCAAACCCCTAAACCCCCTTGAAGTACCCCTACTAAATAC
AGCAATCCTCCTAGCCTCAGGAGTCACCGTTACATGGGCTCACCACAGCATCACAGAA
GGAAACCGAAAACAAGCCATCCACGCACTAACTCTCACAATCCTCCTAGGATTCTACT
TCACAGCCCTACAAGCAATAGAGTACCATGAAGCCTCCTTCTCAATCGCTGACAGCGT
CTACGGCTCCACCTTCTTCGTCGCTACAGGGTTCCACGGACTACATGTAATCATTGGAT
CATCCTTTTTAACAGTTTGCCTCCTACGACTAATCAAATTCCACTTCACACCAAACCAC
CACTTCGGATTTGAAGCAGCAGCCTGATACTGACACTTCGTAGACATCATCTGACTCTT
CCTCTACATATCCATATACTGATGAGG

>c8987_g1

TGCAGGACCACTTGAATAACGCACTGTATATCTGCCACTATCTAGTTGGTACCCAAAAT
ATGCACTGATTTTTAAAGGTGCAAGTACACTATACAAGCTCCCAAGAGTATGCTCAGTG
CTGACAGGGTGTTCAGACTTGAAGAAGCAATATTATCATCTCAACCCAGCTGTCACAA
CTAAAACAGTTATACAAGCTTGATCCCTAACTCTTCTCCGATGATCATGGCGGCTTTCTC
GCCTATTGTGACTGCCACGGAATACGTGTTAGAGCCAACGTTCGTAGGACAAATTGAC
AAGTCAGCAATTTTGAGACCCTGTACGCCGTAGACGTTCAGACGACAGTCAACAACGC
CACATGTCTCGCGCGGCTTCATGGCGCACGTTCCAAGCGAATGCCACGCTGTCATGATA
TTGACCTTGAGCATCTTGTCGATTGCCTTGTCGTCTTCCGCAGTGTACACGATATCCGGC
GCTTCTATGGCTACGGGCTCAGCCGGGTGGTGAGTAATGGCCGCTGCGCTTCCTTCGG
GGAACGTGGGATGGTTCGGCGCGTACTCGCCCCTGTACGATGCCATGCGGCGGGCGTA
CTCTCGGTTGATCTTGTACGACTGTCTCATAGCAACCAGGTCTTCCTTTCGACTGAACA
CCCCCGTATCGAAGTCGGGCGGGGACTCGACATCGTTCGCGGAGGTGATGTGGACGGA
GCCCAACCCCACAGGGTATTCCAAGTAGCTGAACGTGCTGAAGCACTTGTGCGTCGCC
AGGGGCGGAGTGAATCCGGGTGGAGTGACTCCAACATACAGTGATATTATTCCGTGGT
AGAGGAACGGCTTGTCAGGGGCGTTCGCAAAGAATGACTTCCACAAATTGACGAGAT
CTGGGTCCATTGCTCTCAACTCTTCCTCAGTCGGACGATACTTCGATCCCCCATCTATCG
CATTTGTCGACATCAAGCCTTTATTATCTTTGAGCCATTGTGCAGACCATTTCTCAAACT
CGTCTGGATCGCCCCTGACGATACCATCTATCGTCTCCGCCTCCTCCCTCGCATGGAAC
GGAACGAACAGCAAGTGATGATCGTTGTAGTTCTCTCCCACCCCAGGGAGATCCACGA
GCTGCTTGACGCCATTCCTGTCCAACACCTCTTTCGCACCGATGCCCGACCGCTCGAG
GATCGCAGGCGAACCAAACGTGCCACCAGAGAGGACGACGAGCTTCCTTGCGCGTAC
AATGTGGGGCGAGTTGTCAGCATCAGGCAATATCCGATGATTATGGACATATTCGACTC
CTGATGCTTTGCCGTTCTCGATGATGACGCGTCTCACAGCGCAACCGACGCGAAGATG
TAGATTCTCCGATGTCTTCTGCGTGTTGTAAATATAGCTGTGCGCGACGTCAGATCGAC
GGCCGGTGTTCTGGTCGACCCACTTGGGCCACGATTCGAGAACATTCACATCGTCAAC
CATTCGATTAGCATCGTCGACGACCTTAAACTTCTTGTCATACTTTGCCACTATATCCATA
TAGTCTTCCCCGACATTGGTGCGCATACCGCCATACGACACTTTAAGCGGTCCGTTGTA
TCCATGTGTTGGTCTATCCGGTGCGTTCTGATAAGTCTCGGCCTTCTTGATGAACGGCA
GCAGATCTGCGAAGCCCCATCCGGGATTATCGTACAAGCGCGCCCAATCGTCATAATCC
GACGCAGGCGGCCGCGTATACATCGCGAAATTCACACTCGACCCGCCCCCCACGCACT
GCCCGCACTGTACGGAGAGCGCACGGCCGTCGAGGTGTACACTCGGCCTGCCCACAA
AGTCCTTGATCGTCTTGGACCCCGGCTGAAGGTGCGTCAGGAAGCGCGCGGGCTGGAT
GTGCGCGAGGTTGTCCTGGGTGTGTGGGCCGTTCTCGAGCAAGAGGATATGTAGTGAG
GGATCGGCTGCTGCGAGTCTGCCTGCGACCACGCCCGCCGTCGTGCCGCCTATAAATG
ACCGTTAATGGCGTGCAGGGTTCCACCCTCGAGAGGAGCAGGACGTACCTCCCGCAA
AGATGATATCGTATTCTTCCACTGGAGCAGACATCTATAGAGGTGAAAGATATGGGAAC
CGAAGGATGATTCTGGGATGCGACGGTATTGGTATGCCCTATCAAAGACGTGCTTATAC
TTATATTATGCAGTTTCGTAATCCCGCCCACCGCAAGCCTCACGCCAAGCAAAGTTTGG
CGTGACTTCACCCCTTGAGAGTATCATGTGAGCGGATTCTTCAACGCAGCAGAAGTTC
CAAGGCGGTTAGGAGGAGCAAGTGGAACCGCCGACTGAGCTGAAGAAGGGAACATG
GGACGTAATACCCACCATTCAATCGGGCATGTGAGCTATCGCTGTCTTGTTGGACGCCG
AGAGACGTTGGAGCAGGTGTGGAAGAAGGAAAGGACATAACACTAATGTCCTCTGAC
TTCTCATGATCTCGCTGTAAGCTGTCGGCCTTATGGTAGCATAGAACGAGTCATAGTATG

ACAAGACCATCACCTGCAGGGGGCTATTTGGAGAGCGTCATTCAGCAAAATAGCGAAA
CGGACCCCGATACAGCAAACGCACCTCGTCGGACAGAGCAATTACACACACTCAGACT
CCTGACAACTTTGACATCCATGCTACATTGCGTGACAACAATTCCAAAACTAACACACA
CCTTGTATGTACCGGATATAATGCGCAAATTATGAAACAGAAAAAAAGCGAAACAAAA
CAGTTCTCTGAAAGCAAAGCAGCAGCAGCAGCAGGTCGTCGAGGTCAACAACAGCTG
ACACAGTGGGTAAAGTATCATGACCATAACGGTATGGTATGTAAACCACCATCACTCGA
ACGGATTCGTTCGCCCAGCCTGATAATCCCCACCGCGGTCAGGGCCCTCTCCACACCC
ACACAACCACTCATGCCACCACCGCACAGGCGGCATCTCCTCCCTTTCCGGACTCGCG
CTGCGCGCCCCCGTCGCCAAAGGCTGCGGAACAGCCTCTGGCGGGTGGGCTGTCTCC
CTCTCCACCTTAGCCGACGTGATCTCTCTAGGTGGCGGCAAATCCAACGGTTTGGGCG
GCGCATGCCGCTGCTTCTTCTTCGGCGACGACGACGACGATGCCTGCAGCATTGGGCG
TCTCGACGCCTGTAGATGCCCATTAGCCTTCTCCCTCTCCGTCCCTCCCGAGCCTGAGC
CT

>c8176_g4

TTGTTGGTGGGCTTCTGATAGTTCTCGAGACGCCTCATGAGAGGGACAAGGTCGTTCG
AGGCCCAGCAACAATGATACCCATGGCTACGACGGACCGATCGCCATAAGTAACGGTG
GACAAATCACTCCGGTCGCGCAAGACTTCCTCCGCGCTTCACACGCTATCGGCATCCC
CTACAGCGACGATATCCAGGACCTCAAGACCTCGCACGCTGCCGAGATCTGGGCCAAG
TACATCAACCGTCACACCGGCCGTCGTAGCGATGCCGCGACCGCATACGTCCACAGCG
TCATGGACGTGCAGAACAACCTGTTCCTCCGTTGCAACGCGCGCGTATCGCGCGTTATC
TTCGATGCCAACAACACCGCCGTTGGTGTCGCCTACGTGCCCTCACGCAACCGCACGA
GCGGCGGCAAGGTCACTGAGACTATCGTCAAGGCCCGCAAGATGGTCGTCCTCAGCTC
TGGCACGCTCGGAACTCCTCAGATCCTTGAGCGCTCTGGCGTTGGCAATGCCGAGCTT
CTCAAGAAGCTGGATATCCCCGTTGTCAGCGACCTCCCGGGTGTTGGCGAAGAGTACC
AGGACCACTACACTACCCTCTCCATCTACCGCGTGTCAAACGATACACAGACGCTCGA
CGACTTCTTCCGCGGCGACAAGGAGGTGCAAAAGGAGCTTTTCACGGAGTGGGAGAC
GCGCCCCGAGAAGGCGCGTCTTTCGTCCAACGCCATCGACGCGGGCTTCAAGATCCGC
CCGACCGAGGAGGAGCTCAAGGAGATGGGCCCCGAGTTTAACGAGCTTTGGGACCGC
TACTTCAAGGACAAGCCCGACAAGCCGGTCATGTTCGGCTCCATCGTCGCGGGCGCGT
ACGCGGACCACACGCTCCTCCCGCCTGGCAAGTACATTACGATGTTCCAGTACCTTGA
GTACCCGGCGTCGCGTGGCAAGATCCACATCAAGTCTGCCAGCCCCTACGTTGAACCG
TTCTTTGACAGCGGCTTCATGAACAACAAGGCCGACTTTGCTCCCATCCGCTGGAGTT
ACAAGAAGACGCGCGAGGTCGCGCGCCGCATGGATGCCTTCCGTGGGGAACTCACCT
CGCACCACCCGCGCTTCCACCCCAACTCGCCCGCGGTGTGCCACGACCTCGACATCAA
CACCGCGAAGGAGCTGTACCCGGACGGCCTGACTGTCGGTATCCACATGGGCTCATGG
CACCGCCCCGGTGATGCGTACGACGCGTCCAAGGTGCACGAGGATCTCAAGTATACTG
AAGAGGACGACCAAGCTATCGACGACTGGATCGCGGACCATGTCGAGACGACCTGGC
ACTCGCTCGGCACTTGCGCGATGAAGCCGCGCGAGGAGGGCGGTGTCCTCGATAAGC
GCCTCAACGTCTACGGCACCCAGAACCTCAAGTGCGTCGACCTCAGTATCTGCCCGGA
TAATCTGGGCACGAACACGTACTCGTCTGCGCTGCTTGTCGGTGAGAAGGGTGCCGAC
CTCATCGCTGAGGAGCTGGGGCTTAAGCTGCGGATCCCTCACGCGCCTGTCCCGCACG
CCCCGGTCCCGACTGGTAAGCCCGCGACTCAGCCTGCCCGGTAATGGGAACAAGGAA
GCACCGCGATGTTGTGCTTTTGGACTTTCGAGCATTCTGGAGACATTGTGTTCTCGTTA
TAAAGAGTCGGAGGAATGGGGATGTTAATATCCTATTCGCTTTTTTTTGTGCCAACACG

TGTATTACTTATGTTTGTATGTAGGAGTACTGTAGCAGCATCTGGCGAGAGCTCCGCAG
AAAATGACATTTTCATTTTCGTTGTTCAGAATTAACCTGTCGTACCAGGTGGACTGATA
>c8020_g1
GTGTCAGCAACTCTCAGTTTCAACAATATGGCTAGAGGTAAGTAGTCGACAAGTCCGG
ATAAGCCATGCATCAAAATTTTCCCACCGTACGCTCACACAAAGTTGAGGTTGACGGT
GACGCCGAGGTCCTCGGCAATGAGCGCTGCTGCCTTTTCCCCAATCATCATCGCCGTCG
AGTACGTATTCGTTCCTACGTTCGACGGACAGATGGACATATCTGCCACTTTGAGGCCA
TGCACACCGTACACGTTCAGGCGGGAGTCGACCACACCGCCTTTATCGCGTGCCTTCA
TACTACACGTGCCCATCGAGTGCCAGACCGATAGAACTAGAAACTTAAGTCAGTAAGC
CCACACTTGCACATGATGCCCCAGGACGACTTAATGTTCTCCTTGAGCAGCGCGTCAAT
GGCCGCATCATCCTCTGGGGTGTACACAATGTCCGGTGCATCTATGCTCGCAGGGCCAC
CATCCTCTCGGGCAGCAACTGCGACGCTGCCCGCGGGGTAATTGGGGCGGTCGGGGA
CGTACTCGCCCATACACGCTGACATGCGTCTTCCGTACTCCCGGCAGAGTTTATATGCAT
ACCGCATCGCGCTGATATCCTCAGATCGACTAAATATTCCATGATCAAAGTCGGCAGGA
GCGTCAACGTCGTCCGCTGACGTGATATGCCCGTAACCCACACCGGCAGGGTACATCA
AATATGCTCCAATACAATAGCATTTGTGGGATGAGGCAGGCGGCTTGAAGCCTAGATAC
TGCGATAGTATCCCAACCCAGAACATAGATTTATCTGGCCTGTCGGAAAACAATGAGGT
CCATCGCTCCACAAGTTCGGGATCCATGCGACCGACTTCCTCGTCTGACGGCCGATATT
TAATGCCACCGTCCACGCCGTTGCTAGAGAGCAGGCCGGTGCCATCTTTGAGCCATTG
CCCCGACCATTCTTTTTTTTTTGTCGTCACTCAGCATCGCAGCAGAATTGCAGTACCGA
CGGAGGTTCACGTACTCTCCATCTCGGTCGGGTCGCCTCTGACGAGTCCGTCAAAGGT
TTCGGAGTTTTCTTTGGCGTTAAAAGGTACGACCGTCAATATATGGTCTATGAACTCGC
GAGGTGTCAAGCAAATTCATCGGCATAGCTCGACGAGACATTTCGCACCCATGTAGTTC
TCTCCGACCCCAGGGAGGTCGACAATCTGCTTGATACCATTTTTGTCTAATACGTCCTTC
GCCCCAATCCCCGACCTTTCAAGTATGGCAGGCGAGCCGAATGCACCCGCAGAGACTA
CGACCAACCGCTTGGCGCGAATGAGCTTCACCGTATTGCGGTTCTTCTTGTCGATGTAT
TCGATACCAGTGGCTCTGCCGTTCTCAATAATGACACGGCGAACAGAGCACTCGACCA
GTAAATGCGCATTTTGAGACTTTTCGAGCATGGGATATATGAAATGGTGTGCGACGTCC
GAGCGACGTCCGGTATCCTGATCGATCCACTTCGGCCAAAATTCAATACGGTTAACGTC
GTCGACCATACTATTGGCGTCGTCGACCACGACGCGATCCTTGTCGTAATGGGCCACAG
TCGTCACGTAGTCCATGCCGACGTTCGTGCGGTAGCCACCGTACGAGACCTTGATCGG
ACCACTGTAACCGTGCGTGGGCCTGTTTGGGGCGTTCTGGTACGTCTCCAAATTCTTGA
TGAACGGCAGAAGGTCGGCGAAGCTCCAACCGGGATTGTCGTGCAAATAAGCCCAAT
CGTCATAATCCGAGGCAGGCGGACGAGTGTACATGGCGAAATTGACGCTTGACGCGCC
CCCCACGCACTGTCCGCACTGTACGACAAGCTGACGACTGTCGAGGTGCTCGCTCGGC
TTTCCCACGACGTGCTTGACGGTCTTCGATTCAGAGTAGAGGTGTGTCATGAATCGGCC
GACTTGGATGTGTGCAGGGTCGTCTTTGGTGTGTGGGCCGGTCTCGACGATGAGGATC
CGCAACGACGCGTCAGCCGCAGCGAGCCTTCCGGCGGCGACTCCCGCTGCTGTACCA
CCGCCAACGAAAATGATGTCGTACTCGTGGAACAACGGCGGGGCAGTTGTTTCTTCTA
GCCGAACCATCGTTTTGTCCAAATTGTCCATAGTGTCAGTTATTTGTTGAGGAAATCTC
ACTGCAAGAAGGTGGAAGCAGCGTCGAGGACTCAGGAGAGGGGGTGCTCTTCCTGCA
CAACCGTCGGTGAAGTAACTGAAGCAGCGAGAGTCTGTGTAGGGACCACGGCAGTGG
GGTTGGCTATATGGTTTTTGGTGTCCAGAGGGGGCTGTTCTTGGAAATTATCGTTCAGT
CCCATACGACACAAGACATCCGGTGTGATTGGCACGGGATACTACTAGTATGTAATACC

GGAAGCTGAGCCGACTCAATGGTTGGTAGAGAGCCTGCACCATGTCTTGGACAGGAA
GTTCGACGTCAGACTTGGTAGTCACCGGTCACATTGCCGTACTGTAGAACATTTTGAAG
GACCATGCCGAAACGTAAATGCAGCTATGACATTGTAAGAGCCTAGAGGGCTCAACCT
ACATCTGGGCGACGAACAGCAACCCAGAAGCCACGTTTGGCAAATCCGAGTCCACGC
CATGGCACAGCTCTGAGCCTTCGATTTCTGATCCTGCGTCGCCGACTCTCACGAGACC
GTGCTCATGTCGAATGGACGAAGCATCGTGGCTGGTGTTGCTGCATTGTCAAGTGAGC
AGGCCGTGAATGTAGGCAGAGTATCCTGCATTCACTAAGGAGCCCTTTAGCGTGATTAT
TGGCTGAAATCACTCGCTTGCACTGCGGATGTGGTGATTG

>c9143_g1

AGGTGAGTCTTCCCTCTTAGTCCAGTTGGATCATGATTATACATGTCGTGGTGGCGTCGT
TGTGACGTGCCGAAAACTACCCCTTGTAACCCATCTCCATCCTGGAGGGAGGTTTATGT
GTCAGGTGGAAAGTGACGACACGACAAGGCCAGATATGAACTCCCCTTCATTTGTGCC
GGCACTATGATGCAACGCTTACATTAATTCTTGGGGTCCAGATAAACAACTTTCCGGCT
GCCTTTCAGGGAGACTGTAAAAGACAAATTTTGAAACTGTGTTATAGGTATACTCTTCT
AAGGTCTACTGCACCCGTAATTCTCTTACAGATAGGGCACAATTTCGTCGATCCAAGAC
AGCGTAGCCAGCATTCGCGACAGCATGTATGCCAACAACCGGTTGAAACGGTGGGTTC
AGTATAAGGGTCCAAGCAGATTCGACACAGCAACGCTGTCGAAGATGATACCTTGGCC
GATACCAGAAGCCGTACTTTACTCTCCAGAGCGCGCATCAATGCAGCTTGGTCGCCTG
ATCTGTGCGCGAGTTCTATAGCAAGATTGAATGTCTCCGTGCCTTCCAAGCCAATTGCC
TCGTTGACAACCTTCCCCTTCACGACAAGGTCTCGCAGAGTGGTCCCCTTCTCCAGGT
CTCTCTCTGGGACACTCGTCGATGTACTGCCTGAAGCTCCCGCATCCTCAACATCTACG
TCGGCATCTGTGCCCGCGTCTGATTGATCCTCGGAAAGGAGTATGCTCGGCACCAGAA
CGTCCCGTTCTGTGAACTGGGCCGCACCGAATACCACCTCGTCCTCGCCATCGATATCT
ATGTCATCGTCAACGTCCTGGTCATTCCGATTACGACGGTCAAACCCTGTTCCTTGAAA
ACTAACGCCATCCATGACATCCACTATTCCGTCCCCTCCATCCCCATCAATATCCACGTC
TCCGTCCACGCCCACGTCCACGCTTGTTCTGCGCGCCCTCCTTCCCTCATCATCCTGCT
CGTCTCTGAGCCTCAAATTCGCCAAGCACGAATCGACATGCGCCTCTATTACATCCCCG
TCCCCTGCGATGAACTTGGCGCATACCGGACATTTCAAGCCCTCTCCACTCGCTGCATC
CACATGCCTCCTCCCTCCCCACCACATACCATCCTCGTCCTCACGTGTTAACTCACGCA
GCTTGGCACACCGTTGCCGCCTATGCCGTTTGAGCAAGCGCAGCGTCTTCGTCGTCTG
GTCCAGTACCGGCACAGACTTGGAGGACGAGCCCGGCCTGAGATGCTGCCGCGCCTT
CAGAGCCGAACGTCGTGTTCGCGCCGAGCTGCAATGTAAAGCACACACGAAGGTAAG
TCAGACGCTCAGCGTCCTCGGTGTATCTAGTACGGGTGTGTTCACGCACCCGTCATCCG
GATCCGCCTCCTCATGTACTTCTGTAGACCCAATGCACCGTACGATCTCCTCCAGCCTTT
CAGACTCCCAAACGGCGTGCTTCCCGAGAAGTCTTATTGGGATAGACTCATTGCACAC
AGGGCATTTGCGCGTTTCTGCTCGCTTAGCTGCAATATGAATATGTACGAAGAGATTATG
TCCACATGTCAGTCACTGCGACTTTCGCTCTATACCAAATATCAAGGAGAGCGTACATT
TCTTCGCAGGCACGGCTGGCTGCTTCTCCTGAGCACGAGTGGACAATGGAGTTGCCAG
TGTCATGGAAGATGATGCCTCACTCAACGGCTCTTCTTCGCTGAGCAGATCCTGGACC
GCGCGCTTCTTCCCCTTGGAGAGACGACGATTTGTCATAGCACCAGGGAAAGTCGTAT
AATCTAGTCTGAGGATATTTCAGAACTCCAAGAAGCAGAATGCTATGTGCTAGGGAGTA
ATGAACAGGGTGGATTGAAAGTAACCAGCCTGGATGTCAGAACAGAACACGAGGAGA
ATTGGACGCGTGTGTCATGTGATCATGACGTAGCGCCGTTAGCGTAATTTGTAATTTGAC
ACTTGCTTGCAACAATATTTATACGACATGCGTTAAAGTGATACAACCATACAACCACA

ACCGGTTCCGGCATGTACATTCGAGATTTAGTAGCGACTTTGCGGGATCATACAGCTA
AGCGAGGACAGGTGTACCACGACGCGATTTCAGCAAACAACTCGCGACAACAGCACG
CGACCATCTCACAGCTGGAACTTGCCCTTTACGATATCAACGGCTTGTTCAGCAATGGC
GTACACAGTCGCTTGAGGGTGAGCGGCGACGTGCAGAGGGAGTACCGAGAGGTCGGC
AACCCTGAGGTTCTTCGTACCATATACCTTCAATTCAGGATCAACCACGCCCCCGTTCG
CTTTTGGCAACATTGAGCAAGAGCCAGCGGTATGCCATGTCGTCGTCATGGTCTGCTTC
ATCCAGTCGATGATCTGTTCATCCGTCTGAACATCAGGACCAGGGTTCAATTCTTTGGT
GGTTATATCCTTGAGTGGAGAGATGTTGCGCAAATTCCTCGTGAACTTGACCATCTCAA
GCCAAACCTGAAGGTCGACTTCCTCGTCAAAGTAACGAGGGTCAAATGCTGGGTCCA
CTGAAGCGTCTTTAGACGTGGAGTGGACAGTACCCCTGGAGAAGGCGTAGTTCAAGG
CCACGTAGAAAGAAGCATAGCGTTTGCCCTTCACAGGGGGGTTGGGACGCGAGTTCA
AGCCCGGGAAGGAAATGAATTCACAGCCGTTACCGTTCTTCAGGCGTTCGAGCAATAT
CTTGTACTGTTCGAGCACACCGGGAGGGTAGTCGTTGGCATTCTTCTTGATCTTCTCTT
CGGCTGCTTTGATGATCTCGTTCGCTCTTGGGCTGATTTCCGCCAGTGTCGCGAAAGCG
AATCCAACAATCCCCGTCGTGAAAAGGCCAGTGCCGCCAGAGTGAAATTCAATGTGCT
TCGCAAGCACCTCGGGATCGCTCAAAAGATCCAAAGTATCGAACTTCACATCGTCCTTA
ACCTCGAAGCTGACGGCAACAATCATGTGCTCCTGCACATTTTTCCCGACGCCGGGAA
GATCGACTAGGGTCTGAATTCCAAGGTTTCTGAGAATGTTAGGGTTTCCGATACCAGAT
AACTCGAGGATCTGTGGCGTCTTCAATGTACCAGCCGTGAGTATGACCTCTTTCTTTGC
TTGTGCAACGTGGATCTGACTACCGTGTTCGAACTCAACGCTATCAGCCACAAACTTG
GAATCGCTGGCAGAGGACGGGATGACACGTTGGACAAGGGCAGCTACCAGTACTGTC
AAGTTCGGGCGGTCTTTGTTCGGGGTATAGAAAGCCGTCGTGGAGTAAGTTCGAGTGT
GCGTCTCTGGGTCATACGTGATTGGGGCGAAGTAAACACCGTGAGTCTGCCCATTTGA
AGGTCTCGGTGCAGCGGAAAGTCCGGCATTCATGAGCGTCTCTTGGACTTTCATCTCG
GCGTCCTCGATTGTAGGAGGATAAGTGATAACCAGTGGGCCGTCTGTTCCTAGATTCCA
CGTATCGACCTTGAGATTCTTGGCCAAGCGAATCTCCTCCGTGGGGGGTATGAATCCCT
CAGTGCGTTGAACGTATTTCTCATAGTTCTGCCAATTCCACCCAGGATTTCCAAGACGT
TCAAGATCGTCAATGTCCGAGGCGGGAGGCTTTATCCAGCACAAGAAGTTGATGCCGG
AACTACCGCCGAGGCCTTTGCCTCTCTCCCACTTGTGCGTGGTGTCACCTGTCAACTTC
TGCTTGATCGTGGTGTGGTCCCATGAATAAGCCGGATTGCCAAAGTGACTACCGTACGA
CGCTGAGCGGAGGATGCCCATGTCGTCGAGGTTGGCGTTCCCAGCTTCTAAAACTAGA
ACGGTGGTGTTGGGATCCTCGGAGAGTCGGGCGGCAATAGTAAGACCGGCAGTACCA
CCACCGCAGACGATGTAGTCAAAGGACTTGTCCTTGACGTCGTCGATAGTCGCAGCCA
TTGTGAGCGTAAGAGGAGATAGGTCGTTTTGTGAAGCGGTGAGATAAATGTACTAGAG
GGAGGCCAGGATGGGTGACAACGGGGGAGCGGGGGTTTATATAGATATGTTGTTGGGG
GGCGCAGCGTGTGCATGTCCTATGTGCAATCCGGATATGGACATGCTTCTGATTCTGGAT
AAGTTGATGGGCTGTCGCTGCTGGACAGTGGAGAGAGCTTTGCCAGGGCATTGACATT
CCTCGCAATTCGCCAGCCCACTGTTCGGCAGAGTAAGCGATAGATAAGAAGCGTAAGC
GGCGTTACAATACTACAGCGCGCTCAGCCGCGGCCAACTCTAACAGTTGTGCAGCAGG
TTCAGATCGCAGGGCCTCTCGTTCACGCGCACGGACAGTGACAAACGGTTCTATTTTC
TCTTTCTCATCCCGCGATAGTGGCAGGCCAAGTTGAAAAGACCGCTGGGGACGAGCCC
GATCAACGTATGACTGGAGAAAACCCTCCGCTATCGCTCTTCGGACAAGTTACTATGGC
GATATACGCAAACAGGCAATTTAGGGATCCCGGTCCTAGGGCCGGAGATATACGATCCT

GATGCCCTATAGTCTCCGTGACTGTTGTCACCCACGCACCGTACACAGAGACGCTGGC
CGAGCTCTCAAAGAATACGACTGACTGTTCAACAAATCCATGAAGATGGCGTGGGCAC
AGCCATCTCGTACAGGCGTTTCCAGCAACTCCGAATGCCGGACCACCGTGGAGGCCTT
GACATGAAATCAGTAGGCGGGTCTGTTATCAGAACATCACAGCTATGCCGTGCCCTCAA
CCACACGCCTTCGGATCGTTTCGAAGGGTGCGTCTGGGAAGAAACGTCGCCATTGCAA
TGCGTGCGGACGCATGCAACACATAGCACACGTGCTCTTCCACTACCCTGGAGAATCG
CCGCCGGTGGAGGGTGGTGGAGGTACGATGGAGAATCACAAATCTTGCGCGCTCAAA
CAGGCTACTACAGTTTGTGCAGCTTTCTGGACAAAAATAGGACCGCACTCCCGTTCGC
ACACGCTCCTTACGACTTACTCATGTGCGATCGCAACAAATCGAAACAGTCACCCAGA
GGCCTAGAG

>c17518_g1

GACTTAAAACCTTGTTCCCAGAGGTTCAAATCCTCTCCCTAATAGTGTTCTTTATTAATA
TCCTAACACTCCTCGTCCCCATTCTAATCGCCATAGCCTTCCTAACATTAGTAGAACGCA
AAATCTTAGGGTACATACAACTACGAAAAGGCCCTAACATTGTTGGTCCATACGGCATT
TTACAACCATTTGCAGACGCCATAAAATTATTTATAAAAGAACCAATACGCCCTTTAACA
ACCTCTATATCCTTATTTATTATTGCACCTACCCTATCACTCACACTAGCATTAAGTCTATG
AGTTCCCCTACCAATACCACACCCATTAATTAATTTAAACCTAGGGATTTTATTTATTTTA
GCAACATCTAGCCTATCAGTTTACTCCATTCTATGATCAGGATGAGCCTCAAACTCCAA
ATACTCACTATTCGGAGCTTTACGAGCCGTAGCCCAAACAATTTCATATGAAGTAACCA
TAGCTATTATCCTTTTATCAGTTCTATTAATAAATGGATCCTACTCTCTACAAACACTTATT
ACAACCCAAGAACACATATGATTACTTCTGCCAGCCTGACCCATAGCCATAATATGATTT
ATCTCAACCCTAGCAGAAACAAACCGGGCCCCCTTCGACCTGACAGAAGGAGAATCA
GAATTAGTATCAGGGTTTAACGTAGAATACGCAGCCGGCCCATTCGCGTTATTCTTTATA
GCAGAGTACACTAACATTATTCTAATAAACGCCCTAACAACTATTATCTTCCTAGGACCC
CTATACTATATCAATTTACCAGAACTCTACTCAACTAACTTCATAATAGAAGCTCTACTAC
TATCATCAACATTCCTATGGATCCGAGCATCTTATCCACGCTTCCGTTACGATCAACTTAT
ACATCTTCTATGAAAAAACTTTCTACCCCTAACACTAGCATTATGTATGTGACATATTTCT
TTACCAATTTTTACAGCGGGAGTACCACCATACATATAGAAATATGTCTGATAAAAGAAT
TACTTTGATAGAGTAAATTATAGAGGTTCAAGCCCTCTTATTTCTAGGACAATAGGAATT
GAACCTACACTTAAGAATTCAAAATTCTCCGTGCTACCTAAACACCTTATCCT

>c1946_g1

TTTTGATCTCTTCTGTGTAGGTTCGATTCCTACTTTTCTAGGATTAAGGAAGTGAGAGGG
TTGTTATACCTCTATGTTCACTTTATCATAGTGATCCTTTTTGTTCAGGCACGCTTCCTTA
GATTGGGGGGAGGCCGGCGTAGCTGATTGGTATGCTGGTATGTCAGAGGCATAAGGCT
AGGGTTAGGGGGAGGAAGTTTTTTCATAGAAGATGTATTAGTTGGTCATAGCGGAACC
GTGGATATGAGGCCCGGATTCATAGAAATGAGGATGAAAGGAGGAGGGTTTTTGTAGC
GAGTGCAATGGGAAATAATTCTGGTGGGAGATTTAGGAAGCTTGGGTTCAGGAATAGG
ACGGTGGTTAGTGTGTTTATTAGTATAATGTTGGCGTATTCGGCTAAGAAGAATATGGCG
AATGGTCCGGCGGCATATTCTACATTAAATCCTGAGACTAGCTCTGACTCTCCTTCTGTT
AGGTCAAATGGGGCGCGGTTGGTTTCAGCAAGGGTAGAGATGTATCATATTATTGCGAG
GGGTCATGCGGAAAAAATGAGGTAGATGGGTTCTTGGGTGATGGCCAGGGTGCTTAAG
GTGTAATTGCCGCTCAGTATGATTGTGGATAACAGGATGATGGCTAGGGTGACTTCGTA
TGAGATTGTTTGTGCGACGGCTCGAAGGGCTCCGATTAGGGCATACTTGGAGTTTGATG
CTCATCCGGATCAGAGTAGAGAGTAGACAGTTAGGCTTGATATGGCTAGGAGAAATAGT

AGTCCTAGGTTGAGGTCTGCAAGGGGGAATGGTAGTGGGAGGGGGACTCAAATAGTC
AGGGCTAGGAGTAGTGCTAGGATTGGAGTAATAATGAAGAGGAAGGGGGAGGAGGT
AGATGGTCGGATTGGCTCTTTAATGAATAGTTTTACCCCGTCTGCAACGGGTTGGAGTA
GACCAAAAGGGCCCACAATGTTTGGGCCCTTTCGGGCCTGCATGTAGCTTAGGATTTTT
CGTTCTACAAGTGTTAAGAAGGCCACGGCGATTAGGATGGGGAGAATATAGGATAAGG
TTATGATTAGAAGGTTTGTTAGGGTGGGCAGGGTCA

>c4703_g1

TTGTTAGGTTCGTGTTCTGCTGCTTTTGGAAATAGCATGCATTCATTGGAGATTGACAA
ATAAACCGCCATCTACAAGTAATTGCGCTCCTGTCACGTACTTTGCTAGGTCGCTTGCTA
GAAATACTACTGGTCCCGCAACGTCGTCGGGTGCTCCAAGTCGCCCCAATGCGGTGCG
CTTGATCATGCCTTCTCGTTTGGCCATGTCCGAGAGGTCCACCTTGTTGATGTCCGTTTC
GATTGTGCCCGGGAGGATCGCGTTCGCGCGAATGTTGTACTTGCCAAGAGCGACTGCG
CAACTTTGCATCAGCGAGAGGATCCCAGCTTTTGTCGGTGTATAATGTACTTGGTACTC
TCCCCCAACGAGAGCGGATATGGACGAGATGCCAATGATGGAGCCGCCCTGCGGCACC
TGGCTCTTCATCTGGTTTGCAACAGCTCATGCGGTGGTTCAAGGTATGCAACAGGTGG
ATGATAAGGACTGTTGTGACTTACCTTGTGTCACATAGAACGACCCGTCGAGGTTCACT
TGTCGTGTCCGCTCCCATGTTTCCACGGGCATAGTCAAGAATTCTGCGAAGGGGCAAA
TGCCTGCATTGCTGACGAGTACATCTGGCGGTCACAAACACAGGTTGAGTTTGTTTCG
CAATGCAAGAACGGGTGTCCTCGCTACCTATGCGCTGGAACGCGAGCACGCCTTGTTC
GACGATCTAGGATGTATGTGAGTGTTGTTTCACGGTTGCACGGGGCATTACCTTTTGAG
ACGTCTCGCGCTGTGCGATGTCTCCGTCAACAAGGACTATCTTGGCGTTGTACCCGGA
GGCTTCAATCTCGTCCTTCAGCGACGTCACACCCTTAGTGCTCTCCATGTCCCCGAAGA
AATGGATAATCAAGCCCCTCGCGCCCTGTTTCGCGCATGCCAGAGCACAGGCTCGTCC
AATCCCTCTGGAAGCCCCAGTGATGCAGACGACCTTGTTTGCGAGGAGGGACATCTTC
CTGGCGCAAAGGCGGAAATCGGGATCCGACCACCCTAGGAGCGTCGCGGTGCTTTTTG
TACTGTCCTAATCAACCTCCTCCGATCACAACTAGAGCAGCGACGGTATCGGTCATACG
TCTGCAACCCGGGCCGCTGCGACGGGCGTCCCTGCGTCACAGGGACTATTGAACACAC
CCGATGATTTGGTGAGGGGCTTCTGGCGGAGACCCGAGGCCAGAACCGGCAGAACCA
GCCATACTGAACATATATAA

>c8739_g2

GCGGGAACACAAATGTCAGTGACTACCGCCGGAATGTTCGTGCAGTTGTCAGACGTAT
CTCACCGTGTTCAATGCCATCGGTGGTGTGGTTCCCGCCTTCGGAGACTATCGATCTTC
CTTGCCTCTCCCCCTGTCGGTCTGTATTCCTTAATACTGGTTTGATTGGTCACCAACGTT
CGAGGCTGCGAAATGCGGTTCTGGTCCCTGGAAGTCGACATCGGGAGTAGCCATGATG
ATGGAGCATCGGAACCGTCCTAACGTTGTAGACATAGTGCTCGTATTCATCGCGCTATG
GATGTCACATGTGTATGACTGTGACATATCAAGCTTGTCTCATTGCTAACGCTCGAATGC
CTCCTGTTGCGGCGATAGTACAAGGTGAGCGCCTATTGCGATTTTCGAATCTCTTTATCG
GCCTCGCAGGAACGTGGATGGAGCCATATCCCGGGATCACGCATGCATTGTCACTGTG
ATCAGACTGCCCTGCGGCCGCGTGGGCCACTATGGCATCGGTGATAGGCGAAGGTTTG
GCTATTCGCCTATGCAGAGATGTATGGGTCCGATGTCGGCGTTGCCACACGAGTGAAAT
CAACATCAACCATAGAATCTGCATACTGAAGCGCAGGCGCGGTGATTGCGTTTTCGTCT
AGTTTTGTGTTCGTTTTGCATCTTTGAGCATTCGAACATCTGGACATGCACACTATAGAG
TGCGGCTCGCCTGGCGTGCAACGGCCGCCCAGCAAGGTTCTCTCGAAGAGACGTATG
AACGATTTACTGTGGCCGTCGTGACTGCCGTTTCCATGTGGGCAGATTTCTAGCTAACA

TTATGGTCCACGTGCTTGCTCCCGCCGTTGCTATTGATCGTCGGGTCCCTTGACCCGGA
ATTGACCGTTCTTCAGACGGGTCGTGCTTCTGTGGGATCGTGTTGCAAGTCATTGATGT
CTGACTTGACCTCGTACCACGGCAATTGTTCCCTATCTTGTCAAGATGATGCGTATCCTC
GCAAGTTGCCGGTCCCACGGGGTGGTTGGAGCTTGCATATCAGAAAGTGCCTATGTCC
GTCTTTCAAGTACGTCAACGCACACAGATCCCAAATTTCTCCCCTCTTCGTCGGTGTTA
TTTATCGGTGCTAGAACATCATCGACCTCAGGGCGGTTTTATGGTTTTACGTGAAGGAT
GCGGCCTCATTGAAGTTGAGGTAACTTGAATGAAAAACAGCAAGCCGAAGGAACCGA
CGGTGTCTGTAAACCAACGTATCCAGTATGCAAGACACTAGCTTGTGCCCTTGGCCCAT
TCAGCTCTAAAGGATGAAATTACAGCATTGTTACTAGCGATCCCTATCGCCGGGACCCT
CGAGAGACTTTTCAGGCGTACGTGCGGTCTATTAAGCTCGGCGTTTAAACGCGAAATG
AAAACGAGCGTTTGTTCCACGATACACTCAGGTTCGCCGTTGACAGATCGAGGCGTTC
CGGGACATGTTCCCGTGCTCTAGCTCGCGCGGGCCAACCGCCCATCCGATACTCAACG
TCTTCCTCCGGTGCACATCGCCGTGCTGCGAGTCTTGAGTTAAGCTCGATAACAGCTCT
ATACCACCATGCATGATGGCAGAATTTGACTTGTGACTAAATTGTAGGCGTTCCGACGG
CCGTCCGCCCCCCCAAATGCAGAATGCGTCTCGACAGCACGCACGGAACCTTCTGCGC
CCCATTCATTCGGCACATCGGACGTGTGCACTCAGGCTCTGGAGACGCGGAGTGGGA
GCAGCGTGCGCTGACGGGAATTATGCGTGAATAGTCTCCGAGCGAAGGCCTGGGGTGC
GTCAGAGTCGCAGGACTGTTCTCGAGTTCTATTCAGCCTCGTTTTCCATGTCAAATCTT
CCAGCGCGTATCGCCCGCGTTCGCCCCTGGCATTGTGTCGCGCTTCCACTGGCAGGGA
CCGCGCCTCGCACAACCTACGCTGGGAGACGGACTGCGCGCGCGCTCGCCTGATTATG
GATGGATGGATTTGGATGGATGGCGCGTAAGTCAACTTGTATTTCGTTGTTCAAGAAGA
AGGGAGATGAAAAGGAAATCTCCCCTTGTTTCTCTCAACTTTGCCGTCGGAAATGTTG
CGTGCTGACCATGCGAAGCACACCAGGGACAGCATGCGGACCGGCTCGCCTTCATGTT
GTACTCGTCTCCCTGTTCTTTCGTGATCGGTCTTTAAGACCACTACGTATTGGCATGCAC
CTTCGTGGTCCTCATTCACGCGGGAAGCCAGCCGTAAAGGGGCACGGTCGAGTCGTTC
GTCGGACATCCCCTGGGCATGCGATACATCCCGAATGACAACACGCTGTCATTGTCATT
TCGCCCGAAAACGAGGGCAATACGTACATGGTAACGCAAGTGCCATCGCGCATCGCGG
TACGGTTGCGCCCCCTCATGAGGTGGGCGCCGGCATTCACACAACTGGCAGGGGTTTT
TCCATGGCGCGGAGGTCTGGCCGCAACTAGGTCGGCGTAGACTATGTCCTTCGCGCTG
CTTCGCGGACTTCTCCCTCGCGCGGGAGCATTAGCGCGGCACATCTGTCACACTCTCA
CACTCTCACACTCTCACTCACTCACTCACTCACTCA

>c2043_g1

GAGCGAGTCGAGCGGTTGTCTGGTCGCGTCTCGGAAACCCGTAGCCCTTGCAGCATGG
CTGACCAACTGACTGAAGAGCAGATTGCAGAGTTCAAAGAAGCTTTCTCACTATTTGA
CAAGGACGGGGATGGGACAATAACAACAAAGGAGCTGGGGACAGTGATGAGGTCCCT
GGGGCAGAACCCCACAGAAGCAGAACTGCAGGACATGATCAATGAAGTAGATGCGGA
TGGTAATGGCACAATTGATTTCCCTGAATTTCTGACAATGATGGCAAGAAAAATGAAAG
ACACAGACAGTGAAGAAGAGATTAGAGAAGCATTCCGTGTGTTTGATAAGGATGGCAA
TGGCTATATTAGTGCAGCAGAGCTTCGCCATGTGATGACAAACCTTGGCGAGAAGTTAA
CAGATGAAGAGGTCGATGAGATGATCAGGGAAGCAGACATTGACGGGGACGGTCAGG
TAAACTACGAAGAGTTTGTACAAATGATGACAGCGAAGTGAAGACATTGTACAGAATG
TGTTAAATTTCTTGTACAAAATTGTTTATTTGCCTTTTCTTTGTTTGTAACTTATCTGTAA
AAGGTTCCCCCCCCCTACTGTCAAAAAATATGCATGTATAGTAATTAGGACTTCATTCCT
CCATGTTTTCTTCCCTTATCTTACTGTCATTGTCCTTAAACCTTATTTAGAAAATTGATG

AAGTAACATGTTGCATGTGGCTTACTCTGGCTATATCTAAGCCCGTCTGCACATCTAAAC
TTAGATGGAGTTGGTCCAATGAGGGAACATCTGGGTTATGCCTTTTTTAAAGTAGCTTT
TAGGAACTGTCAGCATGTTGTTGTTGAAGTGTGGAGCTGTAACTCTGCGTGGACTGTG
GACAGTCAACAATATGTACTTAAAAGTTGCACTATTGCAAAACGGGTGTTTTATCCAGG
TACTCGGACACTATTTTTTTGTACTGCTGGTATTGTACCAGGAACATTTTCTTTTATTGTT
ACTTGCTTTTTAAACTTTGTTTAGCCACTTAAAGAAAACCTGCTCATGGCACAACTTGC
CTCAAATCCATTCCAAGTTGTATATTTGTTTTCCAATAAAAAAAATTACAATTTAC
>c3257_g1
GCAGCACAGCCAAGACATCCTAACTGTACGCTGGAGACGCCTTCTCTCACCAGGGCTC
TAGTCTGAACATCACCTGACTGTGATCTGCGCATGTGCACACAACACAGAAAATAACA
AGACTTACGTGGTCATAGACGGCAAAGAACTCCGAGTCTGTCTGGTTTCAGGAGTTGT
GGCTAGAGAACCTGGTGGGAAATCCAGCTTGCACTGGATGTTGCGTGTCTCTGTGTAT
GCTGACCTCCCCTCCAGCGGCCTTGGCCTTGTCCCCCTTCCAATCTCAGCTTTACAAAG
CATTTAACACCACTTTTAAGTTAATGGTCTTTTTATTGGAAAAAAAAAAATAAAAGACT
AGCATTTACAACTTGGAATGGACGTAAATTATCATTTCCTGAACAGCAATGTTGATGGT
GTGCTCAAGTCCACAGACACAGCCTACTCTGCTGGCTCCGAGTCATGGTTCACTAGGT
TTTAAAACACAGTCCAAAAATGCTCCCTTAGGAAACATTTCTTAATAAAATGTGTGGGA
TTTGGTGACAGCCTTGTTGTTCAGTCACTCCAATGCAATGCGCACAGCAATGAACCATC
ACACTCGGGCTCGCCACCAGGGTGTCACTGTCACCATGGGACTGGGGCACTTGATGAG
GACTGTCCTACTTCTGGGGCTGTGTCTCAGAGTTTAGCCTTCCCCCATTCTCTGCACAT
CAATCATCCTGAGGTAAACCAATTTTAAATGTTTGCTTGCAGTAAACTGGCTGCTGACA
ATTTATCTGAACAGATTGAACTAGAAGCCATCTGAGATTTTGAATTTGTTTTTTCCATTT
TTCAGTTTTAATACAAATGCCTGACTGTGCTCAATCATCAAGCTGCATGCATGAGAAAC
TGGCCAGCCCAAACAGTGTATTAATCATTAGCAAATGGAACTTTAAGGAGTCCACTAAG
TGCTTCCTTATAAGGTAGTACGAGTATTTATACTGTAAAACTGATGTAGGGGCTTGACTT
TAGGGGCAGGACCACCGACCAATACATGCAGACTTTGTGTGTGGACAGAGGGGCTTCT
GACATTCAGCTTCGCTAGATACAAACAGAATGAAGAAATGAACTTTTTCTTTTTTTTTC
TTTTTTTTGTAAGAGGTAAGTAAAAGATTTCAATTTGATTCTTCTAGAGGGGGAAAAGT
AGTTGAAAGTAGGTCTTCATTTTGCAGTCATCATCTGTACGAATTCTTCATAGTTGACTT
GTCCGTCGCCATCAATATCTGCTTCTCTGATCATTTCATCTACTTCTTCATCTGTTAGCTT
TTCTCCTAAGTTTGTCATGACGTGGCGCAGTTCTGCCGCACTGATGTAACCATTCCCAT
CCTTGTCAAACACTCGGAAGGCCTCGCGGATCTCTTCTTCGCTATCTGTGTCTTTCATTT
TTCTAGCCATCATAGTCAAGAACTCTGGGAAGTCAATGGTGCCATTGCCATCAGCATCC
ACTTCGTTGATCATATCCTGCAGCTCGGCTTCTGTTGGGTTCTGACCCAGTGACCGCAT
GACGGTCCCCAGTTCCTTGGTTGTGATGGTGCCGTCACCATCTTTATCGAATAGGGAGA
AAGCTTCCTTGAATTCAGCAATCTGCTCTTCAGTCAGCTGATCAGCCATGGTGCGAGCG
AAGGAAGGAAGAACGGAGCAGGCGAGGGTGGCTGCACCAGCGCAGGGGCTGTGACC
GCCGAGGTGCCTCCGCTTCTGCTGCTGCTAACGCGCTACCTGCGTTGTGCGCTGAGCC
TCAGCCGCCGCCCGACTTCGAGTAACGGCACCACGGACACGAGACTCCCA
>c4558_g1
GGCCAAAGCACACCGTTTTTCCTGAAACTGCTCCCAAACCGTGTGGGTGGGCAGATTA
AAAGAGGCAGAAGTCTGAGGTCACTGAACATGATTGCCGAAAGCAAACAAAGAGGG
AGGGGAAAGAATACAGGAAGGAGCTTCCCTGCAGCTAGCGTCTGAAGACAAAAAGGA
GATTTACAGCAAAAATAATCTTTCCCTAACCCCACTCCCTCCAAATTGTGTAAGGATTCT

CAGCAGACGTGGCACAGACAGTTCTACGCGACTTTGTAGATGCCAAACTGCCGTTCAG
ATTTTAGAGCCCCAACTCTTTGGCATTAAATACAAAGAGGATTAGGAAACTGGAAAGA
GGAGGAAACGAGGAACACAGATGCCCGATGGCTCCAGATACACAGAGCTGGACTCGC
TCTGCAGATCCTCAGCCAATTTAAAGAGGAGAGGGTTTGCAGAAAGTCCAGTGGAAA
AGGGGAGGGTGGATGGGTCATTCCATCATGTGTAACCTGGGCAAAATGAGAGGACTTA
TTCAGTAGCTTTGGCATCACCAGCTTCTGCCTTGCCGTCCACTTCCTCATCGGAGCACT
CTCCTGTACCCTTCCTGACAAGCCGACGTGTGACGACAAAGGGCAGGTCCCCACGCCT
GAGTTTGCTCTTGAGGGAGCTGACCTCTCTATTCATTGCATCAGCTGTTTCAGTGGCAT
CGTCCAGTTCGCGTTGGAGCTTCCTGCGGGACGCGTTGGCTCGCTGGGCCTCCTCTTC
TGCTTCCTCAAGCTGGCGTTTCAGCTGCTTCAAGCGCATGTTCGCCTTGTCCGCCTGAT
CTTTGAACTGCTCAGCGTTACGCCTCTCATCATCCACCTGCAGCAATATATCTTTCAGCT
TCTTCTCGGCACGGCGCACTTGCTTGCTGGCAGCCTGGCGCTCCTTGGTCTCCATGTCC
AACTGCTCTTCCAGCTGCACTATCTTCGCTTCCAAAGCCGTGATGGTAGCCTTGTACTT
GGACTTCACTGCACTCTCCATCTCCTGCAGTTTAAGCTTAAGCTCCTTGTTCTGGCGCT
CCATCTGCTGGCGAGCATTCTCATTCTTCTGTGCGTTGCTGCGCTCAGCATTCAGGTCG
GCGTTCATCTGATCGATCTGAAGATTTGCCTTCTTGAGCCGGTCATTGATTATCTCAGTG
TTGCCCTGTTCCTCCTCCAGCTCCTCCTCCAGCTGTGCTATCCGAGCCTCCAGGCGTCT
CTTTTCCTCCATGGCCAGCGCTCCTTTACCACTGCTGTTGGCAATCTCATCAGCCAGCT
CATCCCTTTCTTGCTGGGCCTGGCGCTTGGCACGCTCAGCAGCTGCAAGTTCCTCCTGC
AATTGGATCATCTCTGCCTCCATGCTCTTCAACTTCTTCTCATTCTCTTTGGCCTGAGCC
AAAATCTCTTCTCTGGAGGTACGCGTGTCCTCCAGTTCCCGCATGTAATCCTTCATTTGT
GCCTGCAGTTTGCGGAGCTGCTTGATGGCTTCATCACGATTCTTGTTAGCAGTGTCTAT
ATGGCTTTCCAGATCCTTCAGGTCCAGCTCTAGTTTCTTCCTGGCAGCCACAGCAATGG
AACGCTGTTTTCGTTCATCTTCCAATTCCACCTCCATCTCCCGCACTTGTCTAATCAGCT
GTTTCCTCTTCTCTTCATTCTGTTCATCACGTCCCAGCAGGTCTCGGTCAAACTGTGCCT
TCATGGCCTGTTGGTTCACTTCTAGCCGCAGTTTGGCATCCTCTGTGGCCTGCAGTTCA
TCTTCCAGCTCTTCCAGTTGTGTCTTCATCTCCTCCACCTGCTGCTCCAGGGCTCGCTTA
GCCTTCTCCAGCTCATGGACGCTTTTCCCAACATCATCTTTTGAACTCATCAGGTCTTCC
ATCTCTGTGCGGAACTGCTTATTGACTCGTTCCAGCTCAGCTTTCTGTTCTATGGCTTCT
TCAAGGGCCCTGGCCAGGGAGAGGGCCTTTGTCTCCTTCTCACGAGCTTCAGCTTCA
GCACGGTCCCTCTCTTCTGCGTATTTGGCAGAGATGTTCTTCTCCTCTGCCAAGAGCTG
ATCAAACTTCTTCTGCTTCTTCTCTAGGTTGGAAACTGTCTGGCGCTGATGATCCAGGT
CCACAGCGATGTCATCCAGCTCTTGCTGCAGGCGGGTCTTTGTTTTTTCCAGCTTGTCA
TATGCGGCTATCTTTTCCTCATAACGCTGGGTTAAGCTCTCCAAGTCCTTTTGCAGCTTC
TTCTTGGCTTCTTCTGCTATCTCAAGGCAGCCCAGACCATCATCCATCTTCTTTCTGGCC
TCTACTGCCTGCTGCTGAAGAACGGAGATCTGCTTCTCTAGGTTTCTCTTTGCCTCCTC
TTCTTCTTCCAGTTGCTCTTTCAAAGCATTCTTTTCATCCTCTGTTTGCTTCAGTTTAGT
GCTGAAGCTCAGCTTTAGGCGAGTCTCCTCCTGCAGCAGCTCCTGAGTATCCTGAAGC
TGTGACTCCAGAGCAGAGAAGTCCTTTGCTAATTTGATTGATTTGCTGTCAGACTGGTT
CAAAAGGCCTGTGACATTGTCCAGCTCAACCTGCAACTTGTTCACCCTCTCAGCCAAT
TCTGTCTTCACTCTTTCACCTTCTGTAAACTTCACCTGCAGTTCTTGGAGCTGAGCATC
TACTTTTTTCCTCTTGTGCTCTGCATCCCCTTTGCCTTGCAGAAGTACCTTTACTTCATT
GGACAGTTCAGCCCTTTCACTTTCCAGCGCTTGTTTTGCCTTTTCAAGATTTGCTTTCA
CCCGTTTGGTTTGCTCTAACTGCTCTGCCAGTTCTTCAATGGCTTGTGAATGTTTCTGCC

TCATCTCCTGGATCTGAGCCTCGTGTGTCTTCGCCTCATCCTCAAGGGTCTTCTTCAAA
ACCGTTACTTCTTGTTCTCTCTTTGACCTGAGCTCCTGCTGAGCAGCTGTAGAATCTAG
AGTGTCTTCTAGCTCTGTTTTCAATGCTTCCAGCTCTTCTCCCAGGTCTCGCTTCTGTTT
TTCGGCTTTGTTCCTGGAAGCTCTCTCTGACTCCAGGTCTTCCTGCAGCTCAGTGATCT
GGGACTCGAGTTCCCTGATCTTTTTCAGGGCCATGTTCTTCTGAGCTGCCTCTTCTTCC
ACCCTAGCTAAGGCTGCCTGCAGCTCCTCTTCCTTCTTGGACAGCTGTATTTTGAGTTC
TGCGATCTGAGCCTGCAGCTCAGCAATCTGGTCATGCAGATCACTGGAGTCTCCTTCAA
GCTTTCGACGAGTCTTTTCCAATTCTTGCCTCTGCTTTTCTTCACGTCGAAGACGTTCTT
CCAAGTCTGTGATCATGGCCTCATGCTTGTTCTTGAGTTTGGCTAAACTCTTTGATTTCT
CCTCTTCCTCTGTTAGGTTTGTTGTAAATTCAGACATTCTGTCTTCCAACAGTTTCTTTT
CCTTGGCCAATTTGAGATTCTGATCTTCCAGAACTATCACATCTTCTTCTAGTTTCTTCA
GTTTGGCCTCCGTGGTCACTTTTTCCAGCTGCAACTTCTGCCTTGCACTTTCCTCTTCTT
CGAGCTGCTCCTCTAGTTCCTGAATGTTCTGCTGCATTTTCTTCTTTTCAGCCTGCAGAT
GTTGACACCGTTCCTCTTCTTCTTCCACCCTTGCTTCCAAGTCATGGCATATTTCCTCTA
GTTCTTGTTTCTTGGCTGTCAGGCGGGCTCTTATCTCCTCAGCTTCAGCACACAGTTCA
GCTTCTGCTTGCAACTGTTCTTGCAGCTGCATCTTCTCTGCCATTAGCTGGGCCTGGAA
AGTCTCCATCTCACTCAGTCTATTTTCTGCAGCTAGCTGCTTCTCCTTAACCTTTATTAGT
TCTTCTTCCTTGGCCATCATCTCTTCTTCCTGTCTGCTTACTTGCAATAGGGGCTTCACC
TTGGTGAACAACCTCCACCACTGCCAGTTCCGCAGTTTCAAGTAGGCAGCACAGTTCC
TCTGGAGCACCTTCATGGCTGTCAGCTGCTGCTGGCGCTTGGCAAAAGCTTTTCTGGC
CAGGTAGCCTCTGCAGCATGCTTGGAAGCCAATAATGACATCTGTGATTTTTAGATCTC
TTTCTTCTTCAAGATGAGCAAGGACTCCAGCTCTGAAAAACACTTTACTCTGTCCAATT
CGGTAAAGGTTAGAATCCAGCTCCAATGCTTTGATCATCAGCACACAGGCTTGTTTTCC
ATCCATAAATCCTTTGGGAATTGCATTTGGAGTTAAGATTTCATATCTTTGTCTGAATTCC
TGGAATACAACTCTGTTGGGGAAACCCTGACGACAAATACGGATTCCCTCCAACACTC
CATTGCAGCGAAGTTGATCAAGGACCAAGTGGGGGTCGAGCTTTCCAGCCTTTTTCTC
ATGGTTGGGGATAATGCAACGGACGAAGTTGGGGTTGGTGTTCCTCAGGGTGGCCATC
AGTTTAGCCAGCTGTTCTTTGTAAAGTTGTCCCACTGTTCGGAACATACCCTTCCTGGT
TTTGAAGGCTCCCGGCAACGCTGTTTCTGACATGCCAGCTACCTGATCCAGCCCAACA
ATACGATCAACATCTTTCCACAACTCAGACACAAACTTATCCGATGACTGATGGAGAAG
AGTAGCAATGTTATCATTCAGTGGATCCATGTTCTTCATCAGCCACTCATCTGCTTTATA
ATCCACCTTGCCAGCATAATGTATGATACAGAAGTCAGCTTTGTCTTTCAGCTGTTTTGG
CTTCTGAAATTTGGGGTGCGTGCCTTGTTCTTGTACAACTTTTTCCACAAAGCTTTTGT
CTGTTGCTTTTGGGAACCAGCACTCTTCATCCAGCAGGGCCAGTATACCAGGGGGGCC
AGCCGGCTTCTCTATGAGGTCAATGCAGGGCTGCAGGTCCAGGCCAAAGTCAATGAAA
TTCCACTCTATGCCCTCCCTCTGGTACTCCTCTTGCTCCAGAATGAACATGGTGTGGTT
AAAGAGCTGCTGCAACTTTTCATTTGTGTAATTGATACACAACTGCTCAAAGGAGTTCA
GCTCAAAAATCTCAAAACCAGCAATGTCCAGGATTCCAATGAAGGATGCACCCTGTCG
CTTGGTCTTATCCAGCGCCTTGTTGATGCGCATAACCAGCCACCGGAACATCCGTTCGT
AAGTGGCTTTAGCCAGAGCCTCAATAGCAAAGTCAGCCTGTTCTTTGGTCTGAGCTTTC
TGGACATAATCTCGTCCAACTTTGATGCGAGGAGTCAGAATACCTCTGGTGAAGTCCGT
CACGTTGATACCCAGAAGATGAGACACTTTCTGAGCAGCTGTATTGTCTGGCATAGAG
GCTTGGTCTGTGTTGCGCTCCTTCTTGAAGACTATGTTGCCAAGCTGAAGAACACCTG
AGATAACCTTCAGAAGACCTATTTGTTCCTCATCTGGGATGCCCATGATCCTCATTGCTT

CCATGGTCTCCTGAAACATATCTTTGTCTTGCTGACCCGGGATCGTAACATGTCCATTGG
AAAGGAAGCGGTATTTGTTGTAAGGCTCCAGAAGTAGATCAGTCTTCAAATGTTCTCCT
GCTCCAGACAGTAGGTAGTAGAAGATGTGAAAGGTTCTCTCCTCTTTGGCTTGACGAA
TTGCACGTGACTTCTCCAATAGATAGGTCTCAATATTGGCACCAACAATGTAACCGTTG
ACATCAAAGTTGATTCTGATGAACTTGCCAAATCTTGAGGAGTTGTCATTCTTTACAGT
CTTGGCATTTCCAAAGGCCTCCAGGATGGGATTAGCCTGGAGTAACTGCCTCTCTAGTT
CCCCCTGGTCTTTTTAGACTTGTGTGAGGAAGCAACGTGAGCCAGAT
>c8133_g1
GCTATCACTTAAAATGGCAGCCCCGCCCTTGGCCAATTCGGTACGTCCCCCTCCAACCC
TACACCGTCCTCCTCGATCCTCAACCGCCCCTTCTCGTCGCTGCGTCTGGCATGCCCGT
CCTTCCTCCGTCCCTCTCCTCCCGACCACCACCCCCTCCCACCTCTCCGTGCACCTGGC
CCTCCCTCCGCCCAACCCTCCCAGTCGCCCCTTCCACACACACGACCATCGCTCCCATC
CACTCGTGCCCCATCCACATCCGGCGATAATCACCACTTCGTGTCCGCCAACTCTCCAC
AACCGCCACCATCCCGCACTGGTCCTACAGACGACGACATCGAGGCCGTCATCCAAAT
GGCCATGGCCTCGTCCACCGCCCCCCGCACTTCAAATCCTCCCCGCGATACCCGCACG
CAGTTGTTCGTAGGAAACCTGCCCTATCGGGTTCGTTGGCAAGACCTCAAGGACCTCT
TCCGTCGAGCCGGCACAGTGTTGCGCGCAGACGTCTCATTGGGACCAGACAATCGCTC
TCGAGGGTATGGTACAGTCATGCTGGCCACCGCGGAGGATGCAGGAAGGGCGGTCGA
CATGTTCAACGGATACACATGGCAAACACGCACCCTTGAGGTACGTCCCGACCGTATG
GGTGAAGACATTGCCGCTCCAGGCTTTGGCATCGGCGTGGGTAGCGGTAGCATCGCAT
TGGCCAGTCTGGCGGGTACGAGCGCACTCGGCGGCGTAGGGGTATACGGGAGCCCACT
TCTAGGAAATATGTCACCAGCAACCGGCGCTCTCAGCGGCATCGCTACTCCTGCAGGT
GGGTCCGCTCTACTCTCTCCTGCACCGACAATTTCACGTCTGTCGACCTTGCCGTTCAC
AACGTTGGACGAGGGAGAGCCTGCGTCAAGGCCAGGAACAGGCTCACAAACTAGCA
GGAACGTGTTTGTCGGGAACTTGCCGTTTCACATTCAGTGGCAGGACCTCAAAGATTT
GTTCCGGCAGGCAGGCGCTGTGCAACGCGCGGATGTCGCTTTGGGTGCGGATGGGCGT
TCGCGGGGCTTCGGAACCGTGAGCTTCACAAATGAGGCGGATGCCGAGCGGGCGGTG
AGAATGTTCAATGGGTGAGTGCCTGGTCTAGTAAATGGGCTGCCTTTCTCCTGTTGTTT
GCCACGCATGTCGTTGTCGTGAAAGCATGTTCTGCACTTTGCGTCAGGGATGAGCACG
AGTGTCATGCGCAACTGCGCGATCCATCACAATGAACCCCATGTGCGTTCAGTGAAAG
TACCGGTAATTCTGCCTCATGTACGAGTATAACGGACGGCCGCTCAAGGTCCACTTTGA
CAAGTTTGCGCCCCCCGCGAGTTCATCAGCGCCCATGGTCGGACCGACGGCGTTGTCG
AACCCCCACTCCCCTGCTACTTTCGCCTTTTCGCACACACAGACTTCACCACACGCGCT
CAGTACCCAGGTTTATGGTCAACCAGGTTCATCACGTGCATCCTCGCTCGCACAGCATC
TGCTCCAAACACAGCTAGAACAAGGGCGTGGCCCGACGCCTGCGTCGCTGCTGGGGT
CTCGTCAAGTGCATCGGTACGGTGACGTTGTCGACAACGGACGACAGCAGGACATCTC
GGCGCAGGTCGCGCAGAAACTCATGTTCGCCTCGTCATCGCAGGCTGCAGCGCTGCAG
CAACACCAGGCGGAGTCGCCTCAGCTCGCACATGCGCAGGCCCAGAGACAACAGCAG
CAACAATTCGCGCGGTACACACCGTCATATATACCGATCCTGCCAGCGGTCCAGTCGCC
GTACACGTTCGACTTTCTGCACTCTGGTCCAACCACGCCGTATGATGTGTACGATTTGA
ACACGTACCAGCGGCTGAATGCGGTTGCGGCAGGTCTGGATGGATACGCCAGGGCCGG
GGAGCAGGGTGCGCAAGTTGCGTCGCAGCCGCAAACAATGCCTTCGCTGGGGTACAG
CACGGCAGCGGCGGCGGCGGATGCATCACAGAGCGCGCCATCGAGCGGGCCCACAAA
GCCGTCACCGCCACCGATGCCACAGGCTTCGTCCAAGTCAGAGCCAGGCACGCTGGG

GTCGACAGGTCGTACACTATCGCAGTCTCGAGTCTCACCACCCTCTTCACAGCCGCCG
TCGCAGATGTCCCCCGCAATTTCTGCACGGTCGCAGCCGACGTCTAACCATCACCACC
ACCCCGCGCACCCCGGCCCGATCTCTCTGCCTCCCCCTCCACCAGTGACGGCCTTCCC
CGTCCCGCCCTTGCATACGCTCTCGCCACACTACCCCGTGTCGCCGACGATGCCTGGGG
GGCACCCGATGAACCCGCTGCACCACCCGATGATGGCGGCTGCGCTGGGCATGCCCAT
GATAACGCCGCACGGGCTGCCTCCCATCACGCCGAGCATGCCGTCGTTCACGTTCTTCC
CGCAGATATCACCCGGGCTCCCGTCGCCCGTCGCTGAGCCCGACAATAGGCATCCTCC
ACACGCGCACGGGCACATGCAGCACGTCATGGCATCGTATTCGCCGTTCTCGCCGGGC
GTTAGTATGTCCCCTGGCGCGTTCTGGGGACGTGCTGGGGCGGGCCCAAACCCGTTCA
TCAACCCTGCAGTCGGTGCACCTGTGCGGTCTGGACACGGACACATCTGGTTTTCGCA
GCAGCAGCAAACAGAGCAACAGCAACCTCATCTACCACACCAACATGTTCACGAGCA
GGAGCAAACGATAGCGGGGCAGGAGCAAGAGGGCCGACCCCCTGATGGTTACTTCCC
GCCTGTGCCCGTGTCGATGGCAATGGCGCAGCAGCAGCAGGGCGCGGAAGAGCCCGC
TGGCTACTTTCCCTGGGTACCGCCTGTGCAGGCGCAGGTGGACGGATACCAATCGACG
ACGGCAGAGGTGGCGACGAATACCGCGCTGAACGGGCTCGTGCATGAGCTCGAGCGA
GAGAGCCCGAGCGACAGCGATGGGACGGCTACGACGACGCGGAGCACAGGTACGGG
GACGGACGTGGGGACGGGGACGAGCTCATCCCGCGGGACGTCGTGGCACACGGATCC
TGACACGACGGTGCAGAAGCTACGTGCGCAGGGGGCTGATGTGGGCGGGGCTGGTGC
TGTTGTTGAGGACGCGCCTGCGTCTGTTGTGGTCCCACGTGCGGATTCGAATATGAATG
TGAGTGGGAGCGGGAAGCCGCGCGCGTACTCGACGGAGGGTGGTGCAGGAGGCCCT
GAGCGGGGCACGCTGTCGAGGGCGGACTCGGACCCGGTGAAGCAGATACCGAGGGA
GGAGTCGTCGTGAGTCGTGTGGGTTGGTGTTATTTTGCCATGTACGACGGTTGTCCATC
TTGGCTGGGAAGGTGATGCCTCCCTCTCTCCTGGGATCTGGGTTGACATAGAAAGGGA
AGGGAAGGAATGGGTGAGTGGAAGGAAGTTCGTTGTTGCACTATGTTTTGCAGCTGGC
CTTACTTGGCATACTACACACAGGGATTTACTACGTCTGGTTATCGTCATCGTGCATTCG
ATCGGGTTGGAATTGTAGAGACAAAAGATGTATCTTATTCGAGTTCCTGCGGTCGGTAC
TAAGTATGTGGGTGGAATGTCTTAAATGTGTGCAGGACCCTCATCTGTGACTAATATTAC
AGCGCAGGGTGAATCCCTCCGGGAGACTACGGCGAATAGTCGCAATGTGCTATTGTGA
ACCGTTACAGAGTTGAAAACTGGAACAATATGTGCGATGAGCTGGAACGGATAGCCAC
TTCCATCATCCGCCATATCGCAGCCGCCACAGTCTACTCCCGCGTCTGATCACGGCCGT
TGCCTGTGTGATCGAAACATCCCTGGATCTTGCGGCAGAGTGGCCACACGTTCTTGCT
GACTACTTGACACCACTTCTTCAACGTCTGGGTGAAACACATACCTCGTCTTTGTTGCG
TATGGGCTTCGTGAGCTACGGCACTGCGGACACTAGACCAACACCCATTGTCCGCACC
ATTTTCTTCACCTCTCCTCAGACAATGATGGACACCATGCGCGGTCAACCTCAAGAACT
GGGCATCGGTTGTACGGGCTGGGGAGGAAGCCAGGGTATGGCGGCACTAGAAGGAGT
GGTTGCAGCTATGGAGATGTTCGAAACTCTTGAGTCGTCCATTGAAGCAGCTAATACGA
CCGTCACATCTGATACTATCGTCACATCTCATCTTATACACATCGCAAGCGCACACCCTG
ACACCGCTGAAAGGCCAATGTGGAATATGTGTCCATCTCTGCATCATGTGACATGGGCT
TCCATACCTGCGGAATTGCGAAGGCGCGGAATCCACTACAGCAACATCTTGCTGAAGA
GGCTAACTCACTTCTCCGAATTGCAAGCTTCTGCTGCCGTTGGCACGGCACAAACACC
ATGGTTCAACGTTCTCTCTCCACATACCGTCCTTATCACTGGTTTCCCACAGAAGGGTA
CGAAACGTTCAACCGACACCGCCCCAGTATCAGAACGTAGTCCAGATATGAAACGCAC
CAAAGTGCAACCCGCCTCCCAGTCTCCCGCGGTGATCGAGGCTGCAGCCCTACCCGCT
CCTGCGTGGTCTACGCCAGCGCCAGTGCTGGCTGATACCGGATCTGGGCGTTCTCTCA

GATCTCGTCGGCTCGTAATATTTCAGGAACAACTGCGTCAAGCAAAAATGACGCTCCG
GGCTCGAACAACCCAGATTGTTGAGCTCGTGACGGAAAATCACCTGAAAACCGCGCA
ACAAGCGCGACTTATCATTGAGCAACGCGCGGTGAAGCTTCATTCGGCCTTGCTCAGT
GAGGAACAACAAGCACAGGCATCTAGTGCTGGGCAGCCACCAGTTGGACCCTCCGTG
CAACCTACTCCGGTCCCTTCAGCGTTTGCTCAAACATCAGACCCGCCAGAGCCCCCCG
CCGCTGCAGTAGAGCCAGAGTGGACACTCATCCCCCTCGTCAATCGTCACATGGCCAC
GTGGCAAGGGACGCTCTCCTGGAGTGGATTCGACGAGGAGACTCATCTTCAGAAGGA
CGTGCACACAAGCACTCTCGTCGTGAGCCTTACGATGAGTTCAATGAGGCCAAGCGCG
TTGATACTGACTCTCTCTCAACGTCCTGTGGCACCCCCCCAAGTTTTGACCTATTGGTC
GGTGAAGTACTCTATATCCTGTCTAGGCGAAGACATACGACCGCACCCACAGGCTCCTG
ACGTCGAGGCCAACGAAAAGCACTTTCGTAGTCTCATTCGCTTACTGGCCAAGAGGAA
TGTGTATGCATTTGCTGCGTGGGCCGGCCCAGACGGTGCCGTCAAGACTCGCCTTGCC
ATTTGGCAGGTGGAAGGCTGCCTCAGGGCCGTGTACTTCGCATCCCCCAGAGGTATGC
CGGTGCTGCCCAACGGCGAGGTTCCTGCTCCCGCTCCTGCTCTTGTCCCTGTCCCTGTC
GCTGGCGTACCAACTCCCGCACAGAACATGCCGCCGGTCGTCGCATCCCTCACGGAGC
ACATGGTGCCTGAGCAACAGCCAGATTTGCATCGCTCCCGAACGACAGGAAGATGATC
ATCCTATGGGGCATGGTTCAGAAGACGACGACGACGAAGCACAGCAGCAGCAACAGC
AAACATAGCAACAGCCGCAGAAGCAGCCAGGGCAGCTTCGTGTAGTCAGCACGAGCC
CACATCCGCCGATGAATTCATGTACTACGGCCCTGAACCCGGCGGCGGTTCAAAACCA
GACGCACGGCCAATCCAACATGGATGCGCACGAAGTTCATATTGGTTACGCCCTTGTAA
GCTAGCAATGATTTAGCAAGGAACTTACAGCACTACAGCCCTAACTTACAGCACTACA
GCCCTTATCAGCACGCTTACGAGCACTCATAAGTACGCTTCTGAGCTGTCATAAGTACT
TAAAAGGGGCCGTAGTAAAATAAAACTGGGCTCAAAATGGCAATAGCAAATTTGATTC
TTGCGGGTCCAGTGTGGTTGACGCAAAAAGAGACTTTGCGCAGGACATATGTGAACA
AGTGATGGTGAGATACAACTCGCTCGATCCCGAACTCCTCTGCGAGGAGCTTGGATCC
TGC

>c9830_g2

TACACTGCTCAGTGCCCCCGGTGTAACAACTGGTCCAACCGGGCGTCACGGGTCCATA
TAGGGACGCAACGCAACGACACCCGGTTGGACCAGCTTAATGAACCGGACGGAGTTC
GGATTGCCCGATCTTGATCTGCGGCTGGGGACGAAACTGGATAGACGAGCGCTCCACC
AATTTTCTCGTTTCCTGCCTCGGGAGTCACGTGCCGAAAATTTCCTCGGCAACGACTTT
GATTGGTCCTTAGCAAGGACCTTAAAAGCTTGCTCGACTACTGCCTTTACTGCGTTATT
CTGCAGCAACACCCGCTGATTCGTAGACATGGTGTCATTCCAGTATGCGCATTTCTGAC
ATCTGATGCAAACGATATAGCTCACCTTCGTCGACTCGCACAGATGCGATGGTGAGCAA
ACATTCTGCTTCACGAAACCCTGCCCAGGAAATCGCCAGGAGCACGTCTGATTTGCCC
ATGTCGCTCTTGCTCTCACATGCTATCTGCGCACGTGGACAATTGTATCTATGTCTTCAG
CTTGCGGAGATACCGTGAAGAAGCCAAAACTTGACAATCACTACTCAATGTGCCATTC
CTCCGTGACCTGCCTCGACTGCAGCACGACCTTCAGTGGGCCCATGCAGTTCAAGAGC
CACATACAGTGCATAAGCGAGGCTGAGAAGTATCAAAAAGGGCTTTACAAAGGTCCAA
AGCAGCAGAACGTCGGTCCTCGCGGTGCCGGGCAGAACTACCAGAACGGGGCCGGG
AACACGCATGCGTATTCAGGTGGTAAGTATCAGCAGACGTGGACGCGGCAAGGTGGCG
CCTGGGGCGGCCCACAACAACGTTACGAGGCATCTGGCGCTAACAGCACTCCTTTGGG
CACCCCGGTGCGGATGTCGCCCGTCACAGCAGCTCCGAGCCAGCCACCCAAGACCGG
CGACGCTGAGCCGCAGAAGGTACCTAGTGCGGGCGTCGAGAAAAAGAAAAAGAAAA

AGAGGGAAAGGAAACGCAAATCGGTGGATGGAGCAGAGGAGGTTGCGGTATGTCAAT
GCTTGTCTTCCCGCTTCCTCACACTCATGTTGCGATGTATAGGCACTGCAAGATGGCGA
GGTGCCGAAGAAGAAGAAAACAAGACATTCTGAAGCGGCGGACATAAACTCTCCGTC
GCCTGCAAAACCGTCAGCAGCGGATAAACCTTCCGACAAGAATAGGTCGACAAAGAA
GTCAAAACAAGAGAAGAAGGATAAGAAGGGCAACAAGGCCGGTGTGTCCGACGAGC
GCGTCGCTCCCTCCGTCCTAGTTGAAGAGCCTGTGAGCACAGAGTTGAGGATGAGCGA
AAAGTCGACCGAATCTCACTGTGAACAGGAGACGCACTCAGAGATCCCGGTGACGGA
CAAGAAGAGCAAGAGACACAAGAAGGAAAAGGACGCCGCGACGGTGGTTCCATCAG
AGGAAGACGGTTTGACTGGAGCAGTGGAGACGAATGGCGCAGAGGGCACGGTGGAG
GGCCAGTCAAAGAAGCGGAAACGCGCAAATGTGGAAGCAGATATGTCTGGAACCACC
AAGGAGAAGCGGTCCAAGAAAGATAGAAAATCAAAATCACAATCAAAATCTAAGACG
ACGGCCGGGGAAGAAGACAACGGGGACGCACACGAAAAGATGGATGTCGATATGACG
GCTCTCGTGCCACCGGTGACGGTGGAGGTTGGTGCTGATGCCAAGGAGCAGAAGGAG
AAGGATAAGAAGAGCAAAGACAAGAAGAGCCGGTCGAAGAAGGAAAAGTCAAAGAC
CGCCTAATTTGGGATCATCATCTAATACTCTGTTCTGCTATCCTCTCATCTCTGGGTTATA
TCTGCTCTCATCATCTTGTACCGGCTTCCTGTTCTAAACCGGACTATATCACGGTTATGC
ATCTGGTATTTCTCAAAAATGGATAGATCTCTTTTACGCAAGATGTTAC
>c3191_g1
CTGGCACGCACATCTCCGGTGCAGAGCGATAGCCGATATGCAGCAAAGCGTCTATCATA
TTACAATATTACAGCGATACAGCAAACAAGAGACAGTAAGACAAAGACTAAGGTATAC
ATGGCTGGGCCGGATGAAGGGGAATCTAAGCTGCTGGCAAGGACAGAGGAAGGTCAG
TATTCATCTACCACCCAACAGTCGTCTGGGATCGCATACGCTGAGGCCTCTTAGCATCCT
CTTGCTGTCTCTCAGCGTCCTGCGTGCCTTCTGGAGCGGTCGTTGCTGCTTGAATCTTC
TCCTGTTCGCTCTCCTGAGGCTCTGCCTCCTCCTTGGCGTTCTGGAACGGATTTGCTTT
ATCTTCTGCCTCCATATCCTCTCGTTTTTGTTCCCCGGAGTAGATCTTATGTCCCTTCGCC
TCGAGGTCGGGGTCACGAGCTATTTTTCCCTTTGCGATATCTGCCGCACCCGCGATCCT
GTCACCCAGAGTCACGCTGTGCATTTGTCCATAGTTAGGTCCTATATCGACTGCGCCAG
CGTGGAGTTGCCGGGGGTAGGTTTCATCATTAGTTTGACCTGGATTCGTGTTCGTGTTT
GAAACATCTCCGTCAGGCTGATCAGACGTAGACCTGCTGTTGGGTTGTGGAGACGGGG
ATGATACCTGGTGAGTCGACATCGTAGTGGCTGGACGTGTATAGAAAATCAAGATGGGT
GCAACGGCGCGGGGGTGTCCGCGCAAGTAGACCACTGTTATTTATATTGCAAGGTTAAT
AATAGCGACAGTTGGAGAGACTGATGACGCAGTGGAGTGTGGGGGGACAATATGACG
TCGGTATTATCATCCCGCCCTGATGGTAGCTCCAGAAGAGGG
>c5285_g1
GGCACTATAGTGTAGTGGTTTATCACTTGGGATTTTGAACATGCTGGCGGGCTGCCCGT
CACCGTGTCGGCTTGCCGGTGCGGATCTTTCCCAGAACCCAGGTTCGAACCCTGGTAG
TGCCTACATACACATGTAATTTTGGTGGCGTTAGAGAACCTCTTTGAGTCAAAGATTCC
TGATGTCGCTTCGAGATTTGAATACTGGTTACCTCCTTAACATCTCCTATGATATACATCT
ACATCAACCAGCGATGATTCCGCCTTTGATCATGTTCCATCCCAACTGCTCGTACGTCC
ACCCTAACACAGTACCATCCTGACTATAGCAGTGGTATCGCAGAAGCGAACGATGCATG
CCTGGCTTCTTGTGCACTGCGTCAAGGACAGCCATGTCATCGTTTGAAAGCTGAAGCA
ATGTGATGTTCTGCTTGATCCTCTCCGGGTTCTCGCTCTTCGGAATGACGACCGTTCGC
CTCTGCACGCCCCAGCTTAGTGCAACCTGGCCCGGTGTCGCGCTGTGCTTCTGTGCAA
TGGTCACCACGTCCTGGTCATCAAGGAAGACGGGCTTTCCATCGTACTCCTTTGGTCG

GCCAAGGGGCGAATAAGCAGTGAGTAGGATGCCCTTGTCCTCACAGAAACGTTTAAGC
TCGAAAGATGGGAGACACGGGTGCAGTTCGACCTGATTGGTCGCGGGAACGACCGTC
GTCTCCTTCAGGAGCACCTCAAGCAGCTTGATCGAGAAGTTCGATACACCTATGGAGC
GCACTTTGCCCGTGCTCAGGAGTTTCTCCATATCACGCCATGTCTCGACGTACGTCGGG
CTTTGATCGTGCGGGATAGGCTTGTTGTCCTCGGTGAATGCCTGCGGCCAGTGAACGA
GGAAGAGGTCGACATAGTCGAGGCCGAGCTTGTCGAGCGACTCCTGCAGGGACTCGG
CAACGCGGTGGTGGTGGTTCGGAAGCTTTGTGGTCACGAAGATTTCTTCGCGCGG
GACTCCAGATTCGCGAATCACCTTTCCAACCATCTGCTCGTTGAAGTAGCCGGAAGCC
TAGGGGTCATCGTGAGTATGGCGGGAATACACACATCACAACAGAACCATGGATATGTG
AGCTTACTGTGTCGATATGTCTGTATCCTACCTGCTCATGGTCAAAGACTTTAAGCATGC
ACAAACTTCGATTTGGAGACTTGCCTCAAGGGCAGTCTTGCACATTTTCTCCACAATGT
CTTCGCCACCAGATGGACCCAGCCAACAACTAGCAATCCCACACGTTAGCCCACAACG
ATATCTGTGCTGTTGCGCGGGAAAACGATCAAGCAGAATCGCAATCCGGGGCGTGGAC
ACGCAGTATCTGTAATCTAGCACTGGGGAGCTTGTGCTTACCCTATACCAACAGAAGGT
ATCTTGTAGCCCGAGTTGAGGGTAAAGCTAGGAATATCGGCCGCCATTGTAGGCAAGC
GCGGGGGAGTTCAAGACGGGGTAAGGGGTGCCCAGCTGCCCAATATATGTCAGTCTCT
CTGTATCTGTAGGCAGACTGTTACTAGTCCATTCATCAGAACCTTTGAATATCACAATAC
GCTATGTCACTGCGGCCCGGGTTGCAGCGCCGCTGTCCACCTGACGAAACGTCGCGGC
CCGTTAGTATTACATCTTGAACGCAGCAAAAATTGTATGACCAGGGCAGCTGTGAGATG
AGAGAACCGCCCCCCCAAGCTTGGGAACGTGCGAGTTGGATCCGCCGGCCCGTTTGG
GCGGGAAATGGTGGAAATTGCCTGTAAGAAGTTTAGTCAGTATGTTACTGAACGTTGG
CTGAGGCGAACAACTCCGTTTTGCAAGCATACATCGCTCCGTAATCAACTGTCTA
>c4316_g1
GTGCGAGCAGAAGTCGGTGAGCCCGAACCGCTCCGTGCCGCCGTCCGGGGGTCCCCG
CACCCCCCGCTCGCCCCCGATATGGGGTGCCGAGCCGCCTGCGTGCTGGCCGTGCTGC
TGGCCGGGCTCGTCCCGCTGGGGCAGGGCCAGGAGAGGGAGAAGGTGAAATGCTACG
ACTCCGTGCGGGGCACCATCTACGACTACGGGGCGCTGACCATCGATGGGGATGAGTA
CATCCCCTTCAGAAAGTACGCGGGGAAGATGGTGCTCTTTGTCAACGTGGCCACGTAC
TGAGGGCTCACCCTGCAGTACCTCGAACTGAATGCACTACAAAATGAGCTGGGGCCCT
ATGGACTTGTCGTCCTGGGCTTCCCCTCCAACCAATTTGGGAAGCAGGAGCCTGGCCA
GAACTCCGAGATCCTCCCTGCACTGAAGTACGTGCGGCCAGGAGGTGGCTTCGTCCCC
AACTTCCAGCTCTTCCAGAAAGGGGATGTGAACGGGGCCAAGGAGCAGAAGGTCTAC
AGCTTCCTGAAGAACTCCTGCCCTCCGGTGGCAGAGGAGTTCGGCAACCCCAAGAAC
CTCTTCTGGGAGCCGCTGAGGAACCACGACATCAAATGGAACTTTGAGAAGTTCCTGG
TGGGCACTGACGGGGTGCCCGTCATGCGTTGGTACCACCGCGCCAACATCGCCACTGT
CAAGAACGACATCATCGCCTACATGCGGCAGCAGCGGGGCCAGTAGCCCCCCCAGCTC
ACCCCCCCTGCAACCCCCGACCCGGCCCTGGGGAGCAGAGGATGACATCTCCATGAAG
GCCTGGCCTGAAAGCCCCCACCATGGGGTGGGCTCGGCCCGATCCCGCCCAGGCGCG
GTGCAGCCCTTTGGTGCATTCAGGCTGCCCGCGCTGCGCAGGGATGTGCTCCTCTCCC
AGCCCCGGGGGCCGACCCCCAGTGCTCCAACCCCACAGCAATGCCAAATAAAGGCTC
TGCAACCGCAA
>c16064_g1
GTTGAGTATTTTGTTTTCTAGTGTTCCGATTGTGGGGAAGAGGATAAGTAGGATGGTGA
AGTAAGAGAGGGATGCTATTTGGCCAATGATGATGAAGGGGTGTTCTACTGGTTGGCTT

CCGATTCAGGTTAGGATAAGAAGGTTGGCTACTAGAAGTCAGAATAGGGTTTGGGAGA
GTGGTCGGAAGGTTATTGTTCGTTGTTTAGATTTGTGGAGGAAGGGGATTAGGAAGAG
GATGAGGACTGAGGCTGCTAGGGCTAGTACACCTCCAAGTTTGTTGGGGATGGAGCGT
AGGATGGCATAGGCGAATAGAAAATATCATTCTGGTTTGATATGTGGGGGGGTTACTAG
TGGGTTTGCTGGGGTGAAGTTTTCTGGGTCTCCTAGGAGGTTGGGGGAGAATAGGGCT
AGTGTTAGGAATGGGGTGAGTATGAGAGTTAAGCCCAGAATGTCTTTGAAGGAGTAGT
ATGGGTGAAATGGAATTTTGTCAGAGTCGGATGAGATGCCTAGGGGGTTGTTTGAGCC
TGATTCGTGTAGGAAGGTGAGGTGGATGATAGTAATACCTGCGATTGCAAAGGGGAGG
AGGAAGTGTAAAGCGAAGAATCGGGTAAGGGTTGGGTTGTCGACTGAAAATCCCCCT
CAGGCTCACTCTACTAGGGTGTGTCCAATGTAGGGAATTGCTGAGAATAGGTTTGTGAT
AACGGTGGCCCCTCAGAATGATATTTGGCCCCATGGGAGAACATAGCCCACAAAGGCG
GTGGCTATGAGTGTGAGGAGGAGGATTACTCCTGTGTTTCAGGTTTCCTTGTAGAGGTA
GGAGCCGTAGTATAGGCCTCGTCCGATGTGAAGGAAGATACAGATGAAGAAGAATGAG
GCGCCGTTTGCGTGGAGATTCCGGATGAGTCAGCCGTATTGTACGTTCCGGCAAGTGT
GGGCTACGGAGGAGAAGGCTAGGGATGTGTCTGCTGTGTAGTGCATGGCTAGTAGTAG
GCCGGTGAGGATTTGGGTCATGAGGCAGACTGCTAATAGGGAGCCGAAATTTCATCAA
GCAGAGATGTTGGATGGGGCTGGGAGGTCGATTAGGGAGTTGTTAATTATTTTAGTAG
GGGGTGGGATTTTCGA

>c4563_g1

AAATCAACATTCTATTATGCCATTGAACAATTCATATTGTTACAGCCAGAGACGGAGAC
GTATGCGAGGAGAGCGATAAACAAGGGATAATCAGGGATACAGAGACAACAATACATC
CATAGCTACTACCATGAGACCACGAGGATACCTGACAGGAACACGATCCATGCTCAGC
CAGAGCACATGAGGCAGGCTTCCTTGTTCTCGATCGAGCACATCAATTTCGCTTGCTCA
AGCTCCCGTTCACGCTGGCGCTGAAGTGCTGCAGCGAACTCGGGGTCAGCCTCAGCG
GCTTTCTGGGTACGCTCGTCCAAAGAGAGCGTCGCTGTTGCCGTAGCTAGCGCATCATC
GTTCCCGGCTACGACGGGAGAGGGTGAAGGCGGATCAAAGGTGCCTTGGCCTTTTATC
CCTGGGGTGGCAAGAGAAGGCGTCGAGGGCTCCTGCTTGATGGGCGTAGATGGAGTG
GGAGGGGTGGCAGCGCGGACGATGCCGTTGATACCGTTGGTCTTCGCTGTCTGAGACT
TCTGGGCTGCATTTTTGAGGAATGAACTATCAACGGTGAACTTGATCGCCTGCGCAGCT
GGTCGTGTACGAAGATAGTACATTCCCGTCTTCAAGCCCTTCTTCCAGCCATAGAAGTG
CATGCTCGTCAACTGACCGTTTGTTGGCGACTGCAAATGAACGTTGAGGGACTGCGAC
TGGCAAATGAAGGCGCCGCGATCCGCAGCAAGGTCGAGAACCTTCTTTTGCGAGATCT
CCCAGACGGTCTTGTAAATGGCCTTGATCTCATCGGGAATAATGTCGATGTTCTGAACC
GAGCCGTTGTTGGCGATGATCATATTCTTCATGTCATCGTTCCAGAGACCTAGCTCGACT
AGCTCGCGCAAAAGCCATGGGCAAACGATCTGAAACTCCCCCGCAAGGACACGACGA
GTATAGATATTACTCGTGTACGGCTCAAAGCACTCGTTGTTACCAAGAATCTGACTTGTC
GATGCCGTAGGCATAGGGGCAACAAGCAGAGAGTTCCTAAGGCCGTGCTCCGCGACCT
GAGCTTTCAGGCTCGCCCAGTCCCAAAGATCGGTGGGTGCGACACCCCAGAGGTCGA
ATTGAAGCTTGCCTTGAGATGCGGGGCTGCCTTGGTACGTCTCGTAAGGGCCATCCGTT
TTCGCCATCTCGCAGCTGGCCTCGACCGCCGCATGGTAGATCGTCTCGAAAATCTGCAG
GTTGAGCTTGCGTGCTTCGACGGAGTCGAAAGGCAGACGCAGGGCCATGAAAGCGTC
TGCCAACCCTTGTACACCAACACCAATCGGGCGGTGACGCATGTTTGAACGGCGAGCC
TCCGGAATCGGATAATAATTGCCGTCGATGATACGGTTCAGGTTGAACGCAACCACCTT
CGTAACATCATGCAACTTCTGGAAGTTGTACTTTCCGTGCTCGATGAAGGATGGAAGG

GCGATGGACGCGAGATTGCAAACAGCGGTCTCGTCCGGCGAGGAATATTCAATGATCT
CAGTGCAGAGGTTGGACGACTTGATGGTCCCAAGATTCTGTTGGTTTGACTTCCTATTG
GCCGCGTCCTTGTACAACATGAACGGTCCACCGGTCTCAATTTGCGACTCAAGGATGG
CGTACCAAAGCTTCTGCGCGTCTAGTGTCTTGTGCGCACGACCCTCCCGCTCGTACTGC
TCATAAAGCGATTCGAACTCTTCACCCCATATCTCGTGTAGACCAGGCGCCTCGTTGGG
ACAGAACAACGACCACTTGCCGTTGGCTTCAACTCGCTTCATAAAGAGATCGGGTATC
CAAAGAGCGTAGAACAGATCACGAGCACGCACTTCTTCCTTGCCGTGATTCTTTCGAA
GGTCGAGGAATTCGAAGACGTCGGGGTGCCATGGCTCAAGGTAAATGGCGAAAGCGC
CAGGACGCTTGTTACCGCCCTGATCCACGTATCGGGCAGTGGAGTCAAATACACGGAG
CATAGGAACAATGCCGTTCGAGTAGCCATTGGTACCCGCAATGTAAGATCCGGTCGCAC
GAATGTTGTGTATATTGAGCCCAATACCACCCGCCGTCTTGCTTATCATCGCGCAGTCTT
TGAGTGTCGAGTAAATGCCCTCGATGGAGTCGTCCTTCATGCACACCAGGAAGCATGA
GCTCATCTGCGGATTAGGAGTCCCTGCGTTGAACAGTGTTGGCGAGGCATGAGTGAAG
TATCGCTCAGACATCAAGTTGTACGTCTCCAGTACACGCTCGATATCGGAACCATGGAT
CCCCACGGCCACGCGCATAAGGAGATGTTGTGGTCGCTCGGCCACTCGGCCGTTGAGT
TTGAGGAGATAAGAGCGCTCTAGTGTCTTGAAGCCAAAATAGTTATAGTTGAAATCACG
GTCATAAATGATGGCGGAATCCAACACTTGCGCATTTGCCATAACGATGTCATATGTCCC
CTTAGAGATCATTCCAGAAGGCTTGCCGTTCTTGGGGTTGACATAGCGGTACAAATCGC
TGATGACAGCAGAAAAGTTCTTCTTTGTCTCCTTGTGGAGGTTTGATACGGCAATGCGT
GCAGCGAGAATGGCATAGTCGGGATGCTTCGTCGTTAGATAGGCGGCAGTCTCGGCAG
CGAGATTGTCAAGTTCGACCGTCGTCACTCCTGTATACACACCAGCAATGACCTTCTGG
GTGACGCCAATAGGGTCTACATGATCTTGATCAAGCCCGTAGCATAGTTTGGCTATCCG
AGCCGTGATCTTGTCGAACTGAACGCGTTCCTTCCGACCATCTCGCTTGAACACGTGG
GCCATGACCTGGAGGACTATGTATGAGAAGTGATGCCGTCTATAGGAGGTAAGTAAGG
AAGGGAACCGGCGGGCGAG

>c6329_g1

CTAAAATTGGCGCTGAGAAGTTTGCGCGAATATCCCTTGGCAGAGTCCGTCAAGTTATT
GTTATTTCTGCGCACTGACTACGGCGACGGCGAAATGTTACTGGTTCATATCATGCGC
GGTTGACTGCTTGTATCGCAAATGTGATAGGACTTTCGGCAAGTCTGATTCTCTGCTATA
AGGGAGGACGGCCGCTCCTTTCCTTGTTTGGATAGACACTCGTCAGAATGATCTAGTG
GTGACGTCGATCTCCTCTTCTGAGGCTTATCCTTCACCTTGCTGACGCTTATAAATGCAT
GGGGCCATGAAGTAAGTGAACGAACTCTCTTACAATCTACCCTCCACCATGGACTCTG
CTATAGGATCTGTCAAAGAAGCTTTTACCTCCGTTACAGGGCACACCGCCAAAGTCGC
CGACCTCCAGAAAGTGACCTTGGACCCAGGAACGAAGCCCGCTAAAGGCTTGACGAC
CGACCACGGTGTATTAGTCGCCGACACAGATAACTGGTTACGTGCATCTGATGGTACTC
GTCCTGGTCCCGCCCTTATGGAAGATCAGATTGGTCGCGAGAAAATAACACGTTTCGAT
CATGAGCGTATCCCAGAGGTCTGTGTCCTCTGAGTGAGTGGTTTTCGGACCAAATATTG
TTGCTGACCAGTAGAGTGGCCCTACAGCGTGTTGTCCACGCACGAGGGACCGCCGCAC
ACGGATACTTCAAGGTATTGAGACCACACGGTTTGCCGACGTGGCTAACGTCTATCATG
TGGTGGCAATAGGTCTATGACGATCGGGCGTCCAAGTACACCTACGCACCTGTCCTAAC
GGACGCATCTCGCATCACTCCAACATTCGTCCGCTTCAGCACGGTTCAAGGATCCCGT
GGGAGTGCAGATACTGTCCGCGATGTTCGCGGATTTGCGACCAAATTCTATACGCAGGA
GGGCAACTGGGACATCGTCGGCAACAACATTCCCGTATTCTTTATCCAGGATGCGATAA
AATTCCCGGATATCATTCACGCCGTCAAGCCAGAGCCCCAGAATGAAGTACCCACTGG

GCAGAGCGCCCACAATAACTTCTGGGACTTTGTAGGCCTCCAACCTGAGTCTGCGCAT
ATGATCATGTGGGTTATGTCAGACCGCGCTATTCCGCGCTCTTACCGCATGATGCAAGG
CTTTGGTGTAAACACCTACACTCTCATGAACGCCAAACAAGAGCGTTTCTTCGTCAAG
TTCCACTGGATCCCAGAGCTCGGCGTCCATAGTCTCACATGGGACGAGGCACTCAAAA
TTTCTGGACAAGATCCTGACTTCCATCGCAAGGATCTCGGTGAAGCGATTACCACCGGT
GCTTACCCCAAATGGTACTTTGGTATTCAGGTTATCCCGGAGGCTAGAGAGAATGACTT
CGATTTTGACATTCTGGATGCGACCAAGGTTTGGCCAGAAGAATTGGTTCCGGTCGAC
ATCATTGGCGAAATGGTCCTCAACAAAACAGTCGACGAATTCTTCCCGGAGACCGAGC
AAGTTGCCTTTTGTACTTCCAACGTCGTTCCCGGCATCGGGTTCAGCGACGACCCACTT
CTCCAAGGCAGGAACTTTTCGTACTTCGATACGCAGATTTCTCGTCTAGGGGTCAACTG
GCAAGAGCTTCCCATCAACCGTCCCGTCTGCCCGGTCATGAATCATCAACGCGATGGG
AAGATGCGGCACAAGATTCAGAAAGGTCCCAACTACTGGCCCAACCGTCATTCGGTGG
CCGGACCTGTCCCTCCCAGCGAAGGGGGATATGCAGAATACCCACAGAAAATCCAGGG
CATCAAACTACGTCTGCGCAGCGCGAAGTTCCAGGAACATTACAATCAAGCCCAGCTC
TTCTACAATTCATTATCTGACCCCGAGAAAGCGCATCTCGTCTCTGCGCTTTCGTTTGAG
CTTAGTCATTGCGATGACCCGATCGTCCCCGAAACATACACTAAACTTTTGAACAACAT
CGACTTTAACCTGGCCAAACAGGTGGCCATCAACGTCGGTGGCGTTGTCCCGAACAAA
CCCGCTCGTAACAACCCCGGCCGCGCCTCTTCTAAACTGTCACAACTCAGCTTCGCCC
CGAAGGAGCCCACCATCAAGTCCCGTAGGATTGCCATCCTCCTCGCTGATGGCTTCAAT
TTCGCGGAAGTGCAGGCTTTGCGCGCTGCGTTGAAGGCAGGCATGGCAAACACTTTCA
TCATAGGCCCTCGTCGTGGTTCCGTTTATCCTGTCGGAGAGGAGGTTGGATCCGGCAAG
GGATATATGGCCGATCACCATTACGAAGGCCAGCGCAGCACGCTATTCGATGCCCTCAT
CATCCCCAGTGGCGCCAAGCATGCCCAAGCGCTCGCGAATAACGGACGTACCATTCAC
TGGGTAAGAGAGGCTTTTGGTCATTGTAAGCCCATTGGCGCTCTAGGGGAGGGTGTTG
CCTTCCTCCGCGAAGCCGTTCAGTTGCCGAGCGTGCAGTTCACATCGGATCTCAACAC
TGAGAACGTCACGAACTCGTATGGCGTCGTTACCGCAGGCAAGTACAGTGTTGGATCG
GTTGGGGCGGATACCCTGTCCATCGTTAAGGGCGAGAAAGGGTTCATATCGAACTTCG
CCTACGAGGTCAGTCAGCACCGCTGCTATGCGCGTGAGACGGATGGCTTGGTTTCCAA
GGTGGCCTTCTAGGGAAGAAGGTTTTAGGCAGCAAAGACAACCAACCAACTTTGTGC
GCAATGTACACATACGTACGGGATATTGATATTGCGCACAGCCGAAATTACATCGGGCAT
TGAATGTTGCCCAACTGGGC
>c8932_g1
GTCATCTCATAGCTCGAACGCGAGGGTATATCGTCATAGTTAGCAAGGAAACTGGCAGC
TTGAACGTTGACGTTGAACATCAAGGACAAACCCACTTGCTCTCATCCCTCTGACATG
GCTTTTCCTGAGACTATCAAGGCCATCGCGATCGACGAGGCGGGCGGTATTGATGTCTT
GCAGGTAAAGGACGTTCCTTTCCCCGCAGTCGCTCCGGGAAACATCGTGGTAAAGGTA
CTTTTAAACATTTACGGAAACGACATACCAGTTCGCCTCACCGCGCTCTGTTGGATTATT
ATACCAGGTCGTATATGGAGGAGTCAATTTTATCGACACGTACTATAGGCATGTGTTCAA
CAATCTGGGACTACGTCCGATTATATGCGAGTTGAAGGATACTGAAACGAGACGTGTTC
TTCCCCCAGGAAGGGTCTGTATCCCATCAAGTCCTACCCACAAGTCCTCGGCATGGAG
GCATCGGGCACAATCGTCGCGCTCCCCACAGACGAGTCGGTCCTCACCAACGACGAGT
ACAAGAAGCGCGCCTTCCAGATCGGGACCAAAGTCGCCGTAAACACTCTCGGGACAC
ACGCTCAATACGCCTCTGTCCCATGGTCCAAAGTCTTCCCCATCCCCGCCAACGTCTCC
CTCCTCACCGCCGCCGCCACCCTCCTGCAGGGCCTCACCGCCCTCTCCTTCCTCGAAG

AGTCGTACCACGTCAAGCCCGGCGACACCGTCCTCATTCACACTGTTGCAGGCGGGTT
CGGCCTCATCGCCGCGCAGCTCGCGAAGCGCGCGGGCGCGACCGTCATCGGCACGAC
GTCCACCGAGGAGAAGGCTGCGATCGCGCGCGAGCACGGCGCGGACCATGTCATCCT
CTATACTAAAGAGAACGTCGTCGAGCGCGTGCTCCAACTCACCGCAGGCGAGGGCGT
GCACGCCGTGTATGATGGCGTAGGCAAGGATACCTTCCTCGATAACTTCAAGCTCTTGC
GGCGAAAGGGCACGTTGGTAAGCGTTGGAAACGCGTCAGGGGCCGTCGAACCCATCG
CTCCTCTGAAGCTTGTGGAAAAGAACCTTGCGTTGCTGCGACCTACGATGGCAAACTA
CATCTACACCCCAGAAGAGGCACGATACTACGGGGATAAGTTACTTGAATACGTTTCGA
GGGGAGAGCTCAAGACCATAATTCACAAGGAGTATCCTTTCACGACGGAAGGGGCCC
GCCAAGCACAAACAGACCTTACGACCAAGGGCGGGAGCACAGTTGGCAAATTGATCT
ATAAGATCGCGGACGAGTGAATACTAGTACTTGGCCACGATGGCGCGGGTGTTGTGAC
AAATCAGACGATGCGAGAGGCACTTTGCGAAATCATCATCACATTGACAGAGTTGTTG
AATTGGCATAACTCCGTAGTACTGAACAACTGAACAAATATCAAAATGTGAATATGCGC
>c1494_g1
AATCCATTGGTCTTAGGAACCAAAAACCTTGGTGCAAATCCAAATAAAAGTAATCAATA
TTTTCACAACCTCAATCTTATTAATCTTCATTCTTCTACTATCCCCAATCCTAATTTCAATA
TCAAACCTAATTAAACACATCAACTTCCCACTGTACACCACCACATCAATCAAATTCTC
CTTCATTATTAGCCTCTTACCCCTATTAATATTTTTCCACAATAATATAGAATATATAATTAC
AACCTGGCACTGAGTCACCATAAATTCAATAGAACTTAAAATAAGCTTCAAAACTGACT
TTTTCTCTATCCTGTTTACATCTGTAGCCCTTTTTGTCACATGATCAATTATACAATTCTCT
TCATGATATATACACTCAGACCCAAACATCAATCGATTCATTAAATATCTTACACTATTCC
TGATTACCATGCTTATCCTCACCTCAGCCAACAACATATTTCAACTTTTCATTGGCTGAG
AAGGGGTGGGAATTATATCTTTCCTACTAATTGGATGATGGTACGGACGAACAGACGCA
AATACTGCAGCCCTACAAGCAATCCTCTATAACCGCATCGGAGACATCGGATTCATTTTA
GCTATAGTTTGATTTTCCCTAAACATAAACTCATGAGAACTTCAACAGATTATATTCTCC
AACAACAACGACAATCTAATTCCACTTATAGGCCTATTAATCGCAGCTACAGGAAAATC
AGCACAATTTGGCCTCCACCCATGACTACCATCAGCAATAGAAGGCCCTACACCAGTTT
CAGCACTACTACACTCAAGTACAATAGTAGTTGCAGGAATTTTCCTACTGGTCCGATTC
CACCCCCTCACGACTAATAATAACTTTATTTAACAACTATACTTTGCCTCGGAGCCCTA
ACCACATTATTTACAGCTATTTGTGCTCTCACCCAAAACGACATCAAAAAAATCATTGC
CTTCTCTACATCAAGCCAACTAGGCCTGATAATAGTGACGCTAGGAATAAACCAACCAC
ACCTAGCATTCCTACACATCTGTACCCACGCATTCTTCAAAGCTATACTCTTTATATGCTC
TGGCTCAATCATTCATAGCCTGGCAGACGAACAAGACATCCGAAAAATAGGAAACATC
ACAAAAATCATACCATTCACATCATCATGCCTAGTAATCGGAAGCCTCGCCCTCACAGG
AATACCATTCCTAACAGGGTTCTACTCAAAAGACCTAATTATTGAAGCAATTAATACCTG
CAACACCAACGCCTGAGCCCTACTAATTACACTAATCGCCACTTCTATAACAGCTATGT
ACAGCATACGAATCATTTACTTCGTAACAATAACAAAACCGCGTTTTCCCCCCCTAATCT
CCATTAACGAAAATGACCCAGACCTCATAAACCCAATCAAACGCCTAGCATTCGGAAG
CATCTTTGCAGGATTTGTCATCTCATATAATATTCCACCAACCAGCATTCCAGTCCTCAC
AATACCATGATTTTTAAAAACCACAGCCCTAATTATTTCAGTATTAGGATTCCTAATCGC
ACTAGAACTAAACAACCTAACCATAAAACTATCAATAAATAAAGCAAATCCATATTCAT
CCTTCTCAACTTTACTGGGGTTTTTCCCATCTATTATTCACCGCATTACACCCATAAAATC
TCTCAACCTAAGCCTAAAAACATCCCTAACTCTCCTAGACTTGATCTGGTTAGAAAAAA
CCATCCCAAAATCCACCTCAACTCTTCACACAAACATAACCACTTTAACAACCAACCA

AAAAGGCTTAATTAAATTGTACTTTATATCATTCCTAATTAACATCATCTTAATTATTATCT
TATACTCAATTAATCTCGAGTAATCTCGATAATAATAAAAATACCCGCAAACAAAGATCA
CCCAGCTACTACCATCATTCAAGTAGCACAACTATATATTGCCGCTACCCCAATCCCTCC
TTCCAACATAACTCCAACATCATCAACCTCATACATCAACCAATCTCCCAAACCATCAA
GATTAATTACTCCAACTTCATCATAATAATTAAGCACACAAATTAAAAAAACCTCTATAA
TCACCCCCAATACTAAAAAACCCAAAATTAATCAGTTAGATCCCCAAGTCTCTGGATAT
TCCTCAGTAGCTATAGCAGTCGTATATCCAAACACAACCAACATCCCCCCTAAATAAATT
AAAAAAACTATTAAACCTAAAAACGATCCACCAAACCCTAAAACCATTAAACAACCAA
CAAACCCACTAACAATTAAACCTAAACCTCCATAAATAGGTGAAGGCTTTAATGCTAAC
CCAAGACAACCAACCAAAAATAATGAACTTAAAACAAAAATATAATTATTCATTATTTCT
ACACAGCATTCAACTGCGACCAATGACATGAAAAATCATCGTTGTAATTCAACTACAGA
AACACCTAATGACAAACATACGAAAAACACACCCATTATTTAAAATTATTAACCACTCA
TTCATTGACCTACCTGCCCCATCCAACATTTCATCATGATGAAACTTTGGGTCCCTTCTA
GGAGTCTGCCTAATAGTCCAAATCATTACAGGTCTTTTCTTAGCCATACACTACACATCA
GATACAATAACAGCCTTTTCATCAGTAACACACATTTGTCGAGACGTAAATTACGGGTG
ACTAATCCGATATATACACGCAAACGGAGCCTCAATATTTTTTATTTGCTTATTCCTTCAT
GTCGGACGAGGCTTATATTATGGATCATATACATTTATAGAAACCTGAAACATTGGAGTA
CTTCTACTGTTCGCAGTCATAGCCACAGCATTTATAGGCTACGTCCTTCCATGAGGACA
AATATCATTCTGAGGTGCCACAGTTATTACAAACCTCCTATCAGCCATCCCATATATTGG
AACAACCCTAGTCGAATGAATTTGAGGGGGCTTCTCAGTAGACAAAGCCACCTTGACC
CGATTCTTCGCTTTCCACTTCATCTTACCATTTATTATCGCGGCCCTAGCAATCGTTCACC
TCCTCTTCCTCCACGAAACAGGATCAAACAACCCAACAGGATTAAACTCAGATGCAGA
TAAAATTCCATTTCACCCCTACTATACAATCAAAGATATCCTAGGTATCCTAATCATATTC
TTAATTCTCATAACCCTAGTATTATTTTTCCCAGACATACTAGGAGACCCAGACAACTAC
ATACCAGCTAATCCACTAAACACCCCACCCCATATTAAACCCGAATGATATTTCCTATTT
GCATACGCCATTCTACGCTCAATCCCCAATAAACTAGGAGGTGTCCTAGCCTTAATCTTA
TCTATCCTAATTTTAGCCCTAATACCTTTCCTTCATACCTCAAAGCAACGAAGCCTAATAT
TCCGCCCAATCACACAAATTTTGTACTGAATCCTAGTAGCCAACCTACTTATCTTAACCT
GAATTGGGGGCCAACCAGTAGAACACCCATTTATTATCATTGGCCAACTAGCCTCCATC
TCATACTTCTCAATCATCTTAATTCTTATACCAATCTCAGGAATTATCGAAGACAAAATAC
TAAAATTATATCCATGTCTTGATAGTATAAACATTACTCTGGTCTTGTAAACCTGAAATGA
AG

>c8851_g1

CTCAAATACCCGCGGCATTACGTAATATATGATATGGGCATAGACAATTACAGCGCGTAC
TTGGCAGCACAACCCACAACGTTGACCAATCCCGGGGAAGGGTAAACAAATACACAG
AGACCATACAAACCCGATGGTATACTAAAATTATCACATACACTAGACACAAGACGCGC
TCCAAAGAACTGAGAAAGACGAAGAGTAAAACATGCAGGTAATCCAGGTACTGAACA
AATAAATCATAGACGAGATGCCGCTAACCTATCAGCGTTCATGTTCGTGTTTCTTCGAA
GACTGACGGGCCAAGATCTTCTTAGCCGTACTGTACACCCCGTCGTCAGAGTCGCTCG
AGTCGTCCTCCCACACGTCTCGCGCGCCGTGCTGCTCAATGCGTGTGCCCCACCCGTG
CGGAGCGATACTTCCTCCGCCTGGGGACGCCCACGGCGCATGCCGCGACTGCTCGTCC
TCTTCCGTGTCCTCGATCGCGGGAGGCGTCACAGAAAAAGTCGGCGGCAGAGACATC
CGTACGCTCTTCCTGCGTTTCGGTGGCTTGTCAGTTGTCGCCGTAAGGTTCGTCGAGTT
TGTCGAGTGCGACAACTCGCTCCCGTCTTGCTTGTGAGCGTCGGTATCCAACTGTGATG

GAGCAGACAGTTGCGTTGTTTGGGTTGACTCAGGTGAGGAAGGCCTTTGGACGGGTG
AAGCTGGCGGCGATGGCTCATCATCTAAGACCTCATGTCCGGTCTCGTACGACGAGATA
GATGAGATGTCGCTTAGGCGCCGCTGCATGGGAGGTCGCACCACACTGAATGTTGGTG
CAATATCAGGTACGACATCTGGAGGTCCCGATATACCATTGGTCGGTTTGGAAGACGAA
GCACCGTTCCGATTAACCTCCGAGTGAACAGGCTGGGGAGACGGCGTCCGACTTGCTC
TAAGCGCAGATCGAAGGGGCATCTTGACTGGAGTTGGGAGCGGTTTTGACCGATCACT
CAGGGGCTGCGACGGTGCAGATGCTGACCCATTCATATGCAACGAATTTCCGTAGCTGT
TCTCCTCCAGCGGCTGGTGGCTACCCTTTGCGTCTCGACGAACAGGAGAGCCGCCGGT
GTCAGGGCTAACCACAGGAATTTCGCCAATACTATCGCGAATTGAAACGCCGATTGGA
CCGGGGGCTTTGGGTACAACAAGGAGTCGATTCGGATCCTGCTTTATGGCAGCCTCTTC
GTTCAAGCGCGAGATGCCCTCAACGAGGCTTATTAAGTTTGACTCGCCGTTCGTCAGA
GTGAGTCGAGTGGGCACGGTCGTTGACATGGTACGTTTATGTCCATTTGGCGTCGACG
GAGCGGTCTTTGCAGGGGAGGTATTCAACGATGCGGTCCGCTTCCGCGGGGCTGTATT
TCGTCGGCCTGGCGTAGATTGAGTAATGGCAGTCGTCCTCGGAGGGGCGCTGGCAACG
CTCGTGACAGACTGCTTGGAAACGGACGAGTTGCGAGAGAGCGACGAATCTCCTTCC
GCGGCCACCACTGCATTGATGGTCGGAGATTTCTTCAGTCGCCCGCTACCCGATGGGCC
CGGGACCCCATTCGGAATCTTGACAGCACTCCCAAAGACATCCACAGTGGACTGTTTC
GTCGGCGTCTGCTGCCGTCTGCCCTTCGCGAGTACGTTGCTCATAGAGTCAGTCACCGT
ATCACTCGTATAACCCTTGTCCTTGGGCGGCGCCGTGTGAGAAGGCGATTGAACCTGC
GACGAGCTCCTCTTGGACGCCCGCTTCTTGAGTTTCGCCGTACCGCCACCCTCCAGTG
CATTCCCGTTTAACAAAATCGATTGCGGAGAGTCAGTACGCCCAGCGTACGAGTGCGA
GAGATGTGGGCTCTCGTCGTCCGAGCTGTCACCGCGTCGCGCCGCAGCAACATTTTG
TCGATCCGCGTCTGCCACCGCCCGTTTGCTTTTGATGACCCGTTTGACGAAGTTTCTTC
GTCAACCCGCGAGCCTCCGACGTGGAACAGTGCGGCAAGACTGCCAAGCATGCCTGC
ACGGCCGCGCTTCTTGTTGGGAGAGTCTCCGCGCAGCGGAAAAGAGGTCGGTACGTC
GTCCCTTGGGGACCCCTTGGGACCACGGACAGTGAAGGAAACATGGCGCGAGGGGGA
AGGAGGAGGGTCGGTTTTCGCTTTGGGCTTCCGCTTGCTAAAGCGGGGGTTTTCGTAT
ACTTCTAGATGACTTGCGTCCTCTTCTTCCGCACTACTCCCACCCGTCTCCTCGACTATC
GTCGCAGGAGTCGCTACACCATTCGGCAAATCAAAATCTTTGATGGAATGGGCCGCGG
GTATATGGAGAGTATATGGATTCGAAGGCGTTGTCGTTGTGAAAGCAGACGATGTGCGG
AATGTCGGGCCCACCGCGAGACTACTATCAGACGGTGAATGCCCAATTTGCGAGGCTG
ACTGTGAAGGAGCTAAGGAAGAAACATCAGATAGTGCTTTCTCATAACCAGGGAGGG
GTTTGTGAAGGTATGGATGCATCGAAGGACTTCCATGGCCATTTGGCATGTCCAACGTC
TCCAACGCCCGGATGCCCTCCTTCCCCATCTCTCCCCACGCCCAGCCGCATTCTACCAT
CGCTTTGCAGCGCGCGTGTAGTCCGGACCTGACTGTATGCGTGCGGAACGCTTCCAGT
TCCTGCTCCTTCGCAGCCAACTGTGCCTCACAAGCCTGCAGTTCTGTCTGTGCGTTTGT
CATTCGAGGCCCAATTTGGACTTCCGACTTCGATGTGGGGATGCTGAGCGAGGAGCCG
GACGGGGAGCTTCCCAAGTATGAATCTCTATTCGACTTTTGCTGCTTCGTCGCCTTGAT
GAGGCGTGTCACCCTGAGCGGAGATAGTATTTCGTCAGTACATGACATGATAATACTTA
GCTTCAGGCGCGATAATGCAGCATCGAAACCTTGTTCTGACACAAGCACGATGCAAGG
TGTACCTATACGCATTTCCACCATACCTTACAAGCCGGACTCATCAGGACTGCGAGGTC
CGTTGGAGGAAAGCATGCCAGCCCGAGAGAAGCGGCATCGACGAGGCCCCAATCGCA
TGCCTACCTTGCGCTTGGGAATGCAACAACGGAACGCGCACGAGACGAAACTTGCCG
CATGCGACTGACCGCGGATGGAAGTAGGAACCGAACACTCGGTGTACAACTCAGCGTT

ACCAGACGAAGGCTTATGGACATTGTGTGGCGAAATCCCAGGTACCCACTTAGGCGGT
AGAAGATAATGCGGCAGAAGTGCCACGCATCGACATGTCACCTCGTCCCCGCTGCTAT
ATAGAAGCAACCCCGCGCCAGAGTATGGTCCCTGACCTGCCGGATGACCGCACCCCGA
AGCAACAGCGGCGTTCTGTTCCCTCAGACACTGCTCGCGCGGCTGGACATGACGAAAT
AGGAAGAACCAGTATACAATACTCACAGAATCTCCCTATCACGCATGATGTTGCCAAGA
TCATCCTCCATCTTCTTCAGCACCTTCAGCTGTTCTTGCCACCGCCCCACGGCGGACGC
ATAGTTCCGCAACGCGTCGTCCGCACCGGCAAGGGCCCCCGCTACAGCAACGATGACC
TGCGAAGTCGGCGGCGCAGATGCAGCAGCATACGCAGAGAAAGACGCGAGAGACGCT
TGGGAGTATTCAAGGAGGATGGAGAAGTGTTTCGAATAGTCCTTTTCGTGGCTGAGCA
GGTTGGTGAGCAGGCGCGAGTCTGCCGGCCGGTGAACCATGCGAGCGTGGATTCACG
AAGGCAGTAGAGGTAGAAAGTGGAAAGTCCAAGTCCAGAGTTTCTGCAGCCAAAGTC
GTAGAGAAGGCGCAGAGACTCACACGGCGAGCGGGGAAGAGGTTGTACTCAAGCACT
TGGCAGTAGTGATGAGGTTGAGCGTAAAGTGACGTACCGAGCGCTCCAGACGCCCATG
CGCCGCATCAGGCAAGCAGCCATGCCCTTGCTCATGGCTTCCGTCTCACATTGATGAAT
GCCAGAACATCGTTGAACACAAGACAGAGTTGTAAGCACAGCCTCGCGGCTGAAATAT
GAATATGCTGAAGAAGGCTCCAACATGCATCTGCTCCACAAGTTTCCCATCGACATAGC
ATAGTTCATAGTACATGTTTAACTCAGTCGCACGCGCAGTTGGACGCTGGACG
>c4388_g1
TGGTTGTTATTGTCACATAATCAAACTCGCAGAAACCTCGCTACACTGCGGGTTCATGC
TATCTGCTTCGACATGAAAGGTTTGTAGTTTCTGATGACATGATTTTCCTGCCGGTTCAG
GATCTTTGATCCCCAAGTCCGATATTCGCATTCCTGGTATACTATTGTCCTCATGCTCTTG
CTCATGTGCATGAGCTTCCGTTTGCTTGATAGTACTTTGTGCATCCCCGCCCGCGTGCG
ACAGCTTGCTCACCAATTGCACACAGTTTCACCGCGCACACTGGCTCGCAAGAGGCAT
GTCACGGTTGCGCGACCCGGTGGATAAATTAGTCCGTCAGGTGCCAGTGCGGCCTCGG
ACACTCACGCGGACTTGTCTCGGCGACTGGCTCAGACTACTGTGGAGGTCTCCAAATA
TCCGGTACTGTAAGCCGCGTTGCGGTATAAAAAGAGCCATTTTCTTTGATCCTGCTGAC
CGTCCGCTGAGTCTGACTAACGCGACCTCGTATTCGACATGAGCTTCAAGTCCCTTCTG
GCATCCGTTATCCTCGTCGCTGCTGCTGCGTCCGCGCAACTCACTGGGAGTGTGGGGC
CCACGACTCCGCTGTCTGAAAAGAGCACAATCTGTAACGTCCTAAACTATGGTGGCAG
CGTGGGCTCTAGCGACATCGGTCCTGCTATTACCAGCGCGTTTAACAATTGTGTGCTGA
AGAACTCCGGTTCGACCTTGTATGTTCCCGCCGGTAAATACCAGATTAAGACCTGGGTC
GACCTCAAGGAAGGCCACAAGTGGGCTTTCCGTCTTGATGGGTTCATCACCCGTGCTG
CTACCACGGGTGGTAATATGATTGCCATTGAGAACGCCAATGACTTCGAATTTTACTCA
GCGAACAGCGCAGGAGGTATCCAAGGGAACGGGTATCAAGCACGCAACGCAGGCCCT
CGTCTGATCCGTATCATCAACTCCCAGAACTGGTCCGTGCACGACTTAGTTCTCGTTGA
CTCTCCTGAGTTCCACCTAGTCATACAATCCGGCCAGAATGGAGAAGTGTACAACATG
GCTATTCGTGGTGCTGACATCGGCGGCTCGGACGGCGTGGACGTGTGGGGCAATAATC
ACTGGATCCATGATATCGAGGTCACCAACCGCGATGAGTGCGTCACTGTCAAGTCGCC
TGCAACCAACATCCTTATTGAGCGCATCTGGTGCAACCAGTCTGGAGGTAGTGCTATCG
GTAGTCTGTCCGACGGGACGGCAATTGAGAACATCCTCTACCGGAACGTCTATACGAA
CGGAGGAAACCAGGCGTTCATGATCAAAAGCAACCTTGGCTCTGGCTACGTCAGGGA
CGTCCTATTTCAGAATTTTATCTCTCGTGGCACCGCATATGGGCTCAACATCAACCAGTA
CTGGTCCTCTCAGACTGCTGGCACGGGTGCTGGCGTAGAGCTTTCCAACATCACATTCT
CTAACTGGGACGGCAACGTCGTCAATGGTGTACAACGCCCACCTGTCCAGTTAATTTG

TGCGGACGGCGCGCCCTGCTACGATATCACGTTAAGCAACGTGAACATGTGGTCGCAG
AACGACCAGGCTGTCGTCAAGTGCGAGAGTGCGTACGGCTCGGGCCTGTCGTGCATC
AAGAGCGGGTCGTCGCACACCTCCTACGCGATAATTACGCAGTCCGCGACACGCCCGT
CTGGTTATACGACTCCCACTACGATGGCTGGTGACCTCGCCTCTGGATTTGCGACGAAC
GCTGCTATTCCTACCCCGACGATTCCGACGTCATTCTTCCCTGGTCTGCCTCAAATTAGT
CCTTTGGCGAGGAACAAATGAGCTAGGCCGACTGAGCAAAGGCAGTTCGGTACAACG
TATACTCCTCGTGGAATAACCGTCTGTTCTGTTTGAATGAGTCCTATGAGTTACGATAGG
GTGGTCGCTGTTAATAGTAATAAATGGGCTAGCGCCCGCGAGCGCTTGGCAGCAGAAG
CTGTGTTGATCGA
>c4271_g1
CGTCGAGTGGTTCATGAGCGGTGCGAGCTGGTTGCAAGACGGGACCAGACGCCCTCA
ACGAAAGCGGCGGCTGAGACGACGCTCACAGCAGGGCTCGTTCTCGTGTGATTGAGA
GAGTACGAGTTGATGGTCCCAGCGTCTGGAAGCCTTCGCACATACACTTATTGCTCCTG
CGCCGCTTAGAAGACAACACCCACTCAGGTGTGAGCACAACTATCATCGTCACTTTGG
CAGATTCCGATCAAAATCAGTCCCTAAAGCCTGGCATTCTGTCGTTGCCCCATGGATGA
TACAGGTATTGGCGGAAACATAGCATAAAAACGAAGATGTGTCAGATACACGATCCCT
GCACGCTGCTGGTCGAGTTTTTCACGAACGGCGCGAGCTGGTTGTTCGCGCACAAAGC
GCCCTTAACGAAAGCGGCGGAGACCACGTTCGCGCCAGCAGGGCTCGTATGTGTGTG
GTCGTTGGGGTAGTACGAGTTGACCGTCTGGTTTCCGAGCGTCTGAAAGATGTTCGCG
ACGTACTGCCCGTGGTCAACGTACATCGCGCCGAGGGCCTGCGCCGCTGTTTGCGCGT
ACCCTACGAAGCGCGACGGCACATAGCTGAACGTGCCGCCTTCCCATGGATTGTCCGG
TGTCTGGCTGCTGATAATGACGTTCGCACCTTTGGCCATGAGCGCCTTGCCCGCCTGCA
CCTCGTAGTAGACGAAAGTGTATACAGTCTCGCCCGTCGTCGGGCTGATACACGTCTCC
TCCCCGACCCCGGGACAGTCCGTGCGCCCGTTGTCGGTCGTGCTGAGAGAACCCCCAT
CGTTGTGTCCGAACTCGATGACGACGAAGTCTCCGGGCGCGACGGCGTCGATGATAGA
CTGGAAACGGCCCTCGTTCGTATAGGACCGCGCGGAGCGGCCACCAATCGCATCGTTG
ATGACTTGAATGTCCAGGGAGTAGCCAAGGTATTGACCCCAGCCGTCAGTGCCCGTGC
CAGCGCCTCCACCATTTTTGGCCATGGTAGAGTCACCCGCTAAGTAGACCTTCGGCGA
AGCACGCCTCGCGATCGTCCCTACCGTTCCTGCAAGAGCGGTGGTGGATGCGATGAGA
GCCGGCAACAAGGCAAGGATGAAGAACTTCATGACCAAGGGCGTGGGGTGTGGACTG
CAGGTGG
>c9511_g1
GGGCCACGGGTCGCCCAGTGGGTCGTCTGCGCTTGCGCTAGCGTCGGTGAAGAGGCC
CGTGGACAAGACGAGGGAACTTCCAACAATTGAGGTGTAGACTTCATCAAGCTCAAG
TATGCTCGCGCGGAACGGCACGCAAGATTTGAACAGCTCGCGACCCATTTGGTAGTGC
TGTGTTCCTTGACCTGAGAAGATGAACACGATCGGGGGCTTCGTCTTCGACGTGATCA
CTGGCTCTGTGAATCTCGCCGCTTGGTGCGCACCTACGACGGCGAACGAGCGCCACGT
CATGGATCGTGCGCGACGCCCGTAGATCTTTGCCAGCGCCGCCTTCTCATCAGATGCAT
TCGCGAGTGCAAGGAGGCTCTCGCCCAAAGTGGTTGTAGAGCGTGGGGACAGCCCGC
CTGCGACGAACAGAGCAGGGACCTCTACGTCTTTTCTCCAAAATGCGGGGGGCGGTAC
ACGCGACGGTGGTCCTTCGATGACCGCATGTCCGTTCGCGCCACCTATTCCTGAGCTGG
TCATCGCAACAAGAGAACGTTTGGTGGGCGAACGGCATTTGAGTGGTTCGGCAACAA
CGGGGGCGCGCAGACGGTACTGCGACCATCGTATTGCGGGGTTCAACTTTGTCAGATT
CGCGGTGGGAGGGATGAGTCCTCTCTCGAAAATTCCGCAGACTTTGCATAGCGATGCA

AGGAACGCGGTAATTTCGAGGTGCCCAACGTTTCCCTTTACACTACCTATAACTAATTC
GTCATCCCTTTGGAAGGCAGCACCGACCCAATTCGCCTCGGTGGGGTCACCCTGTGCC
GTTCCAGTTGCATGCAGCTCGATAAAGTCAACCTCGCTGGGCGAGCGCCTGGCTTGGT
GGAAAGCGCGGAGCATCGCATCGCGTTGCGCGATAGCTGAGGGAGCATTGGCAGGGA
CGAGGGAGCCGGAGGAGTTCACGCCAGTCCCGAGGATCGTTCCATAGATGTGGTCATG
GTCACGAAGAGCAGCTTCCAGGGGCTTGAGGACAATGGCAACAACACCCTCACCAC
GACCAAAGCCGTTCGCAGATATGTCAAATGGCTTGCACTTCCCATCCGGTGCCAGGATT
CCTCCTTGAGTATACGAGAGCCATTCCGCAAATCTATGATTGATCTGAGAACCTCCAAC
GACAGCAGCATCACATTCTCCGCTATGCAATGCTTGTATCGCTAGATGTGTGGTGTACA
GGGTCGAACTACATGCCGTGTCGATGGGAACAGTTGGTCCACGCAGGTCGAAGTGATA
TGATACTCGGTTCGCAATCGCTGCAGGAGCATATGCAAAAGAACCTGTAGCCTCCATGT
CATCGTGACCTGACACCGAAAATGTGTCATAAGGGACGGCGGACATGTAGCACCCCAC
GTTGTGACCCCTGTAGTCTATACCAGAGTTCCAGAGACTCAGGAAGGTAACCTCTATGA
GCTTCCGGACGGTGAGAGGTAAGAGGCGTGCGTCCTTGGCTGCGATTCCAAACTCCAT
GTAGTCAAACAAGTCGACATCCTTGAGGAATGTTCCTGTGTCGGCGCAAACCTGCCCG
ACGGCACGCCCTTTAATGCTTTGAGTGTTGAAACGCGTAGGGGGGATTGGTTCATAAG
CCTCGCCCTTGCGCAAAAGAAAGTCTATGAAGGATTGATAACCGAGGTTGGCGTCGGT
TGGGCCGAGGCCACTCGGGAGCTGAGCAGCAATCCCGACGATAGCTGTTGCAAACGG
AGTCTGTGAGGTTGGCTGAGACATCAGGGTTAGTAATGCGAGTTACGGCGCTATATGCA
AACGATGTCCTGCGGACCCCAGAGACCGACAGAGAAAGAGCGCAACTACCTAAATAG
CTGTCCGCTTCTTGAGCGGCGAACGGTACTGCAGGGTCCTGAGGAGTCGGAATACCAA
GTGGAGATGGTGCACGTATTATATCGGCAGTTTAATGTAACCCTGAACAAAGGGTACCG
GGTTTGGTAGCCACATGCCACTTGGATGGTTGCTTGTTCTAGCGACTGCGAAGACTCCG
ATGGAAGTGTGACTGCGCGGGGCTCGCACCCAATTGCGGTGTATCAAGATTATGATTAT
GCCATATACACACGAACCACGGACCTGTCAAGATGTGGTCAATGTTCCGACAATGCCG
TACGTACGGACGCTGATAAACCGTCCAAATTGAACAGAATCGAGGATGGTCTGAAAGC
CGTATATATATGCTTACATAACAATGGCTGCGGACCGCCTTGTCCTCGCTCGGTTGCGAC
CACAACTCACCTCCGTAATATTCACAGGCTGGGACTCATTTGTACAGCAATACTGGTAG
CGCATATCTGGCAGGCTTCTTTAGTCGAGGCCTTGGCCTTCAAGGGCAATCTGTCAACG
GCCCTGAACCCAATCACCAACGTGATACGGAGCTCCCACGGACAACTTGCGTTTCTGT
GGCACATCCACCTCTGGTGGTAGCACTGGAACATCAGATCTGAGAAAATGAATGTGTC
CAACACGAGAATGTCCAGGAACAAGTATAGCCTGATAGAAATAGAAACAACAATAACG
CTTCTGCCCGTTGATTATGCTCTGGGCATACAGGTCGCCGGCCTACGCCTGCGATCTCC
CACCACAACCTCTCTCTACAGCATGTCACTCATCACTGCCTCTGCCCACCCGGGAACTA
CCAATGTCTCCTCTCCCGAGGAGCTCGTCAACATAGCCGCGCGCTACAGCTCAAATCTC
CCTCGACGCTTGGCGCCTCTGCATCCACGAGAGCCGGGCGCAAAAGACACCATCCTCG
TCACTGGAACGACAGGAAATTTCGGATGCAGTCTCTTGGAGGCTCTCTTGCGCGACGA
CTCGGTCGGGGTAGTCTATGCGGTCAACCGCAGAGGTGCACAAGCTCTGGAAAAGCA
GAGAGCGGCGTTCCGCGAACGCGAGTGCGACGAGGCTTTGCTCAGTTCTTCGAAGCT
TCAGATGGTACAGGGCGACCTCGAGGCACCCGGTCTGGGTATAGACCCGATAATACTG
GAAGAGATACGCACCTCAGTGACTTGCATCATACACAACGCATGGAGTACAAACTTCA
ATCAACATCTCTCGTCCTTTGAGGTCTATTTCGTGCCTTGCGGCACCTCATCGACCTCG
CCCTTAGTTCTCCCTTCTCCGATCCACCAAAGATACAGCTCGTCAGCACTATATCGGT

GTTCCAAAACTGCCAAGACTCGTTGCGGCCCTTGCCCGAAGCACCCATCGACCCATCG
TGGGCCCTTGGCACGGGATACGCAGAATCGAAATGGATCGCAGAGAAAATCCTTCTCA
GCGTTACTGCTCAGAGCGGAATGCCCGTGCAGATCGTCCGACTTGGCCAGCTGTGCGG
GGAACGCAACGGATACTGGAAAACAGACGAATGGTTCCCAACCATCGTCAAATCTGCG
CACATCACCAAGGGGATACCTGACATTGACGGGGTGAGTGAGTGGGGCAGCACAACC
AACGCACGAAGCACCACCACTTGTACACACGCAGATGGTGGCGCGTTGTGCCTCCACC
CCACCAGCACCGGAATGCGTGGCACGCTGACGCCCCCCACCTCCGGCACAGGTCGCA
GCTTTTGTCCCAAACGACGCTGCCGCAGGCGCGCTGATAGAGATGCGCGCAGCGCCCT
TTGTCATCCTGCACCTTGTTCACCCCCGGCCGCCGCGCCTGCATACGCTCTTCGAAACC
GTTGCCGACGAGCTGAGTTTACCACTCTTGCCGTATGACAAATGGTTGCGCTCCCTTGC
GCACAGCGCACAGTCTATTGCTGTAGGTCAGTGATGATCATCACGAAACTGTCACCTCT
TCTCCCTTGTCTTCCTTCTCTCTCTCCTTCCTTTTCTCTTCTCACTCGGGATGCCATAAAT
GCAAATGCTAACGGCGTTCCTGGCCCCCCTCCCCTTGTCGTCCAGCGGCATCACGGGA
AGGACAGATGCAGATGCTCCGGCAGAATCCGGCGGTGCTGTTCCTTGACTTTTTCTCC
AAATGGCGGCTTACGAGAGATGGCGAGTTGGCTCCGTTTATAGGCCTGTCTACGGAGA
ACGCACGCGCAGCGTCCGAAACGCTTGCGTATCTCCCGGAGCTAGGAGACGATGTAGC
GATACATTGGCTCTCAGCGTGGCGGAGCGCTGGCGCCCTGTTTACTTTGTACTGAATAG
GTGGGGTCCGGTACCGGTGTTTGTTCTGTATTGTACGTGTCTCAGGTTGTTATTGCATTT
AGAAGAACCGGAGAAAGGAATCAGCGGCGATTGGATGAGGATAGTGTATAGATATAAA
TTAGACGCGCCAGTCATGAGATTACACAGTACGATATACACAGCGCCCTACGAATAACA
GCGTCCACGGGATAGTAGTCGATGTAAAAATACCCATCAGGTGTGCATGCGGACCACT
GCGCACGTGAAAGGGGGGTCGCAAGCTCACGCAGGTTCCGCCCAATTAACATCTACAC
CTTGTACATATCCCCATAGGCCCGACCGTGCCCCTCCGAGTCAGGCCTCATCTGCACGA
GCTCCACCGCCGCCTTTGAAGGCAGATCGTCGCGCGTGACGTATTTGTCCTCTACCACC
TCGATGTGGAGCTCGCGCTGGCGCACGCGCCCTGAACCGTCCTGCGGAACAAGGGCG
CGGCTCCTGATGACGGAGCCTGTGCTCTCTGAGAGACTCACGGTGGTCGGGCCCCGCG
TGTCGCCGCTCCGGCGGACAAAGGCCCGGTTGTTTAAGCTCACGACGAGCGAGTTGG
AGTACACCTTTGGAAGGATCAACGCGGGGCATTCAAACAGAAGGTTGTCTGTTTCCAC
GAGGAAGAGTACTGCGTAGATGAGCGCGATCGATGCAGTGAGTGCCCCGGTCTCGATC
ACGAGTTGCACGATGCGATCCACAATGCCATCAGAGTGATGCAGTCCCGTCTTTGATCG
CAGGAGGAGAAATGTCATGGTCACTGCTATTATGATATCTACGACTGTCGCTATGATGA
GACTAGCAGCACTGACTGGTCGGTTCATGGATGCTTGCGCCGCGTCGACGTTTGTCAT
GATAATGCGTAATATGATTCCGCCTGTCATACCGAGAACCATCTGAGCGAGTGACAGGA
GAGCAATCACGCCGACCAAGATCCTCGAGGAGGAAATGATCCAAATCCTCCAGGCAA
AGTATATCTGGACGACAAAGGAGACGACGGCGGACATGATAGCCACACTAAACCAAGT
GTTGTGGTACCCCGTTACGCTGCTGATATCGCCGTAGTGAGTGACGTTGATGTCGAATC
CGCCCTGGGTGACGAGAATTGTTTGGACCCATTCCCATATGAGGATGCCGTAGACCATG
AGTTTTATGATGATGCGGTCTTTAGGAAAGTACAAGTGGTAGATGTAAACTTGCACGCT
GAGAACGCCTTGGAGAGCCCATGAGAAGAGGACACCAAACATCTGTGGACCTGCAAT
CAGCGCAAAGTTGGGAGGTAACTGTGGTATACATGGTGCTACAGGTGCTGGCGAAGCC
ATGACGTTGACGCGCAGAGTTGGGGG

>c9756_g1

ATAATATCTATCATGGTTGCTGCATCGCTATTCCGTGACGCAGTGGTTCCAAGTAAAACC
CGATACTAAGCGCCTGCGGGATTTGACGTGCTTTCGGTGAATCACGGCGTTTTGCGACC

AGCCGCTCTCGCTCTCTTGTGCACACTCCCTTGACTCCATTGCCGACCTTGCATTCCCT
GCGGCCACCTTTGCAGACCGTTCAGTTATGTCTGTCAACCGGTCCGAGCGCGCATACG
CGTCTCGCTGTCCATCTTTAGCCTCTCATTCTCCGAGTTGTAGTTGTGTTGTACTGGTAC
ACTCAACTCGGAGGACGTCCGGAATCGGCGAAACAGGGACCCGCGAGTCAGGGATTC
AGTCCGAGTAGTTGCTCAGCCTGACGGCATGATTCAACCGCAACCGAAGAAAACGGG
CTTACATGTGCACGCAAGGGTAGCACTTCCCCATAGCAGCACAGGCGCACGCCGTACT
TACGGGTTAGCTGAGCGCCGAGCGCCGAGCCCTTGTGTTGTACTTACGAGATCAGCAG
GCGTTTAGCTGCGAACTTGCTCTCGGCATGGGCATTTGTTGCCCGCCCGCGGGTCGCTC
CCGCCCGACAAGCGACCTGAGCTATGATGCGTGTGCTCCCCTTGTCGTGTTTCTTGTTG
GCGTTTTGCATGCTCGTGCTCGTGCACTAGACGTCGGCGGCCTGGCTAGTATTCGGATT
TGTATTGCTTGCGTTTACGTTCACGGCCGCCGCTGCGATCCTGACCAATGACCCCCTATA
GAACTCGTAGCCCCCTCACTCACGCAAGCTGTTCACTGGCAACCGCTCATTCGGCGGG
CGTAGTGTGTTCATGACTGCGGCAGCAAACCATGGATATGGCAATTCAATTCCAGATTG
CTGACACGCTTGGAATATCTGATATTCTGCGCTACGTGATCTATCGTAAGCTCGTGACAT
GATCAGAGTACATTACATGTGCGTCGTGTGTTAGTTCTTGCGGAATGTAGTGCTTACGTT
GTGCCGGAAGGGCTCAGCAGAGCATCATCAACTATGACTATGTTGTGTGTATCCGCGGA
TGTCCGTGAGAACCCCTGTCGAGATCGGTCACCTTGGACGGTAGCCGACGTCGATGTC
TGCTGGCCAGCTCAACACGCTGCTTCACAGAAGCCCCGGGTCACTCACTCTCGAGGAC
CCGCGAGTTACCGTTATCTAGTACGCCGGGTTTTTCGGTGCACTTCTGTGCGCTTCTTGT
AGAGCTCAATCAAACATCGCGGTGGGCCGTCATAAAGTTCAACTTTGTTGTTTTTTCAC
AACTCCCGCGTTCTGGCAAGACATGTGTGAGTGTGTTTGTGTTTTGTTTGTGTTTGTGT
TTGCGGTTCCGTGTAGAGCGTGGCGTGTTCGCTATCGGGGGACATATGCCTCGGTTTCG
TTCAAAGAGCTGCATATGCATGTGCATACGACGGTGTAAATATATCCGTATCTTTGTATC
GCTCACCCCACCAATGCGGGGTCTGGGTGTAAGGACGTTGGTTGGTCTCTCAGGTTGC
ACATATATAGCTACGATGGAGGAAGACGAATGGATATGGATGATGCATTCCACGTGAGC
GAGTGCATCATTATGTGACTACTAATAATACCCGCAATAGATCGCAATGTTTTTCTGTTG
CAATCCTGGTTGCTCACCGAGTCGTGACAGGAGTCCACGGCTTTCTTTGTGTGTTTGA
ATGGAATATAAATATACGACGAACTGCGGTACGTGTGCCCCTTCTGGATGCATTTTTCCA
TAAAACGTTCCAGGTATCAAATCAGAGTCTCTCCGTATGCCTGTCTTCAATGGCGAGCG
CTCAGCTTGCCTTTTTCAAGAAGGAAGACTTGAAGTACCACACCCCCGACTATGCCTC
CAGAGCCCGGAATCTTTCACCTTCGACCTTCATTTGTTGCATTCCGATGGATTTTGATAT
GTTCTCCGTATTGTTTCTTTTGTTTCTTAACATGTACGCGCAGCTGCGCTGCCCTCAGGC
TAATGCTAGAATGTATCATGATTGTCCATGGTTGTTATCCATTTTCCAGACTCTCCTCCAG
ACACCATGCTATCGCTCAGAATATAATATGTATAGATAGACACTTGGGTGTGGATCTCTT
GACGAATTATCGCCGTCGCTGAACAGAACACTATCTCGCTCGGTTCGATCTAGCCTCAC
CCTCCGAAACAATATGCTCCCCGGACCAGTCCTCCTTGGCATTCTATTGCCAATCCTTCC
CCTCGTATCAGGTCATGGAGCCCTCATGTCAGTGCAAATCGATGGGAAGACGTACTCTG
GCGTGAATCCTTTCGATCCTACGGGTGATCCTCATTCTAGTGAGTCATGCCCAACGTCT
ATCTTGTGATTACGCCACGTGGCATCAATTAATCCTCATAACCTCCGTTCCCTTTGCGTT
TGGCACGTACTGGCACTGGCAGAAAACACACCTATTCGAGTGAGAGGGGATGGAGAA
AACGGACCTATAACCGACGCAACAAGCCCGGACCTGCAGTGCGGGATAAAAGCTTCC
CCCGCGCCGTTCGTGGCGCCCGCGAACTCCGGAAGCGAGCTGGTACTCTACTGGGCAT
CTGGCGACGGCAGCACCTGGGTGCACGACACTGGTCCTATGCTCGCCTATCTTGGCGA
ATGTGGCGAGACGAGCTGCGACAAGTATCAGACTAGTGCAAAGACGCAGTGGGCGAA

AATCGCGAACGAAGGGGCTGACCCCCAAAATCCACAGCATTGGCTTCAGGCGCGACTT
AACAAGAATCAGACAGTCAGTGTGAGCCTCCCGCAGAACGTCAAGCCTGGCAACTAT
ATCTTCAGGCACGAGGTGATCGCTATGCAGAATCCAGATGCGGAATTCTATGTGGGTTG
TGTGCAGCTCAAGATTGGGGGCAACGGCAGCGGCGAACCCAACACCACCGTCACCTT
CCCGGGGGCATATAACGACGCTGCCAGTCAGACTGCGCTCAATCATACTGATGTGCGTT
TCATATCCCCTCGCGATCTCTGTTCCGCCATGGCGTCACCTCGAGACTCACGCTGAAGC
ACGAAATGTGAATTTGTGCGCACGCAGGTATACACCAACTTTGATATCTCACAATACCA
TTTTCCGGGGGGGCCTGTCGCCGAGTTTGGGGGAGCTAATACTAGCACTCCCGGAAGC
TCTGGCTCTGGCAATATGTCCGCCTCCGTTTCTCCGACTCCACCTGCGAATACGTCGAG
CTCAATCGCCCCCACTACGGTTTCTTCCGCATCTGAGAGCTCGCCTACCGGTGGTAGCG
ACGGCCGTTGTCCGAGTAGTACTAATAATCGCCGTAAAATTCAGGAAAAGCGACCCGG
TGCTTTCTGATGGAGCACATTTTCTTTCATTTTCATGCATAGAATGCATGTATGTACATGT
ACATACTTTAATGCTGGACACGGCGCGCAGGCGCACACAGATCTGGTAATTTGATAATT
GCAGGCTTGATAATAGGTCTTAGGTCTACATTTACATTTACTCGTCTCTCCGACCACGAA
CATTTGCATTTGTGTCGAATTTTAAGAGATCGATCAAAGCGGCGCTGTGTTGAGCCTCA
TCTGGAGGTTCTTGATCGTGATGTCTGCGAGAAGCTGGACCTGGGAAATCGCAAAGAG
AGGACGGAGGGAGAACGACAAGACTGCTGCTGAGAGAGAGTCGAGACCATACGTGG
TCAACGGGACGTCGATGGACACGTCTTCCTCTGGGAGATCCAGCACGCTACAGACGGT
GCGCAGGATGAACGTTTCTCCATCCGTTTCTGGGGGGGCGACTGCTTCAGCGTCCGCA
GTCGGATCGACAGGGACAAGATGATCGTAGAGCGGCGACGGGCCTACGCTGTCGCGG
ATCAAGCGCCAGTCGAAGTTGGGGACGTATTGCCAGACTGGTCCGTCGCGAAGCTTCA
GGATCCCGTCACCGATGTAGTCGCACAGCTCTCTGCCAGTCATTCCCCACCTAGTCAGG
CGTCGACTGATCTTCGATTCGCCGCCTGGGATTACAAATCGACTATCATGTATAGCAGGC
GAGACGACAGAAAACGCGTTCTTGTATCTGCGTGTCAGCCCTGCCAATGCGGAATTTG
CCGCTGCATAGTTTGTCTGGCCTGCGTTCCCAAACAGGCCGGACACAGACGAGAACGT
AATGAAGAAATCTAGAGATTGAATGTCTATCACCTTCTCGAGTGCGAAGAAAGCCTCG
ATCTTCGGGGGAAACGATAGTTCGAATGTATCCTTGGTGTGCGACGAAAACATAGCGTC
TCCTAGGACCGCGGCCATCAAGACACAACCTGCCAGTGGTCGCCTGAGCGCGTTGACC
ACCGTGGCCATCTCCTGTGGAGATGTAGCATCGGCTGTGATAGTCTGTATAGACAGATC
CCAGCGGCTCTCGAGGTAGGTCAAAATCCGGTGAGCGATGTAGTCGCCATGACGATCC
AAGTTCTCGACGCCTGACCTTGAGGTGAGAACAATATCCCTGGCTCCATTCTCGTACAT
CCACAACGACATGTGCAGGCCCAGACTGCCGATGCCACCGACCAGAAGATACGACTTT
GTCGGATCGAACAGGTTGGAAGAGGGCGGCGCGCCACGCGGCAAGCCAGAGAGCAA
CTCCGACGGAGACGAGTACGCGACGGATAGTGGTCCGCGATAGCCTACTGCAGCGCGT
ACTGCGTCTCCCAACGCCCACGGATCGCGCGCCGTCAACTTCGCCAAGCCATCCTCAG
GATGGTTCCACAGCAGCAGCCTTCCGTGCGGAGCCAGGATGGTGCGCAGGACAGAGG
CCTGATGTGGATCGCGAGTGCCGGACAATATATAGGACGGGCTCTTGAGGAAGCACTG
TTCGAGGTCGGAATTCGTGAGAGACGACGACGACGAAGCAAGCACTACCATATGGAA
GTCCTCGCAGAGACGCTGGAGCTGAGTTCGCAACATGTCGTCTGTCTCGACGATCAGA
ACTCGCTCGCCGCGCAAGCGCTTTGGATTCGCGACTGCAGGTGGACCGACCGCCAAC
GCGACGAGTGTCAACGCCAAAACGGGGATACCAGAGGAGTCGTCGCGAAGCACCTCG
GCTTCGGCCAGAGAACCTTCATGGACGACCAAATGGCTGCTGATAGGTCCCGTAGAAA
CCCCTACAACCGGCCCTGATAGCCCCTTGACAGTACCCACGAACACCCAATGAGAGCC
GTGGCGAGATGCGACACGGCTCACGCTGACAACAGAATGGTCTGTGGGTGCGCGCAG

GGGCTGTAGTGGGGCCACCTTGCCATTGTCAATCTTCCAGGATGAACCGACATCATCTA
ATGCAACACGAGGAGTCGGCGCAGGTGAAAGCTCGACGCGCGGGGCGAACACGGTA
CCATCCGCGTGAACGCGCATCTCCACTTCACCAGCATGCAGTGTCAGGAGTTCCCGCG
CCGCCTGCATTCGCTGAACCTGCGTGAAGCTGGAATCAAGAACGGCGAGTTGGATGTT
CCACGCGGGGAATTCTTTACGCAGAGATCGAGTGAAGCCTTGAGATGCGTCGCCGTGC
AAAGTGTTGTCTTCCGCAATGAAGAACAGAGACAGAGGCTGGAGAGGGTCGCGGGAC
GCAAGCTGTTGTTGTATCTTCATCTCCTCCCCTCGCACGTATTCTATGATCAGCGTGGTA
GTGTCTGAGGCGTGGGCATGTCCGGATACTACACCATTGGGTTGTGACGTTTGCACTTG
GTGGTTGGAAGCGCTACTGGGAGGCGTAGTTGAGTCGATGGGACGATACACGACATCG
AAGCGCTTCTCCAAAGGCTTCATGCGATACCCATGGAGGACCAGCTTCACCTCTTCCA
ACACGCCGATGAGAACGCCTGTGTCGTCACTTATGGTGAAGTTGTACGTTAATGTTTCT
GGAGTCCAGCTTACGAAAGTGGCATGACAGAAGATGGTTGAAGGGAAACTTCGACCC
ACAAATCCTGGACAGAGTCGGAAGGCTGCCACGCTTTCTGGTAGATGATACAAGCTCG
GATCGTGATTTCCTGTGATACTTGGGTGTAAAGCAACGTGAGTTGCTGCGTCGAGTATA
GCAGGGTGGATGCGATAGTCCGAAACGTTTGGGATATCTGTATCGTTTGCTCGGACTTC
CACGAGAGCCTCAACGGCGCCATGTACACCTGCCGCGATGTAGCATCGACGTATGCGA
CGATACATTGGTCCGTAATTTGCAAACGAAGACATTCCGGAGTATATTTCGCTCACGTCT
ACTGGCCTCAACCGCGTCCGGATTGCATCTATGTCCAAATTACGCAGTCCTCTGTCTTG
AATTGGCTCCGTTGAGAGAAACCCAGTCGCGTGCAGGCGGTCGTACTGAATTGGGAAC
GTTGTGCTCGCAGTGGCACTTCTAACACTCCAGCGAGGGCCGTCCAATTGGACTTGGA
CCGGTACTGGGCGTTCCGCAGACAAGGAGAGAAGGCTATGGAATCGCACATTGTAGAC
TTCATTTGCACCGCATTCGAGAGCCATCTCTATGAAGCCTGCGGCGGACATGATGGGCT
CTCCCTTGATCACATGGTCGGAAAGGCCTGGATGGGTCCGCGTGTTGATGCGAAGTTG
GGGATAGTTCAAAGGGCCATTACGGTCTTGACGCTGTCGCGCAATCTCTGCGGTCGGC
GCATACCAAGGAACAGACTTCATCGCGAATGGGTAGTCAGGAAGCCGCCCACTGAATA
CCGCCGACGTGCCATAGAGAACGTCAAAATCGACACAGTTGTGGCCAGCCGCAACCA
CCTTTCCGAGCGTGGTGACGAATTCGTAGACTTCCACTCCAGGTTCGGGCTGGCGCGG
ACGACGAAGCGGACAGGCTACTACAACGCCGTCACGGCCCTCCGTCATGAACTGGATA
TAACTGGCGAGTGCGGGGTGTGGTCCAATTTCGATGAACGTCGGCGACTTGTACTTTG
AGCACAAGGCCTGTATAGCCTCCGCGAATCGTACTGGCCCAAGCGTGCCGTCCCAGTA
ATACTTCGCATCGAACGGGCCAGTGAACATCTGTCCGGTGACAGTCGAGTACGTCTCC
ACTGAGGGCGCTGAAATCTCATGACGAGCGAATACCTCTGCAACGAGCCGATCGTACT
CCGCGTGACAGAGCTCCATCATCGCACTATGCACAGGCACGCGCGTACGCAGCTTCCT
GGCAAAGACGCCAGCTGCGCCCGCTTTCGCGACAGCCAAGTCAATGTACGTCTCCGCG
CCCGAAAGCGTGACCGCGCCGGGAGAGTTGTAGCACCCGATCGTGAGGATGCCGTTC
CCGAGCTCCGCATTGACTTCGTCGATGATCTTCTGCGCGTCCTCTGGAGAGCATGACAC
TGCGGCCATAGTCCCTTTTGCGTCCTCGAGGAGCGACAACGCGCGGCCGCGCAAGATG
GCAAGCTCGAGCGCAGCAGCTTTCGACGACGCG
>c9487_g1
CTTGTATCACTCATTGTAAATGCTGAACACCCGTGAATTTAAGCTGTTGTGGCATACAGT
GAAACTACTATGCGGCCTCCCCACCATTTGCTCTCTATATACTTGTGAGAGATGGGCTAC
TCACCCTTGCAGCCATTCCATCCGCAACTCCATTGGAGTCTTGACTGGTACCCTGTACC
CCTACCCAGGCACTCATTGATAACTCCACCTGCCGCGACTTCCAATCGTTCATCGCGAC
ATCGCTATCCAATTGAATCACATCCGAGCAGCTCGTGCAGTATTGCCATGAGATAAAGG

TGAGCGCAATATGTGGCGATCATCGCCGGAGCCTCGCTGGCTACGTTGCTGTCTTCCAG
TCCACTACAAGTGCAAAAATTGTCAATATGATGCCCCATCTTTGTCGTCTGGAAGAATT
GCGCAGCATGTACGTGCGACCGAATGTCGTAATATATTGACAGACCCGGACACTCGCTC
TCAACTTGCAACTCTCCTCTAACCTACTACATCGACCATCTCGCTGAGGGTGACTCCGG
CTTGATCAAGTGGGTGCTTGCGGATTGTAATGGATCTTTGCGCAGCTGCCATATGTCAG
GCTGCTGCAACGTATAAACCAGATGGAGCGCAACCATATCAGATGATTATACTTGACGT
ATACCTCACAGGTCGATGGAACGAGTATGCGCAGCTTGCGAATCGAAACAGTGGTTTT
TTATACAGCTGACACTGCCCAAGTCCGGCGACATGCACCTCGAGTTGAACTCGGTTCC
GGACGCGTATACTTGTCCACTATTCTGACCCTGACTCCCCAGCCGAGACGACTCCCCGA
CTGCCAATGGAGACCATCGAAACAGCCATCGACGAAAAGCTGAGGGAGACCTTTGAC
TTGCCAAGTACTTCTGCCTCGTTGCCGCCTCCGTCTCTGTCGCTCTGTCAAGGTGCAAC
TCGACTCGGTGCGCCAACTGTCTATTCTTCATTCCCACCTGACAACGGTCATTCATTTTG
AATGACCTACATCATGCGTCATGCCTGTTATAGGCCTCGAACTCACGCGGGTCAGCGAC
ACTTGTCCCTCTCTTTAGTGATACCAACCTACGCCATTGGACGATGCATTGTCTTTCAT
CACATGACACTCGCCGTCCTTCAAGGGGAGTCCATGTATCGAGGACCTGACTCTACCG
CTACGACGCCTCGAAACCCTATCTTGATGCGGACTGTCACGACTGTTAGGAGTCGAGG
AGAGCCGTGAATAGTGCAGATGCCGGTGTCCGGTGTATGCGCTATTCACGAATCGCCTT
TGAGTCGACGAAGATCTGATTGTGGAACACAACATCTTCTTCTTCTTCCGTTACAAGTA
GATTTCAGATGCACTATATATGAATAGAATAGATTTAATGTCCCCATTCTGTCGCAAAATA
TGGGACACCTGATACACAGGACGGCAGCTGCTATCGAGGCTGGGGGGACCACAACGG
GACATACGTAGTACACGCGGCATGCTTCTCACCTGATTGAAAGTACCTCGCATCCCCCG
CATCTGACAAGCTGGTGACACTACGGCTATCTTGAGGCCCGAGTACAAGTGGCTCCTA
CCCCATGAGCTGTCAGACCAGCTGTTTGAGTACTGGGCATGCTGCCTCGGGCTCCTTGT
GCGCACTTGCTCAGCACTGAATACCTCCTAGTCTCGGCAAGCCTTTCGGTTGTATCGTT
TCAACAGAGGTTCTGCACTATACATCCGATGAAGAGGGGACAAGGTTCTGTGGGGAAG
TTCAGGCACGTACTACTTGTGACATCGCAGAACATACACACGACAGGCAGCACCAGGG
ATTTCAAGGGCAAAATCCCAGTAAAGGCCTGCCAAGATCTCCTCGAAAAAAGGCGGG
CGCTGCCTGTCGCGATACATCATCATAAGTTCATTACTGTACACCGGAGATCTTTTATAA
AGCTGCCCTATAAGCTATCAGTGTCCGCAATCACTCAGAAGGTATACGTTCCGCCCGAT
TTGTAATGGTGCAGCGGTACGATAGCCTGTGAATGACCGGGCTCATTTGAACTTGGACT
GGTTTGCCACAAAAGGCATATCAACGCTCCCACCCTGACCCCTCATTATTAATCTCGCT
CGGGCAGCGCATCTGCATAGTATGTAAAGCTTGAAGGATGACTGAACCTCCTGTCAAA
AGCCAGATCAATACCAAGGGTAATACATCAATCTCCCAGTCCGCATGCCGGTATCACTG
CTAGCGGTGACCCCCAGTTTGCCTGTCGACATGTTGCCTTCGCCCTTGAATTGGGACTC
CTCCAGCCTGTCACGTCGAGAAGGCTCGATAGAAACGAGAACCATTTGTTCCGACAGG
TAAGCAGTCAGGACGGTTGACTACAGTCGCACCCTATACGTGATATGCAACAGGTTGC
CTATGCCTCGATCCGCTTGGCAACTCCGGCGCGTCCTCCTCCAGACGCACACGATACGG
CTTTGCTGACGGCCTCGTTCTGGTGATATCAATATTAATATTTTTATTTTCTCAGGTCGGC
AGACGCCTCAGGTCTGACACCGCCGCGTCGCCGCCTTTTACTGGCAGAAGCTACGCAG
GCATCAGAACATTTGACCAGTCGGCGGCCCCCGCTGCGCACCACAGTAGAGGTATTTT
CACAGTCGTGATACAATACAAACCAAATTGCGAAAACATCATTACCATGCGAGTGTGAT
GCCTGCTAACCCCGCGCGCCCGGGTGCGTTTGCGCCGCCGTCACGGAACGCCTTCCGC
AAGATAAGCTCTCACCAGAAGCACGAAGTCAGAAACGCACCCGGGGTCGGGACGCGC
ACTCCGTTCGTCCCAACCCGGCAAGTCCAATCCCCGTCTATCGTAGAGTTCTCAGACGG

CTGTGCATTGTCATTGAGTGGGTGTGGAGAGCGCAAGCTGCGTTCGAGCGCTCATGCT
CGTCACAGAGAGTCCCGTCCATCAGAAGTTCGATCGCGGCACCTCGGGCCTCGAAAAC
CGAAAGTGGCGGACGACAAGGAAACTTCCGGCTTATTTGTACGAAGTAGCCCCCGTGG
GGAATCGTTGTCCCGTAAGCGGTGAGGAGAAGAAGGTCGAGGTGTTTGAAAGGCCAC
AGAGAGTGCAAGGCTGGAAGAAACGAGGCGAAACGACAGATGGACTAGGGAAGGGC
GCATTCCCTTGCTGGCACGTAGTATCCGGAGGTGCGGACTGCACACAGGGCGTGGGGG
TCGTGGGGCCTACCTCTGTGTTCTTGACCTCGACGCTGACAGAAACGACCTCGCACG
GTGCCGTGACACATGTGAGGTGCAAAGAAGCACGAATCGTCATAACTGAGGGCATTAA
TTATACAGAACAGGCAACTGGATTTGTATCAGGGGATGCCACGCAAATTTATACCTGAG
CGAGGACGATCCCGGTGCAAGGCGACGAACAGCACACAGCCCGCTGCGATAAGGGAA
GTTACCGAAAGGAAGCAGCAGCATACGAAAAACGCGTAATTACAGTTACCTCACGGTT
CAATGCGACTTCGCAGCCATGCTGGCATACTTGGCAGCCGGCCTCATCGCAGCCTGAC
CTTTTTTGTTCCGGTAGAAGAGGACGTTCCCGCTGTCTTCTACGAATTGGCAGTTGTTG
TATGACTCCCACAGCGCGCCGAAGATCGGTTTATTAGAGTGACGATAGTGGCCGCCAAT
AAAGGCCTTGAGATACTGGGTTGCCTCAGCACAATGGTAGAATGGCATCTTCGGGAAA
AAATGATGTACGACATGATAATGCGCGACGTCGTGCAGGAAGAAGCGGCCCATCCAGC
CCAGGAAGTCACGGTCCACCGTCGCGGCCGCACCCCTCTGGAAGTTCCATTCCGGCTG
GCGATAGTGCGGCAGGTCGGGTGCCGTGTGGTGCAGGTACGTGATCATGATGAACCAA
TGAGTAACGCACAACCAGGGAATGCCGTAAAACTTGATGACGGCGGCAACTCCCCAA
GTCTTGCAAGCAAATCGCACGAGAGCAATCATCATGAGGATGCCGGCATTCGATACCA
GAACACTATTGCGTTGTTCCTTCGTGAATAGAACGGAATTCGGGTCGAAATGATTTGTC
CACTTGGGATACGTCTTTTGGCCGGAGACGTTAAAGAGAAGGTACGTGGGGAAGGCC
AAGAGTTGCTGGCGCACGAGCATGTACATGGTGTATATCGGAGTGTCGCCAAAGTACT
CATCGTAATCAATCTCCCCTGGCGGAATACCAAGATCGCTCCGAGTCTTAGGTACGTAC
ACCTCATCGCGCTCCATGGAAGCATGATTGCAATGGTGACGGTGGTGAGAAATCTTCC
AGCTGAAATACGGCGTCCACAGCAGGGTATGAAGTATAAAGCCTATCAGGTCGCAGAC
ATAGGTACTGGCCGAAAAGGCGCCATGACCGCATTCGTGGCCGATTACCCATATGCCGG
TGAAGATCAGCCCTTGGAACCACCAACTGAGAGAGCTACAGTCAACTCGACGGCGCG
AGAAGTGATATTTTATACCCACTATACTCCCCAGCAGGCCCATCGGGCAACGTTTGCGC
CAAAGGGAGACAGAAGATGCACGACTGCACGGCTTTTGAAGAGGGGGTCAATGGCGT
AACCCATCCTCCAGCAGACTGCGGCCATGAGCAGGTCGAGGACAAGCCATGCCAAGC
CTCTGCGGGTATCGCGGATGAATAATCGCGGAGGTATGGCGGCTCGAATGTCCTTCAGC
TTCCTGTGCAGACAATCATTAGAGGTTGCCGCTAGTAGACCAGAACGATCTCACAGTG
ACATGGGGGTGAATTCGGGCAGATTCTCTTCGGAATAGGACTTTGACGTCTTCAATTCC
ACCTCAGACGCGGGGCCAGTCGGGCTGCTATCGCTGACGGACTCGAGCATGTCGGAG
AGAAGATGGGAACTGGGCAAGCAGCAGAACGCAGGGGTTGCGAGGGAGGGCGAGGA
GCGTTGTGACTTGAGCCAGAGGGGAATTTATCTATCTTGGACGAGGCGAGATCTCCGG
CCCCGCCCATCTCAAGTGCCGCGCAATGAATGAAGGATTCACAAAAATAATTGATTGCA
CGTGCGCGAGGGGAAGATGGCAGCAAGCGCAAGCGCAGCGCTATGCGGGTGTGACGA
GACGACCGGGGAGATGGCGTGACCGCGTCCATCAAATTTATCGCTGAGACCAGGGGA
GAGTTCTGGCGTGGAAACGACATCCGTAACGAGATTTGTCGTAAACGTTTGCTGACTT
GTCACGCTTTGGGAAAACTCGCATCGTCCCTGACTTGCTGCTTGTTTGTACTCTTCTTG
CGGGGCAGATGCCTAGATCCCAAACAGGATGACCGTTCTGTACGAGATGTGTGGCGAA
AGTAGTTATATGGGGACACGTGTGGCCTAGAGAAAAACTTACAGTTCATGGCACGTCT

CGCAGCCTGACTACGCGCCGATCTTCCTCCTTTGAAGTTAGTTGCCGTTCATCAAGACC
TTTATTCATGTAAGCGAGACCCGGGCGAGACAGCGATAAAAGACAAAAGACACAGCC
CGGGCCTCGTGAAGTCGATCGGAGCAACTGTTTGAGTGTTTGAGTGTTTGAGTGATGG
TGATGGT

>c7390_g2

GCCCTCCTTTGAGCGTTCTCCACTTGGAAGCTAAAGATTTGCGACGTTCAACGTTCTCA
GACTCACAGATTCATCGTTACAATAATAGTGATCATCGCTGGGCTCTTTCGCAACGGCT
TTACCGTTTTCAAGTCCACATGGAACCACGCTTTGAGTTGACGATAAACGGCAGAACG
CCTCCTGATTCGCTTGAAGTCGTCCTGCTAGAGTGGAATATTTCAGAGCCGCACAATCT
GGGCGAAGGAGCTACCACTAGGCGTCGCTTCCTCCGCAGTGGGAATACAAAGACAAG
AAGAACAAAGAAAAACTGCTCTCTTATGGATTCTTGTCCGCTCAAATGGTACCCTTGGT
TCTTGTCAGGGGCACGACTCACAGCGTTCGTCTCTCTCTTCCCGTCGCCGCTCACCACT
TTCCAACGAGGATGCGACTCGAGATTGTACACAAGATGGAACTTCACGCGCCTTCCCT
GCGTTTACAACAATGGTACGGACCGCTGTCGAATCTCGCTGGATCACATCGCGGTAGTG
TGTCTGAATGCATGTCATGCAGGCACGATCCCTCCTGACGCAATTGGCATACTAATAAC
ATTGCAGTTGAGTCACGCAAACGTTCATCTGGCCTTATGCTGCAATCCTGTTGCGTCCC
TCAGGTAGCATTGCTGAACGCTACGCGAAAGGGTAACAGCCGTATTCACTCTTGGCCA
CGACGTCGATCGCAAACGGGACAGTCGTACTCTTGTTACCGTTTATTGCCTGCCGCCGG
GTAGGTAGCCTGGGTTTGATGTGTCTGAAGAATATTTAAGAAGTGCATGATACCAGTCC
GAGTCCTTGAGTTCGAGTTCGACTTCCTCGCATAGCGGCCCGTTCACTATATGCATCTA
GCGACTTGACTTCCGCTTCTCTGGAATCGGAGACCGCAGCTGGTTGGCGACTTCAGTG
CTCGATGCTCTCACAAGCTCTTGTGGTTTAATTTGCGTTGGATCACGGACAAAGTGATG
ACGTGTTCGGCTTCTGACTTAGCGTGTATCCACCTGCTCCGCTGACCATGGAAGCCATT
TGGTTGTGCACCATCTCACTGATGAATAACCGCGAATCCGAAGGGCGCGAATGGCCCC
ACCAGATACATTCACCGTTATAACTTCCCGAGCCTTTCGATGCGGTATTCGTCATCGGAT
AACCGACCTCATTGATGGCGGCCTGCTGCGCTGCCATTTGTGTGGGCGTGAGTTCTCTG
AAGATAGCTCAGGCTCTCACTTAGTACTCCATTCTGTGGGTTTCTAGTCTATCCTCGACA
TTTTCCGGACACACCGAAGGACAAACTTCAGCTATGCGGAGGCGTACTTCCATTCAGG
CGCGCAACCGCCCTCAACGCACTGTGGGGGCCTTTAAGTTCAACTTCGTACGCCGCAC
CTTTCCCGGTTGTCGCGATTCGTTTGAATGCACCCTTATATGTCTCCTGTGCAATAGACG
TCACTTTACCTTCGTTGTAAACTCTCCGCCCACGCACCCAAGTATCCGAGGTACTCAGA
AGTCTGCGACTTCTACACCGACGCGCCGGCGCCGAAGATGGGAGTATGTCAGAGTGAT
AGATATCCCGAGTAACAATGTCTTCCCCAGCCAGGTGCCATAAATGTAGTACCCATTAC
AAAAACGCTGCCATGGGCTCCCTAGACAAGGGAGCGTACATATTCCAGCTCATGTCGT
CCGGTGGGTATAAATCGTGAGCTTCTCCTCTCTCGGTATCACCAGCCTTACCAAGTTCC
AACCCACACATCGTCTTTCACTTGTCTTTTCTTCACCAACATCGTCAGCATGTTTGCCA
AGCTCGCCATCTACAAAGTCGCGGTCTTCTCCGTCCTTGCGGTCGCCACCCCTCTGCCA
TCCGGCGGCGCGGGCTCCTGCAGCACCGGCACTCTCCAGTGTTGCTAGCATACCCTGT
CGTACCCCGACGCCGTCAATAATGGGATCGTGTCTCTGTTCGATCTCGACGTCTCCAAC
ATTGCTGCCTATGTTGCCACCGACTGCACCCCCATCAACGTAATCGCAGTCTCCTCTGG
TTCCTCTTGCCAAGCGCACGCCGTCTGCTGCGACAGCAAGATAACTGATCGCAGGCGA
CCTTGTCAGCGTCGGCTGCATCCCTGTCTTTCTTTAAGAACCTCCAGAGCGCGCAATCA
CACATAACCACCGGTCGCACATGCATCTTGGCGACATGAGGTAGATAGGATATATGGGA
TAGCCATGCGTGATGTGCAACTGGCTGTGAACAGAACTTTATTATTTGTTAGTTGTTATT

GAACGAATGCGTCGATTCTCAAAGGCCAAGTTAACACTTTGACACTTTGAAGCTTTGA
CACTTTGAAGCTGAAGGGTTGAAGTACTGTCTGTCTTCTTTAGGGCTTCCTGCTCCAGC
AGTCTACTGGACCTATCACAACAATCTCATCTCTCAGACTGAACAATTCTGGAATGAGC
GAAAATGGCGAAGAAGAAAGGCAAGGAGACACCGAAAGTCTCTGTGAAAGCTGCTA
AAAAGGTGAAGACGGTGCAAAAAGTGGAACGCAAGGAGAAGAAGAAGGTAACCAA
GAGCATGGACGACTTCGAGGACGACGACCAGGATCTGGAGAGCATCCTTGACAAGAT
ACAAAGAGAATGGGAGGAGGCACATAAAGTTACGGAAGAGCTTGTTGAGGGCCCTCC
TAGCCGTCGAGCAAACGCGACCCTCACCCCTTGTCCGAATGGGAACCATCTGTGGTGT
ATCGGGGGAGAATTCTTCAGCGAGGATGGTAAGGCGTACTTCTATAGCGATGTCTACCG
ATATTCTCCTGAGAAGAATGAATGGCGGAACTTTGTCTCCCCGACGTGTCCCGGTCCCC
GTTCTGCTCATGCCGTGGTAGCGAGTCCTGCCGGAGGCGGCAAGCTTTATCTGTTTGGC
GGGGAATTCTCGTCGTTATACCAAAATTCGTTCCATCACTACCGCGACTTCTGGTGCTT
CGACGTCTCGACGCATACATGGGAGCGAATCGAGACCAAAATTCGCCCGACAGCACG
ATCAGGCCACCGAATGGCAATGTGGAAACATTACATTGTGCTCTTTGGCGGGTTCTACG
ACCCCGGCATCAAAACGAACTACTTGAATGATTTGTGGTTGTTCGACATACAAGAGTAT
AAGTGGAAGCAGATTGAATTCAAAGATGCAGATCGCAAACCATCGCCGAGAAGCGGT
TTCTCATTCCTGCCCACGCCCGAGGGCATCCTCCTTCATGGCGGATATTGCAAGGAGTA
CGTCAAAGGCAGCCGTCCCGTTGGAGTAATGCTAGACGACACCTGGTTCCTTCGCATG
ACTCTCAACACCGATTCCCCCACGCCCGGGAAGTCCGCCGCCGATCCGCTCACGCTGA
AGTGGGAACGCCGCAAGAAGACCTCCACGACCTATGCGCCCTCGCTCCGGTCTGGATG
CACAATGGCACTTTGGGCCGCGAAGAACATGGGGGTTCTCTTCGGCGGTGTCACGGAC
GAGGACACGAGCGAGGAGACGCTCGAGAGCGTGTTTCATAACGATCTGTACGGATACC
AGATAGCGGGGAACGGCCGGTGGGTATCAATCATGCTCAAGAAGCCCAAGAAGAAGG
GCGGGGCGCCGAAGAAGAAGCCGGCTATGGTGCAGCAGAGACGGAAGGAGCCCGAA
CTTGAGGAGGACGAGGATGCAGATGCGGATGAGGGGTCCGACGAAGGGGAGGGCGAT
CGGATCGAGGGCGACGATGATAAGGAGGCAGAGAAAGCATTGAGAAAGAACAAGAA
GACCAGTGTCTTACTCAACCAGACCATGGCTAGTCCGAATGAACCCCTGGGAGATGAC
ATAGACCCAGACGACCCGAGGCTGACCGTGCCGCTTCCGCGATACAACGCGATGCTCG
CGGTGCTCCGAAACACATTATACATATACGGCGGGATTTTTGAGAAAGGTTCGCGTGAG
TACACACTGGATGACTTCTATGTACTCCCGCTCGATAAGCTCGATCGATACGTCTGCCTC
AAGCAGTCCGAAATCGTAATTGGGGACGGCGACGATGCGAGCAGTAGCGACGAGGAT
GACGAATACGATGACGACGATGATAACGAGACGCAGCACGATGAGACGGAGAGCGTG
ACCGTGGTCTGTGAGGATGTGGACATCGCGGAAGAGCAGCAAGTTTGTACAGCGAAA
ATAGAGAGGCAGCGGCATGAACGATTGACTTTAATTGACAGGACTCCCTTCGCGTGCA
AGCGACGGCGTTTATGGGCGTCTCGAGGGATGCGACGCGGTCTCCAGAGGACGTGATC
AGCACACCGCTTCCGGGAGAGACATTGGCCACATTCTACGCACGCTCTCGTAAGTGAT
AGTAGGGCGAAGGTAGGCTATGACGCTGAGAGATGCACGAAACTTCATAGGCGAATAC
TGGACGCAAAAGGCGTTTGGAAGGAGCGACAACCGAGGCAAGATGCTACGGCGGGA
CGGGTTCACCTTCGCCGAAGAACGTTATGGTGACTTGCGCGTCCTAACGGCGAGAAGT
ATCGCTGACATGTTGAGTTGCCCATAGCAGAGTATAAACCTATCCTCGAAGAGGTCGAG
CGTATCCTTGCGGAGGCTGGGCTGGACGAGGAAGAAATGCGGAAGAATGCGGCAGCG
GGGCCCCGTAATGCGAGCGGGGAGAGTAGGAACCGTCGTTAAGTACATACGATGTGTA
TTGTTGCACACACCAAATGTACACACGAGCTGATATGCAGGGGTCCAAGAAACGGCAT
TGAAGACTGTCAGATTCCCAATCCGTCCAGCCACCGTTCGATCTGGAAAAA

>c7166_g1

CTTTTACGCGCAGTTTGCACTGTCCGTCGAGACACAGGTATACAGAACACACAAAACA
CAGCAACTTTGCGGCGCCAGGGAAAATTGGGCAAGCGACTGTAAATTATGCAATGCAA
TTAGTGTGTGTATAGCTCACCACTGGACATTGAGAGACGGTATGTACATACTACAAAGA
CAACTTTTGACACTAATACGCTATTACACAGGGCGTCGTGTATTACGGTCTACAAGTATG
AATATGGGGACAATAACCGGGGCTACCGTAGATGCGGACATGGGGGGGGGAAACATG
AGTGAGGTGAGGGGAGAGGGGAGGGGGGGCGTGATGACTGACGATGAACAGGAAAA
GATAGCAAGTACTTGATAGAGGAAGACGTATTCCGCAGATGCGGGTGGACAGAGAACG
CATCGCGTTGATGGTTTATGTACAAGAAAGATGGTGGACAGCAGCGAACACGAAAGAT
AGATGAGCAAAAGCCCCTCGCGTCAAACTCCGCACCGGCGGCCGTCCGTGTGTGTGT
GTGTGTGTGCGCGTGCGTGCGGTGTGTGGATGTCAAAGGGGGAAGAGGGGGTTACGT
TGGTTCGTCTTTCCACACAAAAGGCGGGCGGTTTCCGGACATCGAAAGAAAAAAAAC
GAGAACGAGAGAGCGTGATTAGGCTCAAACGCGCCGCTTGAAGACGCGCTGCCCAAA
CTCTACCTCCTGCTTGCCGAAGATATGGCGCTGTACGTACGGGTTGCGGAACGTGCCGG
CGGGGTCGACGGTCTCGAGAACTCGAATAAAGTCGTCAAAACGCGGGTAGAGCTTGC
GCAGATCATCGGGCCGAAGGGGGTGTGTCTTGGCCCAGTGAGGCTTTCCCCCGTGCCG
GATGAGAATCTCCTCGACGCGCGCGAACAATTTCCGGTAGGACACATTCATCCCGTAA
GGCCTGTGGCGCGGCACGTCCCGGCGTGGCGCAGGTGTAGGAACACACATGACGTGG
GAGTGAGAAGGTGGGCGAATGGGCGCATCGGAAGGTGGAAGGTGGGAACGCGCGAA
GGAGGAAGGCGTAACGGAGAGGAGGCATTGCGGACAAGAGCATAGCAGGTCCGAGG
CGATGCGGAAGATTGCGAAGGGAAGTCGGTGAAAGGCACGGGTAAGACCGAGAGCAT
GTGGAAATAAGCGATATTAACGGAGCGACGGCGCAGAAGGAGACACCGCGGCAAGGC
GCGAACCGCAGGCAGGCAGAGACAGACAAGGGAAGAAGATGGAGAAGGACAGATGA
ACAGTCAGAAGATGCTTGACGCTGCGGAGTTGAAAAAGCGAGCACTCACTTGTACTG
GATAAAGCCAATCCAACACGTCCGCTGGCCGTTGCTAGGACTAAGCCAGATGTCATCT
GCATCCGTAAACCTGATCTCGACAGGGAAGTGAGGCCGAAGACCGGAAGGGTCGGCT
TGCTCTTCATCCAACCACGCACGCAGTTCGCGTAGGCAGGCTTGTGTGCGCTCATACG
GTATGGCCCATTCGGTCGTATACTGCGGATACTTGCAGTCAACGTTGAAGATGCGATGG
CTGTCATCTACCGCGATGGTTTTCTGCGATCCGGCCAACCAAGCCGCAAACCTACCAAC
CCAGATATTGAAGTCCTTCACATAGCACCCAATGAAGAGCAGGAGCTGGAGGAAATGA
TATCCCACGAGGGAATGCCAAAGCCACGTCGCAACAGGTTTCTTGGCCTCGTGCGTCC
TACTTGCAGAAGCCACACGCACGGTGCCGGCTTGATGGAACCACCAGAAACGGACGT
GCTGTGCAGCGTGGACGTACGTGTCGAGGTTTCGCACGATTTCATCAAAGGTGTGTGT
CTCCGAGACCTCCTTGAGCCTGAAGGCAGGTTCGACTTCGAGGGTGACCTCGAGCAG
GAGTCCTGTGCTGCCGAGACCACATAGTGACGCCATGAAAAGATCGCTGTTCTCTTCA
CGAGAGCAGCGCACTCGGGACCCGTCTGCGAGCATGACTACGAGGGCGCGGACATAC
ATGGGGAGAACGGGATAAGTGATGCCTGAGGCATGTGTTGCAGTTGCGAGGACGCCA
CCAAGAGTCTGGTCAGAGATGGACCCGACATTGATCATGGCGAGTCCGTGTGCCTCGA
GGGCGACATGTAGATGCTTGAGTTTGATGCCAGGCTGTACGACAGCATAGCGTTTCTCA
TAGCTGACCTGTCGGGCAAGACAGAGTCAGAAGCGGGGCCAAGAGATACCAAGCGCC
AGCATGTGCCTGATTATGATTGCCCCATCAGGCGCAATCGTGGGCACGGAGATCCCCGA
GCGGCAATGGCCGAGATAGCAACTCACCTCGATAACTTTATCCAGTCTCTCGGTCCGCA
ACATGTAGCCGGACGTACACGCGAGGTCGCTCGGGCTGTGTCCTACTCCCGCAATGCG
CACCGTCTTCCCCTCTCGCCGCGCGAGCTCCAGGACCAGCTCACACTGGAACTCGGTC

TCTGGCTCGAACACACATAGTGGCGTGCACGTGAAGCTGCGCCCCCAGTTCGTGAAGG
AGGCTCGTGAAGTCCCGACAGGAACAGAGACAGACGCAAGAAGGTCGTAGAGTGCC
GGCAGGGGCAGGCAGGCCAGTTTGCTAGAAGATGCTTCGAGAGCAGACATGGTAGCC
GGAGTCCGAGGGGGGAGGTGTGTGGTTAGCATTTGCTGCCAGCGACGTCCGGGGCTTT
ATAAGTTTTAGCAAGTGCTGATTTCGCGATTTTGTCAACCCAGGAACCTGGCAGACCA
GCAAGACTCCCCCGATAATTATACCACCCATAAATGTCTGATGACTGCCCGGAGTTTTC
GTCAGCTATCGTCCGCACGGCCACGTCTATATGTGGTCTGCTACGCCACTTATGGGGCA
TCTGCAGCTCAAAGCGACCGAATATCCGCCGTTTTGGGCCGTTTTGGGCGCTGCCATAC
GGCGCTTGTGTATCGGACCTTGCCTCTTTCGACCGGTTGGCATGTTACTCATCTCTCGG
CCACTCCCGAGTGGCGGGAAATAGCGAAAAAGTCGATCATCATCTTGATGGCAAACGC
CGGTGGGATGGAGGCTGGTGAAAGGCGAGGCGAGGACAGAGCTCGGAGATGTGGTA
ACTTCGTAGCTCGCATTAATTGAAAGGAGATCTGAGTGTGGTGTTAGATTGTTACATGT
ACATGACACGTCGAGTCTGCACACCTGACTCGATTCGATATATTCATACTTGGAACGTT
TGAAGAGCCCTCGT

>c1758_g1

GCTAATTAAGCTATCGGGCCCATACCCCGAAAACGTTGGTTTAAATCCTTCCCGTACTA
ATAAATCCTATCACCCTTGCCATCATCTACTTCACAATCTTCTTAGGTCCTGTAATCACA
ATATCCAGCACCAACCTAATACTAATATGAGTAGGCCTGGAATTCAGCCTACTAGCAATT
ATCCCCATACTAATCAACAAAAAAAACCCACGATCAACTGAAGCAGCAACAAAATACT
TCGTCACACAAGCAACAGCCTCAATAATTATCCTCCTGGCCATCGTACTCAACTATAAA
CAACTAGGAACATGAATATTTCAACAACAAACAAACGGTCTTATCCTTAACATAACATT
AATAGCCCTATCCATAAAACTAGGCCTCGCCCCATTCCACTTCTGATTACCAGAAGTAA
CTCAAGGGATCCCACTGCACATAGGACTTATTCTTCTTACATGACAAAAAATTGCTCCC
CTATCAATTTTAATTCAAATTTACCCGCTACTCAACTCTACTATCATTTTAATACTAGCAA
TTACTTCTATTTTCATAGGGGCATGAGGAGGACTTAACCAAACACAAATACGAAAAATT
ATAGCCTATTCATCAATTGCCCACATAGGATGAATATTAGCAATTCTTCCTTACAACCCAT
CCCTCACTCTACTCAACCTCATAATCTATATTATTCTTACAGCCCCTATATTCATAGCACTT
ATACTAAATAACTCTATAACCATCAACTCAATCTCACTTCTATGAAATAAAACTCCAGCA
ATACTAACTATAATCTCACTGATATTACTATCCCTAGGAGGCCTTCCACCACTAACAGGA
TTCTTACCAAAATGAATTATCATCACAGAACTTATAAAAAACAACTGTCTAATTATAGCA
ACACTCATAGCAATAATAGCTCTACTAAACCTATTCTTTTATACTCGCCTAATTTATTCCA
CTTCACTAACAATATTTCCAACCAACAATAACTCAAAAATAATAACTCACCAAACAAAA
ACTAAACCCAACCTAATATTTTCCACCCTAGCTATCATAAGCACAATAACCCTACCCCTA
GCCCCCCAACTAATTACCTAAA

>c524_g1

GTCAGGCCTGTAATTAGTTTTGGACTGGTAGTTAGAAGAATAAGTGGAATTATGTGAAG
GGCTATTAGTGTTAGTTCTCGTGTGTGTGAGGGTTGGAGGTTAATTATATGGTTGGTTAG
TTTGCCGCGTTGGGTGGTAATAATTATGTATATTGAGTATATACCTGTAATAATAATGTTA
ATTCCTATAAGAATAATGGTAAAGTTTGATCAAGAAAATAATGATATGGTAATGAATAAT
TCTCCTATTAGATTGATTGAAGGGGGTAGAGCTAGATTAGCTAGACTTGCTATCAGTCAT
CATGTGGCTATAAGTGGGAAGACCATTTGAAGTCCTCGGGCCATGATTATAGTACGGCT
GTGGATCCGTTCGTAGTTGGAGTTTGCTAGGCAGAATAGGAGTGATGATGTGAGGCCAT
GTGCGATTATTAGTATTGTTGCTCCTATGAAGCTTCATGGAGTTTGGATTATGATTGATGC
AATAACAAGTGCTATGTGGCTAACTGAGGAGTAGGCGATTAGTGATTTTAAATCTGTTT

GGCGTAAGCAGATTGAGCTAGTTATAATTATTCCTCATAGGGAGAGAAGGATGAAGGGG
TATGCTATATATTTTGTTAGTGGGTCTAGAATAATGGAGATGCGAATTATTCCGTAACTAC
CTAATTTTAGAAGAATAGCTGCTAGAATTATTGACCCAGCAATTGGAGCTTCAACATGG
GCTTTTGGTAGTCATAGGTGAACTCCATATAATGGTATTTTAATAAGAAATGCTATTATGC
ATGCCAACCATAGTAAGTTGTTAGATCATGAAGCGTCTAAGGTGTGTGTTGTGAATGAT
AAAATTATGAGGTTTAGGGTTCCTACATGGTTTTGGATTAAGATGAGGGCAATTAGCAG
TGGAATAGAACCGATTAGGGTATAAAATAGGAAATAAATCCCTGCGTTTAGGCGTTCAG
TTTGGTTCCCTCATCGGGTAATAATAATAAGTGTTGGGATTAAGGTTGCTTCAAATAAAA
TATAAAATATAATTAGTTCAGTTGCTGAAAAGGTTATGATTAGGAGAATTTGTAAGCTGA
TTAGTATTGAGATGTAGAGTTTTTGTAGTACGTTATTATCTTTTTTTAGGTGGTTTTGGCT
AGCTATTAATATTAGTGGCAGTAATCAGGCTGTTAAAATAATTAATGGTGTGGATAGGGG
GTCTGAGGAGAATATATTTGAAAAGTTTTTATAATTTTCGTCGGTTTGTCATAGAAGTGT
TAGGCTGGTTAAACTAATTAGAAAACTATATGAGGTTACGTTTGTTCAGGTTTTTTTAGG
GCTTGATAGTCAGGTTAGTGGTAGTAGCATTAGTGAGGGAAGAATAATTTTTAGCATTG
TAGTAGGTTGAGATTTTGGACGTAATCTGTTCCGTACGTGTTTGAAACTTTTACTAGTAG
GGCTAGTCCTACAGCTGCTTCGCAGGCTGCGAAAACTAAGATGGTGATGGGGATTGGT
ATGGAGCTTATGGAGTTGGAGTTTAGGGAAGTTACTGAAGTTATAATAAATAAGGATAA
TACTATGCCTTCCAGGCATAGTAATGTGGATATTAGGTGAGAGCGAAATATAAGTGTCCC
TAGAAGTGATAGTGAGAAGGCTATGGTGAGGTTGAAGAAGGTAGATGGCATATTGGTA
ATTATGAACATCA

>c6419_g1

GTCTCAACGTCTCATCGCTCTGCATTCTACGACTCGCCTACGTCGCGAACACAGGTATC
AGATTCGAGCAGATCGCCTATATATTCTCATGATCCTGTTCATGTCCGTTTACCCCATGTC
ATCTCTCCTGCACGGTCCCGGACGCCTTCAGAACAATCTCAGGGATGGCCATCTGCAA
CGTCAGACTCGCAAGGGCACTGGGATCCCATGGATATATTTCCTCATGTCGAGATCCCT
TCGGATGGATTCCCTCAGGCGATGGACGTTCATACATCACCGATGCTCCGCGAGTACCC
TTCGAGACCTCACAACGAAGACTCGATACTCGAGAGTGCCAATACGGGCATACAAGAG
GTCGCGCAACAGCAACCCCCAAGCAAGCTTGGTAGGTTCGGTTTCGGCAAGAAGCCG
ACCAAGTGGGGCCTCGGCATGTTTAGCCATGGTGACAAGGGCTCGCAAGCCCTTCCTC
CTGTGCAGGAGATTCCTGTCAGCCCTGCTGGGTCGACACCCTCTCTCAAACGCACGCA
GTCTTCCAGTACGGACAGTCGGTCGTTGTCAGAGCTTTCCCCTATTCAAGAGGCACCC
CGTGTCATTACAGATGCCAAGCAACGCAAGAAGGAAGCGGCGCGCATGCAGCGCGAC
GCGGAGAAACAGCGAAGATTACTGGCTGAGAAGATGCAGAAAGAGCAGGCCCGTGCT
GTCCTGGAGAAGAGCAAGCGTATCCGTGACCGGTCGTCGGCCGACGACTTCGAATGG
CAGAGTTCTCTCGCAATGATGCAAAACTCCACGAATCACCGTCATGCCAGGCAGCCTA
CCGCCGCCGGACCCATTCGCCAGACGCAGAGTCACGGTGTCTCATCTGTTGACGCCGC
TGGTGGAAGTTTCCTCAGTCCAATCGACGCACATCCACGGAGCGATTGGCGTGTACGC
GATGAACGGCTCCCGAAGGCGCGTAGGCGGGACTTTGATGATGATCATTCCATGTCGTC
GACGGACATGCCAAGCGGCGTTTCTGTGATCTCGTTCGCGACTGTCGACAGCGATCCT
GGTCCGGCGCGATTACGACATAAACCGAGCGCGCTGGGTATCAGTCGGATGACGTCCA
TGTCGTCGCTAGGGACAACACTATCAGAAGAGTTTTCGCCGCGCGTGCGATCGTCTAC
CTCGCTGACGATGGAACAGCAGTTCGCGAACGATTTCCATGTACGTGCGTCTGTCGATT
CGTCGTCCATATCGGACGGCGGTTCGCCCGCTGTGCCATCGATGCACATGCTTTCGCTG
TCGTCGCCGATACCTTGGGCACGGCATGAGAACGGGAGTCAGTCAACTGTAGAGAATG

GGAACTGCAGGTCCCCTTCCGAGGGATACCGCCCCACATTGACCATTCCCCCACAACC
GAAACACCCAACAGGCTTCCAGCCGCAGGGGCCGATAAGTCCCTATGCGTCAGGGAAT
GGAAGTTATCCTTCTAGTCCTGGTCTTGCGCCGAAGTCTGCTATTAATCCTATCTTCAAA
GTGCCGCCATTCACGACGCTGCTTACGCAACCAGGCCCCCTTCCCCCTTTCTCCCAACT
CGAGGCTGTCGCGGAGGGCGAGTATCCCCCATTGTCCCCTATGTCATTCACGTCGCCGG
AGGATATCGCCAACGACTTTAGCTGACACTTATGGTGATCGTCGTCTCCCTGGCTTTTG
CAGTCGCCGTCCCGCACAGCTCGTGGTTCAATTGTTGTACATCTTCCTGGGTCGCCTGT
TGCATATCACGCTCGTTCTTGCTTCCTATTTAAGGCTCGCATCGTGCTCTCATCCTCATCT
CCATCTCTGCACGCGCCTTGCTCCTAGCGCACTTCGCCCCCCTCTGTAATTCACACGGA
CGTCCACATTGCCCCGCGTCGTTCTCTCCATTGCATGCACATTTTCCACCACCACCGCAT
ATCCAGTTTCTCCGAACTTATTCGTTGCATTCATTCGTCATTCATTGGCTCTGGTCGTAC
GTTCACAGTTCACGACGACCTCCCTCAATACCTCTTCGGGACGGCCTATCTGCATCCGC
CGGCGCGCCTTCACATGGAAGACGATGCCACGCCCGATGCCTTGGCGTTCCCCCACAC
TCCCTCACCTTCAATCTTTCATCCTCAGCCATATCCCGCGCTCTATTCGCTTTCATTACTC
GCTACATTACAATTAGTCCACTATCACTATTCTGACGACTCGCGCGTCCGTCCCAGGCA
GCGCGCCGGCGCATGCTGTTGCATCGCATTATCATCACCGTCGTCGCACGGGTTTTTT
TCAATCCACTTACAATACATAGACTACATCCTCATGTGTATTTGTACAAGTACCTCGAGG
TGGTGGTTGACCCAAAAAAGGAAACAAACACAACTCCAAAACGTGTGGGTGTGTGCG
GGGAATCCGGCCAGATGCGCCTAAAGGAAGACGGTGGCGCGAAGAGTCTTAACTAGC
GGGTCGTCCTAGGCTCTGGAGTGTTCGAGGGGCTGGCTCGAGAGCGGACAGAAGCGT
CAACGTATACGGCAAGGGATCACATGCACAAGCAACGTGAACGCTTGCGGCGACATCA
CTATCGCCGTGCAGCCACCGCACGGCGTGATTTCAGAGCGTTGTATGAACGGTTGCGG
GGACAGAGCGAAGACGATTACTCATTACGCGTGCAGGACGTATCATAAAGATCTGCGT
TGGATATACAGAGACTCGAAGTCCCCGATCAATGCCACGTTAATCGGATCTCAAGGTTT
CATGCAATACAGCCTCGGCTTTGCACGGCTTGTCCAACGCCTCGTGTAGGCTAATTCGC
CGGAGAGTCTGTCCTGCTTGCATTGAGGCGGTGAGATATTGACCTCCCGCACACCGTA
AACGTCAGAGCCACAATTTCTCGCTGCGGAAATGACAGGGTTGACGGGTGATTCTATT
GTGTAACGGATATCCGTTATTTACAGCCCCGCGTCCGAACATGTGCAATCTTTCACAG
GGCCTGGCAGTGGGAATCGAGAGAGCGTCGGGAGGCAAGTGCGTGAATAGGTTCTGG
AGAGCGTTCGGGGCGATCGGCGTTCGCACGCACAGGCATCCGGGACGGCAGGAGAAG
ATAGGATGCGGAAGTTAATGCCGCAAGACGTGCGGGAATTGAATGGAATGCGGGAGCA
GGGAGCAATAGCATTGGTCAGCTGCTGTACAGAATTGCGTACAGAAGGGATGCCATCT
AGTGATGAAGTGCCTGCTCGAAACGGAGACCACAATAGCTGCGAGAACGAAGGGGAG
GGGATGAATTGATTCAGCTAGGCGTCGCACAAATTCTATAGCAATCAAGGGGAGACGC
ACCCGCAAGCCCGAGGACCTGGCTTGTCCTATATCAACGCCGTCAGCTGCTGTCTCAC
GAGTACCTTCGTATGAGTAACTGACCAGGTTAATAAAAATCTTCGGGTGACCAAGAGG
ACCTGCCCCGCCATCACAGACTGCTTTCCGGCCATGAACAACACGAATGGGCTCATTG
CTAATCATTTCCATCGCACTGAGAGGGTTAGGTTGGAGCTCCATGATGGTCTGCTCGAA
GCGAGGACCGGAATGGGCCTGTGGACGAGGATGCTGGCTCGTAGACCATGTGTTTGGG
AAGTTGGGGGCTTGCGGGACCGCCTCGGCGGGAGTTGTGGGTTGGTGTTTGGGAACG
GGAGTAGAAGTCGACGCAAGACGGGTGAGAGGACGGGCCACGCCCTGGAGAGCCCG
ACGAGCGAGCATGGTGGTCAAAAGGTTGGACCCAATATGGAAGGAGCGGGCAAGGCG
AGGGCCGGGAAGAACGTCCGGGGAAGTCCGGG

>c2305_g1
CTCTGTTCATTCTAATCCTTTTTGTGTTCATTCATATGCTAGGCCTAGAGATAGAATTGTG
ACTAGAATAAAGGCTATAATTATTATAGTAGAGGTTTTAATTGTTTGAATTGCTCATGGTA
GTGGAAGTAGAAGAGCAATTTCTAGGTCAAATAATAGAAATGTAATTGCTACCAAGAA
AAATTTTATTGAGAATGGTAGACGTGCAGAGCTTGTAGGGTCGAATCCGCATTCATATG
GATTTGCTTTTTCTGAGTACAGATTTATTTGGGGGAGTCAGAATGCAACTAGAATTAGC
GTTAGGGATAATAAAATATTAATGAAGATAACAGTGTACAGGTTAATTACTCTCTTCTGG
GTTTATTCAGAATCTACTAATTGGAAGTCAGTTATATTAATTATACTAAGGG
>c3247_g1
GCCACCCATTCATTTGTATTCCAAAGTTAAGCCCTTATTGTTACATGTTTTACATCCACC
ATGTTTTACATCCACGAGACCCTCCCGGAAGCCAAATCTCACAGTCGCTTCACTGACG
ACAACACAACATTTTACCCCATCACACGCGTCGGATGTCCTTCCAAGAATGCAGCGAT
GTTTTCGACCGTCTGTCCCCAGAAAACCTGGAAGAGAACGAGATCAAATATCAATTCC
ATGATATGGGGGCAGTCGCGGCCACACACCTCGTAGTTGGTGTCGCTAACGTAGCCCG
TGTGCGGGCTGAGCGTCACGTTGTCCAACTTTCTCAGTGGATGATCCAGCGGCAATGG
TTCCTGATCAAAAACGTCCAGCCCGGCACCCGCAATCCGCTTCTCTTTCAACACTCCTA
CCAATGAGGCTTCATCGACCAACGGACCGCGAGACGTATTGATGAAAAAGGCCGTGG
GCTTCAAAAGTGCCAGGTCGGCTGCAGTGATCATACGCGTTGTCTGCTCCGACAGGAC
CATGTGAATCGAGACGATGTCGCTGCGCTTGAACAGGTCCTCCTTCGTTGGTACGAAC
GTCACGCCCGCCTTCTCGGCTCGATCAGGAGTGAGATTCGGCGACCAGCCGATGACTT
CCATTCCAAAGGCCTTCGCTATTTTACCAGTCGCGGCACCCAGATTGCCCACGCCAACT
AAACCCAGCGTTCTTCCTGCAAGACCGAAAGGGATCGTAACTTGCCAAGGGACAGCG
CTAGCCTTGACCCGCGCGTCCTCCACGGCGATGTATCGAACTGTGGCCAGTAGAAGGG
CCCAAATATGCTCTAACGTTGAGTGGCCCTTGCCAGCGGTCCCGGACACAACGATGCC
TTTAGACTTTGCGTGGGCAACGTCAATCCCTCGGTTATAAAGGCCCGTCGTAGCAATGA
GTCGCAAATTTGGAAGCCGGTCCAAAAGAGAGGCGTTGAATTTTGTCCTCTCCCTCAT
CGCGCAAATGATGGGTGCTAGTCTCTCCGCAAGAACCCCTTCATCAGCAGTCGTATCAG
GGAGGGAGTCTACAGCAAGTCGATCTCGTATAGCGGACCAGTCCGCGAACTTGAATGC
GACACTTTGGTAATCATCGAGAATTGCAACGCGAATCGCTTCGGAGGGCATAATCTGGA
GCAATGAGGTGAGAGGGACCACAGGTTGCCTGAGAACAG
>c7606_g1
GGAGTATTCAGGATTGCAGCCGCCAAACTCTGCTATGAGGTCATACTCTCGTCGTCCTT
CCGAGGCTACAAGTTAAAAGTGGGCTTCATCGTCTCCTCGCTCGACCCTCAAGGAGAT
AGCATACATCGAGATGGCGATGCTTCTGCAGGTGCTGCCAGTCCGTTCTACCGCGTTTC
TTCATTAGATGACGAACGTACATACGCACAGATGACCACTTTCCTCACCCAATACCTCC
ATGCCTCCGGACATCAACCACCCAAATCGCAGGATGCCCTCCGCATGGGCATCATTTCG
ACCGCTAACATCAACTCCGCCGCCCTTGTAAATCCCGCAAAGAGCCATGGCGGTGTCA
TCATTTCCGCTGTTGCCTCTCGCGATCTCGAGCAAGCACGCAAGTTTGCCAAGCAGTA
CGACATACCCAAAGCGTATGGATCATACGACGACCTCTTGCGCGAGCCTGGTATCGACG
CTGTATACATTTCTCTGCCTAACGGCATGCATGGCACATGGGCGAAGAGGGCCTTGAAC
GCGGGGAAACATGTTCTCCTAGAAAAGCCTTTCACATCCAACGTCGACGAAGCTCATT
CACTTGTCGAGCTTGCTGTACAGAAGAACCTCATCCTCGTGGAGGCCTTCCATTGGCA
ATTTCATCCGGCTGCACATTCCGTCAAGGCACTTCTCGACAGCGGCAAGTACGGTCCG
ATCGTGCGTACCTACGCGCGCATGACTACCCCGAAAGGCTCCATCCCACGTAGCGACAT

TCGCTGGCAGTTTGACCTCGCAGGTGGTTCTTTGATGGATATGACCTACGTCATCTCCT
CCACCCGTTACTTTCTCGACGCGAGGACTCCTCAGGAAGTCGTGCAAGCAAAGGCCC
GCCTCGCGCCGTTCGATTCGCGTGTGGACGATGCGATGGAAGCCACGATGCGCTTCAA
TGTTGCCGGACGCACGGTACAGAGCGACATATACAGTGACATGTGGCGCGCGAACATA
CTGGGGGTCATCCCGCGAGTATGGGAGGCACCCAGCATTGAGATTGAGACGGAGCATG
CAGTGATTTACTTCTACAACTTCATGATGCCACACCTCTATCACTACATCACTATCTACG
ACAAGCGTACACAGCAGACTACCACCGAGAAACACTATTCTGGTGGACCAAAGTGGA
ACAGCCGAGGTGAGACTTGGTGGAGCACGTATAGGTACCAACTCGAGGCATTCGTAGA
TATGGTTCGTGGGCGCGACCCCCCGCACTGGATCACTCTCGACAGCAGCATATCTCAGA
TGGAAACGATCGACATGATATACGAGAAGGCGGGTCTTGGGAAGCGAATTTCGACGCC
AGAGGGCCTGTACTAAATCCTGTGACAATGTTCTGGACCAGAGTTCGTCAATTGTATTA
TCTCCACCGCACATCACTCGCGCCGGAGGCATGGGCCTGTGGGACACCATGTTATATTG
TGACTTCATT

>c7742_g1

TCCCGAACATGCAGCCAAATTTCTCGCAGACGTTCTGGCAGCATCCTCCCAACGCGCG
CAATGCAGGCCTTCCGCCGGCAGGCTCTCTCACAGCCAACGCCCTTACATCTGCTCTC
GCAAACCCGTATGCTCAGGCACCATACGCTTATCCACAATCCTCTCACTATGCCCAAGC
ATACGCCTCATACTTCAGCCAGCCAGGACCTTCTTCAGTAACAACGGGAGGATATACCC
TTTCATCGACGTACAACCCGAACACACAACTCCAGTTTAACACACCTCAACGTCAACA
TGCATTTCCTAATCCACGCCCGCAAAACTATCCGCGAGGACAGCCTGCCGTGGGGTGG
TATCAGCCTGGTACCTCCCGATGCTTGAAACAGGGATGCGCATTCAACGGGTCGAAGA
AGTCGGTCGATATCCACATGATGGACCGACATCTCATCTACCCGCCCGGTTGGGAGCAC
CGGAAGCTCAACAACGACTGGGATGCTGATCCTAGTCTCAAAGGGAAGCCTATTCCTA
TACAGGGTACGAGCTTCCGACTAGATACACCCGAGGCAATAGAGGCGTGGATGGCGGA
GCGCAAGAAGCGGTTCCCGACCGCCCAGAAGGTCGTGGAAAAGGAGCAGAAGATGC
GCGAGGCTATTGAGCGGGGCCAGCTCCCGTTTGATTATCGGAACAAGCGGAAAAGAAC
AGACGATGGAGGCGCGGAGAGGTCAGGCAACGACCGCGGGTTTGGGAGAGGGTGGC
GGGGAAGAGGCAGAGGAAGGGGTCGGGGCCGTGGTAGTGTTGACTGTGGGTGGAAT
GGGAGAAGGCCCGCCGAACCCGAACAGCAGTCGGTGGCTCCTGCGCAATCTACTCCT
GCTGTGCATCCGCAGGCCCTGGTCGCCGGAAAGGAGAGATCCTCCGAAGAATCTAGCT
CGGACTCTGATGACGCGCCAGAGGCTATATCATCAAAGCCGCCTCCAACAGGCACAAT
CAGCGCTGTGGATGTAAACAACGACGACGACTCTGATAAGGAAGAAATGTCTGCTGTT
CCGCATGTAGCCGGCCACACCATCGCGCGAACGCAACAACGCCCCCGTCCCAAGCAA
CCTCGCAAACCCATACGTAATCCTTTCGCCCAGCATCCATCGTTATTGGGTAACGTATGT
TGTTTGATGATCCCTCAGGCAAATTAGCAATCTCTGACCCTTCACATCGTTCCTCCGCA
GTTGTTACATCCGGAAGTGCGAATGACCGTGTCTAATCTCTCGCAAGCCATTCGATTCC
TTGTCGATAATGACTACCTTGACAACGTCGAGCTCAAGCCTGGTCAGGCGAGCGAGCG
CATGATAGAGGTAATTGGGGACCATAAATAAGACAAACCGGAGGCGGACGTTGATACC
ACAACTCCCGCCGGCCCGGCAAGAGAGTTACGTGAACACCGCTTCTTCTCTTCCAGTG
TGTCTCTGTCGTCCGACATTCAGTTGTCAGATCCCATCTTTTTCAGCTGTCGACGCGTCT
CTGATCACATGAGGGCGTCAAGGCGTCTTGTTGGCACTTATCATAAATGCTCCCCGTTA
TCATACACGTGTGGATATAGATATACATCACAATTACTACGTACAAGATATCCTACACCC
ATGAAAAGCTTCCTGTCAAATTATCTCTCCCGTGTAAGCACAACTTCATGCTGTCCACT
CATCGACCCTGCTGAGTGACGGGCATCCAGGTGCTGGTGGGTTTGAAAACCTCGTCTG

AACAAAGACTTTCTCGACTCCAGCGACGCGCTTGTCAAACGCCGCATACAGCTTCGGA
AAGTCCTCAATGTCGACACGGTGAGAGATCATAAAGGTGGGGTCAAGCTTTCCTGTGA
CGATCAAGTCGAGGATCTCCTTCCACCACTTGTGCACGGGAGCCTGACCATTGCCGAT
GAGGCGGATGCCCTTCTCCATGAGCGCGCCAATGTTGAAGTGGTTCGTGTACCCCGCG
TATGCGGCAATGATCCCACATCGGCCGCCCTTCTTGACAGACATGAGCATCTCGTTGAC
AATTTCCGAAACATCCGTCTCCAGCATGAGCGTCTTCTCAATCTTATGCAAGAGTGATT
TGGGTTGATGAAATGTTCCGCAATCCATGGCCACATCGAGGCCTCCCGGGACGATCTCC
TGAAGACGTTTCACAACGTCAGAGTGTTGTGAAAAGTCGACAGTCTCGACACCAATTT
CCTGCGCCTTCGTAAGCCGCTCGGGAACGCGGTCGATACCGATAACGCGTGCAGCGCC
TTTCATTTTAGCAAACTTGGCAACACAGAGGCCAATTGGCCCAAGACCCCAGACGCCT
ACAACGTCCCCGGCGTTAACACCAGTATCCATGACGTTATGATATGCGGTACAGAGGAC
GTCGGAGAGGTAGAGTGCTTTTTCGTCCGGAATGTCGTCCGGAATGGGGAGCAGGTTG
ACATTGCCTTTGGGGACGCGGACGTATTCTGCCTGGCCGCCAGCAAAGCCTCCGGTAA
AATGAGAGTAGCCAAAAAAGCCCGCGTCACGGTGACCATACATAGCATTCTGCAACG
TCGAGGGGTTGGTTCTATCGCAGAAGGAGGACAGCTTCTGTTTGCAGTATTCACATTCC
CCGCACGCAATCTGAAACGAGGCAACGACGCGCTGGCCGATCGAGAGGTTGTTGACG
TTAGGGCCGAGTTTGTCAACTACGCCCATGAACTCATGACCAAGGATGTCCCCCTTCTG
AAGGGTCAAAATCTCTCCATGATAGAGGTGGAGGTCGGAACCACAGATGGTCGTTCCA
GTAACTTTGAGTATGACGTCGTCCGGTTCGGTGATATCGGGAATGGGGGTATCAATGAG
ACGGACATCCTTCGTGCCAAACCAGGCGAGAGCTTTCATGGTCTTTCCGTCCGTGCGT
GTGTTGTATTCTGGATGAACCTCGGTGGGTATTACACCCATTGCATTCTGTGCTGCGTTT
GCGGCTGCCTGCATGTTGAGAAGCGATGTTATGATGTTACAAGGGCTTTGGTTTGGTGA
TGCGTAAGATGTGAGGAGAAGAGCGTCGGGGATGAGTTATATACTTCTCGTTCTGGAA
GAGACGATGAGCGACGTATGACGGCACCGACACCCACCTTCCACCACAATGACATCAC
GGTGCCATTCTCCACGGTTGTTATGTTACAAGTCTTTGGAGTTATGGTGATTGCAAAATA
ATAATGCTCAGCTGTGCCATGAGGAGACGTAGTAAGTAACCGCTTGCGAGCTGTTAGC
CTAACAGATGAAGGTACGTTCAAGAAAGGCATCCTCCCTTCAAGCTCTGCATATGACTG
CACGAACAGCGCGCAATCACCCGCATTCTCATGCCCTGCGTGCATAGCCTGCGCGTCG
GATATAGACACGCAGCCCGGAAGCGAGCGCAAGATGAAAGGCTTCCCGTTCCATACGT
CGGGCCAGCTGGCGCGATCGCCCAAAGCCCACGACCAGTCTGACTGCTCTCTTTTCGA
ATACGAATAGGGCGATTGCC

>c14038_g1

GGTCAGTCTCTTAAATCATGAAGATAAAGATAACTGCTAGTGCAGTGATGAGGCCTGCA
AATGCATAGCATGCAATTTTACCAATCACACCAGTGTGGAATTTAAGGTCGTGCATGCC
GTGGTGTACACGGTGCATTGCGTGCCACATTGGCAATGCTAGCGTGCCAATGATGAATA
GCGCACCGATGATGCTGGTTGCGAATGCAGAGACACGCTCGTAGCTCATCGCTTCTGC
ATCAATCACGCCTAGTGGGACCAAGATACCTAGCACAAGAACAGTGATTGGCGTGATC
ATCGCGAACCAAGTACCACCTGCGCCAAACAGACCCCACCAGATTGGCTCATCAGAAC
GTTTTGGTGATGTGTTTACACTATAATTCGGTTTCATTGCTCAGCTCCTTACACCACGAT
GAGAACGATCAGTGAGATGAACGCAACCGCGGCCCACTGAGTCAATACGATGATCTTC
TTGTCCACGGGTTTGCCTTTCAAACGAATTGGCATTACTTGTGGCATCATGCTGAAGAA
AGTTTGAGCGTGGAATAGGCTACCGAGTAGTGCAACGATGTTGATTGCGACTACGATTG
GGTTCGCCATGAACGCTAACCAACCTTGCCATGCTTCAGGACCTTTCACCAAGCAACC
TAAACCGAAAGTTAGGAACAGAGTGAATAGGATTAACGGAAGCACCGTCGCTTCACGT

AGCATGTAGAAGCGGTAGAAAGGATGGTTCTTCCACCAAGTGCGTTTTACTTCACGAA
CGTAAGGTTTACGATTGCTCATCCTTATGCCTCCACTTTCTTTGGTGAACCATCAGGTTT
CAACATAGCAATCACGAAGTCCATAGAAGACTCCACTTTGCCTTGGTTTACTGCCGCTG
CTGGGTCAACGTTCTTCGGACAAACTTCAGAACAGTAACCAACAAACGTACAACCCC
AAGCACCGTTTTCACCGTTGATAAGCTTCATGCGCTCATCTTTACCGTTGTCACGGCTG
TCTAGGTTGTAGCGGTGGGCTAGCGTTAGTGCAGCAGGACCGATGAACTCTGGATTAA
GACCAAACTGAGGACATGCTGCATAGCACAGACCACAGTTGATACAGCCAGCGAACT
GCTTGTATTTCGCCATCTGTTCTGGCGTTTGCAAGTTCGTACCGTCTTCTGGTTTACGAT
CGTTACCAATGATGTATGGTTTGATTGCTTCTAGGCGCTCGATGAATGGCGTCATATCAA
CAATCAAGTCTTTCTCGATTGGGAAGTTCGCTAGAGGCTCGATAGTCACACCGTCTGGG
TAGTCACGTAGGAAGCTCTTACAAGCAAGTTTCGGTACGTTGTTCACCATGATGCCGCA
AGAACCACAGATCGCCATACGGCAAGACCAACGGTAAGACAGATCTTTGTCTAGGTGG
TCTTTGATGTAACCAAGTGCGTCAAGTACAGACATGGTTTCATCGAATGGGACTTCGAA
TGTTTGCTTGTATGGTTCTGCATCTTTTTCTGGGTCGTAACGCAGGATGTCTACTTTTTG
AATGCGATTTGCTGACATTATGCTTGCTCCTCTGCGTTCTTCTCTTCAGCCGCGGCTTTT
TCTGCTGCTTCACCGTATAGACGAGCTTTTGGTTGTGACTTAGTGATGGTCACATTGCTT
AATCGATACTTGGTGCTGACTCTGGCTTGTAGAAAGCAAGTGAGTGTTTTAGGAAGTTA
CGTCATCACGTTCTGTACAACCGTCGTCTAGACGTTGGTGCGCACCGCGAGATTCTTTA
CGTAGAATCGCAGAGTGAACCATCGCTTCTGCGACTTCTAGGCCGTAACCCACTTCGAT
TGCGTATAGAAGGTCTGTGTTGAACACTTTACCTTTGTCTTTGATGCTGATGCGTTTGTA
GCGTTCTTTCAACTCAGTGATTTTGTCGATAGTCGCTTGCATCAAATCTTCTTGGCGGTA
GATACCACAGCCCGCTTCCATGGTGTGGCCCATTTCTGTGCGTATTTCTGCCCAGTTTTC
GTCGCCTTCTTGGTTCATCAGTTTTGCGATGCGTTCTTCAACGGCTTTGACTTGCGCGT
CAATTGCCGCGTCGTTCCAGCCTTTGAATTCTGCCGCACGTTTCACTGCGTTTTCACCA
GCTACGCGGCCAAATACTACGAATTCAGCCAGTGAGTTAGAACCTAGACGGTTTGCAC
CGTGAAGACCAACAGACGCACATTCACCCACCGCGAATAGGCCTTTAATGCGAGTTTC
ACAACCGCCGTCCGTTTCAATACCACCCATGGTGTAGTGAACGGTTGGACGAATTGGA
ATTGGCTCTTTTGCAGGGTCCACGTTTACATAAGCTTTTGCTAGCTCACAGATGAACGG
TAGACGTTCTTGTAGGTACTCTTCACCAAGGTGGCGAAGGTCTAGGTGCACCACGTCA
CCCAGTGGGTGTTTGATGGTGTTGCCTTTCTGTTGTTCGTGCCAGAAAGCTTGAGAAA
CTTTGTCACGAGGACCCAGTTCCATGTATTTGTTCTTCGGTTGGCCAACTGGAGTTTCA
GGGCCCATGCCGTAATCTTGTAGGTAACGGTAGCCATTCTTGTTAACGATGATACCGCCT
TCACCACGACAACCTTCCGTCATCAGGATACCTGTGCCTGGAAGACCGGTTGGGTGGT
ATTGAACGAATTCCATATCACGTAGAGGAACGCCGTGACGGTAAGCCATTGCCATACCA
TCGCCCGTTACGATACCGCCGTTGGTGTTACAGTGGTAAACACGGCCCGCGCCACCTG
TTGCTAGAACAACAGATTTTGCTTTGATAGTAACAAGCTCGCCTTCAGACATGTGAATA
GCAATCAGACCTTGAACTTCGCCTTCATCAACAAGCAGATCCACCACAAAGTACTCAT
CAAAACGTTTGATGTTGCTGTACTTCATTGATGTCTGGAATAGCGTATGAAGCATGTGG
AAGCCAGTTTTGTCTGCAGCGAACCAAGTACGTTCAACCTTCATACCACCAAAGCGGC
GTACGTTGACTTCACCGTTCTCTTTACGACTCCATGGGCAACCCCATTGCTCCATTTGG
ATCATTTCGCGAGTCGCGTTCTCTACAAAGTATTCAACAACATCCTGTTCACATAGCCA
GTCACCACCGCCAACAGTGTCGTTGAAGTGGTTGTCTAAGCTATCTTCATCCTTGATAA
CTGCTGCTGAGCCACCCTCTGCAGCGACCGTGTGGGAGCGCATTGGATAAACTTTAGA
AATCAGAGCGACTTCTAAGTCAGGGTTTGCTTCAGCCGCTGCAATAGCAGTACGAAGA

CCAGCGCCGCCTGCGCCGATGACTGCGATATCTGTGGTGATAATTTGCACAGTTATCCT
CCAGTGTGTGATTGCGGAAATCCGCTTGTTATTGTGATTGCGAGGCGCTCTTTTAAAAG
AAC

>c7145_g1

GATTCTTTGGGGGGTTAGAATGACGCAAACGCGTCGCGCGCCACATGCAAAAAGTCTC
CATTTTGGGAAGCAACGCGGTTCGTCACAGATCACGAGGGCGTCGCGCTCACGCCTTT
CTGGGGTACAACTTGCTTGCGCGCGGCCACACGATTGCCGAGGTGCCGAAACGGAAG
GTTCAAGTTCAATGATGTCACGGGCACGGGCCGGGAAATTGTGCTTCAGCAATATGATT
GAAGGTCAAGCTCTTCACAGCGACAGATAAAGATCAGCTGCATAAATTTCGTCGTCTTT
GTCTGTGTTGGCGCATCCTAGCTTTCTACCCTCACTCGTACAACTCCGTCTCTGTCCAC
CTCGTTGCCCACGATGCCTGAAGGAAACATGAAAGCTTTATACTACAGCGCGGCAAGT
GCACCGTGCGATGATGTCTATCGGAGTTGCCATCTAATCGCCGGTGTCTAGCCTCACAA
ATTCGAGATCAAGGAGGTCCCCATTCCGAAGTGTGGTGATGACGATCTCCTGCTCAAG
GGTACTACACCAGTCGTTCTAGATCTGCCTAACCCACGCTCACTCGCACACGCATATAG
TCACTCTTTGCGGTGTCTGTGGAACCGATGCACACATCCACGAGGGTGAGTTTATCGC
GAAATTCCCGGTACGTTTTCATCAGTATGTGACTATATGCGTCATCTGCCGCTCAACCTA
CGCACCCTCTCCTCACAGCTCATTCCAGGCCACGAAGCGATCGGCCGCGTGGTCGAGA
AGGGTAAGAATGTGAAGGACTTCGAGATCGACGACCGCGTCGTTGCAGACGTCGGTAT
CTCATGTGACGACTGTTTCTACTGCAGACGCGGTAACCCCGTTCTTTGCGAGAACTTCA
ACGCGCGCGGTGTCACCTTGGATGGAGGCTTCGCCGAGTACATCGTATACCACCAGCG
CAAATGCTACAAGATTCACAACCTCACTGATGAGGAGTCAACACTTCTTGAGCCCGCC
GCATGCGCAATCCATGGTCTCGACAAGCTCAACCCACCCGTCGGCATCGACGTCCTGC
TGCTCGGTGCAGGCCCAACAGGTCTCATCCTGGCCCAACTACTGAAGCTAAATGGCGC
CCATCGCGTAGTGCTCGCAGCAAACAAAGGCATCAAGATGGATATTGCGAAGCAACTC
GGCGCTGCCGACGAATACATCGAGCTTGATAGGCAGAATCCAGGTCAGCAGTGGGAAC
AACTCAAAAAGGAGAACCCGTACGGCTTCGATGTCGTCGTCGAGGCTACGGGTGTCG
AGAAGGTGGCGCAAGACAGTATCAACTACGTTCGCCGAGGGGGTACAATCATGATCTA
TGGCGTGTACGAGAACAAGGCATTGCTCCACTGGCCACCATCAAAGATCTTTGGAGAT
GAGATTAAAATCATTGGTTCATTCTCGCAAGCGTACTGCTTCCCTCGCGCAGTCGCATAT
TTGGATGGTGGCAAGGTTAACGTGAAGGGCATGGTTACGGATGTGTTCAAGCTTGAAG
ATTTCCAGGGCGCACTCGACAAAATGAACAGTCGAGGTGCACTCAAGATTGCCATCAA
ACCTTGAAAGGTAGCTTCGAAGTGACGATCGCAGGTGGCCTTCTGAGGCGCCAGGCTA
CTGTATAAGTAGTATTATACGAAAGATGATACATATACATATACCCTTTTGACATA

>c10255_g1

TTTTAGGAGATTCCCTATGCCTGTAACTAATATGGCTGAACTAGATGCAATGGTTGCTCG
CGTTAAGAAAGCGCAAGAAGAGTTCGCTACTTACTCTCAAGAGCAAGTAGATAAAATC
TTTCGTGCAGCATCGCTTGCAGCTAACCAGGCCCGTATCCCGCTAGCTCAACAAGCGG
TAGAAGAATCTGGTATGGGTATTGTTGAAGATAAAGTAATCAAGAACCACTTCGCTTCT
GAGTTTATCTACAACAAATACAAAGACGAACAAACGTGTGGCATCTTAGAAGAGGATG
ACAACCTAGGTACTATGACTATCGCTGAGCCTGTAGGCATCATCTGCGGTATCGTACCA
ACGACTAACCCAACTTCTACAGCGATCTTCAAATCTCTAATCTCTTTGAAGACTCGTAA
CGGTATCATCTTCTCACCACACCCACGTGCGAAAAACTCAACTAACGATGCAGCTAAA
CTCGTTCTAGATGCAGCAGTAGCAGCAGGCGCACCAAAAGACATCATCGGTTGGATCG
ACCAGCCTTCTGTTGAGCTTTCAAACGCTCTAATGAAACACGATGACATCGCGCTTATC

CTTGCAACTGGTGGTCCAGGCATGGTTAAAGCCGCTTACTCTTCTGGTAAGCCAGCAAT
CGGTGTAGGTGCAGGTAACGTTCCTGTTGTTATCGATGAAACAGCAGACATCAAACGT
GCTGTTGCTTCTATCCTTATGTCCAAGACTTTCGACAACGGTGTAGTATGTGCTTCTGAG
CAGGCGGCAATCGTAGTTGATGAAGTATACGACGAAGTGAAAGAGCGTTTTGCTTCTC
ACAAAGCTTATGTCCTAAGCAAAGCAGAAGCTGATAAAGTTCGTAAAGTGCTTCTTATC
GATGGCGCGCTAAACGCGAAAATCGTAGGTCAGCCAGCAGCAGCAATCGCTGAAATG
GCAGGCGTTAAAGTTCCAGCAGACACTAAAGTTCTTGTTGGTGAAGGTCTGGGCAAA
GTATCTCACGATGACGCATTCGCTCACGAGAAACTATCTCCAACTCTAGGTCTATTCCGT
GCTGACAACTTCGAAGACGCGGTTGCTCAAGCGGTAACTATGGTTGAGATTGGTGGTA
TCGGCCACACATCTGGTCTTTACACTAACCAAGATACTAACGCTGACCGTATCCGTTAC
TTCGGTGACAAGATGAAGACAGCTCGTATCCTAATCAACATCCCGACTACTCACGGCG
GTATCGGTGACTTGTACAACTTCAACGTTGCGCCATCTCTAACGCTAGGTTGTGGTTCT
TGGGGTGGTAACTCTATCTCTGAGAACGTAGGTCCTAAACACCTAATCAACAAGAAAA
CTGTAGCGAAGCGAGCTGAAAACATGTTGTGGCATAAACTACCTAAGTCAATCTACTTC
CGTCGTGGTAGCCTTCCAATCGCACTGGGCGACCTAGAAGGTAAGAAACGTGCATTCC
TAGTAACTGACCGTTTCCTATTCAACAACGGTTACGCAGATGACGTAGTAAGCCTACTA
AAAGCTCAAGGCATGGAAGTTCAAACATTCTTCGATGTTGAAGCTGACCCAACACTGT
CTGTAGTAGAAAAAGGTGCGGCTCAAATGGCTAGCTACCAACCTGACGTTATCCTAGC
GCTAGGCGGTGGTTCACCAATGGATGCAGCGAAAATCATGTGGGTAATGTACGAACAC
CCAGAAACTCACTTTGAAGAACTAGCAATGCGCTTTATGGACATCCGTAAACGTATCTA
CAAGTTCCCTAAAATGGGTAAAAAAGCTGAGCTTGTATGTATCACTACAACTTCAGGTA
CTGGCTCAGAAGTTACTCCATTCGCGGTTGTTACTGACGACAAGACTGGTGCGAAATA
CCCACTAGCTGACTACGAAATCACGCCAAACATGGCAATCGTTGATGCGAACCTAGTA
ATGAACATGCCTAAGTCTCTAACAGCATTTGGTGGTTACGATGCAGTAACTCACGCTCT
AGAAGCTTACGTATCAGTTCTTGCTAACGAATACTCAGATGGCCAAGCTCTTCAAGCAC
TTAAGATGCTAAAAGAGTACCTACCATCAAGCTATGCGAACGGTGCAAATGACCCAATC
GCTCGTGAGAAAGTACACAACGCAGCTACTATCGCTGGTGTTGCATTTGCGAACGCAT
TCCTAGGTGTTTGTCACTCAATGGCTCACAAACTGGGTGCTGAGTTCCACTTGCCACA
CGGCCTAGCTAACGCACTACTTATCTCTAACGTGGTACGTTACAACGCGAACGACAATC
CAACTAAACAAACTGCGTTCTCTCAATACGACCGTCCACAAGCACGTCGTCGTTACGC
TGAAGTTGCTGACCACCTAGGCCTAAGCCAAGAAGGTGACCGTACTGCTCAGAAGATT
GAACGTCTACTAGCATGGTTGGATGAACTAAAAGTGAACCTAGACATCCCAATGTCTAT
CCAAGCGGCAGGTGTTGCTGAATCAGATTTCGTAGCGAAACTGGATGAGCTAGCGGTA
GAAGCGTTCGATGACCAATGTACTGGTGCGAACCCACGTTACCCTCTAATCAGCGAAC
TTAAAGATGTGTTGATGGCTTCTTACTACGGTAAAGCATTTGTTGAAGGCGAAACTTTC
GAAGGTACAACTGTTATCAAGAAGAAAGCAGACCAAGAAGCAGCAAAAGCAGCACCT
AAAGCAAAGAAAGAAAAAGCTGACGCGTAATTAAAAAATCGAGTGAGCAAGGTTTTC
GCTAGCACGGAACCTTAATCGGGAAAAG

>c12085_g1

AGGCCGCCGCCGGCGACCTCTACGGCATCCACCGCCTGGTGCTGGACGTGGCCCGGC
GCCGCCATCTGCCCCGCGAGCAGGCCGCCGCACTGCTCAACCTCGCCGATCTGGACGC
CCGCACCGGCCGGACCGCCGACGCCCTGGCCCGCTACCGGGCCGCGCTGGACGCCGG
ACGCGCCGCGAACGACCCGTACGCGACCGGCAGGGCGATGGAATCCGTAGGCGGCGC
CCACCAGGAGCTGGGCGACTTCGACCGGGCGGCCGACTGGTTCGGCCGGGCGCTCGC

CCAGCGCCTGGCTCGCGACGAACGCGAGGACGCCGCCCGGCTCTACGGCCGGATCGC
GACCGCCCACACCTACGCGGGCCGCTACGGCGAGGCGCTGCGCAACTGGCGGGCCGC
CGTCGCCGCGCACCGCAAGGCTGGCGATGTCGCCGCGCACGCCCGGGCGTTGAGCGA
ACTCGCGCGCGTCCAGGAGTACGCCGGGCGGCCCGCCGACTGCCTGCGCACCTGCCA
GGAGGCCGTGGAGCTGGCCCGCCGCGCCGAGGACGTACGGCTCCAGGCGGCGCTCCA
ACTGCGGCTCGCCGACACCCTGGAGCGACTCGGCGATCCGGCCGCCGCCGCGCTGCA
CCGGGGCGCCGCCGAACGCCTGCTCGCCGGCGAAATCCGGCCGCCCGGCCCCGCCGA
CGCGTCCACGGCCCAGGACGGTCAGGACGGCACATCACGGGAACAGGCCGCTGACGC
CTGCGAAATCCGTAGCACGCCTGGTGAAGATTGATGCAATGAAAGGCTAGACAGGGAG
AACGCCTTCATTAGACTGGCTCTGCCGCTCGTTCTCCTGCGGTGTCTCCTGGTATGCCC
GTGCGTGCCGGGGTACGTCTTGTATTGCCCCACATTCCCTGAGCCAAGGACCGTGATCG
ACGTGAAGGTCGGCATCCCCCGCGAGGTCAAGAACAACGAGTTCCGGGTGGCCATCA
CCCCCGCCGGCGTGCACGAGCTGGTGCGCAACGGCCACCAGGTCGTCGTCGAGCGGA
ACGCCGGCGTCGGCTCCTCGATCCCGGACGCGGAGTACGTGGCCGCCGGCGCCGAGA
TCCTGGAGACGGCCGACGAGGTGTGGGCCGCCGCCGACCTGCTGCTGAAGGTCAAGG
AGCCGGTCGCCGAGGAGTACCACCGCCTCCGCAAGGACCAGATCCTCTTCACCTACCT
GCACCTGGCCGCCTCCAAGGAGTGCACGGACGCGCTGCTGGAGTCCGGCACCACGGC
GATCGCCTACGAGACGGTCGAGCTGCCGGGCCGCGCGCTGCCGCTGCTCGCCCCGATG
TCCGAGGTCGCGGGCCGCCTCGCCCCGCAGGTCGGCGCCTACCACCTGATGGCCGCCG
CGGGCGGTCGCGGTGTGCTGCCGGGCGGCGTCCCGGGCGTGCCGGCCGGCAAGGCCG
TCATCATCGGCGGCGGCGTCTCCGGCTGGAACGCCGCGCAGATCGCCATCGGCCTGGG
CTTCCACGTGACCCTGCTCGACAAGGACATCAACAAGCTCAAGGAGGCCGACAAGAT
CTTCGGGACGAAGATCCAGACCGTCGTCTCCAACACCTTCGAGCTGGAGAAGGCCTG
CCTGGACGCCGACCTCGTCGTCGGCGCGGTGCTCATCCCGGGCGCCAAGGCCCCCAA
GCTGGTCACCAACGAGCTGGTCTCCCGGATGAAGTCCGGAAGTGTCCTTGTCGACATC
GCGATCGACCAGGGCGGCTGCTTCGAGGACTCGCACGCCACCACGCACGCCGAGCCG
ACCTTCCGGGTCCACGAGTCGGTGTTCTACTGCGTGGCCAACATGCCCGGCGCGGTGC
CCAACACCTCCACCTACGCGCTGACCAACGCCACGCTGCCCTACATCGTCTCGCTCGC
CAACAACGGCTGGGTCGAGGCGCTGCGCCGCGACGCCGCGCTCGCCAAGGGTCTCAA
CACCCATGACGGCAAGGTCGTTTACAAGGAGGTCGCCGAGGCGCACCACCTGGAGCA
CGTCGCGCTGGAGACCCTGCTCGGCTGACCCGGACGGCCGCGCCGCCGGCCAAGTGC
CCAGGGGTCACAAGGCGATACGTCAACATGTGTCGTCAACCCCGCCCACCCGGCCGG
ACCTTGCCCGACAAGGTCCGGCCGGGTGTGTGTATGGTCACTTCGCCGCACTCGGCCA
ACTCGCCTCGA
>c19597_g1
ATTGAACATAGCAATAGCTTGAGTTGGTTCAACGTATTCCAAGTCAAAGCTTTCTGCTA
CTTCTTTACACGTTACTTTTCCGTGAATAACGTTTAGACCTTCAAGGAAGCCTTTATCTT
CTAGAAGTGCTTCGCGGTAGCCTTTATTTGCTAACTTCACGATGTATGGAAGTGTAGCG
TTGTTGAGTGCAAATGTTGAAGTGCGAGCAACTGCGCCAGGCATATTTGCTACACAGT
AGTGAACCACTTCATCAACGATGTAAGTTGGATCTGCGTGAGTTGTCGCGTGTGAAGT
TTCGAAACAACCGCCTTGGTCAATTGCCACGTCAACCACTGCCGCACCAGGCTTCATC
TTAGCAATATGCTCTTTAGTGACCAGTTTAGGTGCTGCTGCGCCAGGAATTAGAACGGC
ACCGATAACAAGGTCCGCTTCTAGAACATGCTTCTCGATAGCGTCTTCAGTAGAATAAA
CCACTTTTGCGCGACCTTGGAATTCTTCGTCAAGCTTACGAAGTGTGTCTACGTTACGG

TCTAGAATAGTGACGTCAGCGCGAAGGCCAACCGCCATACGAGCGGCGTTTGCACCAA
CAACGCCACCACCAACGATAACGACTTTTGCAGGTTCAACACCAGGTACGCCGCCTAG
GAGTAGACCACGACCGCCATGCGATTTTTCCAACGTTTGCGCACCAGCTTGGATAGAC
ATCCGACCTGCCACTTCTGACATTGGTGCTAACAGTGGCAAACGACCCATATTATCTGT
TACAGTCTCATAGGCTATACAGACAGCTTTGCTCTTGATTAGCTCATCAGTTTGTGGAA
AATCTGGTGCTAGGTGTAAATAAGTAAATAAAATTTGCCCTTCGCGGAGCATTGCTCTC
TCTACTGCTTGGGGTTCTTTTACTTTTACAATCATGTCTGCTTGCGCAAATACGTCAGCA
GCATGAGGGAGAATGGATGCGCCGACAGCGATGTAATCATCGTCTGAAAAACCAATGC
CGGCACCTGCATTGGTCTCAACTAAAACTTGGTGACCATGTGAGATGAGTTCTCTCAC
GCTAGCTGGGATCATACCAACACGATATTCGTGATTCTTGATTTCCTTAGGTACGCCAAT
GATCATCCTGTATCCTCTTTCTATAATGGTTGTATTAACTGTTAGTAGG

>c17591_g1

AAATTAAGTTGTGGAGCAACTATAATGACTATCAAAGTAGGTATTAACGGTTTTGGCCG
TATCGGTCGTTTCGTATTCCGTGCAGCGCAAGAGCGCAACGACATCGAAGTAGTAGGT
ATTAACGACCTTATCGATGTAGATTACATGGCATACATGCTTAAGTATGACTCAACTCAC
GGCCGTTTCAACGGTACTGTTGAAGTTGAAGGCGGTAACCTAATCGTTAACGGTAAAA
CTGTACGTGTTACTGCAGAGCGCAACCCTGAAGACCTAAAATGGGACGAAATCGGTGT
TGACGTTGTAGCTGAAGCAACTGGTCTTTTCCTAACTGACGAGACTGCACGTAAGCAC
ATCACTGCTGGCGCGAAAAAAGTTGTACTAACTGGTCCTTCTAAAGACGCTACTCCAAT
GTTCGTTAACGGCGTAAACTTCGACACTTACGCTGGTCAAGACATCGTTTCTAACGCTT
CTTGTACTACTAACTGTCTAGCGCCTATCGCTAAAGTTCTTAACGACAAGTTCGGTATCG
AATCTGGTCTTATGACTACAGTTCACGCAACTACAGCAACTCAAAAAACTGTAGATGGT
CCTTCTGCTAAAGACTGGCGCGGTGGTCGTGGTGCTTCTCAAAACATCATCCCATCTTC
AACTGGTGCTGCTAAAGCTGTAGGCGTTGTACTTCCAGAAGTAAACGGCAAACTAACT
GGTATGGCTTTCCGCGTACCAACTGCTAACGTTTCTGTAGTTGACCTAACTGTTAACCT
AGTTAACGGTGCTTCTTACGAAGACATCTGTAAAGCGATGAAAGAAGCTTCTGAAGGC
GAACTAAAAGGCGTTCTAGGCTACACTGAAGATGCAGTTGTATCTCAAGACTTCATCG
GCGAAGTTTGCACTTCAGTATTCGATGCTAAAGCTGGTATCGCACTAACTGACAAATTC
GTTAAAGTTGTATCTTGGTACGACAACGAAATCGGTTACTCAAACAAAGTTCTAGACCT
AATCGCTCACATCTCTAAGTAATTAGAAAGTTAGCAATTAGCGAATAACTTCGTTAAAG
AAAAG

>c8341_g1

GTCTGTTACAAAAGTGTTGCGCGAAGATCCAACCACAGTATGCGTATGTCGCTTCTTGT
ACGGCATGGTCCCCACTGGGGCCCTTTCCGGCATAAAAGAAAGGGTTATCGGGCAAAC
AATAGAGCAGCGAAGCCTCCTTCCGGACTTGGAAGATGGAATGCTCGATTGGAGGCGC
ATTGGCACCAGGAAGTGGGTTGAGATACAGCGAGATGATGGTGATACCATTAGAGAAT
TGTTCAACATATTTCCTGGCCGAATACAGCGAGTAAAAGTGGTACAGATTGGAGAGCG
CGCTGAGCAGCTTCTTCGTGCCGCCCATCTTGTACCCAATCACGAGGCGGCGTTCTCG
GGAGCCCTCCACTTCGAATACTTCAATCACAGGGCCAAATCGGTCCTCCACGTTCCAC
ATCACACGCTGGTAGATCTCGAGTGTGTTTTCGCTTGCTTTCTCGAGGAAAATAGGATC
GGAAACCGTGCGGATATCGGTTTTGCCATCAGTGCGCGTGGATGCAGGTGGGTTCGCA
GGGAAGCTGCACTTTGTGACAAAGTAGCACCTCAGCTGCTGCGACGCGGTCACGGAG
ATGCTTCCCTGAGAGCGGTACGTCTCGAGGCGGTATGCGTTCTCGGGGGTGGACTTGT
CGAGGAAGAGCTCATCGATGCGTGTCTCGCAGGTCGCGCCAGGCCCTTCGGTGACCGT

TTTGCCCGGAGAACTTGTGTGGATGAACGTGGCTCCATTACCGTTCTCATCGATCTTCT
CGAGGTCGATCACGAGCTTCGAAGGGTCGTGCTTGGTGTACGCGAGGACCTTGGCACC
AAAGAGGGCAATGACATGATCTGATATTACCTCGACGGACTCGCCCTGGAAGTACGTG
TCGTCGATGCCGAGGTGGGTGTAGAACCAGTTGACCTCATTGGCGACGAGTTCGCGAG
GAACAAAGCCCTTGGCGGCAACAGTGGCTTGGACCTTCGCGCGTTGCTCCTCCTTGCC
CTTGAAGACGGGTGTTGTGTACCCGGGGACGTTTTTGAGGCGTTGGATAACCTCTGCG
TGTTGCCCGGGAACCTGGAGGTGCCCGATCGAGTCAGTATCGGAGTGGGCAGGCGTCC
TGCTAGATGGGGTCGACATTATCGCGTCGAAAGCTAGTGTGCAAGAGTGGGGGATGAG
GAGAAGGAGACGGGGAGACCGATGGCATCTGGCCGGTAAATCTGAGGATTTTATACGT
CGACACAAGCTCACGAGCGGTGCGTTTGGGCGATCTGGTCGATGGATTCCAGATGTGG
ACACTAGCGCCAGGCCCCCCCTTACCGACAATCCCGCCTCCCGTGCTCGTCACCCCGA
CGCCGTACCAGCCTTCCTCGCCACCCCGCACCTCGCCATCTCCCCGCACAGTCCACAAT
TCACGACGGACAATCCGCAATATTGCAGGTCAGAAGATCCCTGCTCATCCCCGCGTCA
GCAGTCTCGGCCGGTGCATATGTACATAAGTATGTCCAAGCGCAACACGCATCACGCGC
CGCACTGCTGACCGCCAGTCAGTCGCCCCGCGCGCCTCCGTGTCTTGTGCCCGATGTC
CGGAAATCCGTCGCCCCTACCCCATTGACGGCTACGCAACCCGGACGCGGTCCACCTC
CGTTAAAACTAAATTGATCGGTCCCGGTCAGCCCTCAAAGCCCAATTGTCAGACCCGG
ACCTTGCAATGCATCATGCAGTGTCCCGGATCCTTACGCGTACTACGCGTGGTGGCCAG
CTGCACAATACCATTCAACTACTAGGACTAGGTGCCCTGACCAGGCCTGACCTTGCGTG
TCCATGTAGATCTACGCTCCTTTGGCACTACCATTGGTGCAGTCACCCGAAAGTGCACC
GCATCGGTGTCGGTGGTCGCCCGCTTGACCACCCACCGGGCAAGTCGTATAGACTCGG
ATCTACAAGCGAGCGTACAGTCTGACGCGGTCTAGGGTCACCTGCATCCGACCTGTTC
TACGCGATGACTCCCGCACCTAC

>c8341_g2

GTCTGTTACAAAAGTGTTGCGCGAAGATCCAACCACAGTATGCGTATGTCGCTTCTTGT
ACGGCATGGTCAAGCTTACCTAGCATTGAAAAACATCTTGGACGAGTCGAATCCATCG
CACGCGAGCGTCCTAGACGATATCAAGCGTCGGTTCAGGGAGGAGACCTTCACGCGA
AAGAGCATTGCACAGGTCATTCATGCTCACGCAGACTTGATCAACCTGCTTTACGTTAA
CTTTGCGATGAGCCATTATCCAGAGACGGACGAGGCGGCGCAGCTCATGCCCACGCTT
TCGTACCAACGTCTCCGCACGCAGATGCCGCTCTCAGACGAGGAACTGTACGATAAGA
TGAGGCGAACCGTCACCAGCAAGCATGAGCTTCAAGTGTTGGAGAGTTTCCTTATCTT
CAACAAGAATGTCCTCAAGACGAACTTCTACCAGCCGACGAAAGTCGCATTGTCCTTC
CGCCTTGCGCCCGATTTCTTGCCTGAGATCGAGTACCCGAGGAAGCCGTTTGGACTCTT
CTTCGTGATTGGCGGCGAGTTCCGCGGCTTCCACATCCGTTTCAAGGATGTTGCTCGTG
GCGGTATCCGGATTGTCATGTCGAGGAACAGGGAGAACTACTCGATCAATCAGCGCAT
GCTCTTCGATGAGAACTACGGTCTCGCGTCAACCCAGTCGCTAAAGAACAAGGACATC
CCAGAGGGAGGTGCCAAGGGAACCATTCTGCCTTCACTCGGGGAGAACCCGAGGCTA
TGCTTCGAGAAATATGTTGACGCCATAATCGACCTCCTGATTCCTGGTCAAACTCCTGG
TATCAAAGAGAAGTTGGTCGACTTGTACGGCAAGCCGGAGCTACTCTTCTTCGGCCCC
GATGAGGGCACTGCGGATCTGATGGACTGGGCAGCGATCCATGCCCGTTCGCGTGGTG
CGGAAACGTGGTGGAAGTCCTTCACGACCGGGAAGAGTGCCAAGCTGCTTGGTGGTG
TTCCCCACGACGTGTACGGTATGACGTCGCTGTCGGTGCGCCAGTATGTCTTGGGTATC
TATAAGCAAGCGGGCCTGCGGGAGAAGAATATCACCAAAGTGCAGACTGGTGGTCCTG
ATGGCGATCTCGGTTCAAATGAGATTCTCTTGAGCTCAGATAAGACGATCGCCATTATC

GACGGTAGTGGTGTTCTCCACGATCCTGCTGGTCTCAGCCGCGAGGAACTCATTCGAC
TCGCGACGCTGCGCAAGCCAGTTGGCTTCTTCGACAAGTCGAAGCTCAGCAAGGACG
GATATCTCGTCAAGGTCGAGGAACAGGATGTCAAGCTCCCATCCGGCGAGGTTGTACT
TGATGGCACTGACTTCCGCAATGGCGCCCACCTCCGTTTCAAGGCTGACCTGTTTGTGC
CTTGTGGTGGACGGCCAGAAGCTGTGAACATATCCAACGTCGCAGCTCTTGTTGACGC
TGATGGCAAGCCCCACTTCAAGTACATCGTCGAAGGTGCCAACCTCTTCATCACCCAA
CAAGCGCGGCTATTCCTCGAAAAACGCAAAGTCGTCCTATTCAAGGACTCTAGTGCCA
ACAAGGGTGGTGTGACCAGCTCGTCGCTCGAGGTTCTCGCCGGGCTTAGTCTCTCGTC
GCAAGAGTACGAGGATCACATGATCTTCAAGGACGGCGAGCCGTCGGAGTTCTACCAG
AGCTACGTGCGTGACATTCAGCAGGCGATCACGGCGAACGCGGCGAACGAGTTTGGG
TGCATCTGGCGCGAGCACGTGCGGCTGCAGGGCGCGAAGCCGCGTACGCTCATCTCGG
ACGAGCTGTCGATCAGGCTCAACGAGCTGCAGGAGGAGCTCGAGAACTCTGATCTGTT
CAACGACGGGCCGAGCAAGCGAGGGGTGATGCGTCGTGCGGTCCCGAAGACTCTTGT
GGATAAGATCGGTCTTGATACGCTCTTGCAGCGGCTGCCGGTGCCATACCAGAGGGCG
CTGTTCTCGAGCTGGGTTGCGTCTCACTTTATTTACAAGTACGGCGTGAACGCGTCGTC
GGTCGACTTTTTCCATTTCGCGCGCGGCCTTGCGGCATAACGTTTATCGGGGGTTGATAT
TGACATGAGATGCGGGTCGGGTTCGCTTTGGTGAACTTACAAGGGAAAGAAATGAATA
GGTTCGTTTGTATGTGTATGACTTGGTGATAAACCTTACAACACAAGTGCCATAATCCG
CATGGGGAAATGGTGTAAATATTTACAGTAGAAAGCACGCGGATGTCAGGTATATCATG
TCTTTGGCGCAAACTGTACCGGAAGATCCTATAGAGCTTATTTCCCTG

>c8523_g1

GCTGTCGGGATTATAACGAAACATGCATTGCCTAACCACCGTGGTCACAGTAATACAAT
TTGCATCCAGCATGGGTACGTAGCCCTCGCCCTTCTGTTCGAGCAGCCTGAATCCCGAA
ATGTTGCATATAGGGCATTGTAGATGAGGAGACCCAGACCGTGATGCAGTGTGATGGTT
AGTCCCTCGTTGCCACCGCGCATGCATCGATGCAGATCATGCAGTGTTGGAAAACTCG
ACGCCTTGCGCGAGCGGGGCTTTGTCCGAGAAGTTGAGCGTGCAGGCGCTCCAGCGG
ACATATTGCTTCCATGCGTCGCCGCCGGACTCGCGACCCCAACCGGTGCTCTTGTTCCC
CCCGAACGCGGCACCGATCTCAGCGCCGCTAGTGCCCACGTTGACATTAACGATGCCC
GCGTCGGACCCAGCAGGTCCGATCCACTTGCCGACATTGCGAATGTCGCGCGTCCAGA
GGCTACTGCTCAAGCCTTGAGGGACGCCATTGTTCCACTCGATAGCCTGCTCGATCTCG
TCGAACACGCCTACATGCAGCACCGGCGCGAACGTCTCCTCGCTCCACACGGGGTCCT
TGACGTCCACCTTCTGCGGGACTGCGATCGTCGGGATGACAAAGTTGCCCCCGCTCAG
TGGCGCCGTGAGGTCTGTGTACGCCGCGCCCCCGGTAAGGATCTTTGCCCCGCCCTTC
CGCAGACGGTCGACGGCGGAGTGGTAGACGCCAACGGCCGCGCGCGAATGCAAGGG
ACCGAGGAGCGTCGCGCGGTCGAGCGGATCGCCGGGGCGGACAGACGCGTAGAGCG
ATTCGAGGCGGGGGAGGAATTTCTCCACGACAGATGAGTGCAAGTAAAGCCGCCGCG
TGGAGGTGCACCGTTGACCGGCGGTGCCGACTGCCCCGAAGAACACTCCTGGGATGG
CGAGATCAAGGTCTGCGTCGGGCATAATGATGACGGCGTTATTCCCACCTAGCTCAAGT
AGGACCTTTCCGAAGCGCGCCTGGACGACCTTGCCAACCTCGCGGCCGACCGCCTCA
CTTCCTGTGAAGGAGACCATGTCGACATCGGAACTTGCAACAACGTCCTTGCCGACGT
CCTTTGCTCCTAAGACGAGCCCTGCAACTGCGCCGGGAACGCCGTTTCGCTCTAATACT
TCAGAAACAATCTTGGTGACTGCAACGGAGCAGAGAGGCGTGCTCGACGCGGGCTTC
CAGAGCGTTGCGTTCCCCGCGGCGAACGAAAGGGCGAGGTTCCAGCCATACACGGCC
ACAGGGAAGTTGAATGCGGAGAGCACAGCAACAACGCCGAGAGGGTTGGGCACTTC

AAGGATGCTGTGGCCAGGTCGCTCGGAGGCTACGACTCTGCCGTTCATCATTCTGGAA
AGTCCGACTCCGTAGTCTGCCATATCCACGAATTCCTGCACTTCTCCTTCACCCTCGGT
CCGAATCTTCCCCATCTCGAGGGAAATTAACGCGCCCAATGCTTCTCGCTTCTGCGCCA
GCGCTTCGCGGATTTGCCTCAGAATCTCCCCACGCTTTGGCGCAGGAACATTTCGGAA
GGTCTTGTACGCTTCTCTCGAACGCTCGAGGGCACGCTGCACCTCCGTCGGAGTGGCC
GACTTCACGCGCGCGAGGACCTCCCCAGTGGTAGGACACACGCTCTCAAGCAGGTCG
CCAGACCCAGTCCATTCGCCATCGTATACGCCGTGCAACTCGGACTCGGTCTGTATGTC
CAGCGCAGAGAGGACAGCGGAAGCCCTAGATGAGAGACCTCTCGAGAGCGGTGCGC
GCCGCAGAGATGAAAGTGCCCGCCTGGAGATCATGATGGTATGAAACAAACCGCCTCC
GTTCTTGAAGATGCGTCGGAGAGCATGATCCGCCCGAGCGCCCTGCCCGACGCTTGAT
ACGCGCGGCGGTTACTACGTGCGTGTGCGTGTCTTCCGAACGTCGAAGGGCGTCGTTC
GCGCAATCGCACTGGTAATGTATGGCATTTGAATACTATCAAAGGTCGGCAAAGAAAGC
TGGTTCAGCCTCAACCTCATTCCTGCCCTTCTTCCGCTTCGTCATCTGCAACGGATCCTT
CTCTTTCTTCCTCTTGCGCTTCTTCTGCTCGATTTCCTCATCGCTACTCTCGTTCTCATCT
TCTACATCTCCTTCCTCCTCGTTGACGAGTCTCGGAGAACTTGCTTCCTGCTGTTCCTC
CTCCTCTTGGTCCGAATCACCTGTGTCACTGCCTTCTGCCTCCTCGTCGGATTCTTGCCC
CTTGTCGCTGTCCTCTTTCTCGTCATCCTCAAACACCTCTTGAAGGTCTGTCAACTTTA
GCACCTCCTCATGACCCACACTCCCAACCCATCGCCCCTCGCCATTCATATCAACCGCA
ATCCGCTCGATAGGGAACTCTCCGTGGTCGGCAACAACCCCGAGTAGCTTAGTCGGGA
ATAACTGCACGGCACGCAACAGTCCGTCCGATGACCCAGTGAGTATGGTCGACTGGGC
ATTGGGATAACGAGAAGGTATAGAGCAGAGCGCGTCAATGGATTGCGGGTGACCAGGG
ATGCGATCCACGCAGTCTCCCCAACCACTTTTGCGGTTGAATATCGAAAGAATCCCGAG
CTGGGTTCCCACAACGACTTTCTGTCCGCCTTTGATTGGCAGTATTGAAAGCAATTCGT
CCTCTTGGTCCTCCGATTGCGCCACGGGCTCCACCTTCTTTGACCGTACATCCATCACA
GAGAGCGACCCATCACCGCTCGTCGCAACGAGTTGCTTCTTGTCATTCAGCCACATAA
AATCCGAAATGAAATCAAAATGGTGCTTGTACTCGCGCACGGCATCCGACTTGCGTGG
GTCCCACAACTTCAACAGCAAACGTCAGCTTCCCTTGACCACTCCGTCATCATCGCCC
GAGGCGAAGAGCTGCGGCAACAATCGCTTCAGTCTGTTGATGGGTACGCTTCGCGCGC
ACACTACCCAGGTCAGCGACCACGTTTCCTTCGACGAGCATAGGATAAATGCGGACAG
ACTCACTCGTGTGCAGCAGCGCGCGTATCGACTACCTCGCCCGTGCCTACGTCGATCGT
GCTAACGTCCGTTCTGAACGTCGCTTGATCATGAGTTACGAGCACAAGACGTACTGAA
TAGCCTTCGCTTTCCCTCCTGCCCAGAGTTGCGAACCGTCCTTGCTCATCGCGAGCGTG
CGACATGACCGCTTGGTAGGCCGAGTCGCAAACTTTTCTTCGTGGTTACCCTGCTCATG
GTAGCTGAAAGCCTTGATGTGACCGGTGAGAAGACCACTAAATACAAGCGGTCTCTCT
GGGTGAAACGCGAGATCGAATATCTGCGCGCCGACAGGAATGTCGGGCATGGTCAAG
GGAGAGAAGAAAGGTTCGAGGCGCCCACGCTAGAAATTAGAATTGAAATCTATAGTAT
CCGTTCTACAGAGCTG

>c1893_g1

GGGGTTCCTGAGATTAGTTCTGGTTTGAGGATGAGTGTTAGTATGGGGATGATGTGGAG
GGTTATGAGAAGATGTTCTCGAGTGTTTGAGTTGGGGGTTGTTGTGATGTGGGATGGA
AGAGTGCCTCGTTGGGTTGAGAGTAGTATGTAGAGGGTGTAAGAGGCGGTTAGGAGTG
TTGCGGTGCCGGTTAAGATAATTGTGGGGGAGGATCAGTTGAAGAGGGCAACTATAAT
TGTTAGTTCTGCTATCAGGTTGGTGGTTGGGGGCAGGGCCATGTTGGTTAAGTTGGCTA
GTAATCATCATACTGATATCAATGGTAGGAGGGGTTGGAGGCCTCGTGTCAGGATAAGA

ATGCGGCTGTGTGTCCGTTCGTAGTTTGTGTTTGCTAGGCAGAATAAAAGGGAGGAGG
TTAGTCCATGGGAGATTATGAGGATTATTGCACCTGAGAATGATCATTGGGTTTGGATTA
TACTTGCAGCGATGACTAGGCCCATATGGCTTACGGATGAGTAGGCAATGAGGGATTTT
AGGTCTGTTTGGCGTAAGCAGATAGAGCTAGTTATTAGGGCACCTCATAGGGCTAGGGT
GAGGAAGGGGTAGTGTAAGAAGTTGGATACGGGCTCCATTAGGAGGGTGACTCGTATG
ATGCCATATCCGCCTAGTTTTAGTAGTAGAGCAGCGAGTAGTATGGAGCCTGCGATTGG
TGCTTCTACATGGGCTTTAGGCAGTCATAGGTGTAGTCCGTATAGGGGTGCTTTGACTAT
GAAGGCTATTAGGAGAGCTAGGCTAGATAATAAGCTTGTTCAGGAGGCAGGTAGGTTG
GGGTGGGTGAGTTTGATAATGGGTAGGTGGAGGGTTCCAGTATTTGTGTGAAGGTAGA
GGATGGAGACTAGTAAGGGAAGAGAGCTAATTAGGGTATAGAATAGAAGGTAAATGCC
TGCGCTAAGTCGTTCTGGTTGGTTTCCTCAGCGTGTGATTAAGATGAGGGTTGGGATTA
GGGTTGCTTCGAATGAGATGTAGAATAGTATGAGTTCAGTTGCTGAGAAGGCTAGGATG
ATGAATGGTTGGATGATGATTAAGGTGGAGATGAATATTCGTTTTCGTTTGTGGGGTTCA
TGTTGTAGGTGGCCTTGGCTAGCTATAATTATGAGGGGGAGAAACCAGCAGGAGAGTA
CTAAGAGGGGAGTTGAGATTTGGTCTATGCCTGTTCAGAGGGTTAGGGTTTTTGTGGG
GTAGTATGATGGGGTTAGTCAGTGTAGGCTGATCGAGGCGATTAGAAGGCTGTATATTG
TGGTGTTAGTTCATATGGATTTTGCTGGGGATAAGAGGGCTGTGGGCAAGAGTATGATT
GTTGGTAGGATGATCTTTAGCATTGTAGAAGGTTTAGGTTGTGTAGGTGGTCAGAGCCA
TGTGTTCGTGCAGAAGCTACTAGTATGGCTAGGCCTGTGCCAGCTTCGCATGCTGAGAA
GGCTAGTATTAGAATAGGTACAAGGGCGAATGATGGGGTTTGGTTTTCTACTGGTCAGA
TTGAGAGGGGGATGAATATGGATAGTATTATGCTCTCTAGGCATAGTAGGGCAGAGATG
AGGTGTGTTCGGTGGAATGCTAGTCCTAGGCTGCTGAATGTGAATGCAGAATAGAAGC
TGAAGTGTAGGGGAGACATAAGAAAGTTATAGGTGGGTCTATAATTTGCT
>c7209_g1
ACTTGTTTGTCCATTTTCCTGCGACGACAAGGACTCGGCAATGGGCCTGAACCTCGGT
TTCCTATCGCCTCGCTTGGCTCATTTTACTATTTACAAGCCACATGTAATGCGCAAAGCG
CGGCCACTTTGTGTACAGGCGATCGATTCAATAACACCTCATTCATCGCGTCAATCCAA
GACATGACACGGTGGCCTCTTCGACACGGACCTAGACACTTCTGCCACACGATGTTTG
CACCTCGTGGTTGTTGACCCATCTATTGCGAGAGAGAGTCGTCGTGCGAGCATTCCCA
GGTCAGTCATAGTGTCGGGTGCAGCGTGTGCCAAAGATCACAGGTCCCGCTTTATCGC
CGACGCGCACAGACCTTGTTGGGAATGACAGGAGGAATTGACGCAAGCGACGCTGTC
CGAACAGCCTCGCACAAAATCGACTGCTGGAGGGCTTGGCAGACAATGCCAAGCCTG
TCGCGTCTCCCCAACTGCCAGATGCGGACTTTACCTTGCGTAATTGCGCGGACAGAAC
CTTAACGCCGCACACGACGCCCGTTGGGAGGATCGTCGTCAGAACCCTCTCAGACGCG
ATTCTCACCACGTGCGGCCCATCCCGACATGGCCTCCAACTCGCCATTCCATTCTTTTT
TATTTAACCATCCTTCCCTCAGCTTATAAGCCCTACTCCCCTACACCACATCCACATCTC
CACTCCCTTCCCTCATCAAACATGCCCGGTCTTGACCTCTTCAGCACCGCCTCCATGCC
CAGCAGCACCATCGTCGAACTCCCCACATCCACCCTAGAGTCCTCACATCTTCACCCA
AACGATACATCAGACTCGGAGCCCACAACACCTTCGACTTTGGAACATGAACACCCCC
TAGTACCAGTTCCGCGCAGCCCCGGGGCTCCCGCGCCCTACTACAACCCCCGTCTCGAT
CCCAACAACTTTCTCGAGGGCCCGCTCTCGTGGAATCCCGCAACGCGCCTCCGCCAGC
TACTTGCACGTCCCGGCATTGTCGTTGCCCCGGGTATATGCGACGGCATCAGCGCACGG
TGTGCACTCGAGGCCGGCTTCGAGTGTTTGTACCAGAGCGGGGCTGCAACAACCGCAT
CACGCCTTGGCCAGCCCGATCTGGCGATTGCAACCCTGAACGACTTTGTCCAGAGCGC

ACAGATGGTCTGCAGCCTTGACCCCACCTGCCCCGTCATCGCCGACGCAGACACCGGG
TTCGGCGGTCCGGCGATGGTTGCGCGGACAGTCACGCAGTATGCACGGGCGGGCGTC
GCTGCACTGCACATCGAGGACCAGGTGCAGACGAAGCGCTGCGGGCACTTGATGGGC
AAGCAGGTGGTATCGCGCGAAGAGTTCATCACGCGCATACGCGCGGCGGTCATTGCCC
GCGACTCGATACCTGGCGGGTCTGACTTCGTGATCATCGGGCGCACAGACTCGGCTCA
AGTGCTCGGGATGGACGAGGCACTGATGCGGCTGAGGCTCGCCGCCGACGCAGGCGC
GGACGTGTGCTTCATCGAGGGCGTGCGCACCGCCGAGCTCCTCTCGTCCACCGTCGCC
GCGCTCGCGCCCAAACCCGTGCTCGTCAACGTCATCTCGGGCGGGCTCACGCCGTCGT
TCACGACTTCTGAGGCTGAGGCGCTCGGGGCCAAGATCATCATCTTTTCGCTTGTGAG
CTGCGTGGCGGCTGTGCATGGGATCAGGGCGGCGATGGCGTCCCTCAAGAAGACGGG
AACAGACTTTACGAGCGCAAAGGGTATGGACCCCAAGGCGTTCTTCGAGGTCATGGGC
TTGAAGGATGTTGTCAAGCTTGACGCGCAGGCTGGCGGCAAGGCGTTCGAAGCGGTC
TAGATTGGAGTGAGTATCAGCGACGCATCCGCTCCTTAAGTGCGCTTGCGCGTTGTCAT
TGGCCAGACGTCACACCGTGGATGAAGACCTCTGCCTTAGAGAGAGCTCGGTCATCAA
CATCAACTATCCCCGCCGCTGCCGCTTCCGCTGCATCACGTTTCTTAATGTTATTCGTGG
TTATGGGTTGCGTCATTGTCATTCTATCATAGATCTACGTCTCATTGTTGTACAACCTCGT
CTTCTTTCCCTCGCATCTTTTTTCATTAGTGCTCCCATTTCTCTCTCTCTTTCTCCTGTCT
GCGCATGGGTTTTATTTGGTGATCTTGGGATGACTCAGTCTACATACGCGAGTTTTTAC
ATCATATTTTTTTTCTTTATATCTCATTGCAAAAACCGACGCGTCCGTCGGAATCTTGTAC
TACACCAATGACATGAAAATGGTATAGCTATAGAAGCGTAAGCTCGTGCCTGCCCCATG
GCCTACAGA

>c6742_g2

CCTTCCACAGCACCTCTCATCATGATTATCCCAATTCCCAGCCCCCCTGCAATCCCATTC
CTCGGACATGTCAACTCTCTGGATAAAGACCTCCCCTTGAAGTCTCTTAATCTCCTTGC
TCAGCAATATGGCGAAATCTACCAGCTCGTCATCTTCGGGAACAAGAGAAACTACGTAT
CTTCCTACGACTTGGTCAATGAGCTTTCAGACGACAAACGATTCCCCAAGAAAATCAA
TGCTAACCTGCATCAAGTACGCAATGGTATCGGAGACGGTCTCTTCACTGCTCACTACC
CCGAGGAGAAGAATTGGGCTATCGCTCGTAAGCTTGACTCCTTAGTTAATCAAGCAGC
AGATCTGGAGCTTAGACTGAGGGCTATGTGATAAGATCGCCTTCTCATGCCGTGCTTTG
GAACGGCCAGTATCTCCAACATGTTTGACGGCATGGTTGATATTGTCTCCCAGCTGATT
CTCAAATGGGAGCGCTTCGGGCCGAACGACGTTATAGATCCTGCTGCAGACTTCACCC
GACTCACCTTGGATGCCATCTGCTTCTGCGCCATGTCGTATCGGTGCGCTCGCTCGCTG
ACCCTGCGGCATATCAATCGCTGACATTCGGCGTTCTTCCTATTTACGTCCGCACAGTTT
GAACTCGTTCTACCGGGAGGAGCCGCATCCGTTTGCGCGCGCAATGGCGGACTTTTTG
TTGGAGAGCGGCCTTCGTGCGAACCGTCCGTCACTTGTTCAGGCCATCATGACCGGTA
GCAATGCCAAATATCAGGAAGCCTTACGGATCATGTCCAAGCTAGTCGACGAGATCTTG
CAGGATCACCGTGCGAATCCTCCTGAAAGGGATGATGTTGTCCAAGTCATGTTAACTG
GGCGAGATAAGGAGACGGGTCAGGGTCTCCCTGACGAGAATATCAAGTTTAACCTCCT
CACATTTCTTATCGCTGGCCATGAGACCACTTCTGGAATGCTCACGTTCACCGTGTATC
ACCTGCTCAAAAATCCCGAGGCGATGCGTAAACTGCGCGAGGAGATCGACAACAAGA
TTGGCGACCGAGTGATGACGGTTCACGACATTGGCAAACTGCCCTATCTTCTAGCTGTT
ATGCGGGAAAGTCTGCGCATGGACCCTCCCGCCCCAATGCGAACAGTTATGCCGGCCG
AGGATACGGTTCTTGGTGGCAAATACGCTCTCGAAAAAGGAGCAACCATAATCATCAA
CGCATACGCTCTCCATCGGGATCCGAAGGTCTGGGGAGAGGACGCAGACAAGTTCCG

GCCGGAGCGCATGTTCGATGGCAAGTTTGAAGCACTGCCGCCGAATGCGTGGCAGCCA
TTTGGCTTTGGGATGAGGGGATGCATCGGTCGTTCATT
>c7929_g2
TCCTCGCCCAGCTGGTCCTCATCGTGCACAACAACATTAGAAGAGAGTGTAAGAGGGA
ACAGTACGCGTTTACGCTTTTTACCCGAGGCAAGGTTGGAGGTACGCAGGATGCGTAC
TGTAGCCTGTCGATTCCGATGTTGAAAAGCCAATGCCTATCAGAAGCGTTTGCAGAATT
AAAGACAGCAACGGGGTACGAAGTCATTCCCTGGAATGTTTGTCGGGCTGGCGGAGAT
CACCGCTGGGTTGATCAACACAGATGGTCTAAGTACTTGATGAACCAGCTGCCAATGTT
AGAACCAGCTGCCAATGTTAATCCGTTCTGAATTCAACAGAGTCAGATGCCGCGCACC
TCAGTTATGAGAGCAACTATGCTGGAAGACCGTGGAGGAGTCAGCTCACGAGCCAAC
ATGAATATAGACACAATGAAGGTTGACACTTGCGTGTTAGGGTTGCGGTTAACAGGGA
CGTTTCCTACAAATCTCCGCGGGAGGTGGTCGGGTGTGTTAGGCAGCTTTCGGTCACC
TCGAAGTATGTTTGCCATTCAGTGCTAAAGCTAAAGTCGCCGGACCACAAATGGGGGA
CAGAGTTTGCTCTTTAGAAAAAAAAAATGACTCACTAAGGGGACAGCAGAAGTATACA
TAGCGCGGGGCGCGGTCTCCCACAAAGAGGTCGATGTATGCGCATTGCGACATTGCGA
ATGAGAAGGGCAAACACCTGCAATGTTGCAAGGAGGCTGGCACCAAAGGGTAACTCC
CCCATTTTGAGGGGGGCGAAAGCAAAGCACCCTGACCCTGGGACTGACTACATGTTGA
ACGTTGAGTGGGGACTGACTCAGATAACTAACTAACTTTCTCACAGTAAGTGTTCTTTT
CCACACAGACCTTATCTCGCTGGGACGTGCTATTCTGGACGGCTTTCGCTCGTGCCGAC
GCATTCATATCACGATGAGATCGTTCAGATAACTCGACGGGCCTCCACACGGTCCTTCT
CGAGCTCTAATATACAACTCTTGGGAGGGCATACACGGTCAAGATGTCTTCGACCACGA
ACAGCCCACCCCCACCCCCTGGAGTTTATGTCCCGGCGGTCGTCTTCTTCGATGAGAA
CGAAGAACTTGACGTGCCGTCCATTACAGCTCATGTTCTTCGCCTAGCCCAGGTATCTT
GTACTCCGACATGCAACAGGACGTCGATCCCACTGAATTCACTACCAGGGCGGAGTGA
CGGGCATCCTAGTGCAAGGGAGCAATGGCGAAGCCCAACACCTCTCGCATGAGGAGC
GCAAGGAGACTATTCGTCTCACCCGCGCGACTTTGGACGGGAATGGGTTCCAAAACGT
GCTCGTCATCGCAGGCACGGGCGCGCAATCTACAAAAGAGACCAAGAAGCTATGTGTG
GACGCAAAGGAAGCAGGCGCAAGCCATGCGCTCGTGCTGACACCGGGTGTATGGCCT
CCGCAAATGACAAGGGAGGTCATCATCAGCTTCCATTGCGACGTTGCAGACGCGTCCC
CAATCCCCACCATGATTTACAACTTCCCGAGGGTGACTGCTGGGATAAACCTTGATTCC
GATATCATCAGCTTGCTTGCCGAGCATCCCAACATCGTCGGCACCAAGCTCTCCTGCGC
AGACATTGGCAAGCTGCACAGACTCACCTCTACCCTCTCCCCATCCAAATTCGCCACCT
TCCCCGGCGCCTCAGCAGTCTTCCTGCAAGGGCTCATGTCCGGAAGCGCGGGACTTAT
CGGGGCACTCCCGAACGTCGCACCCAAGGCTCATGTGGAGCTGTATAGGCTGTGGAAG
GAGGGCCAGATGGGTGCGGCGGTAACGCTACAGGCTTTACTCGGGCATGCCGATTGGG
AATTGTCGAAGCTGGGAAGCATTGCGGGGATCAAGGCGATCGTGACAAAGCACTTTGG
GTATGGGAGCGGAAATGTGAGGGGCCCGCTGCCATCCCGCGACTTAAAAGGCGCTGCA
ACTGTCAAGCTGAAAGAGTTGATCGCCTTTGAGAAGACTTTGTGATGTCGATGCAAAA
TCGCTCACGCCAATGCACGCACAATTGTTAT
>c3657_g1
CGGTTGCCTTGATTGATTCTCGATTATACCCAAGTGATAGTACATGCTGCTCATCGGCGA
CGAGCGTATCAAACAATTCGCGACTCTATATGTCTTCACTGCTTCGGAAACCCGACATT
GGATTTGCCCTTCCAGCCCCACTCTATCTGATTTGCAACCTTCAGTCTCACCCCCAATTC
TTCTGCAAACTGCCACACAGTAGCAGGGTTCTTCTGGAACTGACGTCCAACCAATACA

GCGTCGGCCTGACCCTTATCAAGGGCGCCCTGGGCGACATGACCGTCCTGGATACCTC
CCACAGCGCCGACGAGAATCTTGTCACCATGTGTCTTCTTGACCGCTTCGGCGAAGGG
GGTTTGATAGGCGGGTTCAACGCCAACTGGGCCAATCTTCTGCGCTGGGTGAAGCCCT
CCGGTCGAGACATCGAGGAGGTCAACGCCATGTTCGGCGAGGATGCCAGCGAAGCGC
ACGGTGTCTTCCGAGCGCCACGAAGGCTCGTTGGGGAGAGTCTCCTCGAGCCAATCA
GTTGCAGAGATACGTAAAAACAGAGGCATAGTCGGCGGAATGATAGCCCGCACAGTGT
CCACAATTTCGAGGCTCAGACGAATGCGGTTTTCAAAGCTACCGCCATATTCATCGGTG
CGCTTGTTGCTGGCAGGACTGATGAACGAGTGCAGTAGATACCCGTGTGCATTATGGAT
CTCGATGACATCGACACCGGCCTTCAAGGCGCGTTTCGCTGCGGCGACGAAAGCGGCT
ACAATTCGCTGGATACCCGCCTTGTCTAATTCGTTTGGCTTTGGGTAGTCGTCGTTGAAT
GGCAGCACGCTCGGGCCGAAGACGTCGTTGGGCCAGCCACCCACAGCTTCTGTCGCG
ACGGTGTTCCTGCTCAACCACGGCGCGAGGGTGGAAGCCTTGCGGCCTGCATGCGCA
AGCTGGATGCCTATCTTCTGATTCTGCGCATGCGCGAACTGGGCGATGCGGCGGATGGG
CTCGATCTGATCGTCGTTCCAGAGTCCCGAGTCTTCTGGCGTGATGCGTCCTTCTGGTG
TGACCGCGGTTGCTTCTATGAATGTGAGACCAGGTCCGCGCGTGAAAATACCTCCCAG
ATGGGCCATGTGCCAGTCGGACAGCTTGCCATCCTCGCAGGAATACTGGCACAAGGGA
GAAAGGAAGATGCGGTTGTGGAACTCTACGCCTCGGATCTTTAATGGCTGGAAGAGCG
TCGGGATGGGAGAACCCTCGTCGATGGCTGTACCTGAGGGAGGATACTGAGCGGGGGT
GAAGTAGGGAACGTCGGGAGCGGGGATGTTGAAAGTGCTGGCCATGTTTTTTTCTCTG
CTGGAGGCAAGTCGACGGCCCTCTCTATATATAAAGCTCTCGAGGTTTGGTTTGTAATA
ACGACTGCGAATAACTATTGACGCCATTTATAAGTATCTTAGTCAGGGTGGTCGACCAG
TCGGGCAAACCGGG

>c5369_g1

TCTTGTGAATCTCGGATGGAAGTCCCGGCGGGTCTACGCATGTGCATATGACCGTCATT
AGTACCGCTGAGTGTGTCAACGGTTGCTTTACTAGCGTCTCCATTTGCATCGACCTCTT
CATTGAAGGTTCAACTTTGTCGACCTTATGACATCTATGATTTATCGATACAGGGCTTCT
CGGTCTCGTATTCGTATTCTTGTCTCTTATGTGCTATTGCTGACGGTGCCTGCGTATAATT
CAGTCGTCTGTATATTCAGTCCGAGTCATCTTTTCTTATATGGACACGCAGACAACCGTG
ACAATGCTAGGGCCGCCTAAATGGTAGAACAGCCTGGTGCTAGGCTGCTAGGCAGTCA
TCAGATAGCAGCGGGATAGGTGTCGCGCTTCCAGCTGGCAGTACCACCGCCATCGTGC
TCCAAATTGGCAGCGCCTGCATGTTCGCGCTTTTCCACGCCAACAGCAAGATTGTTGAT
GGGCTCAGCGACGACGAGGCTGGCAAATGAAACCGCTAAAAATGTAGAGAAGAAGGC
GTGGAAGCGCATGGTTGTTGTACTGAGGGGATAGGAGAGAAAGGAGAGCAGAACTAT
TTGTATTCTGTTCGTATCGGCCTTGATAAGTACATCCGACGGCCTGATGTTTGAGCTCTA
ACTCTGCACTCATGGTTGCAATAATATTATCGCATGACAAGATCGCAACGCCGAGGGGG
TATGTGCGATTTGTTACTGTGAGCGAGTTGTTACTGTGACTTGAGGGAAGCTACAAGAC
GAGTGGCGCCAGCCTGAGTCTGCTAGGAATTCGAAAAACTG

>c3191_g1

CTGGCACGCACATCTCCGGTGCAGAGCGATAGCCGATATGCAGCAAAGCGTCTATCATA
TTACAATATTACAGCGATACAGCAAACAAGAGACAGTAAGACAAAGACTAAGGTATAC
ATGGCTGGGCCGGATGAAGGGGAATCTAAGCTGCTGGCAAGGACAGAGGAAGGTCAG
TATTCATCTACCACCCAACAGTCGTCTGGGATCGCATACGCTGAGGCCTCTTAGCATCCT
CTTGCTGTCTCTCAGCGTCCTGCGTGCCTTCTGGAGCGGTCGTTGCTGCTTGAATCTTC
TCCTGTTCGCTCTCCTGAGGCTCTGCCTCCTCCTTGGCGTTCTGGAACGGATTTGCTTT

ATCTTCTGCCTCCATATCCTCTCGTTTTTGTTCCCCGGAGTAGATCTTATGTCCCTTCGCC
TCGAGGTCGGGGTCACGAGCTATTTTTCCCTTTGCGATATCTGCCGCACCCGCGATCCT
GTCACCCAGAGTCACGCTGTGCATTTGTCCATAGTTAGGTCCTATATCGACTGCGCCAG
CGTGGAGTTGCCGGGGGTAGGTTTCATCATTAGTTTGACCTGGATTCGTGTTCGTGTTT
GAAACATCTCCGTCAGGCTGATCAGACGTAGACCTGCTGTTGGGTTGTGGAGACGGGG
ATGATACCTGGTGAGTCGACATCGTAGTGGCTGGACGTGTATAGAAAATCAAGATGGGT
GCAACGGCGCGGGGGTGTCCGCGCAAGTAGACCACTGTTATTTATATTGCAAGGTTAAT
AATAGCGACAGTTGGAGAGACTGATGACGCAGTGGAGTGTGGGGGGACAATATGACG
TCGGTATTATCATCCCGCCCTGATGGTAGCTCCAGAAGAGGG

Supplementary Table 5. Primer sequences used in qRT-PCR amplification.

DEGs No.	Forward	Reverse
c43_g1	TGTATGAGCCCACCACATATTC	CACCGGTAGGAATTGCGATAA
c3657_g1	TTTCGAGGCTCAGACGAATG	CCATAATGCACACGGGTATCT
c9143_g1	CGGCAGAGTAAGCGATAGATAAG	CCTGCTGCACAACTGTTAGA
c9468_g4	TGAAGTTGAGCGGGTTAAGG	GCGAGAGCTTGGTGTTATGT
c10001_g1	CAGGTCCTACGTCAACATCTAAC	CGAGAGAAGAGAACTGGAGAGA
c4703_g1	TGCAACAGGTGGATGATAAGG	TTGACTATGCCCGTGGAAAC
18S rRNA Previously identified	TGAGAAACGGCTACCACATC	TCCAATTATGAGACCCGAAAGAG
